# A computational model for classification of *BRCA2* variants using mouse embryonic stem cell-based functional assays

**DOI:** 10.1038/s41525-020-00158-5

**Published:** 2020-12-08

**Authors:** Kajal Biswas, Gary B. Lipton, Stacey Stauffer, Teresa Sullivan, Linda Cleveland, Eileen Southon, Susan Reid, Valentin Magidson, Edwin S. Iversen, Shyam K. Sharan

**Affiliations:** 1 Mouse Cancer Genetics Program, Center for Cancer Research, National Cancer Institute, Frederick, MD 21702 USA; 2 Department of Statistical Science, Duke University, Durham, NC 27708 USA; 3 Leidos Biomedical Research, Inc., Frederick National Laboratory for Cancer Research, Frederick, MD 21702 USA; 4 Optical Microscopy and Analysis Laboratory, Frederick National Laboratory for Cancer Research, Frederick, MD 21702 USA

**Keywords:** Genome informatics, Genetic techniques

## Abstract

Sequencing-based genetic tests to identify individuals at increased risk of hereditary breast and ovarian cancers have resulted in the identification of more than 40,000 sequence variants of *BRCA1* and *BRCA2*. A majority of these variants are considered to be variants of uncertain significance (VUS) because their impact on disease risk remains unknown, largely due to lack of sufficient familial linkage and epidemiological data. Several assays have been developed to examine the effect of VUS on protein function, which can be used to assess their impact on cancer susceptibility. In this study, we report the functional characterization of 88 *BRCA2* variants, including several previously uncharacterized variants, using a well-established mouse embryonic stem cell (mESC)-based assay. We have examined their ability to rescue the lethality of *Brca2* null mESC as well as sensitivity to six DNA damaging agents including ionizing radiation and a PARP inhibitor. We have also examined the impact of *BRCA2* variants on splicing. In addition, we have developed a computational model to determine the probability of impact on function of the variants that can be used for risk assessment. In contrast to the previous VarCall models that are based on a single functional assay, we have developed a new platform to analyze the data from multiple functional assays separately and in combination. We have validated our VarCall models using 12 known pathogenic and 10 neutral variants and demonstrated their usefulness in determining the pathogenicity of *BRCA2* variants that are listed as VUS or as variants with conflicting functional interpretation.

## Introduction

Breast cancer is one of the most frequently diagnosed malignancies in the world. As per GLOBOCAN 2018 estimates, the number of new cases world-wide exceeded 2.08 million and there were more than 600,000 deaths^1^. In the United States, breast cancer associated mortality has been declining gradually since 2001 due to improved treatment options and availability of cancer screening tools (https://seer.cancer.gov/csr/1975_2014/)^2^. In addition to mammogram-based screening, there is a marked increase in sequencing-based genetic tests to identify individuals at risk of developing the hereditary form of the disease. Mutations in *BRCA1* and *BRCA2* are known to significantly increase the risk of hereditary breast and ovarian cancer (HBOC) (https://seer.cancer.gov/csr/1975_2014/)^2^. Risk of other cancers such as prostate and pancreatic cancers are also increased in *BRCA2* mutation carriers^3–7^.

Sequencing-based genetic tests to screen for mutations in *BRCA1* and *BRCA2* are recommended to individuals with a personal or family history of early onset and/or bilateral breast and/or ovarian cancer, or a history of male breast cancer. Sequencing of *BRCA1* and *BRCA2* has resulted in the identification of more than 21,000 variants that are listed in ClinVar (https://www.ncbi.nlm.nih.gov/clinvar/). Recent efforts to have a more reliable and accessible record of variants resulted in the establishment of BRCA Exchange (https://brcaexchange.org/) by the Global Alliance for Genomics and Health (GA4GH)^8^. BRCA Exchange has combined the information from ClinVar, the Breast Cancer Information Core (https://research.nhgri.nih.gov/projects/bic/index.shtml) and Leiden Open Variant Database (https://www.lovd.nl/) as well as a number of population databases making it the largest open source of information on *BRCA* variants. Currently, BRCA Exchange lists 40,331 unique *BRCA* variants.

Determining the functional consequence of variants that result in a single amino acid change or small in-frame deletion or insertion is challenging and a major hurdle in their risk assessment. A number of in silico models have been generated to aid in predicting the impact of amino acid change on protein function^9–13^. Epidemiological studies provide the most reliable information to classify the variants as neutral or deleterious based on co-occurrence in trans with a known deleterious variant, detailed analysis of personal and family history of cancer in probands and co-segregation of the variant with the disease in families. Because most variants are rare, very limited, if any, epidemiological data is available for them and they remain unclassified and are referred to as variants of unknown clinical significance or VUS. To date, more than 2800 *BRCA1* and 5000 *BRCA2* variants are reported as VUSs in ClinVar. In BRCA Exchange, only 7445 variants out of more than 40,000 variants, are classified by the Evidence Based Network for the Interpretation of Germline Mutant Alleles (ENIGMA) expert panel^14^.

In recent years, the value of functional assays in determining the impact of VUS on *BRCA1* or *BRCA2* function has been widely recognized. Significant effort has been devoted to developing assays that have high specificity and sensitivity. Such assays include those that examine the *BRCA1* transcriptional activity, or functional complementation assays in *BRCA1*-deficient or *BRCA2*-deficient hamster or human cancer lines^15,16^. A CRISPR–Cas9-based high throughput approach of saturation genome editing has been utilized to examine the effect of *BRCA1* variants in HAP-1 cells, a haploid human cell line^17^. More recently, *BRCA2* variants were expressed in *BRCA2-*deficent DLD1 cells and their response to multiple PARP inhibitors (PARPi) was used to functionally classify them^18^. We and others have utilized mouse embryonic stem cells (mESC) as a model system for functional evaluation of *BRCA1/2* VUS based on the observation that both genes are essential to the viability of mESC^19–28^.

A standardized five tier classification system is used to classify variants based on their likelihood of pathogenicity^29^. As per the American College of Medical Genetics and Genomic (ACMG) guidelines and the recommendations of the International Agency for Research on Cancer (IARC), class 1 and 2 designations are used for variants that are “not pathogenic” or “likely to be not pathogenic”, respectively and 4 and 5 for variants that are “likely pathogenic” and “definitely pathogenic”, respectively. Variants whose functional significance remains uncertain are class 3 variants (http://www.lovd.nl). Based on the recommendations of ENIGMA and ACMG, variants evaluated by a functional assay should be classified based on their impact on function. As per the guidelines, variants are classified as “functionally normal” or “functionally abnormal” based on the results of any functional assay^30,31^. “Functionally abnormal” variants can be further characterized as complete loss-of-function, partial loss-of-function/intermediate effect/hypomorphic, gain-of-function, dominant-negative^30^. In this study, we have followed these guidelines for functional characterization of variants evaluated by our mESC-based functional assay.

The ultimate goal of any functional assay is to obtain reliable information on the impact of the VUS on the tumor suppressor function. However, the results of most functional assays are not binary. Due to experimental variabilities and the hypomorphic nature of several VUS, the measured functional activity spans a range of values between the positive (wild type (WT)) and negative controls. This makes it challenging to classify variants using the results of functional assays. To address this, computational approaches are being utilized to deduce the pathogenicity of VUS. The Bayesian hierarchical model referred to as VarCall was first utilized to classify *BRCA1* VUS using the results of an in vitro transcriptional activation assay^32^. A similar approach was subsequently described for classification of *BRCA2* VUS using a homology directed DNA repair assay^33^.

**Fig. 1:**
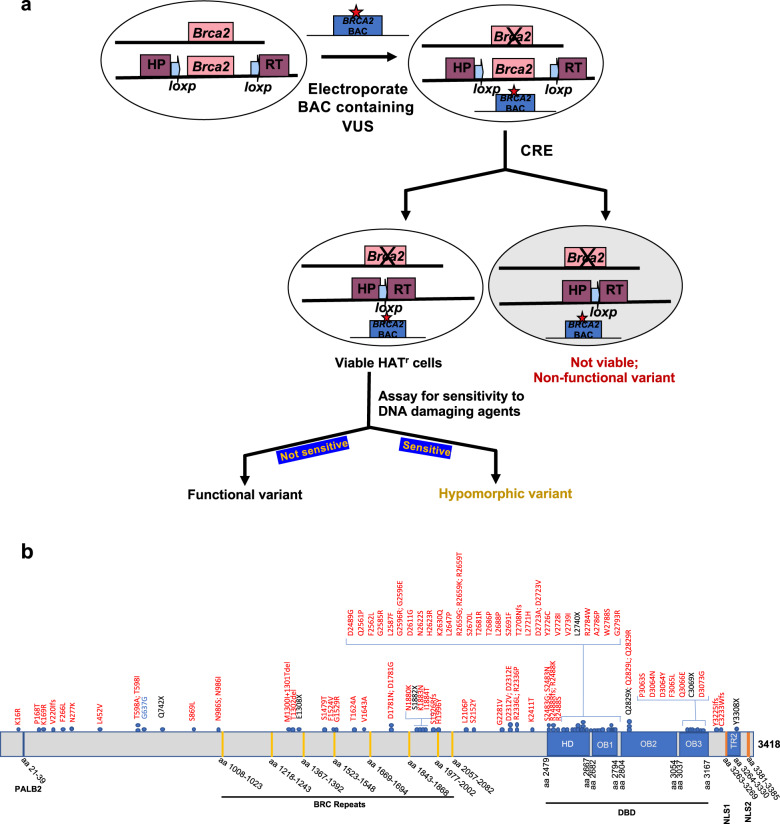
Mouse ES cell-based functional assay for *BRCA2* variants. **a** Schematic representation of the functional assay. BAC DNA encoding human *BRCA2* gene with any variant was introduced into PL2F7 mES cells containing a conditional allele and a knockout allele of *Brca2*. Conditional allele was further deleted by CRE and the recombinants were selected on HAT containing media. Depending on the impact of *BRCA2* variants, HAT^r^ cells may or may not be viable. Viable HAT^r^ cells were further tested for sensitivity to different DNA damaging agents to distinguish between variants that have no impact on function and those that have some loss of function (hypomorphic). Star in the BAC construct represents variant. Two halves of *HPRT* mini gene are marked in solid boxes as HP and RT. Solid arrows denote *loxP* sites. **b** Schematic diagram of BRCA2 protein with different domains and position of variants selected. Different domains are marked as colored boxes and the amino acids (aa) for the respective domains are noted below. HD helical domain, DBD DNA-binding domain, OB oligonucleotide binding fold, TR2 C-terminal RAD51-binding site, NLS nuclear localization signal^15,43,44^. Variants selected for analysis are denoted as colored solid circles on top. Missense, silent or synonymous and nonsense variants are marked in red, blue, and black colors, respectively.

In this study, we have developed a VarCall model for predicting the probabilities of impact on function (PIF) of *BRCA2* VUS using the results obtained from our mESC-based functional assays. In this assay, variants are generated in a bacterial artificial chromosome (BAC) clone containing full-length human *BRCA2* by recombineering and their impact on cell survival and sensitivity to DNA damaging agents is examined in mESC lacking endogenous *Brca2*^19–24^ (Fig. 1a). Variants that partially or fully rescue the lethality of *Brca2*^*KO/KO*^ ES cells and are functionally indistinguishable from WT are considered to be neutral or “functionally normal”. In contrast, variants are considered to be hypomorphic if they rescue the lethality of *Brca2*^*KO/KO*^ ES cells but are not fully proficient in one or more functions (Fig. 1a). Our computational model combines the impact of the VUS on cell viability with their sensitivity to six different DNA damaging agents to calculate the PIF. We have used our VarCall model to estimate the PIF of 88 *BRCA2* variants distributed across the length of the protein, including 24 that are listed as VUS in the ClinVar database. We have also examined 26 variants that have conflicting classification data in ClinVar including at least one report classifying them as a VUS. We have further validated the model by examining an additional 25 variants that were previously functionally characterized by our mESC-based functional assay^23,24^.

## Results

### Functional analysis of variants: impact on viability of *Brca2*^*KO/KO*^ ES cells

We selected 88 variants for analysis in this study, each based on one or more of the following criteria: (i) the variant was listed as “uncertain” in ClinVar or class 3 in IARC classification; (ii) the variant was observed in Fanconi anemia (FA) patients; (iii) the variant may have an effect on splicing based on proximity to an exon–intron junction; (iv) truncating variants. Forty-seven variants are located in the DNA-binding domain (Fig. 1b). Twenty-four variants are listed as “uncertain” and twenty-six variants as having “conflicting interpretation” in ClinVar. Five variants are listed as class 3 in the IARC classification. Thirteen variants are located in close proximity to an exon-intron junction (within +3 or −3 from a splice junction site) (Supplementary Table 1). We included ten variants with previous classifications in the ClinVar database or based on published functional assay data for purposes of evaluating our assays and classification models. These were P168T, F1524V, G1529R, D2312V, K2411T (benign) and Q742X, E1308X, L2740X, C3069X, D3073G (pathogenic, Supplementary Table 1)^20,34,35^. In addition to these ten previously classified variants, we included V220Ifs as a negative (loss-of-function) control; WT constructs were used as a positive control.

We selected variants that map throughout the length of the protein unlike some of the other functional studies that have focused mainly on the variants in the DNA-binding domain (Fig. 1b)^33^. Because the variants were not completely randomly selected, the number of variants with normal or abnormal function does not reflect the overall frequency of pathogenic or benign variants in *BRCA2*. All 88 variants are listed using HGVS nomenclature in Supplementary Table 1. For simplicity and readability, the variants are identified in the text by using the single letter amino acid code to describe the change.

To evaluate the functional consequences of *BRCA2* VUS, we generated the selected variants in BAC containing full-length *BRCA2*. We confirmed the expression of the variants in mESC by reverse transcription polymerase chain reaction (RT-PCR). Two independent clones expressing each variant were subjected to CRE-mediated deletion of conditional *Brca2* allele. The reason for examining two independent clones expressing each *BRCA2* variant was to rule out any position effect associated with the site of integration of the BAC DNA into the ES cell genomic DNA. A functional *HPRT1* minigene is generated upon deletion of the conditional *Brca2* allele that allows selection of recombinant clones on hypoxanthine–aminopterin–thymidine (HAT) media (Fig. 1a). Loss of conditional *Brca2* allele in HAT-resistant (HAT^r^) colonies was further confirmed by Southern hybridization (Supplementary Fig. 1). Rescue rate was calculated based on the total number of viable *Brca2*^*KO/KO*^ mES cells that survived in HAT media over the total number cells plated. In each batch of the assay, the number of surviving colonies for each variant was compared to the number of surviving colonies for WT *BRCA2* (Supplementary Table 2).

Variants that failed to result in viable *Brca2*^*KO/KO*^ ES cells or showed reduced survival compared to WT controls (V220Ifs, R2336L, R2336P, Q2561P, F2562L, G2585R, G2596R, G2596E, D2611G, H2623R, K2630Q, L2647P, R2659G, R2659K, R2659T, S2670L, L2686P, S2691F, L2721H, D2723A, D2723V, Y2726C, R2784W, A2786P, W2788S, G2793R, Q2829X, Q2829L, Q2829R, C3069X, D3073G, and C3233Wfs) were further examined to confirm the expression of BRCA2 by Western blot analysis (Supplementary Fig. 2a). *Brca2*^*KO/KO*^ ES cells expressing truncating variants such as Q742X, E1308X, S1882X, S1926Rfs, R2488Rfs, L2740X, T2708Nfs, and Y3225Ifs variants were confirmed by RT-PCR due to failure to detect the truncated protein (Supplementary Fig. 2b). We failed to detect protein expression at levels comparable to WT in any ES cell clones with the variants R2336P, R2336L, S2691F, Q2829X, and C3069X (Supplementary Fig. 2a). It is possible that these variants result in an unstable protein. We have previously reported that c.7007 G > A (R2336H) results in exon skipping and the ES cells expressing the variant show lower protein levels compared to WT^24^. In cells expressing C3069X variant, we detected a very low level of expression of the protein in only one clone (Supplementary Fig. 2a). Inability to detect the C3069X protein in multiple RT-PCR positive clones suggests that the protein truncation may be affecting protein stability.

### Functional analysis of variants: effect on sensitivity to DNA-damaging agents

BRCA2 plays a major role in maintaining the genomic integrity by repairing DNA double strand break (DSB) by homologous recombination (HR) as well in protecting stalled replication forks^36–39^. BRCA2 is also required for repairing DNA interstrand cross-links^40^. Because of its involvement in multiple repair processes, cells lacking BRCA2 show hypersensitivity to a number of different DNA-damaging agents^20^. Therefore, variants that rescued the lethality of *Brca2*^*KO/KO*^ ES cells, partially or fully, were further tested for BRCA2 function by examining the sensitivity of rescued cells to six different agents: poly (ADP-ribose) polymerase (PARP) inhibitor (olaparib), DNA topoisomerase inhibitor (camptothecin), DNA interstrand cross-linking agent (cisplatin and mitomycin C, (MMC)), alkylating agent (methyl methane-sulfonate, MMS) and DSB inducer (ionizing radiation (IR)). Sensitivity of *Brca2*^*KO/KO*^ ES cells for each variant was compared with *Brca2*^*KO/KO*^ mESC expressing WT *BRCA2* (Supplementary Fig. 3 and Table 3).

### VarCall model for prediction of functional status

We developed a VarCall classification model for the data obtained from the cell viability (survival on HAT) assay as well as for the drug sensitivity (DS) assay to estimate the PIF of the VUS. The HAT survival and DS assay data were modeled both separately and together. The joint model assumed that the two forms of data are conditionally independent given variant pathogenicity status. This assumption implies that the two assays are correlated with binary function status, but given that a variant is functional or nonfunctional, the readouts from the two assays vary independently around their functional status-specific means. This does not, however, restrict the effects to be consistent with one another: population variation in an assay readout within the functional and nonfunctional groups may overlap. We evaluated each of the three resulting models (HAT, DS, and HAT + DS) on basis of the accuracy with which it predicted the status of the ten previously classified variants using a leave-one-variant-out analysis described below. The combined approach proved to be more powerful: jointly modeling the two data types allowed clear separation of VUS as neutral or pathogenic.

### VarCall model of cell viability

**Fig. 2:**
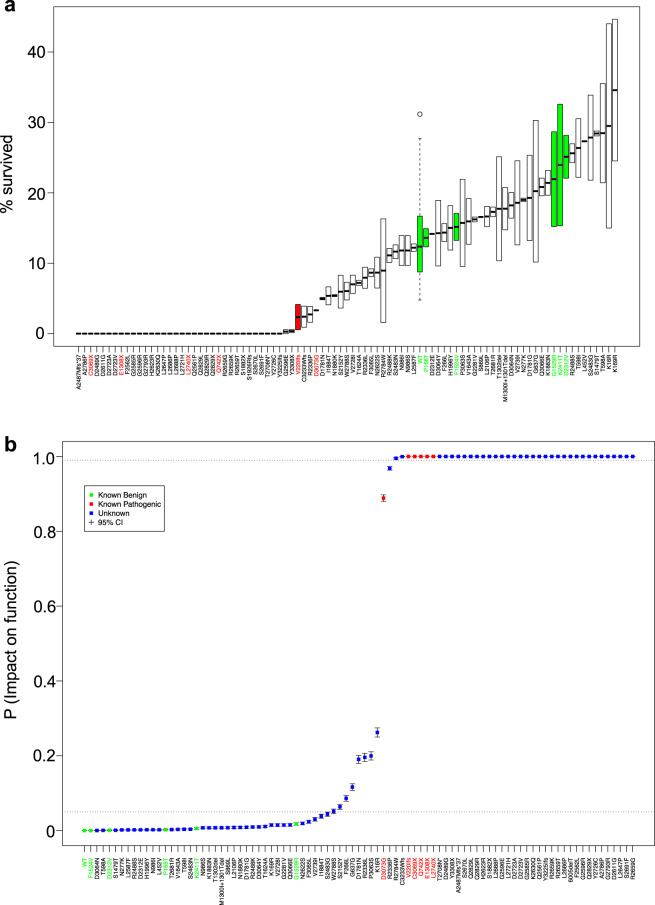
Probability of impact on function (PIF) estimates of cell survival. **a** Cell survival fractions are plotted for each variant ordered by average survival fraction. Known neutral variants are highlighted in green and known pathogenic variants are highlighted in red. The box plotted for each variant range from the lower replicate value up to the upper replicate value with the mean value highlighted at the midpoint; for the WT we have plotted a box and whisker plot of the distribution of WT values across batches (the box ranges from the first-to-third quartile with a horizontal line plotted at the median, whiskers extend from the box down to the smallest value and from the box up to the largest value within ±1.5 standard deviations of the median and points beyond this limit are depicted individually). **b** A plot of the estimated PIFs from the cell survival data VarCall model, depicted in increasing order. Posterior mean estimates are plotted as dots; whiskers extend from the lower to upper limits of 95% posterior credible intervals.

Eighty-eight variants were evaluated using the cell viability (HAT) assay, each batched in duplicate with a WT *BRCA2* construct (positive control). The number of surviving mES cells was recorded along with the total number of cells that were plated. Survival fractions were computed from these data and the average survival of two independent clones were determined (Supplementary Table 2 and Fig. 2a). Because only two clones expressing each variant were used to address any concerns related to the impact of the BAC integration site, standard deviation values were not obtained. However, it is evident that although the actual survival percentages differ between the two clones, no major differences (“full rescue” vs. “no rescue”) were observed for any variant (Supplementary Table 2). The HAT survival data were modeled as having a zero-inflated negative binomial distribution with a mean that depends on a random effect for batch and a random effect for variant. The latter follows the two-component normal mixture model common to all VarCall models. All VarCall models are classification tools because the true pathogenicity status of only a subset of the variants in any given analysis is known and these are labeled as functional or nonfunctional controls, as appropriate. The remaining variants have their impact on function status treated as an unknown binary variable, *IF*, indicating their status (impacted function/nonfunctional = 1; no impact/functional = 0). Statistical inference therefore focuses on estimating the posterior probability that *IF* = 1 for each such variant given the data. We refer to this as the variant’s PIF.

We estimated the accuracy of the VarCall models based on their predictions of the ten previously classified variants (P168T, F1524V, G1529R, D2312V, K2411T and Q742X, E1308X, L2740X, C3069X, D3073G). All analyses also included V220Ifs and the WT as negative (non-function) and positive (functional) controls, respectively. We labeled the two controls and ten previously classified variants as functional/not functional when classifying the VUS. In order to characterize the accuracy of the approach, we ran ten additional analyses, where in each we systematically left one of the ten (six missense and four nonsense variants) previously classified variants unlabeled and estimated its classification status with the remaining nine known +2 controls (WT and V220Ifs) labeled. We report and plot classification probabilities for the VUS based on the full analysis and for the known missense and nonsense variants based on the leave-one-variant-out analyses. We base our estimates of classification accuracy on the latter set of values.

We classified variants as “functional”, “nonfunctional”, or “indeterminate” based on the probability of impact on function (PIF) estimated by the VarCall model. In particular, we define variants as functional that have PIF ≤ 0.05, 0.05 < PIF ≤ 0.99 as indeterminate and those with PIF > 0.99 as nonfunctional. Figure 2b plots the estimated PIFs and associated 95% interval estimates in increasing order. Note that our cell survival (HAT assay) VarCall model co-clusters WT and 39 variants including S2483G and those to its left as likely functional and 39 variants to the right of R2336P as likely nonfunctional. The model classified all five benign variants correctly and four of five pathogenic variants correctly, leaving one (D3073G) unclassified with a PIF = 0.889. While our resolution is limited by the modest number of unlabeled variants of known status, we estimate sensitivity of the HAT-based classification procedure to be 0.69 (95% high density interval (HDI) from 0.37 to 0.98) and its specificity to be 0.85 (0.58, 1.00). The scaled Brier score was estimated to be 0.0051.

### VarCall model of sensitivity to DNA damaging agents

Fifty-six variants that supported viability of *Brca2*^*KO/KO*^ ES cells, partially or similar to WT *BRCA2*, were evaluated using the DS assay. Each variant was evaluated using two independent clones, batched with the WT expressing ESC control and was subjected to various concentrations/doses of each of the six DNA damaging agents. In all, the DS data comprises of 5488 measurements of the survival fraction and each measurement represents the average of three values.

**Fig. 3:**
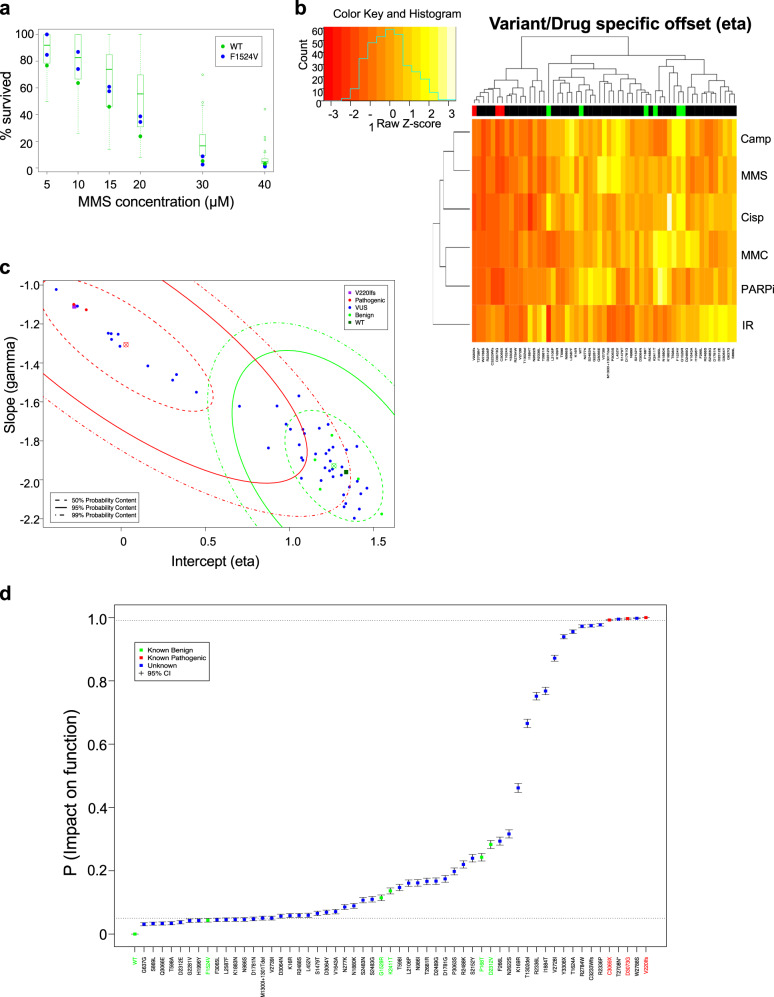
The drug sensitivity data and probability of impact on function (PIF) estimates. **a** A plot of the observed MMS assay survival fractions for variant F1524V (blue points); paired wildtype values are plotted as green points and the distributions of WT values across batches are depicted as boxplots (open circles represent outlier values). Note the characteristic nonlinear decline in survival fraction from 1.0 (100%) to zero as a function of concentration. This relationship is modeled well using the chosen family of dose response curves. **b** Bi-clustered heatmaps of the drug sensitivity model intercept parameters from the drug sensitivity model. Cells in the plot represent variant- (column) and drug-specific (row) effects. Higher survival percentage values are plotted in shades of yellow; lower values in shades of red and orange. The lower the value of eta, the faster the survival fractions decline as a function of dose, i.e., the greater the sensitivity of the variant. Variants tend to co-cluster as likely pathogenic or likely neutral based on these estimates (the green and red bars at the top of the plot indicate the known benign and known pathogenic variants, respectively). **c** Slope vs. intercept scatter plot of the estimated variant-level drug sensitivity effects for each variant. Contours of the bivariate VarCall mixture model components are plotted in green (neutral component) and red (pathogenic). Variants plotted at similar contour levels for both components will have equivocal PIF estimates; variants plotted at dissimilar levels (e.g., those in the upper left corner) will be more clearly classified (see panel **d**). **d** Estimated PIFs plot from the drug sensitivity data VarCall model, depicted in increasing order. Dots represent posterior mean estimates with the whiskers extend from the lower to upper limits of 95% posterior credible intervals.

The DS assay data are assumed to follow a multilevel dose response model. We evaluated the family of dose response models described by Slob (2002) over the range of variants and drug assays and found that curves of the form *y* = exp(−(*x*/*b*)^*d*^), where *x* is dose, *y* is survival fraction and *b* and *d* are parameters, provide a robust and accurate fit (Fig. 3a)^41^. After complementary log–log transformation of this parameterization, the response is linear in the log of dose. We model the dose response data on this scale, separately normalizing the log dose variable for each drug.

The model includes random effects that adjust the response variable for location and scale shifts due to batch and due to the drugs; the resulting variable is modeled as a simple linear regression on the log of dose with drug- and variant-specific intercept and slope coefficients. To ensure parameter identifiability, location and scale shifts for one drug and one batch are set to 0 (for location) and 1 (for scale). The bi-clustering of the DS model’s drug- and variant-specific intercept terms provides a side-by-side comparison of the drug sensitivities of individual variants in which each variant is depicted in a single column and in which its sensitivities to the individual drugs/treatments are pictured in the various rows (Fig. 3b). It reveals distinct drug response signatures among the known benign (highlighted in green) and among the known pathogenic variants (red) (Fig. 3b). These are segregated into the two main clusters defined by the top branching in the dendrogram; the effects profiles of the two groups are distinct with lower values corresponding to greater sensitivities (depicted in red and dark orange) typical of the left, loss-of-function cluster and lesser sensitivities (light oranges and yellows) typical of the functionally unimpaired cluster on the right.

The individual drug effects are variable (SD(intercept) = 0.86; SD(slope) = 0.55) relative to the differences (1.24 and 0.62, respectively) between their average effects in the functional and nonfunctional components of the mixture model. However, they provide complementary information: pairwise correlations between the drug treatment effects across variants range from near zero to 0.76 with a median of 0.49. This is evident in Fig. 3b where, for example, we see that the IR effect is often high (depicted in yellow) in variants where the PARPi effect is modest or low (oranges to reds) and vice versa. It is, therefore, useful to borrow strength from multiple agents. Indeed, combining the six results in a much less variable pair of composite drug effects: the intercept effect has SDs of 0.38 (functional component in the mixture model) and 0.34 (nonfunctional), while the slope effect has SDs of 0.17 and 0.11, respectively. Despite this sharpening of the drug signal, the two components of the mixture model retain a large amount of overlap (Fig. 3c), resulting in numerous equivocal calls when used alone for classification (Fig. 3d).

The variant- and drug-specific intercept and slope coefficients are random effects centered at variant-specific values. We perform variant classification based on the quantities measuring the overall drug sensitivities of the variants. In particular, we assume that variant-level slope and intercept terms follow a bivariate normal mixture model, with one component reflecting variation in the drug effects among neutral variants (green contours) and the other reflecting variation among pathogenic variants (red contours) (Fig. 3c). As with all VarCall models, we classify each variant according to the posterior probability of the variant belonging to the pathogenic component of the model.

Three of the known pathogenic variants and 29 of the VUS failed to rescue function and were not evaluated using the DS assay. Of the remaining 56 variants, 38 had PIFs in the indeterminant range, including four known neutral variants; 2 VUS and 3 pathogenic controls were called nonfunctional and 13 (including one neutral control) were classified as functional (Fig. 3d). PIFs from the DS model reflect greater classification uncertainty than those from the cell survival model (Fig. 2b). PIFs ranged from 0.044 up to 0.28 among the 5 known benign variants; the three known pathogenic variants had PIFs of 1 (V220Ifs), 0.9924 (C3069X), and 0.9996 (D3073G). The scaled Brier score, estimated using the seven labeled variants, was 0.1027.

### Combined model of cell survival and DS assay

We created a combined model for the cell survival and DS data that assumes their conditional independence given pathogenicity status. In particular, variants with both HAT and DS data provide information for the model’s HAT and DS parameters and both data types simultaneously and independently contribute to the estimation of the pathogenicity status indicator of those variants; variants with only HAT data contribute information to the model’s HAT parameters only and these data contribute solely to estimation of their classification indicators. Adding the DS data to the HAT model provides increased resolution for estimating the parameters of the HAT data model by providing complementary information on the pathogenicity status of the variants with DS data.

**Fig. 4:**
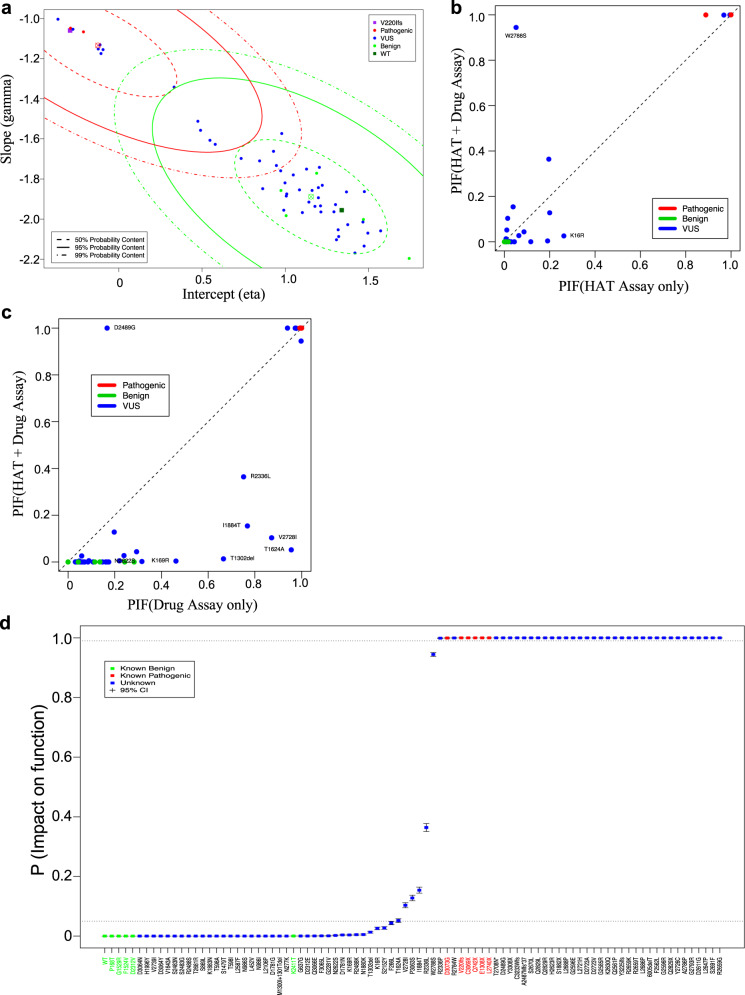
Classification parameters and estimates from the model combining the cell survival and drug sensitivity data. **a** Slope vs. intercept scatter plot for the combined model. **b** Plot of the PIF from the HAT model (*x*-axis) against the PIF from the combined model (*y*-axis). **c** Plot of the PIF from the drug assay model (*x*-axis) against the PIF from the combined model (*y*-axis). **d** Plot showing the estimated PIFs plot from the combined VarCall model of HAT survival and drug sensitivity data, depicted in increasing order. Posterior mean estimates and represented as dots and the whiskers extend from the lower to upper limits of 95% posterior credible intervals.

The pathogenic and neutral components of the variant-specific drug effects from the combined model (Fig. 4a) are better separated than those of the model for the DS assay data alone (Fig. 3c) and the variants of known status all co-cluster under the appropriate distributional component. Indeed, addition of the HAT survival data to the DS data leads to substantial refinement of the PIFs and results in more certain classifications and greater accuracy (Fig. 4b, Table 1): PIFs are closer to zero or one and the PIFs associated with the known neutral variants (green) are systematically closer to zero (all fall below the diagonal) and those associated with the known pathogenic variants (red) are systematically closer to one (all fall above the diagonal). Addition of the DS data to the cell survival data results in more modest changes to the estimated PIFs, however, it provides important resolution to variants that show evidence of rescued function. The effect on the PIFs inferred for these variants can be seen in Fig. 4c. With addition of the drug data, the PIF associated with the known pathogenic variant D3073G changed from 0.9996 to 0.9966 (Table 1). Several of the VUS show increased evidence of pathogenicity, most notably D2489G and R2336L, while others show decreased evidence (Fig. 4c).

**Table 1:** Probability of impact on function (PIF) of variants based on cell survival (HAT) and drug sensitivity (DS) assay results individually and combined (HAT + DS).

VARIANT	PIF[HAT]	PIF[DS]	PIF[HAT + DS]^a^	VARIANT	PIF[HAT]	PIF[DS]	PIF[HAT + DS]^a^
K16R	0.2624	0.0584	0.0258 (F)	Q2561P	1	NA	1 (NF)
P168T	0.0026	0.2426	0 (F)	F2562L	1	NA	1 (NF)
K169R	0.0148	0.4618	0.0038 (F)	G2585R	1	NA	1 (NF)
V220Ifs	1	1	1 (NF)	L2587F	0.0020	0.0454	0 (F)
F266L	0.0864	0.2936	0.0438 (F)	G2596R	1	NA	1 (NF)
N277K	0.0018	0.0854	0 (F)	G2596E	1	NA	1 (NF)
L452V	0.0024	0.0594	0 (F)	D2611G	1	NA	1 (NF)
T598A	0.0008	0.0342	0 (F)	N2622S	0.0192	0.3164	0.0020 (F)
T598I	0.0036	0.1472	0 (F)	H2623R	1	NA	1 (NF)
G637 =	0.1164	0.0312	0.0002 (F)	K2630Q	1	NA	1 (NF)
Q742X	1	NA	1 (NF)	L2647P	1	NA	1 (NF)
S869L	0.0078	0.0330	0 (F)	R2659G	1	NA	1 (NF)
N986S	0.0074	0.0460	0 (F)	R2659K	1	NA	1 (NF)
N986I	0.0024	0.1616	0 (F)	R2659T	1	NA	1 (NF)
M1300I + 1301Tdel	0.0076	0.0504	0 (F)	S2670L	1	NA	1 (NF)
T1302del	0.0076	0.6654	0.0132 (F)	T2681R	0.0028	0.1666	0 (F)
E1308X	1	NA	1 (NF)	L2686P	1	NA	1 (NF)
S1479T	0.0010	0.0654	0 (F)	L2688P	1	NA	1 (NF)
F1524V	0	0.0436	0 (F)	S2691F	1	NA	1 (NF)
G1529R	0.0176	0.1146	0 (F)	T2708Nfs	1	0.9950	1 (NF)
T1624A	0.0110	0.9554	0.0518 (I)	L2721H	1	NA	1 (NF)
V1643A	0.0032	0.0710	0 (F)	D2723A	1	NA	1 (NF)
D1781N	0.1906	0.0478	0.0038 (F)	D2723V	1	NA	1 (NF)
D1781G	0.0090	0.1744	0 (F)	Y2726C	1	NA	1 (NF)
N1880K	0.0086	0.0892	0.0052 (F)	V2728I	0.0148	0.8716	0.1034 (I)
S1882X	1	NA	1 (NF)	V2739I	0.0302	0.0506	0 (F)
K1883N	0.0074	0.0458	0 (F)	L2740X	1	NA	1 (NF)
I1884T	0.0386	0.7680	0.1540 (I)	R2784W	0.9952	0.9724	0.9998 (NF)
S1926Rfs	1	NA	1 (NF)	A2786P	1	NA	1 (NF)
H1996Y	0.0024	0.0428	0 (F)	W2788S	0.0516	0.9976	0.9444 (I)
L2106P	0.0084	0.1610	0 (F)	G2793R	1	NA	1 (NF)
S2152Y	0.0636	0.2396	0.0272 (F)	Q2829X	1	NA	1 (NF)
G2281V	0.0150	0.0422	0.0008 (F)	Q2829L	1	NA	1 (NF)
D2312V	0.0010	0.2830	0 (F)	Q2829R	1	NA	1 (NF)
D2312E	0.0022	0.0374	0.0002 (F)	P3063S	0.1998	0.1978	0.1278 (I)
R2336L	0.1960	0.7516	0.3642 (I)	D3064N	0.0004	0.0566	0 (F)
R2336P	0.9684	0.9770	0.9990 (NF)	D3064Y	0.0100	0.0688	0 (F)
K2411T	0.0058	0.1364	0.0002 (F)	F3065L	0.0236	0.0448	0.0006 (F)
S2483G	0.0440	0.1100	0 (F)	Q3066E	0.0152	0.0336	0.0004 (F)
S2483N	0.0038	0.1072	0 (F)	C3069X	1	0.9924	1 (NF)
R2488Rfs	1	NA	1 (NF)	D3073 G	0.8888	0.9996	0.9966 (NF)
R2488K	0.0096	0.2200	0.0048 (F)	Y3225Ifs	1	NA	1 (NF)
R2488S	0.0022	0.0590	0 (F)	C3233Wfs	0.9996	0.9744	1 (NF)
D2489G	1	0.1672	1 (NF)	Y3308X	1	0.9392	1 (NF)

^a^Functional classification indicated in parenthesis. *F* functional, *NF* nonfunctional, *I* indeterminate, *NA* not available.

The estimated PIFs and associated 95% interval estimates derived from the combined model are plotted in Fig. 4d. PIFs from the combined model reflect improved classification accuracy over those from the cell survival (Fig. 2b) and DS (Fig. 3d) models. Forty-one variants including F266L and those that are plotted to its left are classified as likely functional (PIF ≤ 0.05) (Fig. 4d and Table 1). The variant R2336P and the 40 variants that were plotted to its right are classified as likely nonfunctional (PIF > 0.99), whereas the remaining six variants (T1624A, V2728I, P3063S, I1884T, R2336L, and W2788S) are indeterminate (0.05 < PIF ≤ 0.99) (Fig. 4d and Table 1). The combined model demonstrates improved accuracy, correctly classifying all ten variants of known pathogenicity status: the largest PIF among the known benign variants was 0.0002, while the smallest PIF among the known pathogenic variants was 0.9966. We estimate both the sensitivity and specificity of classifications based on the combined model to be 0.846 (95% HDI from 0.58 to 1.00). The scaled Brier score was estimated to be 8 × 10^−8^.

### Performance of VarCall model for mES cell-based functional assay

Finally, we tested the performance of our VarCall model by examining 25 *BRCA2* variants that were previously analyzed by our mES cell-based approach^22–24^. Among these 25 variants, 5 are listed as benign and nine as pathogenic or likely pathogenic in ClinVar. The variants map to different regions of the protein and include 19 missense variants, 1 truncating variant, 1 splice site variant, and 4 in-frame deletion variants (Table 2). Those variants have been comprehensively characterized not just by their impact on cell viability and sensitivity to DNA damaging agents but also by IR-induced RAD51 foci formation, HR efficiency, cell proliferation, as well as impact on genomic instability by cytogenetic analysis. Our functional studies had revealed that 10 were likely to be neutral, 13 were likely to be pathogenic, and 2 variants exhibited an intermediate phenotype^23,24^. We had reported G25R to have no impact on cell viability but cells expressing this variant were mildly sensitive to DNA damaging agents and exhibited mild genomic instability compared to the other pathogenic variants^23^. In contrast, the variant lacking the 105 amino acids encoded by exons 4–7 (del exon 4–7) resulted in a mild reduction in cell viability but there was no effect of DNA damaging agents, cell proliferation, IR-induced RAD51 foci formation^24^.

**Table 2:** BRCA2 variants used to test the performance of the VarCall model.

Variant^a^	Amino acid change^b^	ClinVar classification^c^	BRCA exchange^d^	mES-based classification^e^
c.73G > A	p.Gly25Arg (G25R)	Uncertain (2)		Indeterminate
c.91 T > C	p.TrpW31Arg (W31R)	Uncertain (1)		Non-functional
c.93 G > T	p.Trp31Cys (W31C)	Likely Pathogenic (1)/Uncertain (1)		Non-functional
c.73_118del (delex3-5’)	p.Gly25_Ala40del (G25_A40del)			Non-functional
c.119_312del (delex3-3’)	p.Pro41_Asp104del (P41_D104del)			Functional
c.317_631del (delex4-7)	p.Gly106_Ile210del (G106_I210del)			Indeterminate
c.581 G > A	p.Trp194Stop (W194X)	Pathogenic (10)	Pathogenic	Non-functional
c.631 + 2 T > G	IVS7 + 2 T > G	Pathogenic (2)/Likely Pathogenic (1)	Pathogenic	Non-functional
c.1964C > G	p.Pro655Arg (P655R)	Benign (22)	Benign	Functional
c.6323 G > A	p.Arg2108His (R2108H)	Benign (27)	Benign	Functional
c.6853 A > G	p.Ile2285Val (I2285V)	Benign (18)	Benign	Functional
c.6842_6937del (delex12)	p.Gly2281_Asp2312de (G2281_D2312del)l			Functional
c.7007 G > A	p.Arg2336His(R2336H)	Pathogenic (23)	Pathogenic	Functional
c.7216_7217TT > GA	p.Phe2406Asp (F2406D)			Functional
c.7218 T > G	p.Phe2406Leu (F2406L)	Uncertain (1)		Functional
c.7468 A > G	p.Ile2490Thr (I2490T)	Uncertain (1)		Functional
c.7529 T > C	p.Leu2510Pro (L2510P)	Likely Pathogenic (4)		Non-functional
c.7878 G > C	p.Trp2626Cys(W2626C)	Pathogenic (16)	Pathogenic	Non-functional
c.7958 T > C	p.Leu2653Pro (L2653P)	Pathogenic (4)/Likely Pathogenic (3)		Non-functional
c.8084 C > T	p.Ser2695Leu (S2695L)	Likely benign (1)/Uncertain (2)		Functional
c.8187 G > T	p.Lys2729Asn (K2729N)	Benign (16)	Benign	Functional
c.8830 A > T	p.Iso2944Phe (I2944F)	Benign (26)	Benign	Functional
c.9004 G > A	p.Glu3002Lys (E3002K)	Pathogenic (11)/Likely Pathogenic (4)		Nonfunctional
c.9285 C > G	p.Asp3095Glu (D3095E)	Pathogenic (6)/Likely Pathogenic (2)		Nonfunctional
c.9371 A > T	p.Asn3124Iso (N3124I)	Pathogenic (20)	Pathogenic	Nonfunctional

^a^Variant names are according to HGVS nomenclature.

^b^Single letter amino acid codes are in the parentheses.

^c^Number of entries in ClinVar as of 23rd September 2020 are in the parentheses.

^d^Website: https://brcaexchange.org/variants.

^e^Variants previously characterized by ES cell-based functional assay^22–24^.

**Fig. 5:**
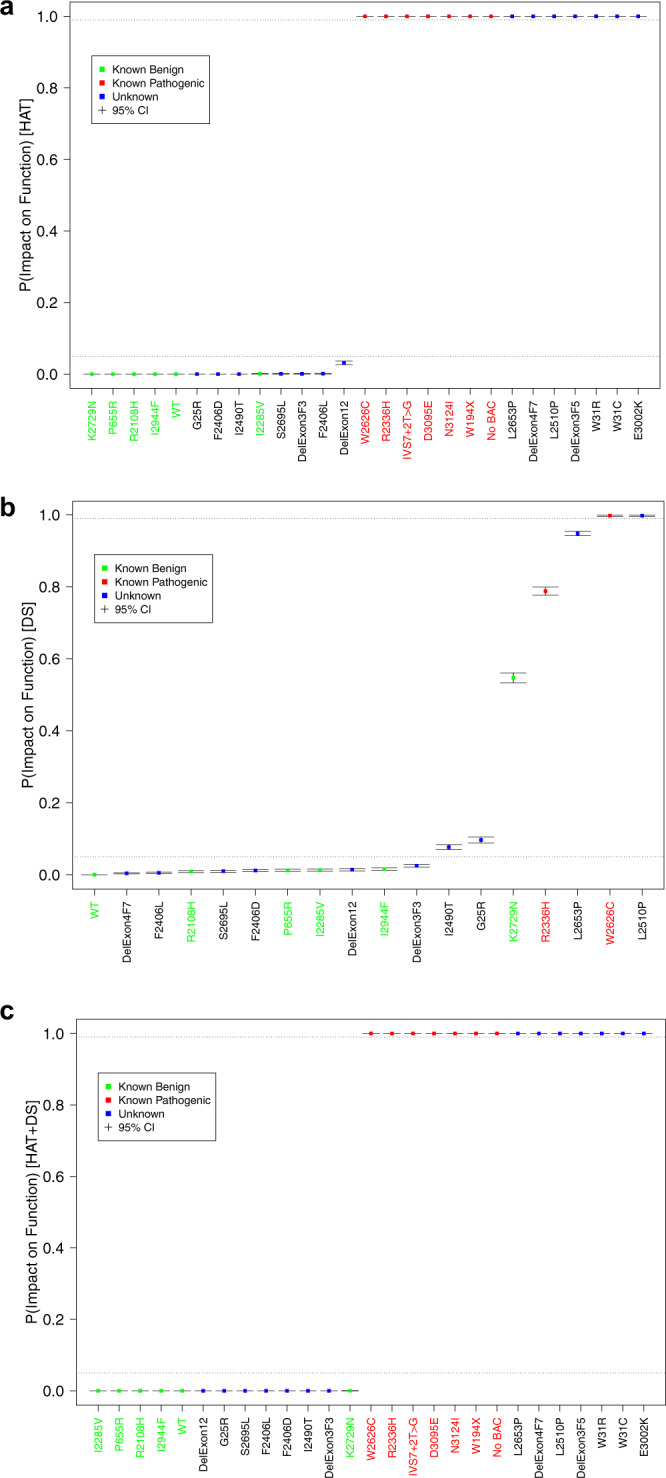
Classification of variants using combined data of drug sensitivity assay and cell survival. Boxplots of the PIFs estimated for the validation set variants, plotted in increasing order. **a** Based on HAT model, **b** based on the DS model, and **c** based on the HAT + DS model. Note that variant del exon 4–7 is estimated to be neutral based on the HAT data and pathogenic based on the drug sensitivity assay. It remains unclassified when the data are combined.

We reanalyzed these variants using our VarCall models as an additional independent validation of the model. PIFs estimated by these models (HAT, DS, and HAT + DS) are plotted (Fig. 5). All variants of known status were called correctly by both the HAT and HAT + DS models (Fig. 5a, c). Specificity of both models was estimated to be 0.846 (95% HDI from 0.58 to 1.00), while sensitivity was estimated to be 0.867 (95% HDI from 0.63 to 1.00). Here, addition of the DS to the HAT data did not change the classification. However, comparing variants classified by both the DS and HAT models only one variant, del exon 4–7, was discordant: it was classified as nonfunctional (PIF = 1.00) by the HAT model and as functional by the DS model (PIF = 0.004).

### Alternative splicing in pathogenic variants

We next characterized some of the BRCA2 variants that are predicted to be functionally null but our functional studies revealed that they are partially functional. The variants V220Ifs (c.658_659delGT [886delGT]) and C3233Wfs (c.9699_9702delTATG [9927del4]) are predicted to generate a premature stop codon in exon 8 and exon 27, respectively but we were able to detect protein, albeit at a reduced level as compared to WT (Supplementary Fig. 2a). Both variants were also able to rescue the lethality of *Brca2*^*KO/KO*^ ES cells, albeit at low levels (Fig. 2). RT-PCR analysis using primers from exon 2 and exon 10 detected transcripts that lacked exon 7, 3′ 39 bp of exon 6 and exon 7, exon 3 and exons 3–7, in mES cells expressing V220Ifs (Supplementary Fig. 4). Transcripts lacking exon 7 in V220Ifs code for BRCA2 protein (exon 7 skipping deletes 115 bp and 2 bp deletion from exon 8 in V220Ifs brings the protein in-frame) with an internal deletion of 39 amino acids that have been shown to be dispensable for BRCA2 function (Supplementary Fig. 5)^24^. The transcript that lacks 3′ 39 bp of exon 6 and exon 7 will also code for a BRCA2 protein with an internal deletion of 52 amino acids in V220Ifs expressing variant (3′ 39 bp of exon 6 and exon 7 skipping deletes 154 and 2 bp deletion from exon 8 in V220Ifs brings the protein in-frame). This explains the detection of protein and the rescue of *Brca2*^*KO/KO*^ ES cells viability in the cells expressing V220Ifs variant. We failed to detect any alternatively spliced transcripts in the C3233Wfs variant (Supplementary Fig. 4). The detected protein in the cells expressing the C3233Wfs variant is thus the truncated protein (Supplementary Fig. 2a). Based on clinical data, the C3233Wfs variant is considered benign and classified as a variant with “special interpretation”. This variant was found in trans with a known pathogenic variant in several individuals without developing FA and also does not segregate consistently with developing cancer in families harboring this variant^42^. The variant C3233Wfs results in a truncated BRCA2 protein that lacks nuclear localization signals and part of the RAD51 binding domain^43,44^ and it is also upstream of a known pathogenic variant that results in truncation at codon 3308 (Y3308X) (https://www.ncbi.nlm.nih.gov/clinvar/)^20^. In our assay *Brca2*^*KO/KO*^ ES cells expressing C3233Wfs variant is also defective in both IR-induced RAD51 foci formation and also in protecting replication fork stability, the two known functions of BRCA2 (Supplementary Fig. 6). At present, it is unclear how this potentially pathogenic variant in our functional assay behaves as a benign variant based on familial segregation data.

We failed to detect any protein expression or changes in splicing pattern of T2708Nfs (c.8122dupA [8122insA]), though there were very few *Brca2*^*KO/KO*^ ES cells in this variant after HAT selection. The rescued *Brca2*^*KO/KO*^ ES cells showed the presence of mutant variant when we sequenced the region of the BAC from mES cells (data not shown). It is possible that few cells expressed very low levels of the protein due to alternative splicing that we failed to detect in RT-PCR or Western blot analysis.

### Effect of *BRCA2* variants on splicing

The variants G2281V, D2312V, D2312E, R2336L, R2336P, R2659G, R2659K, R2659T, Q2829X, Q2829G, and Q2829R are present at or nearby the consensus splice site of exons (Supplementary Table 1). All the variants except R2336L and R2336P either complemented the lethality of *Brca2*^*KO/KO*^ ES cells or expressed the protein comparable to WT (Fig. 2 and Supplementary Fig. 2a). R2336L and R2336P are located at the splice donor site of exon 13 and they caused skipping of exon 13 and also of exons 12 and 13, which resulted in the generation of a premature stop codon in exon 14 (Supplementary Figs. 4 and 5). The variant G2281V is located at the splice donor site and the variants D2312V, D2312E are located near the splice acceptor site of exon 12. The G2281V and D2312V variants resulted in either complete or partial skipping of exon 12 whereas the D2312E variant did not affect splicing of exon 12 (Supplementary Figs. 4 and 5). None of these three variants had any effect on the rescue efficiency of *Brca2*^*KO/KO*^ ES cells (Fig. 2). This is in agreement with our previous findings showing that exon 12 is dispensable for known *BRCA2* functions^22^. The variants R2659G, R2659K, and R2659T are located at or near the splice acceptor site of exon 17 (Supplementary Table 1). The variants R2659K, R2659T results in exon 17 skipping that generates a protein with an internal deletion of 57 amino acids, whereas R2659G variant results in only a minor skipping of exon 17 (Supplementary Figs. 4 and 5). Skipping of exon 19 was observed in the cells expressing Q2829X, Q2829L and Q2829R variants, that results in internal deletion of 52 amino acids (Supplementary Figs. 4 and 5). The S2691F variant fails to complement the loss of *Brca2* and the expression of protein was significantly reduced compared to WT (Fig. 2 and Supplementary Fig. 2a). In a minigene-based splicing assay, S2691F was reported to cause minor skipping (5.1%) of exon 18^45^. We further checked if this variant causes any aberrant splicing but we failed to detect any effect on splicing in the mES cells (Supplementary Fig. 4).

## Discussion

Accurate determination of the pathogenicity of variants of uncertain clinical significance (VUS) is a major hurdle in the clinical interpretation of genetic testing results. Despite the progress made by individual laboratories and international collaborations and consortiums such as the ENIGMA and BRCA Exchange to classify variants using familial and clinical data, the vast majority of *BRCA* variants are VUS. This remains the biggest challenge for physicians and genetic counselors who have to offer guidance to individuals who are positive for a *BRCA* VUS. A number of functional assays including mES cell-based assays have been developed and are being used to classify *BRCA2* variants to overcome this problem^20,34^. However, most functional assays determine the functional impact of variants without determining the probability of impact on function (PIF). An important consideration is how to best incorporate functional assay data into multifactorial predictive models. A computational tool called VarCall was previously used to evaluate 139 *BRCA2* variants located at the C-terminal *BRCA2* DNA-binding domain (DBD)^33^ using data from a *BRCA2* HR-based functional assay. In this study, we have analyzed *BRCA2* VUS using a mES cell-based functional assay and used our VarCall models to determine their probability of impact on function (PIF).

We developed novel VarCall models to classify VUS based on data obtained from a cell survival assay, sensitivity of the mES cells expressing *BRCA2* variants to six DNA damaging drugs and to the combined effect of both assays. The VarCall models previously described were each structured for a single functional assay, one for a BRCA1 transcriptional activation assay and the other for a BRCA2 HR assay^23,24^. These models assumed a log-normal sampling model and included batch and variant random effects. The VarCall model developed for the present study was tailored to the multivariate assay data it generated. In particular, it utilizes a negative binomial sampling model for the HAT colony counts (cell survival) and a family of dose response models for the DS data, the latter representing a total of 40 survival measurements collected from 6 different agents at multiple concentrations or doses for each variant. Data from the two assays are independently batch corrected and the model includes a variant-specific random effect measuring function based on the HAT data and two variant-specific random effects measuring function based on the DS data. The two DS effects are modeled as correlated and independently combined with the HAT effect, conditional on binary pathogenicity status. This defines a multivariate, two-component mixture model that is used to estimate PIFs and variant classifications.

Based on the hypothesis that sensitivities to various DNA damaging agents reflect the same basic defect in BRCA2-mediated DNA repair, we treated a weighted average of the drug-specific effects as a common drug response effect, instead of treating them as six independent functional assays. While the combined effect of cell survival and drug assays are used to determine the PIF of any variant, variants that fail to rescue viability of *Brca2*^*KO/KO*^ ES cells rely exclusively on the results of the cell survival data. In the combined HAT + DS model, only the HAT data directly inform PIFs for variants that fail to rescue, while the PIFs of those variants that do survive are directly informed by the DS data as well. This leads to more accurate estimates of the parameters associated with the two-component mixture model on basis of which these PIFs are estimated.

**Fig. 6:**
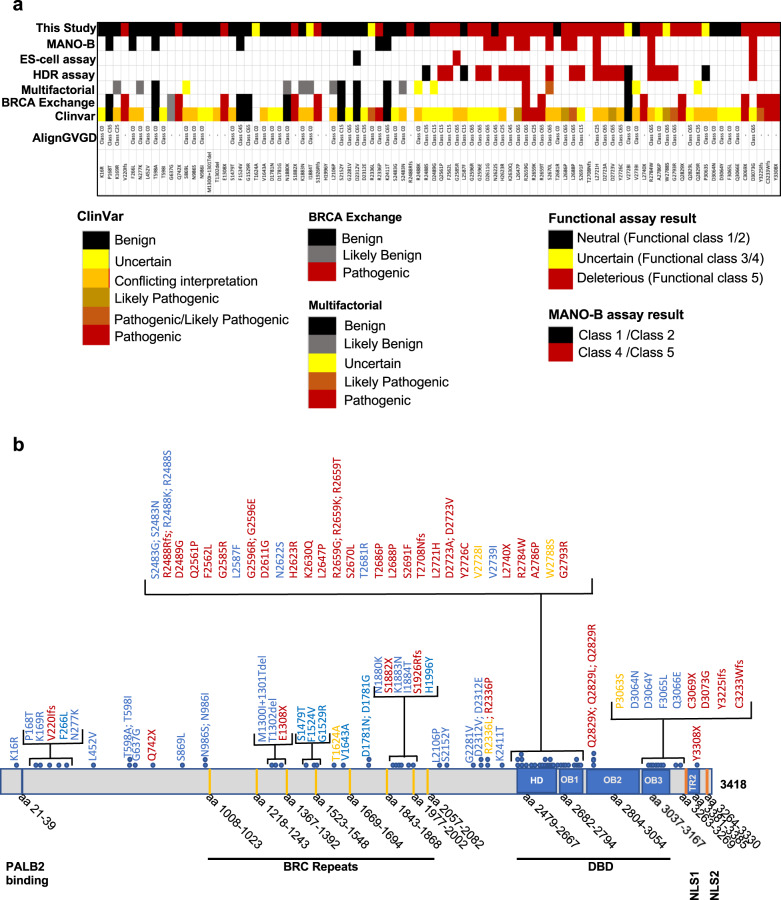
Classification of variants analyzed using the VarCall method. **a** Variants analyzed using VarCall method are compared to ClinVar, Align GVGD, BRCA Exchange, classification by multifactorial likelihood ratio and published BRCA2 functional assays. Colored boxes represent different classifications listed below. The MANO-B (mixed-allnominated-in-one (MANO)-BRCA) method of classification includes the classification by Ikegami et al. (2020), the ES assay includes the classification of variants using mouse ES cell-based assay reported by Mesman et al. (2019) and the HDR assay includes the classification of variants using HR assay reported by Guidugli et al. (2018)^18,19,33^. The Multifactorial classifications are based on the study by Parson et al.^51^. **b** Schematic representation of classified variants in different domains of BRCA2. Neutral, hypomorphic and pathogenic variants are marked in blue, yellow, and magenta colors, respectively. Different domains are marked below with the range of amino acids (aa) containing in each domain.

Among the 88 VUS analyzed in this assay, 41 showed >99% probability in favor of abnormal function and 41 showed >95% probability in favor of normal function. The variants, T1624A, V2728I, P3063S, I1884T, R2336L, and W2788S showed intermediate phenotype in our model (Fig. 6a and Table 1). Among these, five variants T1624A, V2728I, P3063S, I1884T, and R2336L, are close to having normal function (likely neutral) with PIFs in between 0.05 and 0.36, whereas W2788S is close to abnormal function with PIF 0.94 (>0.85 PIF < 0.99). Future studies will be focused on integrating the PIF values obtained from our functional assays into the posterior probability models that will help in assessment of the clinical relevance of a VUS^46^.

Classification of VUS based on posterior probabilities from a Bayesian “system”, which by construction, requires specification of prior probabilities has been suggested by Plon et al.^29^ previously. Some recent *BRCA* VUS classification models have used the Align GVGD sequence conservation model to specify these prior probabilities^33,47,48^. In contrast, in the VarCall models we describe here, we specified a non-informative prior distribution on each variant’s function status. This allows us to compare the calibration of our PIF estimates to the Align GVGD based prior probabilities. We found twenty out of 29 variants (~68%) that are Align-GVGD categories associated with moderate (Class C35–C55) to high (Class C65) prior probabilities are classified as nonfunctional or close to non-functional (W2788S with PIF 0.94) using our VarCall model (Fig. 6a). Interestingly, among the remaining nine variants that are Align-GVGD categories C35-C65 associated with moderate to high prior probabilities of pathogenicity but did not have impact on function based on our VarCall model, seven are located outside the DBD (Fig. 6b). Three (G2281V, D2312V, and D2312E) out of these seven variants are located at or close to the exon–intron junction (Supplementary Table 1). Two (G2281V and D2312V) result in full or partial exon 12 skipping but exon 12 is dispensable for BRCA2 function (Supplementary Figs. 4 and 5)^22^. Considering the high sensitivity and specificity of our assay, these results suggest that the Align-GVGD may over-predict the probability of pathogenicity for *BRCA2* VUS. The VarCall model for the BRCA2 HDR assay also had significant discrepancies with the Align-GVGD prediction^33^. Variants with lower prior probabilities of pathogenicity (Class C0–C25) by Align-GVGD showed better agreement (~80%; 34 neutral or likely neutral out of 42 that are in class C0-C25) with our functional classification. Eight variants with lower prior probabilities of pathogenicity (Class C0–C25) by Align-GVGD that are classified as non-functional using our VarCall model, are located in the DBD. Recent calculations of prior probabilities of pathogenicity also take into account the location of the amino acid in the protein as well as the impact of the nucleotide on splicing (http://hci-priors.hci.utah.edu/PRIORS/)^49^. These prior probabilities of pathogenicity (PPP) show better agreement with the PIF values obtained by our VarCall model (Table 1 and Supplementary Table 1).

Interestingly, in our model, all pathogenic missense variants except two that affect splicing (R2336L and R2336P) are clustered in the DBD (Fig. 6b). It is not surprising because the function of BRCA2 in HR and replication is facilitated by the DBD located at the carboxy (C) terminus of the protein^36–38,50^. Our VarCall classifications shows high concordance with that of the HDR assay (22/24), mES cells based HDR assay performed by other groups (5/5) or with a recently published high-throughput functional assay based on complementation of PARPi sensitivity (15/17) (Fig. 6a)^18,19,33^. The variants V2728I and W2788S are classified as neutral or deleterious by HR assay whereas in our assay they have PIFs 0.10 and 0.94, respectively (Table 1)^33^. We have classified them as indeterminate but clearly V2728I is close to functional and W2728S is close to non-functional. The variant R2336P is classified as neutral by Ikegami et al.^18^, whereas this variant that affects splicing is pathogenic in our model and in agreement with ClinVar. This further raises a concern about classifying variants using cDNA based functional assays that fail to consider the potential effect of the variants on splicing. Importantly, there are no discordances with the variants that scored pathogenic or neutral based on our VarCall model and the pathogenic/likely pathogenic and benign/likely benign classification in ClinVar except V2728I and R2336L, another variant that affects splicing. V2728I is classified as benign, and in our model it is indeterminate variant with PIF of 0.10 that is closer to neutrality (>85% probability) (Table 1). In ClinVar, R2336L is listed as “likely pathogenic” whereas based on our VarCall classification it appears to be indeterminate variant with PIF of 0.36.

We also compared the results of our VarCall model with the recent classification of *BRCA1/2* variants using multifactorial likelihood ratios and found 13 out of 19 variants to be classified similarly^51^ (Fig. 6a). Among the six variants that were not in concordance, V2728I is considered benign according to the multifactorial model, but classified as indeterminate by our assay with PIF of 0.10 (in favor of normal function with >85% probability) (Table1). The remaining five variants (S869L, R2488K, D2489G, V2739I, and Q2829R) were classified as uncertain by the multifactorial model. Three of these variants (S869L, R2488K, and V2739I) showed probability in favor of normal function and two (D2489G and Q2829R) showed probability in favor of abnormal function in our assay system (Fig. 6a). Additional clinical and/or functional data are needed to ascertain the impact of these variants.

In conclusion, we have performed an extensive functional analysis of *BRCA2* variants and demonstrated that our mES-cell-based functional assay is a robust tool for classification of *BRCA2* VUS. Furthermore, our VarCall classification model can be used to determine the probability of impact on function that can aid in the clinical annotation of VUS. However, the disease risk associated with any VUS must be cautiously interpreted based on any in vitro functional assay either alone or in combination with in silico prediction tools. As is true of other BRCA2 functional assays, our results are not intended to be directly used for clinical risk assessment. Given that both sensitivity and specificity of our assay are based on relatively small number of established pathogenic and benign variants, additional studies are warranted to further validate the reliability of our mESC-based assay and the VarCall models.

## Methods

### Variant nomenclature and in silico analysis

HGVS nucleotide nomenclature reflects cDNA numbers with +1 corresponding to the A of the ATG initiation codon in the reference sequence of *BRCA2* (GenBank accession number NM_000059.3). Variants were examined for evolutionary conservation using Align-GVGD (http://agvgd.hci.utah.edu/agvgd_input.php). Grantham variation (GV) and Grantham deviation (GD) scores combine protein multiple sequence alignments and the physiochemical properties of the amino acids. The GV and GD scores are C0, C15, C25, C35, C45, C55, C65, with C0 being most likely neutral and C65 being most likely pathogenic.

### Generation of *BRCA2* variant expressing PL2F7 mES cells

A BAC clone (CTD-2342K5 with a 127 kb insert) containing full-length *BRCA2* in SW102 cells was used to generate variants by a recombineering method as described previously^23,52^. All BACs were sequenced to determine that no undesired mutations were generated after recombineering. Oligonucleotide sequences are available upon request. After electroporating BAC DNA (20 µg) carrying various mutant alleles of *BRCA2* into 1.0 × 10^7^ PL2F7 mES cells, cells were selected in the presence of G418 (Invitrogen) and characterized as described previously^20^. RT-PCR was carried out using the Titan One Tube RT-PCR system (Roche) following the manufacturer’s protocol to confirm *BRCA2* expression. Primers used are from exon 11 (5′-TGGTTTTGTCAAATTCAAGAATTGG-3′) and exon 14 (5′-CCAATCAAGCAGTAGCTGTAACTTTCAC-3′). Amplified 627 bp product was detected in agarose gel. After the BAC containing mESC were obtained, presence of the correct variant and lack of undesired mutations were confirmed by sequencing again.

### BRCA2 functional analysis

For mES cell viability assay, the conditional allele of *Brca2* in mES cells expressing each variant was deleted by electroporating 20 μg of *Pgk-Cre* plasmid as described previously^23^. For each variant two independent *BRCA2* expressing clones were used to address any variability due to the site of integration of the BAC clone in the ES cells. After electroporation, 1 × 10^6^ cells were plated in two 100 mm dishes and one was used for selection in the HAT media (Gibco) to select recombinant colonies. The second 100 mm dish was used as plating efficiency control. The mES cell colonies were visualized by staining with methylene blue (2% methylene blue (wt/vol) in 70% ethanol for 15 min followed by washing in 70% ethanol). Sensitivity assays to different drugs and IR were performed by XTT assay as described previously^20^. In brief, 10,000 ES-cells per well (15,000 cells per well for slow-growing mutants to compensate for lower seeding and growth efficiency) were seeded in 96-well plates. After 18–20 h, cells were treated with different damaging agents in triplicate. Concentrations/dose used were: Camptothecin: 0, 2.5, 5, 25, 50, 100, and 200 µM; Mitomycin C(MMC): 0, 5, 10, 20, 40, 60 ng/ml; Cisplatin: 0, 0.2, 0.4, 0.6, 1.0, 1.2, 1.5 µM; methyl methanesulfonate (MMS): 0, 50, 10, 15, 20, 30, 40 µg/ml; PARP inhibitor (olaparib): 0.01, 0.1, 1, 10 µM; IR: 0, 0.5, 1, 2, 4, and 6 Gy. Plates were exposed to a ^137^Cs source at 146.3 rad/min without media change for γ-irradiation. After irradiation, fresh media was added. Cell viability of treated cells was determined relative to untreated after 72 h. using XTT assay. All values were adjusted for a no-cells control.

To count the colonies, petri dishes were stained with 0.3% methylene blue and scanned at a resolution of 600 dpi, and processed with ImageJ software (NIH, Bethesda, MD). Area of image near dish walls was not analyzed. Because colonies have different size and morphology, and may overlap, the ImageJ script (see “Supplementary methods”) relied on user input to select parameters which result in optimal identification of individual colonies, as judged by visualization with overlaid labels. The parameters were kept uniform across all dataset.

### BRCA2 expression analysis by Western blot

Proteins (15–20 µg) extracted in radioimmunoprecipitation assay buffer (50 mm Tris–HCl, pH 7.4, 1 mm ethylenediaminetetraacetic acid, 150 mm NaCl, 0.1% sodium dodecyl sulphate, 1% Triton X-100, 0.25% sodium deoxycholate, 1 mm sodium fluoride, 1 mm orthovanadate) were separated using Bio-Rad 3–8% tris-acetate gradient gel (Bio-Rad) electrophoresis for Western blot analysis. Rabbit polyclonal BRCA2 (recognizes an epitope between residues 450-500) antibody (BETHYL lab, Cat # A303-434A-T-1, 1:2000 dilution), rabbit monoclonal GAPDH antibody (Cell Signaling technologies, Cat# 5174, 1:200,000 dilution) and mouse monoclonal Vinculin antibody (Santa Cruz biotech, Cat# sc25336, 1:200,000 dilution) were used to detect proteins. ECL plus Western blotting detection system (Amersham) was used for chemiluminescent detection. All Western blots were derived from the same experiment and were processed in parallel.

### Splicing analysis in mES cells expressing *BRCA2* variants

To detect the effect of potential splice site variants on aberrant splicing, total RNA was extracted using RNA-BEE (Tel-Test, Inc.) according to the manufacturer’s protocol. RT-PCR analysis was performed using the Qiagen one-step RT-PCR Kit (Qiagen) to detect an alternatively spliced form of *BRCA2*, following the manufacturer’s protocol. RT-PCR fragments were run on a 2% agarose gel. DNA fragments were eluted from the gels and directly sequenced using the primers used for RT-PCR. Sequence of PCR primers are provided in Supplementary Table 4.

### RAD51 foci formation assay

Cells were irradiated with 10 Gy radiation and fixed in 4% paraformaldehyde 5 h post IR in PBS followed by permeabilization with 0.1% TritonX-100 in PBS. Antibody staining and imaging was done as described previously^20^. Rabbit polyclonal RAD51 antibody (Calbiochem, Cat# PC-130, 1:100 dilution) and mouse monoclonal anti phospho-histone H2A.X (Millipore, Cat# 05-636, 1:1000 dilution) were used for staining.

### DNA fiber assay

The DNA fiber assay was carried out as described previously^53^. Briefly, cells were pulsed sequentially by 8 μg ml^−1^ CldU for 15 min followed by 90 μg ml^−1^ IdU for 15 min, and then were treated with 4 mM HU for 3 h. Cells were harvested in phosphate-buffered saline (PBS). Approximately, 3 × 10^5^ cells were lysed with lysis buffer (200 mM Tris-HCl (pH 7.4), 50 mM EDTA, 0.5% sodium dodecyl sulphate) on glass slides and incubated at room temperature for 8 min before DNA fiber was spread. Methanol and acetic acid (3:1) was used to fix the fibers followed by rehydration by PBS and denaturation in 2.5 M HCl for 1 h. Staining was done by incubating with anti-BrdU antibody (mouse, #347580, Becton Dickinson, 1:100 dilution) and anti-BrdU antibody (rat, ab6326, Abcam, 1:500 dilution) at 4 °C overnight. AlexaFluo488-conjugated anti-mouse IgG secondary antibody and AlexaFluo594-conjugated anti-rat IgG secondary antibody was used for visualization. The images were captured in Zeiss Axio Imager Z1 microscope and the fiber length was measured using ImageJ software (NIH).

### Statistical computations

All higher-level computations were carried out in the R programming language^54^. We used JAGS^55^ to fit all models using Markov chain Monte Carlo (MCMC) algorithms. We ran each MCMC algorithm for 10,000 burn-in iterations followed by another 500,000 iterations. We ignored the burn-in iterations and retained every 100th of the remaining samples for inference. We estimated marginal posterior means, standard deviations and 95% HDIs of the model parameters by their sample-based counterparts. We carried out standard output analysis to assess for convergence and to verify that the chains were run long enough.

We estimated sensitivity and specificity of the classifications using a model-based analysis. In particular, we fit a Bayesian Dirichlet-multinomial model to the conditional distributions over the classification categories given true pathogenicity status, assuming Jeffreys’ prior distribution on the vector of multinomial probabilities for each conditional distribution. We estimated sensitivity and specificity by the associated posterior expected probabilities and report 95% Bayesian high density intervals for each parameter. In addition, we report the scaled Brier score, a summary of the discordance between the known binary classifications and the PIFs that result from a given model. Computations were carried out within a reproducible research framework using the R package knitr^56^.

### Reporting summary

Further information on research design is available in the Nature Research Reporting Summary linked to this article.

## Supplementary information

Supplementary Information

Reporting Summary Checklist

## Data Availability

All raw data and the BACs or cell lines generated for this study are available upon request.

## Code availability

The code and data are available upon request.

## References

[1] Bray F (2018). Global cancer statistics 2018: GLOBOCAN estimates of incidence and mortality worldwide for 36 cancers in 185 countries. CA Cancer J. Clin..

[2] Kuchenbaecker KB (2017). Risks of breast, ovarian, and contralateral breast cancer for BRCA1 and BRCA2 mutation carriers. J. Am. Med. Assoc..

[3] Wooster R (1995). Identification of the breast cancer susceptibility gene BRCA2. Nature.

[4] Wang Y (2014). Rare variants of large effect in BRCA2 and CHEK2 affect risk of lung cancer. Nat. Genet..

[5] van Asperen CJ (2005). Cancer risks in BRCA2 families: estimates for sites other than breast and ovary. J. Med. Genet..

[6] Murphy KM (2002). Evaluation of candidate genes MAP2K4, MADH4, ACVR1B, and BRCA2 in familial pancreatic cancer: deleterious BRCA2 mutations in 17%. Cancer Res..

[7] Breast Cancer Linkage C (1999). Cancer risks in BRCA2 mutation carriers. J. Natl Cancer Inst..

[8] Cline MS (2018). BRCA Challenge: BRCA exchange as a global resource for variants in BRCA1 and BRCA2. PLoS Genet..

[9] Tavtigian SV, Samollow PB, de Silva D, Thomas A (2006). An analysis of unclassified missense substitutions in human BRCA1. Fam. Cancer.

[10] Schwarz JM, Cooper DN, Schuelke M, Seelow D (2014). MutationTaster2: mutation prediction for the deep-sequencing age. Nat. Methods.

[11] Mathe E (2006). Computational approaches for predicting the biological effect of p53 missense mutations: a comparison of three sequence analysis based methods. Nucleic Acids Res..

[12] Kumar P, Henikoff S, Ng PC (2009). Predicting the effects of coding non-synonymous variants on protein function using the SIFT algorithm. Nat. Protoc..

[13] Adzhubei IA (2010). A method and server for predicting damaging missense mutations. Nat. Methods.

[14] Spurdle AB (2012). ENIGMA—evidence-based network for the interpretation of germline mutant alleles: an international initiative to evaluate risk and clinical significance associated with sequence variation in BRCA1 and BRCA2 genes. Hum. Mutat..

[15] Guidugli L (2014). Functional assays for analysis of variants of uncertain significance in BRCA2. Hum. Mutat..

[16] Carvalho MA, Couch FJ, Monteiro AN (2007). Functional assays for BRCA1 and BRCA2. Int J. Biochem. Cell Biol..

[17] Findlay GM (2018). Accurate classification of BRCA1 variants with saturation genome editing. Nature.

[18] Ikegami M (2020). High-throughput functional evaluation of BRCA2 variants of unknown significance. Nat. Commun..

[19] Mesman RLS (2019). The functional impact of variants of uncertain significance in BRCA2. Genet. Med..

[20] Kuznetsov SG, Liu P, Sharan SK (2008). Mouse embryonic stem cell-based functional assay to evaluate mutations in BRCA2. Nat. Med..

[21] Chang S, Biswas K, Martin BK, Stauffer S, Sharan SK (2009). Expression of human BRCA1 variants in mouse ES cells allows functional analysis of BRCA1 mutations. J. Clin. Investig..

[22] Li L (2009). Functional redundancy of exon 12 of BRCA2 revealed by a comprehensive analysis of the c.6853A>G (p.I2285V) variant. Hum. Mutat..

[23] Biswas K (2012). Functional evaluation of BRCA2 variants mapping to the PALB2-binding and C-terminal DNA-binding domains using a mouse ES cell-based assay. Hum. Mol. Genet..

[24] Biswas K (2011). A comprehensive functional characterization of BRCA2 variants associated with Fanconi anemia using mouse ES cell-based assay. Blood.

[25] Bouwman P (2013). A high-throughput functional complementation assay for classification of BRCA1 missense variants. Cancer Discov..

[26] Mesman RLS (2020). Alternative mRNA splicing can attenuate the pathogenicity of presumed loss-of-function variants in BRCA2. Genet. Med..

[27] Sirisena N (2020). Functional evaluation of five BRCA2 unclassified variants identified in a Sri Lankan cohort with inherited cancer syndromes using a mouse embryonic stem cell-based assay. Breast Cancer Res..

[28] Stauffer S, Biswas K, Sharan SK (2020). Bypass of premature stop codons and generation of functional BRCA2 by exon skipping. J. Hum. Genet..

[29] Plon SE (2008). Sequence variant classification and reporting: recommendations for improving the interpretation of cancer susceptibility genetic test results. Hum. Mutat..

[30] Spurdle AB (2019). Towards controlled terminology for reporting germline cancer susceptibility variants: an ENIGMA report. J. Med. Genet..

[31] Brnich SE (2019). Recommendations for application of the functional evidence PS3/BS3 criterion using the ACMG/AMP sequence variant interpretation framework. Genome Med..

[32] Iversen ES, Couch FJ, Goldgar DE, Tavtigian SV, Monteiro AN (2011). A computational method to classify variants of uncertain significance using functional assay data with application to BRCA1. Cancer Epidemiol. Biomark. Prev..

[33] Guidugli L (2018). Assessment of the clinical relevance of BRCA2 missense variants by functional and computational approaches. Am. J. Hum. Genet..

[34] Guidugli L (2013). A classification model for BRCA2 DNA binding domain missense variants based on homology-directed repair activity. Cancer Res..

[35] Easton DF (2007). A systematic genetic assessment of 1,433 sequence variants of unknown clinical significance in the BRCA1 and BRCA2 breast cancer-predisposition genes. Am. J. Hum. Genet..

[36] Xia F (2001). Deficiency of human BRCA2 leads to impaired homologous recombination but maintains normal nonhomologous end joining. Proc. Natl Acad. Sci. USA.

[37] Schlacher K (2011). Double-strand break repair-independent role for BRCA2 in blocking stalled replication fork degradation by MRE11. Cell.

[38] Moynahan ME, Pierce AJ, Jasin M (2001). BRCA2 is required for homology-directed repair of chromosomal breaks. Mol. Cell.

[39] Sharan SK (1997). Embryonic lethality and radiation hypersensitivity mediated by Rad51 in mice lacking Brca2. Nature.

[40] D’Andrea AD, Grompe M (2003). The Fanconi anaemia/BRCA pathway. Nat. Rev. Cancer.

[41] Slob W (2002). Dose-response modeling of continuous endpoints. Toxicol. Sci..

[42] Rosenthal E, Moyes K, Arnell C, Evans B, Wenstrup RJ (2015). Incidence of BRCA1 and BRCA2 non-founder mutations in patients of Ashkenazi Jewish ancestry. Breast Cancer Res. Treat..

[43] Roy R, Chun J, Powell SN (2011). BRCA1 and BRCA2: different roles in a common pathway of genome protection. Nat. Rev. Cancer.

[44] Gudmundsdottir K, Ashworth A (2006). The roles of BRCA1 and BRCA2 and associated proteins in the maintenance of genomic stability. Oncogene.

[45] Fraile-Bethencourt E (2017). Functional classification of DNA variants by hybrid minigenes: Identification of 30 spliceogenic variants of BRCA2 exons 17 and 18. PLoS Genet..

[46] Toland AE, Andreassen PR (2017). DNA repair-related functional assays for the classification of BRCA1 and BRCA2 variants: a critical review and needs assessment. J. Med. Genet..

[47] Tavtigian SV, Greenblatt MS, Lesueur F, Byrnes GB, Group IUGVW (2008). In silico analysis of missense substitutions using sequence-alignment based methods. Hum. Mutat..

[48] Li H (2020). Classification of variants of uncertain significance in BRCA1 and BRCA2 using personal and family history of cancer from individuals in a large hereditary cancer multigene panel testing cohort. Genet. Med..

[49] Vallee MP (2016). Adding in silico assessment of potential splice aberration to the integrated evaluation of BRCA gene unclassified variants. Hum. Mutat..

[50] Yang H (2002). BRCA2 function in DNA binding and recombination from a BRCA2-DSS1-ssDNA structure. Science.

[51] Parsons MT (2019). Large scale multifactorial likelihood quantitative analysis of BRCA1 and BRCA2 variants: An ENIGMA resource to support clinical variant classification. Hum. Mutat..

[52] Biswas K, Stauffer S, Sharan SK (2012). Using recombineering to generate point mutations:galK-based positive-negative selection method. Methods Mol. Biol..

[53] Biswas K (2018). BRE/BRCC45 regulates CDC25A stability by recruiting USP7 in response to DNA damage. Nat. Commun..

[54] R: A Language and Environment for Statistical Computing. *R Foundation for Statistical Computing*. (Vienna, Austria, 2016).

[55] Plummer, M. in *Proceedings of the 3rd International Workshop on Distributed Statistical Computing* (DSC, 2003).

[56] Xie, Y. *Dynamic Documents With R and Knitr*. Second edition. (CRC Press/Taylor & Francis, 2015).

