# Supplementary material for: A computational model for classification of BRCA2 variants using mouse embryonic stem cell-based functional assays
Source: NPJ Genom Med. 2020 Dec 8;5:52. doi: 10.1038/s41525-020-00158-5 (PMC7722754; doi:10.1038/s41525-020-00158-5)
Supplement: Supplementary file 1 — Supplementary Information [file 41525_2020_158_MOESM1_ESM.pdf]

Supplementary Information:

Supplementary Figures 1-7

Supplementary Tables 1-4

Supplementary Methods

SUPPLEMENTARY FIGURES

Supplementary Figure 1: Representative Southern blots showing genotyping of HAT resistant colonies to identify *Brca2*$^{KO/KO}$ mES cells. Top panel shows the scheme of *Brca2* alleles for Southern hybridization. DNA was digested with *Eco*RV. Probe used is marked with thick line. Lower panel shows two representative Southern blots showing either full rescue or partial rescue of cell lethality as marked by loss of the conditional allele. Each lane on the Southern blot represents individual HAT resistant clone obtained after CRE mediated deletion of the conditional allele (CKO). First lane, marked as C, on both blots represents the parental PL2F7 mESC DNA containing both Conditional (CKO) and knockout (KO) allele. The asterisks mark the lanes with DNA from rescued *Brca2*$^{KO/KO}$ clones in which the top band (CKO allele) is deleted and only the lower band (KO allele) is retained.

Supplementary Figure 1

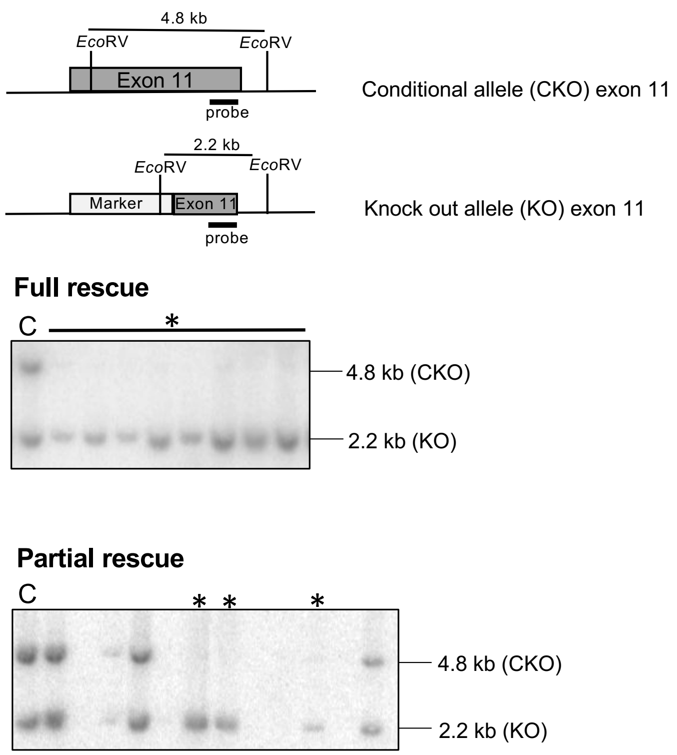

Supplementary Figure 2: *BRCA2* expression of variants that fail to complement *Brca2*$^{KO/KO}$ mES cell viability. (a) Western blot showing the expression of BRCA2 in two independent clones (marked as 1 and 2) containing the indicated variant cloned in BAC. GAPDH or Vinculin was used as loading control and marked as "control". WT represents the clone expressing BRCA2 WT BAC. (b) RT-PCR analysis detects a 627 bp fragment (ex11-14) to validate mRNA expression of the clones expressing nonsense variants using the primers from exon 11 and 14 (5′-TGGTTTTGTCAAATTCAAGAATTGG-3′ and 5′-GTGAAAGTTACAGCTACTGCTTGATTGG-3′). Lower band (531bp) represent BRCA2 splice variant lacking exon 12 (Δex12). Two independent clones are marked as 1 and 2 were further analyzed. Left lane marks the DNA size marker (1kb Plus Ladder, Life Technologies) and marked as 'M'.

Supplementary Figure 2

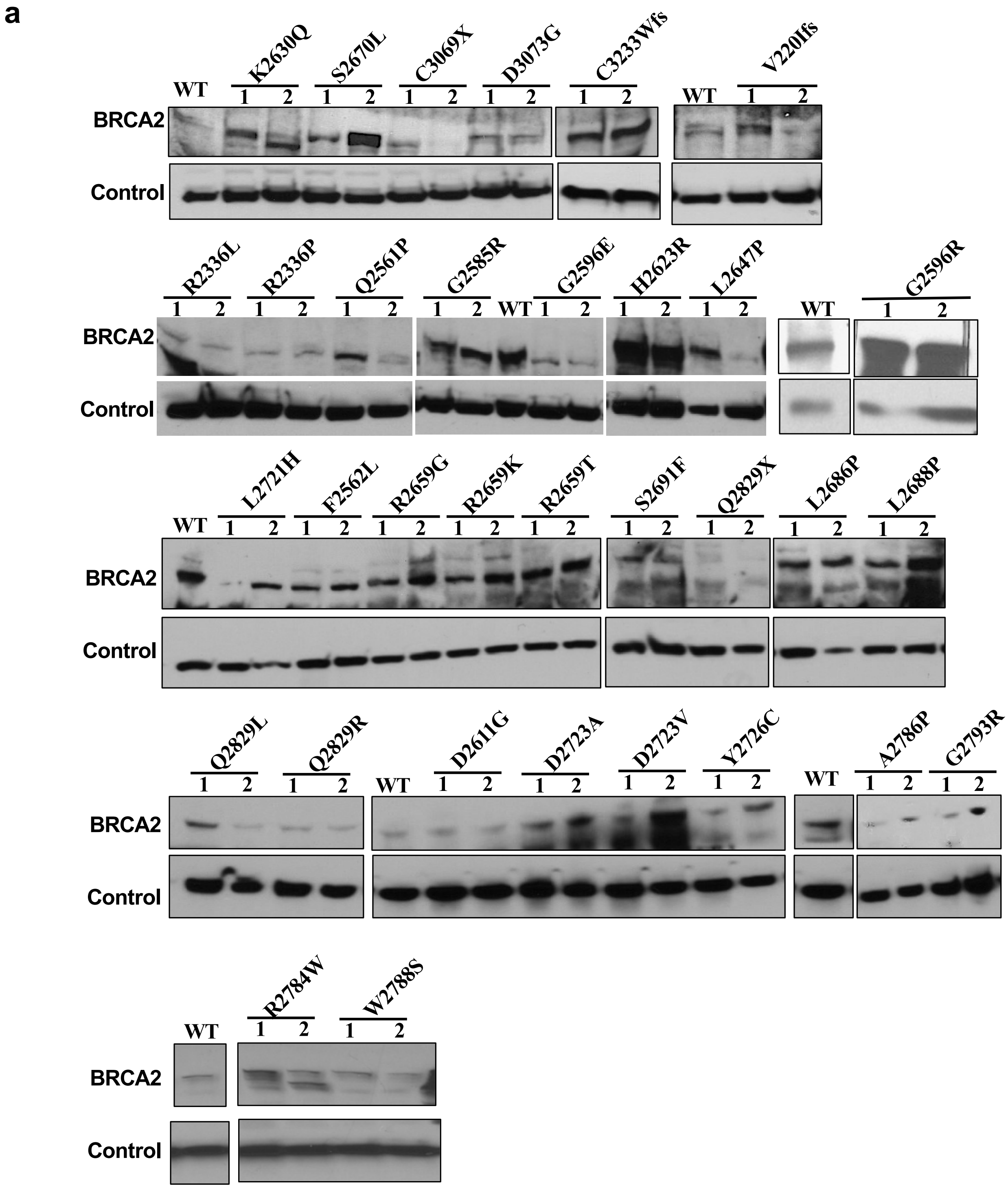

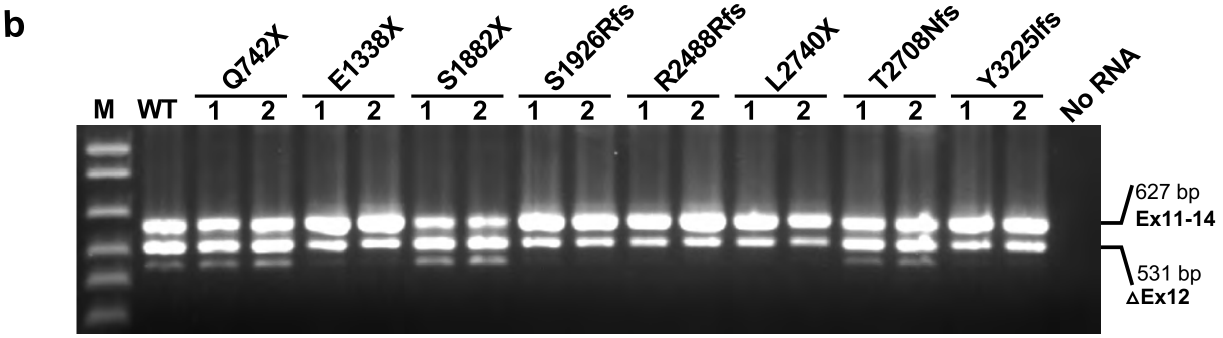

Supplementary Figure 3: Representative graphs showing response of mouse ES cells expressing BRCA2 variants to DNA damaging agents. Survival of *Brca2*$^{KO/KO}$ mouse ES cells expressing BRCA2 variants (a. P168T, b. R2488S, c. V220Ifs, d. C3233Wfs, e.R2336L, f. V2728I, g. W2788S) as determined by XTT-based assay 72 hrs. after treatment with indicated doses of camptothecin, Mitomycin C (MMC), cisplatin, methyl methanesulphonate (MMS), PARP inhibitor (olaparib), and γ-irradiation (IR). *Brca2*$^{KO/KO}$ mouse ES cells expressing WT BRCA2 was used as control. Two independently generated mES cells clones expressing each variant were used.

Supplementary Figure 3

a P168T

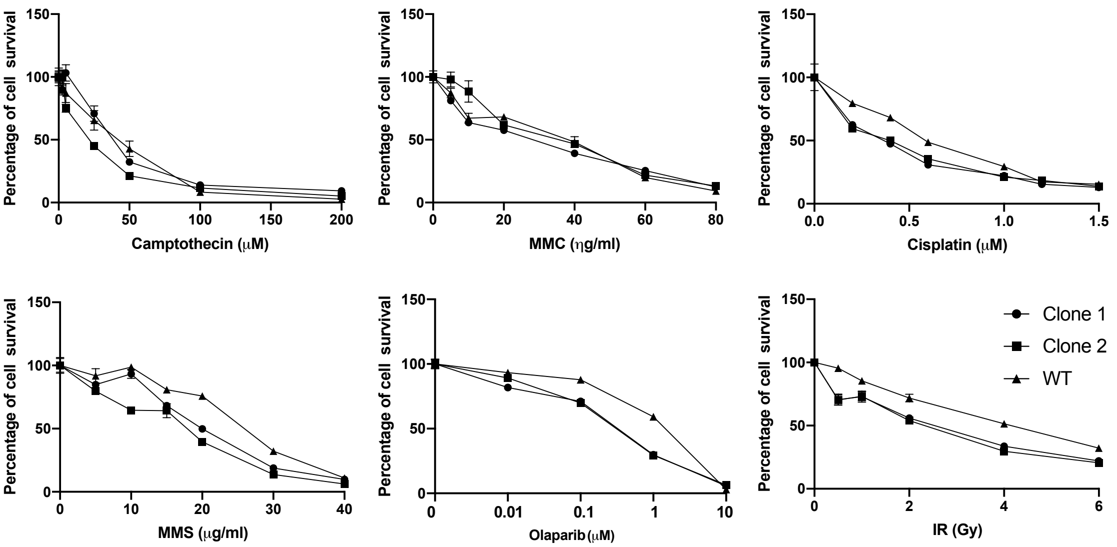

b R2488S

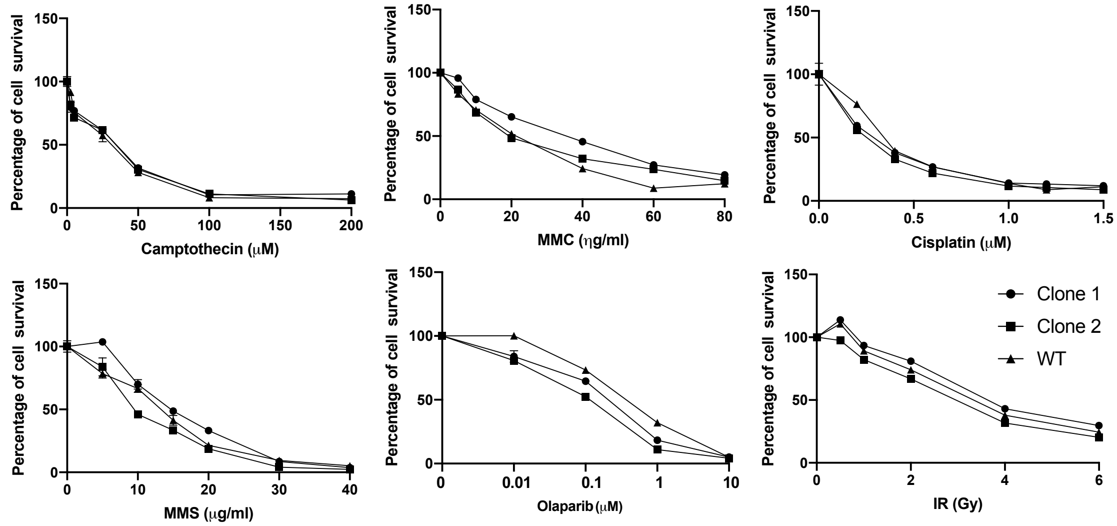

c V220Ifs

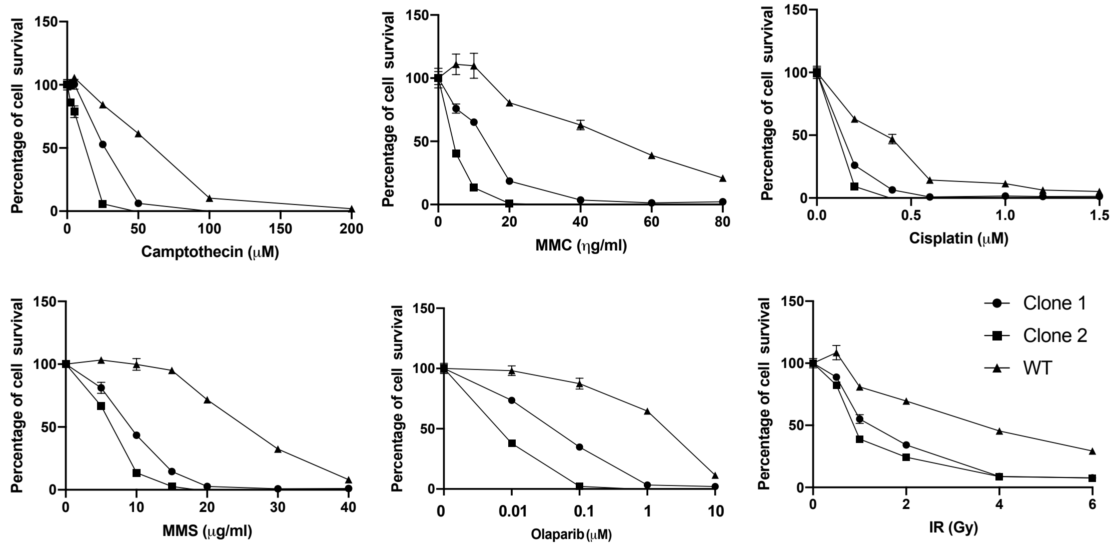

d C3233Wfs

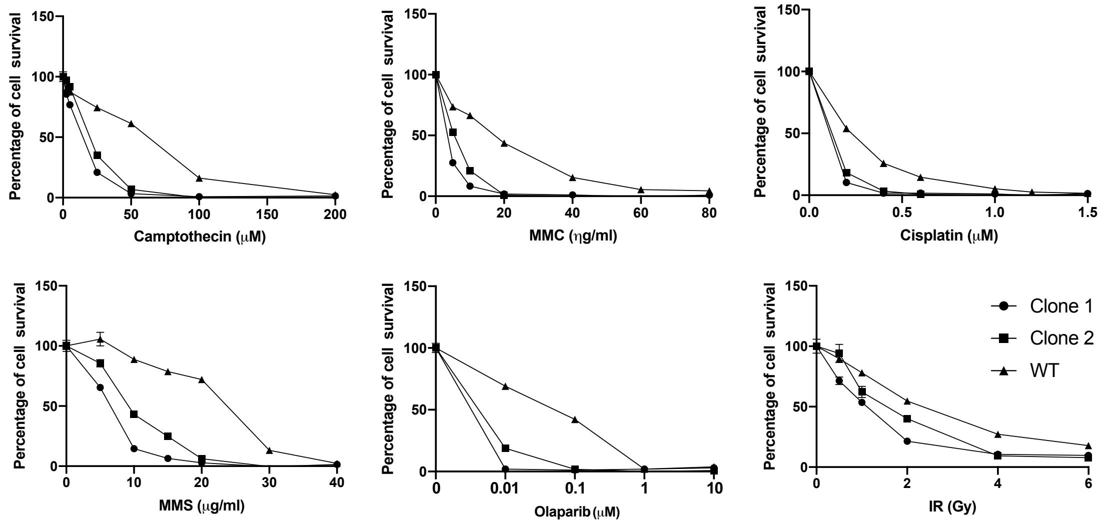

e R2336L

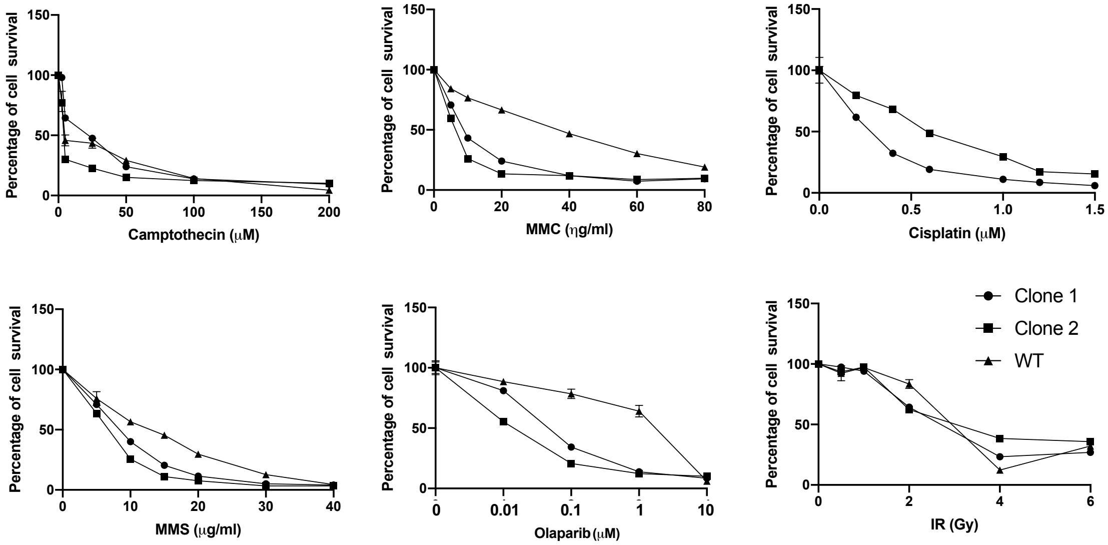

f V2728I

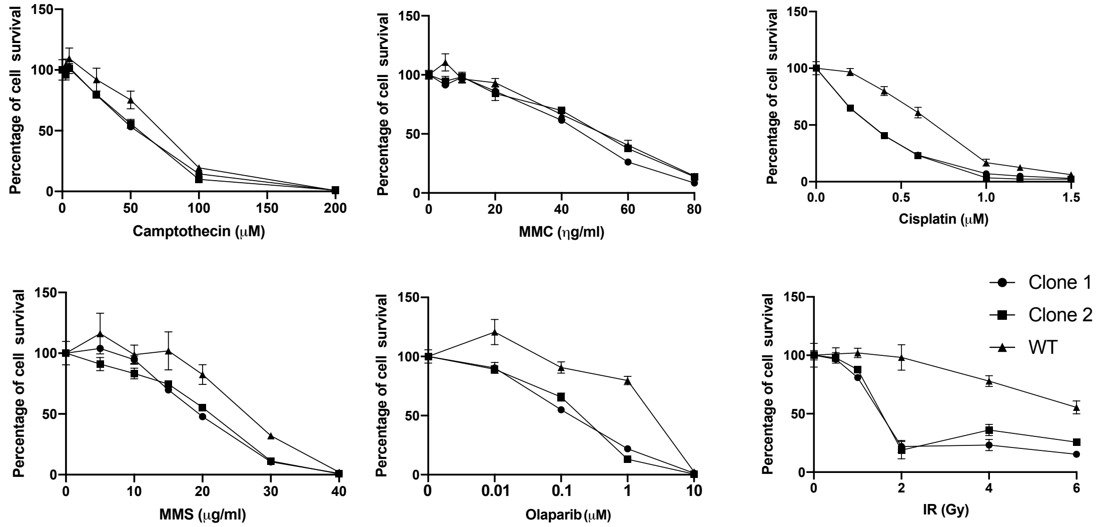

g W2788S

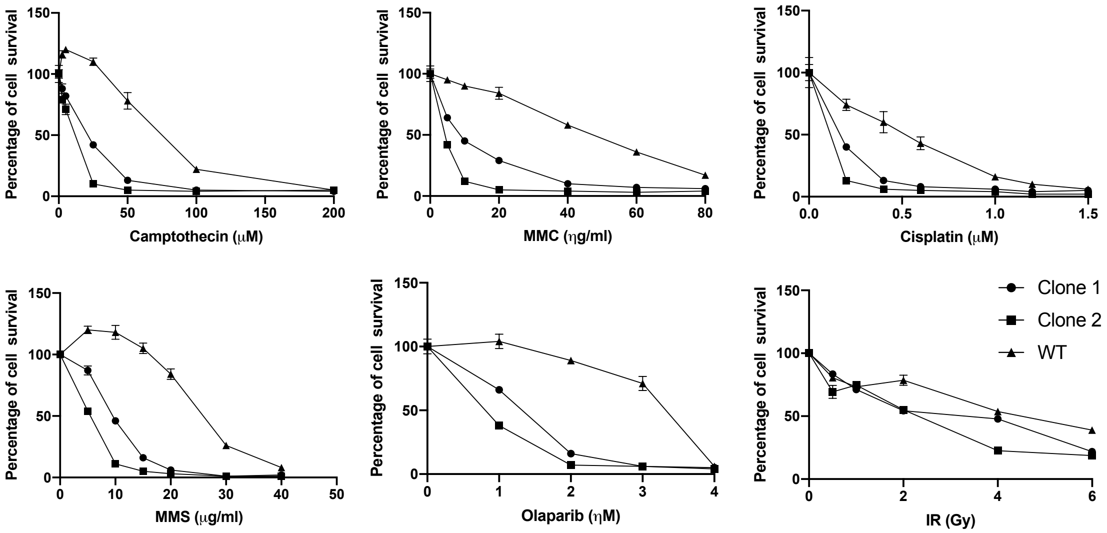

Percentage of cell survival
Camptothecin (μM)
MMC (ηg/ml)
Cisplatin (μM)
MMS (μg/ml)
Olaparib (ηM)
IR (Gy)
Clone 1
Clone 2
WT

Supplementary Figure 4: Alternative splicing analysis of variants. Alternative splicing of variants that potentially affect splicing machinery. Two independent clones were analyzed for each variant and they are marked as 1 and 2. WT BRCA2 expressing cells were used as control. The sizes of the various transcripts are marked on right side. The primers used are listed in Supplementary Table 4.

Supplementary Figure 4

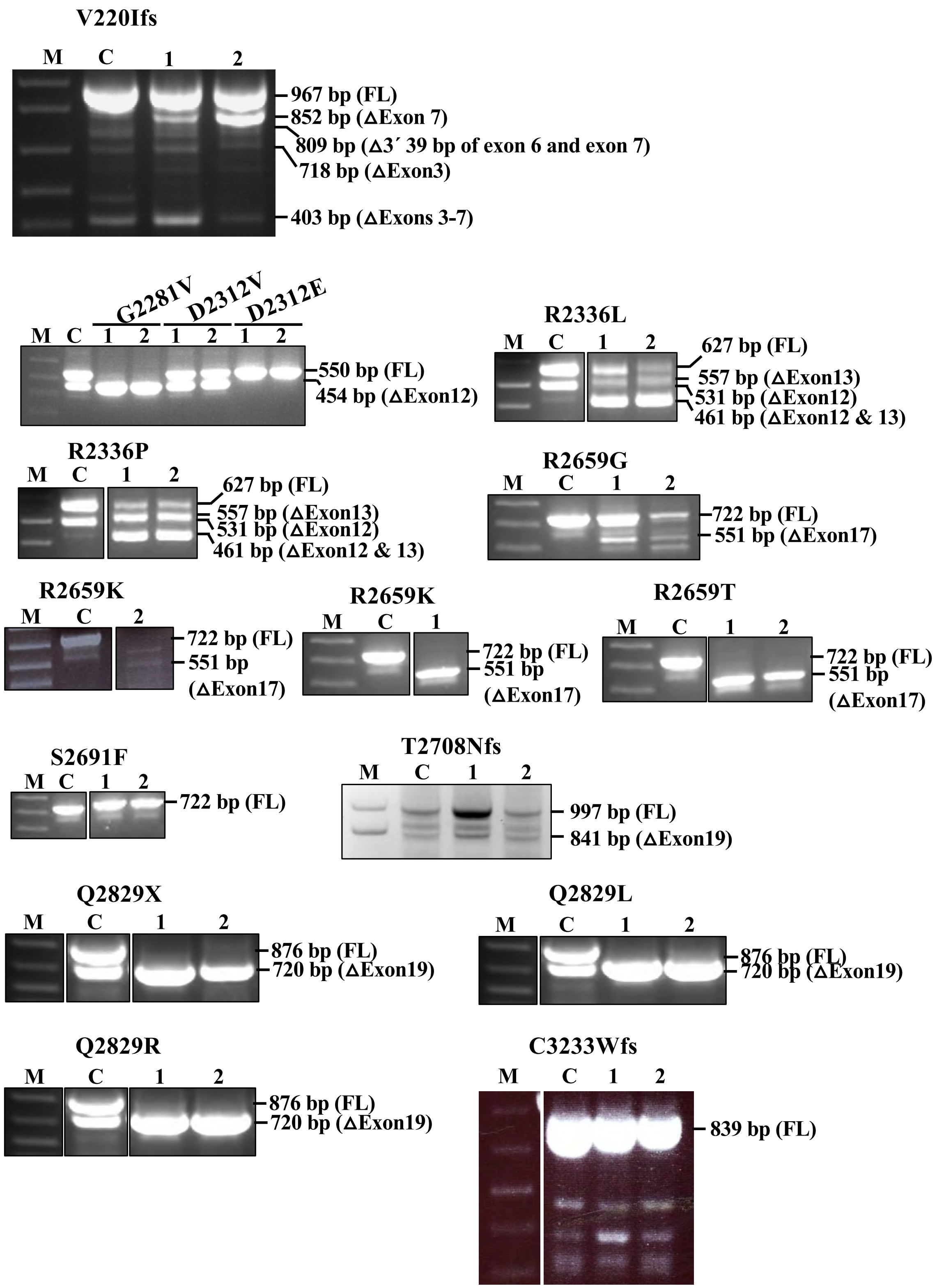

Supplementary Figure 5: Chromatogram of relevant splice variants with skipped exons. Sequence analysis of relevant alternatively spliced *BRCA2* transcripts from Fig S4, that may result in early protein truncation or internal deletion of some residues. Exons numbers are marked on the top of the chromatogram and transcripts with alternative splicing are labeled on the left.

Supplementary Figure 5

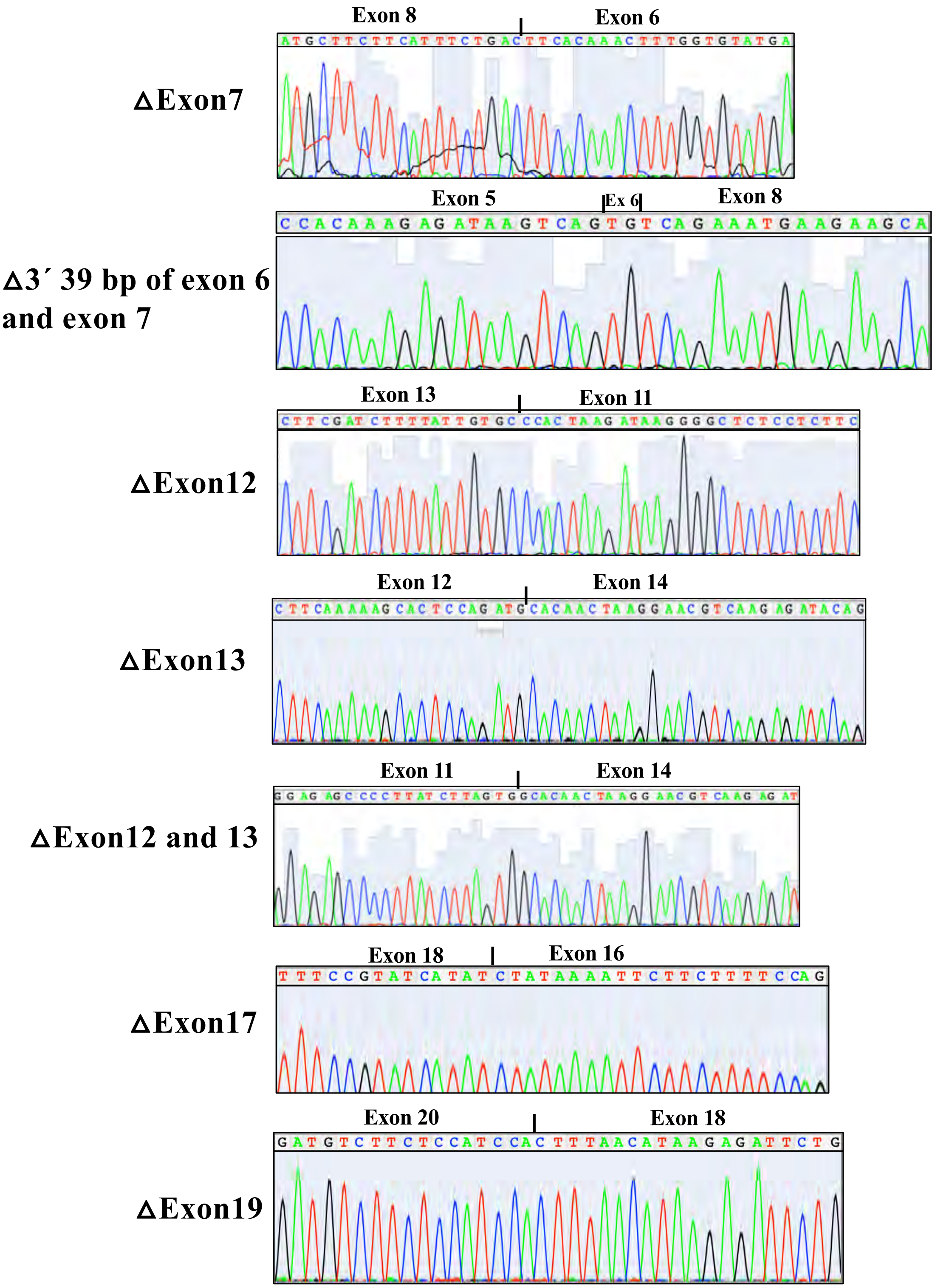

Supplementary Figure 6: Effect of C3233Wfs mutation on BRCA2 functions. (a) Ionizing radiation (IR) induced RAD51 foci formation in *Brca2*$^{KO/KO}$ mES cells expressing either WT or C3233Wfs variant. Representative images are shown. γ-H2AX was used as control for DNA damage. (b) Quantification of RAD51 foci. 150-170 nuclei were counted for each genotype listed below. Two independent clones were used for *Brca2*$^{KO/KO}$ mES cells expressing C3233Wfs variant and are marked as clone 1 and 2.**** represents P<0.0001 (Mann-Whitney test). Error bars represent mean ± s.d. c) Scatter plot for DNA fiber assay of the indicated genotype. *Brca2*$^{KO/KO}$ mES cells expressing WT and Y3308X were used as positive and negative controls respectively. Error bars represent mean ± s.d. P values were calculated by Mann-Whitney test and **** represents <0.0001.

Supplementary Figure 6

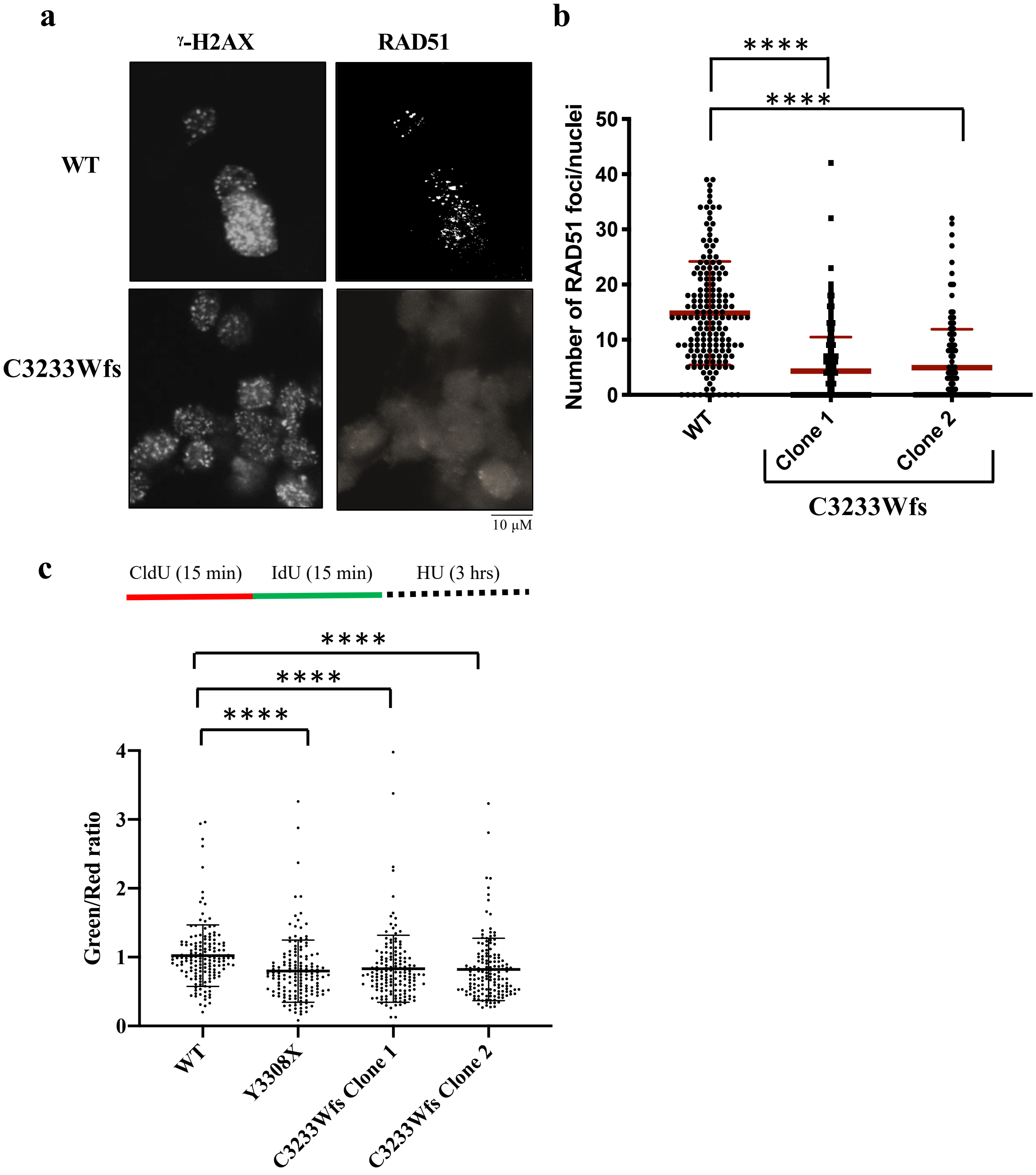

Supplementary Figure 7: *BRCA2* expression of variants. Uncropped Western blot results showing the expression of BRCA2 in two independent clones (marked as 1 and 2) expressing the indicated variant as shown in Fig S2. GAPDH or Vinculin was used as loading control as marked on the autorad.

Supp Fig 7

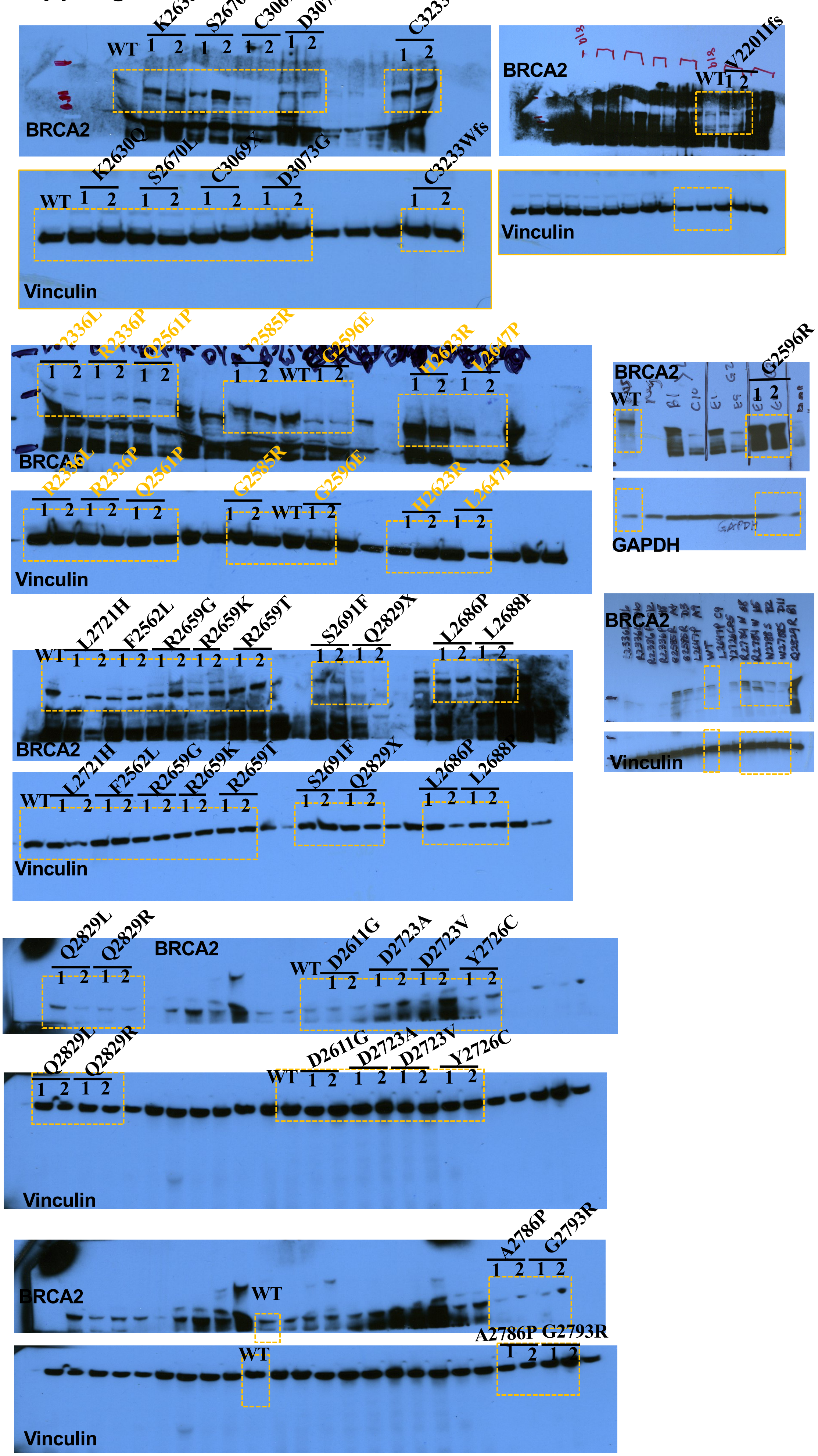
BRCA2
WT
K2630Q
S2670L
C3069X
D3073G
C3233Wfs
V2201Ifs
Vinculin
R2336L
R2336P
Q2561P
G2585R
G2596E
H2623R
L2647P
G2596R
GAPDH
L2721H
F2562L
R2659G
R2659K
R2659T
S2691F
Q2829X
L2686P
L2688F
Q2829L
Q2829R
D2611G
D2723A
D2723V
Y2726C
A2786P
G2793R
1
2

Supplementary Table 1: List of *BRCA2* variants examined by mouse ESC-based functional assay

Variant[a]	Amino acid change[b]	Ex no.	Splice Distance[c]	IARC class	Align GVGD Class	Prior Prob. (PPP)[d]	ClinVar[e]	BRCA Exchange
c.47A>G	p.Lys16Arg (K16R)	2	-21		Class C0	0.03	Uncertain (1)	
c.502C>A	p.Pro168Thr (P168T)	6	-15		Class C35	0.02	Benign (10)	Benign
c.506A>G	p.Lys169Arg (K169R)	6	-11	2	Class C25	0.3	Benign (1)/Likely Benign (5)/Uncertain (5)	
c.658_659del GT (886del GT)	p.Val220Ilefs (V220Ifs)	8					Pathogenic (29)	Pathogenic
c.796T>C	p.Phe266Leu (F266L)	10	3		Class C0	0.04	Likely Benign (3)/Uncertain (3)	
c.831 T>G	p.Asn277Lys (N277K)	10	38	2	Class C0	0.02	Benign (1)/Likely benign (7)/Uncertain (3)/Likely pathogenic (1)	
c.1354C>G	p.Leu452Val (L452V)	10	-556		Class C0	0.02		
c.1792A>G	p.Thr598Ala (T598A)	10	-118	1	Class C0	0.02	Benign (25)	Benign
c.1793C>T	p.Thr598Ile (T598I)	10	-117		Class C0	0.02	Uncertain (6)	
c.1911T>G	p.Gly637= (G637G)	11	2			0.04	Likely Benign (2)	Likely Benign
c.2224C>T	p.Gln742Ter (Q742X)	11	315			0.99	Pathogenic (12)	Pathogenic
c.2606C>T	p.Ser869Leu (S869L)	11	697	3	Class C0	0.02	Likely Benign (1)/Uncertain (2)	
c.2957A>G	p.Asn986Ser (N986S)	11	1048		Class C0	0.02	Benign (1)/Uncertain (4)/Likely Benign (3)	
c.2957A>T	p.Asn986Ile (N986I)	11	1048		Class C0	0.02	Uncertain (4)	
c.3900_3902 del	p.Met1300Ile+1301Thrdel (M1300I+1301Tdel)	11					Uncertain (1)	
c.3904_3906 del	p.Thr1302del (T1302del)	11					Pathogenic (1)/Uncertain (1)	
c.3922G>T	p.Glu1308Ter (E1308X)	11	2013			0.99	Pathogenic (16)	Pathogenic
c.4436G>C	p.Ser1479Thr (S1479T)	11	-2406		Class C0	0.02	Uncertain (4)/Likely Benign (3)	
c.4570T>G	p.Phe1524Val (F1524V)	11	-2272		Class C45	0.02	Benign (12)	Benign
c.4585G>A	p.Gly1529Arg (G1529R)	11	-2257		Class C65	0.02	Benign (22)	Benign
c.4870A>G	p.Thr1624Ala (T1624A)	11	-1972		Class C0	0.02		

c.4928T>C	p.Val1643Ala (V1643A)	11	-1914		Class C0	0.02	Benign (1)/ Uncertain (2)/Likely Benign (5)	
c.5341G>A	p.Asp1781Asn (D1781N)	11	-1501		Class C0	0.02	Uncertain (5)	
c.5342A>G	p.Asp1781Gly (D1781G)	11	-1500		Class C0	0.02	Uncertain (3)	
c.5640T>G	p.Asn1880Lys (N1880K)	11	-1202	2	Class C0	0.02	Benign (23)	Benign
c.5645C>G	p.Ser1882Ter (S1882X)	11	-1197			0.99	Pathogenic (23)	Pathogenic
c.5649A>C	p.Lys1883Asn (K1883N)	11	-1193	2	Class C0	0.02	Uncertain (3)/Likely Benign (2)	
c.5651T>C	p.Ile1884Thr (I1884T)	11	-1191	2	Class C0	0.02	Likely Benign (1)/Uncertain (1)	
c.5778delT (6005delT)	p.Ser1962Argfs (S1926Rfs)	11					Pathogenic (1)	Pathogenic
c.5896C>T	p.His1996Tyr (H1996Y)	11		2		0.02		
c.6317T>C	p.Leu2106Pro (L2106P)	11	-525	2	Class C0	0.02	Benign (2)/Likely Benign (6)/Uncertain (3)	
c.6455C>A	p.Ser2152Tyr (S2152Y)	11	-387	1	Class C15	0.02	Benign (10)	Benign
c.6842G>T	p.Gly2281Val (G2281V)	12	1		Class C65	0.34	Uncertain (4)	
c.6935A>T	p.Asp2312Val (D2312V)	12	-3	1	Class C65	0.50	Benign (16)	Benign
c.6936T>A	p.Asp2312Glu (D2312E)	12	-2		Class C35	0.04	Uncertain (1)	
c.7007G>T	p.Arg2336Leu (R2336L)	13	-1		Class C0	0.34	Pathogenic (3)/Likely Pathogenic (4)	
c.7007G>C	p.Arg2336Pro (R2336P)	13	-1		Class C0	0.34	Pathogenic (13)	
c.7232A>C	p.Lys2411Thr (K2411T)	14	-204	1	Class C65	0.02	Benign (12)	Benign
c.7447A>G	p.Ser2483G (S2483G)	15	12		Class C0	0.03	Uncertain (7)	
c.7448G>A	p.Ser2483Asn (S2483N)	15	13	2	Class C0	0.03	Uncertain (3)/Likely Benign (7)	
c.7463insAT(7691insAT)	p.Arg2488Argfs (R2488Rfs)	15						
c.7463G>A	p.Arg2488Lys (R2488K)	15	28	3	Class C0	0.03	Uncertain (3)/Likely Benign (5)	
c.7464A>C	p.Arg2488Ser (R2488S)	15	29		Class C35	0.66	Uncertain (7)	
c.7466A>G	p.Asp2489Gly (D2489G)	15	30	3	Class C15	0.30	Uncertain (8)	

c.7682A>C	p.Gln2561Pro (Q2561P)	16	65		Class C15	0.29	Uncertain (2)	
c.7684T>C	p.Phe2562Leu (F2562L)	16	67		Class C15	0.29	Uncertain (6)	
c.7753G>A	p.Gly2585Arg (G2585R)	16	-53		Class C65	0.81	Uncertain (2)/Likely Pathogenic (1)	
c.7759C>T	p.Leu2587Phe (L2587F)	16	-47		Class C15	0.29	Likely Benign (1)/Uncertain (7)/Likely Pathogenic (1)	
c.7786G>A	p.Gly2596Arg (G2596R)	16	-20		Class C65	0.81	Uncertain (8)	
c.7787G>A	p.Gly2596Glu (G2596E)	16	-19		Class C65	0.81	Uncertain (2)	
c.7832A>G	p.Asp2611Gly (D2611G)	17	27		Class C65	0.81	Uncertain (5)	
c.7865A>G	p.Asn2622Ser (N2622S)	17	60		Class C45	0.66	Uncertain (9)	
c.7868A>G	p.His2623Arg (H2623R)	17	63		Class C25	0.29	Uncertain (3)/Likely Pathogenic (4)	
c.7888A>C	p.Lys2630Gln (K2630Q)	17	83		Class C45	0.66		
c.7940T>C	p.Leu2647Pro (L2647P)	17	-37		Class C65	0.81	Likely Pathogenic (6)	
c.7975A>G	p.Arg2659Gly (R2659G)	17	-2	5	Class C65	0.81	Pathogenic (6)	Pathogenic
c.7976G>A	p.Arg2659Lys (R2659K)	17	-1	5	Class C25	0.34	Pathogenic (18)	
c.7976G>C	p.Arg2659Thr (R2659T)	17	-1	5	Class C65	0.81	Pathogenic (3)	Pathogenic
c.8009C>T	p.Ser2670Leu (S2670L)	18	33	4	Class C65	0.29	Pathogenic (2)/Likely Pathogenic (6)/Uncertain (1)	
c.8042C>G	p.Thr2681Arg (T2681R)	18	66		Class C0	0.03	Likely Benign (1)/Uncertain (9)	
c.8057T>C	p.Leu2686Pro (L2686P)	18	81		Class C65	0.66	Likely Pathogenic (5)	
c.8063T>C	p.Leu2688Pro (L2688P)	18	87		Class C65	0.81	Pathogenic (1)/Likely Pathogenic (5)	
c.8072C>T	p.Ser2691Phe (S2691F)	18	96		Class C15	0.03	Uncertain (2)	
c.8122dupA (8122 insA)	p.Thr2708Asnfs (T2708Nfs)	18						
c.8162T>A	p.Leu2721His (L2721H)	18	-170		Class C25	0.29	Uncertain (1)/Likely Pathogenic (1)	
c.8168A>C	p.Asp2723Ala (D2723A)	18	-164		Class C65	0.81	Uncertain (1)/Likely Pathogenic (4)	

c.8168A>T	p.Asp2723Val (D2723V)	18	-164		Class C65	0.81	Likely Pathogenic (4)	
c.8177A>G	p.Tyr2726Cys (Y2726C)	18	-155		Class C65	0.81	Uncertain (2)/Likely Pathogenic (2)	
c.8182G>A	p.Val2728Ile (V2728I)	18	-150	1	Class C0	0.03	Benign (31)	Benign
c.8215G>A	p.Val2739Ile (V2739I)	18	-117	3	Class C0	0.03	Benign (1)/Likely Benign (6)/Uncertain (4)	
c.8219T>A	p.Leu2740Ter (L2740X)	18	-113			0.99	Pathogenic (4)	Pathogenic
c.8350C>T	p.Arg2784Trp (R2784W)	19	19		Class C65	0.81	Uncertain (2)/Likely Pathogenic (5)	
c.8356G>C	p.Ala2786Pro (A2786P)	19	25		Class C0	0.03	Uncertain (1)	
c.8363G>C	p.Trp2788Ser (W2788S)	19	32		Class C65	0.66	Likely Pathogenic (2)	
c.8377G>A	p.Gly2793Arg (G2793R)	19	46		Class C65	0.81	Pathogenic (8)/Likely Pathogenic (3)	
c.8485C>T	p.Gln2829Ter (Q2829X)	19	-3			0.99	Pathogenic (3)	Pathogenic
c.8486A>T	p.Gln2829Leu (Q2829L)	19	-2		Class C65	0.34	Uncertain (1)	
c.8486A>G	p.Gln2829Arg (Q2829R)	19	-2	3	Class C35	0.34	Uncertain (1)/Likely Pathogenic (2)/ Pathogenic (1)	
c.9187C>T	p.Pro3063Ser (P3063S)	24	70	-	Class C0	0.03	Uncertain (1)/Likely Benign (2)	
c.9190G>A	p.Asp3064Asn (D3064N)	24	-67	-	Class C0	0.03	Benign (1)/ Likely Benign (3)/ Uncertain (2)	
c.9190G>T	p.Asp3064Tyr (D3064Y)	24	-67		Class C0	0.03	Uncertain (6)	
c.9195T>A	p.Phe3065Leu (F3065L)	24	-62		Class C0	0.03	Uncertain (3)	
c.9196C>G	p.Gln3066Glu (Q3066E)	24	-61		Class C0	0.03	Uncertain (1)	
c.9207T>A	p.Cys3069Ter (C3069X)	24	-50			0.99	Pathogenic (3)	Pathogenic
c.9218A>G	p.Asp3073Gly (D3073G)	24	-39		Class C65	0.81	Uncertain (10)	
c.9672dupA (9900insA)	p.Tyr3225Ilefs (Y3225Ifs)	27					Pathogenic (1)/Likely Pathogenic (1)	Pathogenic
c.9699_9702 delTATG (9927del4)	p.Cys3233Trpfs (C3233Wfs)	27					Pathogenic (17)	Pathogenic
c.9924C>G	p.Tyr3308Ter (Y3308X)	27	276			0.50	Pathogenic (13)	Pathogenic

[a]Variant names following HGVS nomenclature guidelines

[b]Amino acid change as per HGVS guidelines. Variant name using single letter amino acid code shown within parenthesis indicates the variant name used in the text and figures

[c]Splice Distance indicates distance of the variant from the nearest intron-exon junction.

[d]Prior probabilities of pathogenicity (PPP) based on in silico prediction of pathogenicity (http://hci-priors.hci.utah.edu/PRIORS/)

[e]Number of ClinVar entries as of 23rd September 2020 are indicated in parentheses.

SUPPLEMENTARY TABLE 2: Survival of *Brca2KO/KO* cells expressing different *BRCA2* variants in HAT media.

Variant[a]	Batch[b]	Number Survived (HAT)[c]	Total Number (M15)[d]	Percent Survived[e]
P168T	21	1406	9428	14.91
P168T	21	1186	9620	12.33
WT	21	851	6986	12.18
V220Ifs	63	379	9165	4.14
V220Ifs	63	32	5592	0.57
WT	63	1962	9300	21.10
F266L	44	534	3418	15.62
F266L	44	588	4496	13.08
WT	44	1351	8361	16.16
H1996Y	33	1303	7170	18.17
H1996Y	33	893	7524	11.87
WT	33	1045	7045	14.83
E1308X	46	0	9930	0.00
E1308X	46	0	10999	0.00
WT	46	1373	7679	17.88
V1643A	38	1416	7379	19.19
V1643A	38	1012	7966	12.70
WT	38	1146	8078	14.19
S2483N	19	1090	10204	10.68
S2483N	19	1030	8164	12.62
WT	19	835	4765	17.52
S2483G	39	4104	12111	33.89
S2483G	39	2352	10788	21.80
WT	39	1206	7696	15.67
R2488S	29	2101	8649	24.29
R2488S	29	1954	7247	26.96
WT	29	943	6943	13.58
A2487Mfs*37	15	0	8207	0.00
A2487Mfs*37	15	0	9613	0.00
WT	15	755	8323	9.07
V2739I	30	825	6538	12.62
V2739I	30	2124	8648	24.56
WT	30	944	6509	14.50
L2740X	17	0	13022	0.00
L2740X	17	0	14212	0.00
WT	17	787	11796	6.67
T2681R	53	1439	8656	16.62

Variant[a]	Batch[b]	Number Survived (HAT)[c]	Total Number (M15)[d]	Percent Survived[e]
T2681R	53	1270	7065	17.98
WT	53	1536	9247	16.61
S2670L	46	0	7392	0.00
S2670L	46	0	9521	0.00
WT	46	1373	7679	17.88
Q3066E	30	1305	6664	19.58
Q3066E	30	1953	8842	22.09
WT	30	944	6509	14.50
D3064Y	20	801	8332	9.61
D3064Y	20	1434	7570	18.94
WT	20	841	7768	10.83
P3063S	16	1798	8210	21.90
P3063S	16	519	5429	9.56
WT	16	782	8128	9.62
D3073G	57	239	7149	3.34
D3073G	57	336	10188	3.30
WT	57	1720	9231	18.63
D3064N	51	2429	12121	20.04
D3064N	51	1700	10362	16.41
WT	51	1483	7662	19.36
C3233Wfs	60	90	9847	0.91
C3233Wfs	60	432	11052	3.91
WT	60	1802	9237	19.51
S869L	10	1365	8233	16.58
S869L	10	900	5435	16.56
WT	10	602	7329	8.21
G1529R	34	2742	9563	28.67
G1529R	34	1529	10052	15.21
WT	34	1062	10256	10.35
F1524V	10	1416	10689	13.25
F1524V	10	1208	7075	17.07
WT	10	602	7329	8.21
R2336L	42	385	4082	9.43
R2336L	42	649	10062	6.45
WT	42	1283	10151	12.64
D2312V	51	2287	8121	28.16
D2312V	51	2143	9705	22.08
WT	51	1483	7662	19.36
D2312E	56	1185	8369	14.16

Variant[a]	Batch[b]	Number Survived (HAT)[c]	Total Number (M15)[d]	Percent Survived[e]
D2312E	56	1286	9075	14.17
WT	56	1635	8775	18.63
G637G	31	1516	5003	30.30
G637G	31	528	5194	10.17
WT	31	960	9455	10.15
T2708N*	52	0	9428	0.00
T2708N*	52	0	7857	0.00
WT	52	1514	9517	15.91
N2622S	13	459	7088	6.48
N2622S	13	663	6098	10.87
WT	13	694	4940	14.05
G2281V	13	1107	6679	16.57
G2281V	13	522	3287	15.88
WT	13	694	4940	14.05
Q2829L	32	0	9893	0.00
Q2829L	32	0	9521	0.00
WT	32	996	9016	11.05
Q2829R	32	0	9191	0.00
Q2829R	32	0	8722	0.00
WT	32	996	9016	11.05
H2623R	47	0	8309	0.00
H2623R	47	0	11042	0.00
WT	47	1383	9392	14.73
K1883N	55	1358	6907	19.66
K1883N	55	1689	7286	23.18
WT	55	1583	10610	14.92
Q742X	9	0	8956	0.00
Q742X	9	0	8118	0.00
WT	9	575	9771	5.88
S1882X	9	0	4478	0.00
S1882X	9	0	7372	0.00
WT	9	575	9771	5.88
K16R	2	648	4320	15.00
K16R	2	1817	4129	44.01
WT	2	372	4221	8.81
D1781N	24	297	5775	5.14
D1781N	24	471	9775	4.82
WT	24	882	10131	8.71
I1884T	8	361	5429	6.65

Variant[a]	Batch[b]	Number Survived (HAT)[c]	Total Number (M15)[d]	Percent Survived[e]
I1884T	8	355	8653	4.10
WT	8	566	6679	8.47
C3069X	28	0	9098	0.00
C3069X	28	0	12777	0.00
WT	28	930	12220	7.61
R2336P	26	120	7399	1.62
R2336P	26	347	9002	3.85
WT	26	912	10250	8.90
K2411T	64	1472	9606	15.32
K2411T	64	1949	5980	32.59
WT	64	2241	13362	16.77
D2489G	5	0	6630	0.00
D2489G	5	0	6075	0.00
WT	5	529	10817	4.89
R2488K	36	751	7425	10.11
R2488K	36	901	7438	12.11
WT	36	1066	9590	11.12
F3065L	2	334	3627	9.21
F3065L	2	308	3802	8.10
WT	2	372	4221	8.81
N1880K	7	434	8237	5.27
N1880K	7	496	8950	5.54
WT	7	553	8933	6.19
Y3308X	45	11	7831	0.14
Y3308X	45	39	6646	0.59
WT	45	1352	8302	16.29
L2688P	66	0	8003	0.00
L2688P	66	0	8220	0.00
WT	66	2665	8547	31.18
G2596E	40	0	10124	0.00
G2596E	40	40	7019	0.57
WT	40	1229	11444	10.74
L2721H	54	0	9085	0.00
L2721H	54	0	9085	0.00
WT	54	1547	7049	21.95
L2587F	54	1300	11121	11.69
L2587F	54	1415	11105	12.74
WT	54	1547	7049	21.95
D2723A	61	0	11903	0.00

Variant[a]	Batch[b]	Number Survived (HAT)[c]	Total Number (M15)[d]	Percent Survived[e]
D2723A	61	0	11636	0.00
WT	61	1856	11669	15.91
D2723V	61	0	11893	0.00
D2723V	61	0	10336	0.00
WT	61	1856	11669	15.91
G2585R	6	0	6679	0.00
G2585R	6	0	7706	0.00
WT	6	549	7831	7.01
K2630Q	50	0	8570	0.00
K2630Q	50	0	11375	0.00
WT	50	1449	11537	12.56
Q2561P	58	0	9682	0.00
Q2561P	58	0	9088	0.00
WT	58	1722	10715	16.07
N277K	1	1037	5388	19.25
N277K	1	1272	6788	18.74
WT	1	348	7303	4.77
Y3225Ifs	49	0	10098	0.00
Y3225Ifs	49	0	9956	0.00
WT	49	1436	10082	14.24
T598I	59	2078	9356	22.21
T598I	59	2205	7220	30.54
WT	59	1796	9758	18.41
S1479T	62	2442	8696	28.08
S1479T	62	2067	7181	28.78
WT	62	1889	7422	25.45
T1624A	25	862	12599	6.84
T1624A	25	852	11243	7.58
WT	25	894	10118	8.84
L452V	62	2449	8986	27.25
L452V	62	1825	6663	27.39
WT	62	1889	7422	25.45
T598A	59	1931	9012	21.43
T598A	59	2806	7897	35.53
WT	59	1796	9758	18.41
R2659K	48	0	9398	0.00
R2659K	48	0	8993	0.00
WT	48	1412	7461	18.93
R2659T	48	0	8079	0.00

Variant[a]	Batch[b]	Number Survived (HAT)[c]	Total Number (M15)[d]	Percent Survived[e]
R2659T	48	0	9336	0.00
WT	48	1412	7461	18.93
L2686P	14	0	8910	0.00
L2686P	14	0	9643	0.00
WT	14	712	9352	7.61
S1926Rfs	23	0	10913	0.00
S1926Rfs	23	0	8481	0.00
WT	23	876	10355	8.46
N986S	18	1183	8484	13.94
N986S	18	883	9108	9.69
WT	18	812	9217	8.81
F2562L	14	0	13705	0.00
F2562L	14	0	11424	0.00
WT	14	712	9352	7.61
K169R	59	3958	8864	44.65
K169R	59	2169	8841	24.53
WT	59	1796	9758	18.41
G2596R	65	0	12243	0.00
G2596R	65	0	10123	0.00
WT	65	2436	8784	27.73
W2788S	35	639	8752	7.30
W2788S	35	491	10250	4.79
WT	35	1065	10197	10.44
R2784W	35	90	5667	1.59
R2784W	35	472	2894	16.31
WT	35	1065	10197	10.44
N986I	18	1183	8484	13.94
N986I	18	883	9108	9.69
WT	18	812	9217	8.81
Q2829X	12	0	7778	0.00
Q2829X	12	0	10966	0.00
WT	12	692	4976	13.91
V2728I	22	699	8415	8.31
V2728I	22	526	9210	5.71
WT	22	876	10335	8.48
Y2726C	27	0	9610	0.00
Y2726C	27	0	10247	0.00
WT	27	913	11114	8.21
L2106P	41	1575	10362	15.20

Variant[a]	Batch[b]	Number Survived (HAT)[c]	Total Number (M15)[d]	Percent Survived[e]
L2106P	41	2458	13589	18.09
WT	41	1262	9583	13.17
S2152Y	25	875	10573	8.28
S2152Y	25	396	10916	3.63
WT	25	894	10118	8.84
A2786P	11	0	9240	0.00
A2786P	11	0	10448	0.00
WT	11	689	10233	6.73
G2793R	11	0	9260	0.00
G2793R	11	0	8069	0.00
WT	11	689	10233	6.73
D2611G	4	0	11976	0.00
D2611G	4	0	5986	0.00
WT	4	524	6514	8.04
D1781G	67	1741	13160	13.23
D1781G	67	3297	13015	25.33
WT	67	2788	13349	20.89
L2647P	43	0	9468	0.00
L2647P	43	0	8504	0.00
WT	43	1299	11217	11.58
T1302del	37	799	7722	10.35
T1302del	37	1321	5257	25.13
WT	37	1077	11748	9.17
M1300I+1301T del	37	1872	9019	20.76
M1300I+1301T del	37	1580	10718	14.74
WT	37	1077	11748	9.17
S2691F	3	0	5590	0.00
S2691F	3	0	5937	0.00
WT	3	510	6485	7.86
R2659G	3	0	5933	0.00
R2659G	3	0	6234	0.00
WT	3	510	6485	7.86

[a]*BRCA2* variant used in the cell survival assay (HAT)

[b]Batch number refers to group of variants analyzed together in one experimental with a common WT control

[c]Number of mES Cell colonies that survived HAT selection after Cre-mediated loss of condition allele of *Brca2*

[d]Total number of ES cells plated and grown in M15 media without HAT

[e]Percentage of mES cells expressing each variant that survived HAT selection

SUPPLEMENTARY TABLE 3: Results of drug sensitivity (DS) assay of BRCA2 variants

Variant[a]	Batch ID[b]	Drug[c]	Concentration[d]	Percent Survived[e]	Clone ID[f]
F266L	B1	Camp	2.5	84.51	1
F266L	B1	Camp	2.5	82.17	2
WT	B1	Camp	2.5	100.00	1
F266L	B1	Camp	5	82.13	1
F266L	B1	Camp	5	95.68	2
WT	B1	Camp	5	100.00	1
F266L	B1	Camp	25	53.22	1
F266L	B1	Camp	25	60.15	2
WT	B1	Camp	25	73.79	1
F266L	B1	Camp	50	30.17	1
F266L	B1	Camp	50	30.86	2
WT	B1	Camp	50	37.95	1
F266L	B1	Camp	100	4.60	1
F266L	B1	Camp	100	5.42	2
WT	B1	Camp	100	6.89	1
F266L	B1	Camp	200	2.22	1
F266L	B1	Camp	200	2.06	2
WT	B1	Camp	200	3.62	1
F266L	B1	MMC	5	81.20	1
F266L	B1	MMC	5	81.39	2
WT	B1	MMC	5	71.98	1
F266L	B1	MMC	10	61.57	1
F266L	B1	MMC	10	63.11	2
WT	B1	MMC	10	53.68	1
F266L	B1	MMC	20	43.39	1
F266L	B1	MMC	20	49.51	2
WT	B1	MMC	20	45.21	1
F266L	B1	MMC	40	32.85	1
F266L	B1	MMC	40	24.11	2
WT	B1	MMC	40	32.87	1
F266L	B1	MMC	60	16.53	1
F266L	B1	MMC	60	14.89	2
WT	B1	MMC	60	14.84	1
F266L	B1	MMC	80	21.90	1
F266L	B1	MMC	80	8.09	2
WT	B1	MMC	80	14.01	1
F266L	B1	Cisp	0.2	38.19	1
F266L	B1	Cisp	0.2	48.29	2

Variant[a]	Batch ID[b]	Drug[c]	Concentration[d]	Percent Survived[e]	Clone ID[f]
WT	B1	Cisp	0.2	51.44	1
F266L	B1	Cisp	0.4	14.57	1
F266L	B1	Cisp	0.4	15.69	2
WT	B1	Cisp	0.4	31.74	1
F266L	B1	Cisp	0.6	9.71	1
F266L	B1	Cisp	0.6	12.28	2
WT	B1	Cisp	0.6	25.09	1
F266L	B1	Cisp	1	11.02	1
F266L	B1	Cisp	1	6.96	2
WT	B1	Cisp	1	16.94	1
F266L	B1	Cisp	1.2	8.66	1
F266L	B1	Cisp	1.2	8.19	2
WT	B1	Cisp	1.2	16.31	1
F266L	B1	Cisp	1.5	7.48	1
F266L	B1	Cisp	1.5	3.41	2
WT	B1	Cisp	1.5	13.43	1
F266L	B1	MMS	5	68.56	1
F266L	B1	MMS	5	83.44	2
WT	B1	MMS	5	67.63	1
F266L	B1	MMS	10	51.93	1
F266L	B1	MMS	10	47.21	2
WT	B1	MMS	10	58.85	1
F266L	B1	MMS	15	38.13	1
F266L	B1	MMS	15	24.92	2
WT	B1	MMS	15	41.78	1
F266L	B1	MMS	20	14.60	1
F266L	B1	MMS	20	19.04	2
WT	B1	MMS	20	33.88	1
F266L	B1	MMS	30	7.51	1
F266L	B1	MMS	30	6.35	2
WT	B1	MMS	30	10.79	1
F266L	B1	MMS	40	5.07	1
F266L	B1	MMS	40	2.79	2
WT	B1	MMS	40	3.26	1
F266L	B1	Parp	0.01	72.59	1
F266L	B1	Parp	0.01	70.86	2
WT	B1	Parp	0.01	73.99	1
F266L	B1	Parp	0.1	56.39	1
F266L	B1	Parp	0.1	54.09	2
WT	B1	Parp	0.1	66.92	1

Variant[a]	Batch ID[b]	Drug[c]	Concentration[d]	Percent Survived[e]	Clone ID[f]
F266L	B1	Parp	1	18.70	1
F266L	B1	Parp	1	11.75	2
WT	B1	Parp	1	24.97	1
F266L	B1	Parp	10	3.54	1
F266L	B1	Parp	10	2.54	2
WT	B1	Parp	10	4.72	1
F266L	B1	IR	50	100.00	1
F266L	B1	IR	50	95.71	2
WT	B1	IR	50	100.00	1
F266L	B1	IR	100	88.61	1
F266L	B1	IR	100	90.95	2
WT	B1	IR	100	90.94	1
F266L	B1	IR	200	79.33	1
F266L	B1	IR	200	77.25	2
WT	B1	IR	200	81.68	1
F266L	B1	IR	400	58.62	1
F266L	B1	IR	400	48.33	2
WT	B1	IR	400	45.14	1
F266L	B1	IR	600	45.18	1
F266L	B1	IR	600	42.39	2
WT	B1	IR	600	36.95	1
D3064N	B2	Camp	2.5	91.59	1
D3064N	B2	Camp	2.5	98.20	2
WT	B2	Camp	2.5	93.49	1
D3064N	B2	Camp	5	94.98	1
D3064N	B2	Camp	5	87.12	2
WT	B2	Camp	5	92.32	1
D3064N	B2	Camp	25	77.29	1
D3064N	B2	Camp	25	82.68	2
WT	B2	Camp	25	77.96	1
D3064N	B2	Camp	50	26.31	1
D3064N	B2	Camp	50	46.67	2
WT	B2	Camp	50	43.91	1
D3064N	B2	Camp	100	4.26	1
D3064N	B2	Camp	100	6.86	2
WT	B2	Camp	100	7.10	1
D3064N	B2	Camp	200	3.49	1
D3064N	B2	Camp	200	4.01	2
WT	B2	Camp	200	4.42	1
D3064N	B2	MMC	5	100.00	1

Variant[a]	Batch ID[b]	Drug[c]	Concentration[d]	Percent Survived[e]	Clone ID[f]
D3064N	B2	MMC	5	88.33	2
WT	B2	MMC	5	97.28	1
D3064N	B2	MMC	10	86.53	1
D3064N	B2	MMC	10	83.64	2
WT	B2	MMC	10	92.46	1
D3064N	B2	MMC	20	60.57	1
D3064N	B2	MMC	20	75.35	2
WT	B2	MMC	20	77.91	1
D3064N	B2	MMC	40	26.51	1
D3064N	B2	MMC	40	41.55	2
WT	B2	MMC	40	44.43	1
D3064N	B2	MMC	60	13.47	1
D3064N	B2	MMC	60	23.23	2
WT	B2	MMC	60	25.42	1
D3064N	B2	MMC	80	8.87	1
D3064N	B2	MMC	80	16.47	2
WT	B2	MMC	80	17.09	1
D3064N	B2	Cisp	0.2	59.89	1
D3064N	B2	Cisp	0.2	61.33	2
WT	B2	Cisp	0.2	74.03	1
D3064N	B2	Cisp	0.4	27.95	1
D3064N	B2	Cisp	0.4	38.67	2
WT	B2	Cisp	0.4	46.00	1
D3064N	B2	Cisp	0.6	18.54	1
D3064N	B2	Cisp	0.6	24.38	2
WT	B2	Cisp	0.6	24.40	1
D3064N	B2	Cisp	1	9.76	1
D3064N	B2	Cisp	1	11.17	2
WT	B2	Cisp	1	10.22	1
D3064N	B2	Cisp	1.2	8.25	1
D3064N	B2	Cisp	1.2	6.34	2
WT	B2	Cisp	1.2	5.85	1
D3064N	B2	Cisp	1.5	8.07	1
D3064N	B2	Cisp	1.5	6.77	2
WT	B2	Cisp	1.5	5.77	1
D3064N	B2	MMS	5	83.02	1
D3064N	B2	MMS	5	100.00	2
WT	B2	MMS	5	91.37	1
D3064N	B2	MMS	10	63.20	1
D3064N	B2	MMS	10	76.55	2

Variant[a]	Batch ID[b]	Drug[c]	Concentration[d]	Percent Survived[e]	Clone ID[f]
WT	B2	MMS	10	72.64	1
D3064N	B2	MMS	15	41.67	1
D3064N	B2	MMS	15	47.96	2
WT	B2	MMS	15	51.77	1
D3064N	B2	MMS	20	25.02	1
D3064N	B2	MMS	20	35.95	2
WT	B2	MMS	20	43.22	1
D3064N	B2	MMS	30	9.10	1
D3064N	B2	MMS	30	11.93	2
WT	B2	MMS	30	15.04	1
D3064N	B2	MMS	40	3.90	1
D3064N	B2	MMS	40	4.25	2
WT	B2	MMS	40	6.05	1
D3064N	B2	Parp	0.01	74.33	1
D3064N	B2	Parp	0.01	74.33	2
WT	B2	Parp	0.01	86.96	1
D3064N	B2	Parp	0.1	63.73	1
D3064N	B2	Parp	0.1	63.73	2
WT	B2	Parp	0.1	79.80	1
D3064N	B2	Parp	1	23.98	1
D3064N	B2	Parp	1	23.98	2
WT	B2	Parp	1	42.24	1
D3064N	B2	Parp	10	6.34	1
D3064N	B2	Parp	10	6.34	2
WT	B2	Parp	10	4.96	1
D3064N	B2	IR	50	85.23	1
D3064N	B2	IR	50	80.12	2
WT	B2	IR	50	86.10	1
D3064N	B2	IR	100	82.75	1
D3064N	B2	IR	100	88.10	2
WT	B2	IR	100	83.68	1
D3064N	B2	IR	200	54.17	1
D3064N	B2	IR	200	47.81	2
WT	B2	IR	200	61.90	1
D3064N	B2	IR	400	26.17	1
D3064N	B2	IR	400	28.63	2
WT	B2	IR	400	37.99	1
D3064N	B2	IR	600	19.81	1
D3064N	B2	IR	600	21.41	2
WT	B2	IR	600	27.69	1

Variant[a]	Batch ID[b]	Drug[c]	Concentration[d]	Percent Survived[e]	Clone ID[f]
H1996Y	B3	Camp	2.5	94.91	1
H1996Y	B3	Camp	2.5	94.03	2
WT	B3	Camp	2.5	93.49	1
H1996Y	B3	Camp	5	94.25	1
H1996Y	B3	Camp	5	92.61	2
WT	B3	Camp	5	92.32	1
H1996Y	B3	Camp	25	91.32	1
H1996Y	B3	Camp	25	82.86	2
WT	B3	Camp	25	77.96	1
H1996Y	B3	Camp	50	43.21	1
H1996Y	B3	Camp	50	39.02	2
WT	B3	Camp	50	43.91	1
H1996Y	B3	Camp	100	10.57	1
H1996Y	B3	Camp	100	10.98	2
WT	B3	Camp	100	7.10	1
H1996Y	B3	Camp	200	5.19	1
H1996Y	B3	Camp	200	8.24	2
WT	B3	Camp	200	4.42	1
H1996Y	B3	MMC	5	83.53	1
H1996Y	B3	MMC	5	96.51	2
WT	B3	MMC	5	97.28	1
H1996Y	B3	MMC	10	89.53	1
H1996Y	B3	MMC	10	94.96	2
WT	B3	MMC	10	92.46	1
H1996Y	B3	MMC	20	78.68	1
H1996Y	B3	MMC	20	79.86	2
WT	B3	MMC	20	77.91	1
H1996Y	B3	MMC	40	46.41	1
H1996Y	B3	MMC	40	54.37	2
WT	B3	MMC	40	44.43	1
H1996Y	B3	MMC	60	30.62	1
H1996Y	B3	MMC	60	34.12	2
WT	B3	MMC	60	25.42	1
H1996Y	B3	MMC	80	19.48	1
H1996Y	B3	MMC	80	21.07	2
WT	B3	MMC	80	17.09	1
H1996Y	B3	Cisp	0.2	70.50	1
H1996Y	B3	Cisp	0.2	62.81	2
WT	B3	Cisp	0.2	74.03	1
H1996Y	B3	Cisp	0.4	40.83	1

Variant[a]	Batch ID[b]	Drug[c]	Concentration[d]	Percent Survived[e]	Clone ID[f]
H1996Y	B3	Cisp	0.4	34.43	2
WT	B3	Cisp	0.4	46.00	1
H1996Y	B3	Cisp	0.6	26.28	1
H1996Y	B3	Cisp	0.6	21.62	2
WT	B3	Cisp	0.6	24.40	1
H1996Y	B3	Cisp	1	13.39	1
H1996Y	B3	Cisp	1	12.10	2
WT	B3	Cisp	1	10.22	1
H1996Y	B3	Cisp	1.2	11.82	1
H1996Y	B3	Cisp	1.2	8.63	2
WT	B3	Cisp	1.2	5.85	1
H1996Y	B3	Cisp	1.5	10.00	1
H1996Y	B3	Cisp	1.5	8.81	2
WT	B3	Cisp	1.5	5.77	1
H1996Y	B3	MMS	5	71.31	1
H1996Y	B3	MMS	5	71.31	2
WT	B3	MMS	5	77.13	1
H1996Y	B3	MMS	10	52.51	1
H1996Y	B3	MMS	10	52.51	2
WT	B3	MMS	10	67.58	1
H1996Y	B3	MMS	15	37.40	1
H1996Y	B3	MMS	15	37.40	2
WT	B3	MMS	15	53.33	1
H1996Y	B3	MMS	20	20.99	1
H1996Y	B3	MMS	20	20.99	2
WT	B3	MMS	20	30.68	1
H1996Y	B3	MMS	30	6.61	1
H1996Y	B3	MMS	30	6.61	2
WT	B3	MMS	30	11.35	1
H1996Y	B3	MMS	40	2.11	1
H1996Y	B3	MMS	40	2.11	2
WT	B3	MMS	40	1.47	1
H1996Y	B3	Parp	0.01	86.38	1
H1996Y	B3	Parp	0.01	58.84	2
WT	B3	Parp	0.01	84.61	1
H1996Y	B3	Parp	0.1	70.72	1
H1996Y	B3	Parp	0.1	48.44	2
WT	B3	Parp	0.1	64.90	1
H1996Y	B3	Parp	1	40.19	1
H1996Y	B3	Parp	1	28.87	2

Variant[a]	Batch ID[b]	Drug[c]	Concentration[d]	Percent Survived[e]	Clone ID[f]
WT	B3	Parp	1	31.16	1
H1996Y	B3	Parp	10	3.90	1
H1996Y	B3	Parp	10	4.55	2
WT	B3	Parp	10	4.48	1
H1996Y	B3	IR	50	82.59	1
H1996Y	B3	IR	50	80.27	2
WT	B3	IR	50	86.10	1
H1996Y	B3	IR	100	87.65	1
H1996Y	B3	IR	100	76.76	2
WT	B3	IR	100	83.68	1
H1996Y	B3	IR	200	54.33	1
H1996Y	B3	IR	200	57.91	2
WT	B3	IR	200	61.90	1
H1996Y	B3	IR	400	41.98	1
H1996Y	B3	IR	400	36.01	2
WT	B3	IR	400	37.99	1
H1996Y	B3	IR	600	26.55	1
H1996Y	B3	IR	600	22.64	2
WT	B3	IR	600	27.69	1
D3073G	B4	Camp	2.5	86.09	1
D3073G	B4	Camp	2.5	73.68	2
WT	B4	Camp	2.5	100.00	1
D3073G	B4	Camp	5	80.26	1
D3073G	B4	Camp	5	50.79	2
WT	B4	Camp	5	100.00	1
D3073G	B4	Camp	25	22.38	1
D3073G	B4	Camp	25	12.16	2
WT	B4	Camp	25	73.79	1
D3073G	B4	Camp	50	14.57	1
D3073G	B4	Camp	50	14.31	2
WT	B4	Camp	50	37.95	1
D3073G	B4	Camp	100	13.25	1
D3073G	B4	Camp	100	16.17	2
WT	B4	Camp	100	6.89	1
D3073G	B4	Camp	200	14.70	1
D3073G	B4	Camp	200	12.30	2
WT	B4	Camp	200	3.62	1
D3073G	B4	MMC	5	63.64	1
D3073G	B4	MMC	5	38.88	2
WT	B4	MMC	5	82.50	1

Variant[a]	Batch ID[b]	Drug[c]	Concentration[d]	Percent Survived[e]	Clone ID[f]
D3073G	B4	MMC	10	41.04	1
D3073G	B4	MMC	10	20.11	2
WT	B4	MMC	10	72.70	1
D3073G	B4	MMC	20	24.57	1
D3073G	B4	MMC	20	15.04	2
WT	B4	MMC	20	59.24	1
D3073G	B4	MMC	40	15.61	1
D3073G	B4	MMC	40	12.59	2
WT	B4	MMC	40	34.54	1
D3073G	B4	MMC	60	12.98	1
D3073G	B4	MMC	60	10.69	2
WT	B4	MMC	60	18.34	1
D3073G	B4	MMC	80	8.83	1
D3073G	B4	MMC	80	10.53	2
WT	B4	MMC	80	8.17	1
D3073G	B4	Cisp	0.2	47.78	1
D3073G	B4	Cisp	0.2	27.26	2
WT	B4	Cisp	0.2	80.27	1
D3073G	B4	Cisp	0.4	22.66	1
D3073G	B4	Cisp	0.4	14.45	2
WT	B4	Cisp	0.4	57.26	1
D3073G	B4	Cisp	0.6	17.20	1
D3073G	B4	Cisp	0.6	11.78	2
WT	B4	Cisp	0.6	44.13	1
D3073G	B4	Cisp	1	15.29	1
D3073G	B4	Cisp	1	12.33	2
WT	B4	Cisp	1	26.50	1
D3073G	B4	Cisp	1.2	13.72	1
D3073G	B4	Cisp	1.2	11.63	2
WT	B4	Cisp	1.2	17.94	1
D3073G	B4	Cisp	1.5	13.38	1
D3073G	B4	Cisp	1.5	11.08	2
WT	B4	Cisp	1.5	12.06	1
D3073G	B4	MMS	5	61.38	1
D3073G	B4	MMS	5	42.72	2
WT	B4	MMS	5	88.28	1
D3073G	B4	MMS	10	25.11	1
D3073G	B4	MMS	10	13.69	2
WT	B4	MMS	10	66.47	1
D3073G	B4	MMS	15	11.21	1

Variant[a]	Batch ID[b]	Drug[c]	Concentration[d]	Percent Survived[e]	Clone ID[f]
D3073G	B4	MMS	15	9.34	2
WT	B4	MMS	15	49.07	1
D3073G	B4	MMS	20	8.92	1
D3073G	B4	MMS	20	6.80	2
WT	B4	MMS	20	37.35	1
D3073G	B4	MMS	30	5.38	1
D3073G	B4	MMS	30	5.46	2
WT	B4	MMS	30	11.21	1
D3073G	B4	MMS	40	3.15	1
D3073G	B4	MMS	40	3.72	2
WT	B4	MMS	40	3.35	1
D3073G	B4	Parp	0.01	47.62	1
D3073G	B4	Parp	0.01	24.66	2
WT	B4	Parp	0.01	92.46	1
D3073G	B4	Parp	0.1	15.90	1
D3073G	B4	Parp	0.1	8.65	2
WT	B4	Parp	0.1	66.50	1
D3073G	B4	Parp	1	12.16	1
D3073G	B4	Parp	1	9.20	2
WT	B4	Parp	1	49.84	1
D3073G	B4	Parp	10	10.87	1
D3073G	B4	Parp	10	8.65	2
WT	B4	Parp	10	3.79	1
D3073G	B4	IR	50	91.62	1
D3073G	B4	IR	50	85.15	2
WT	B4	IR	50	100.00	1
D3073G	B4	IR	100	77.54	1
D3073G	B4	IR	100	69.21	2
WT	B4	IR	100	90.94	1
D3073G	B4	IR	200	52.32	1
D3073G	B4	IR	200	37.84	2
WT	B4	IR	200	81.68	1
D3073G	B4	IR	400	27.50	1
D3073G	B4	IR	400	25.23	2
WT	B4	IR	400	45.14	1
D3073G	B4	IR	600	22.05	1
D3073G	B4	IR	600	23.07	2
WT	B4	IR	600	36.95	1
V2739I	B5	Camp	2.5	100.00	1
V2739I	B5	Camp	2.5	94.60	2

Variant[a]	Batch ID[b]	Drug[c]	Concentration[d]	Percent Survived[e]	Clone ID[f]
WT	B5	Camp	2.5	100.00	1
V2739I	B5	Camp	5	98.39	1
V2739I	B5	Camp	5	100.00	2
WT	B5	Camp	5	100.00	1
V2739I	B5	Camp	25	100.00	1
V2739I	B5	Camp	25	100.00	2
WT	B5	Camp	25	100.00	1
V2739I	B5	Camp	50	79.93	1
V2739I	B5	Camp	50	76.91	2
WT	B5	Camp	50	100.00	1
V2739I	B5	Camp	100	22.18	1
V2739I	B5	Camp	100	25.34	2
WT	B5	Camp	100	24.49	1
V2739I	B5	Camp	200	5.33	1
V2739I	B5	Camp	200	4.95	2
WT	B5	Camp	200	8.99	1
V2739I	B5	MMC	5	73.06	1
V2739I	B5	MMC	5	95.08	2
WT	B5	MMC	5	75.29	1
V2739I	B5	MMC	10	71.90	1
V2739I	B5	MMC	10	57.20	2
WT	B5	MMC	10	62.74	1
V2739I	B5	MMC	20	49.10	1
V2739I	B5	MMC	20	45.51	2
WT	B5	MMC	20	52.89	1
V2739I	B5	MMC	40	19.41	1
V2739I	B5	MMC	40	19.31	2
WT	B5	MMC	40	23.94	1
V2739I	B5	MMC	60	7.64	1
V2739I	B5	MMC	60	5.04	2
WT	B5	MMC	60	11.62	1
V2739I	B5	MMC	80	4.45	1
V2739I	B5	MMC	80	5.66	2
WT	B5	MMC	80	7.62	1
V2739I	B5	Cisp	0.2	28.81	1
V2739I	B5	Cisp	0.2	25.11	2
WT	B5	Cisp	0.2	26.13	1
V2739I	B5	Cisp	0.4	12.15	1
V2739I	B5	Cisp	0.4	8.16	2
WT	B5	Cisp	0.4	7.09	1

Variant[a]	Batch ID[b]	Drug[c]	Concentration[d]	Percent Survived[e]	Clone ID[f]
V2739I	B5	Cisp	0.6	3.81	1
V2739I	B5	Cisp	0.6	2.97	2
WT	B5	Cisp	0.6	3.55	1
V2739I	B5	Cisp	1	1.86	1
V2739I	B5	Cisp	1	2.01	2
WT	B5	Cisp	1	3.14	1
V2739I	B5	Cisp	1.2	1.24	1
V2739I	B5	Cisp	1.2	1.17	2
WT	B5	Cisp	1.2	2.05	1
V2739I	B5	Cisp	1.5	1.77	1
V2739I	B5	Cisp	1.5	2.54	2
WT	B5	Cisp	1.5	2.25	1
V2739I	B5	MMS	5	99.57	1
V2739I	B5	MMS	5	100.00	2
WT	B5	MMS	5	84.62	1
V2739I	B5	MMS	10	91.66	1
V2739I	B5	MMS	10	100.00	2
WT	B5	MMS	10	96.24	1
V2739I	B5	MMS	15	89.41	1
V2739I	B5	MMS	15	91.26	2
WT	B5	MMS	15	85.07	1
V2739I	B5	MMS	20	70.80	1
V2739I	B5	MMS	20	74.45	2
WT	B5	MMS	20	73.34	1
V2739I	B5	MMS	30	27.06	1
V2739I	B5	MMS	30	33.12	2
WT	B5	MMS	30	28.98	1
V2739I	B5	MMS	40	9.09	1
V2739I	B5	MMS	40	13.17	2
WT	B5	MMS	40	12.61	1
V2739I	B5	Parp	0.01	86.83	1
V2739I	B5	Parp	0.01	81.42	2
WT	B5	Parp	0.01	93.33	1
V2739I	B5	Parp	0.1	75.14	1
V2739I	B5	Parp	0.1	70.52	2
WT	B5	Parp	0.1	79.98	1
V2739I	B5	Parp	1	32.14	1
V2739I	B5	Parp	1	35.37	2
WT	B5	Parp	1	44.35	1
V2739I	B5	Parp	10	9.85	1

Variant[a]	Batch ID[b]	Drug[c]	Concentration[d]	Percent Survived[e]	Clone ID[f]
V2739I	B5	Parp	10	9.79	2
WT	B5	Parp	10	6.67	1
V2739I	B5	IR	50	97.60	1
V2739I	B5	IR	50	86.18	2
WT	B5	IR	50	89.24	1
V2739I	B5	IR	100	65.18	1
V2739I	B5	IR	100	68.50	2
WT	B5	IR	100	76.02	1
V2739I	B5	IR	200	39.21	1
V2739I	B5	IR	200	45.49	2
WT	B5	IR	200	52.79	1
V2739I	B5	IR	400	19.60	1
V2739I	B5	IR	400	27.21	2
WT	B5	IR	400	26.16	1
V2739I	B5	IR	600	14.81	1
V2739I	B5	IR	600	18.45	2
WT	B5	IR	600	17.85	1
Q3066E	B6	Camp	2.5	90.88	1
Q3066E	B6	Camp	2.5	94.94	2
WT	B6	Camp	2.5	100.00	1
Q3066E	B6	Camp	5	100.00	1
Q3066E	B6	Camp	5	83.42	2
WT	B6	Camp	5	92.54	1
Q3066E	B6	Camp	25	87.80	1
Q3066E	B6	Camp	25	69.48	2
WT	B6	Camp	25	74.05	1
Q3066E	B6	Camp	50	55.92	1
Q3066E	B6	Camp	50	47.76	2
WT	B6	Camp	50	47.17	1
Q3066E	B6	Camp	100	13.71	1
Q3066E	B6	Camp	100	5.06	2
WT	B6	Camp	100	15.25	1
Q3066E	B6	Camp	200	2.44	1
Q3066E	B6	Camp	200	1.00	2
WT	B6	Camp	200	6.25	1
Q3066E	B6	MMC	5	100.00	1
Q3066E	B6	MMC	5	89.74	2
WT	B6	MMC	5	92.58	1
Q3066E	B6	MMC	10	93.50	1
Q3066E	B6	MMC	10	91.51	2

Variant[a]	Batch ID[b]	Drug[c]	Concentration[d]	Percent Survived[e]	Clone ID[f]
WT	B6	MMC	10	97.62	1
Q3066E	B6	MMC	20	99.33	1
Q3066E	B6	MMC	20	80.37	2
WT	B6	MMC	20	86.32	1
Q3066E	B6	MMC	40	63.68	1
Q3066E	B6	MMC	40	55.37	2
WT	B6	MMC	40	69.03	1
Q3066E	B6	MMC	60	36.77	1
Q3066E	B6	MMC	60	27.72	2
WT	B6	MMC	60	43.27	1
Q3066E	B6	MMC	80	22.50	1
Q3066E	B6	MMC	80	20.45	2
WT	B6	MMC	80	26.93	1
Q3066E	B6	Cisp	0.2	80.30	1
Q3066E	B6	Cisp	0.2	76.80	2
WT	B6	Cisp	0.2	73.01	1
Q3066E	B6	Cisp	0.4	48.02	1
Q3066E	B6	Cisp	0.4	43.67	2
WT	B6	Cisp	0.4	42.12	1
Q3066E	B6	Cisp	0.6	30.27	1
Q3066E	B6	Cisp	0.6	25.17	2
WT	B6	Cisp	0.6	31.36	1
Q3066E	B6	Cisp	1	16.89	1
Q3066E	B6	Cisp	1	12.45	2
WT	B6	Cisp	1	18.93	1
Q3066E	B6	Cisp	1.2	11.14	1
Q3066E	B6	Cisp	1.2	10.68	2
WT	B6	Cisp	1.2	14.21	1
Q3066E	B6	Cisp	1.5	10.64	1
Q3066E	B6	Cisp	1.5	9.12	2
WT	B6	Cisp	1.5	11.85	1
Q3066E	B6	MMS	5	100.00	1
Q3066E	B6	MMS	5	96.98	2
WT	B6	MMS	5	94.24	1
Q3066E	B6	MMS	10	100.00	1
Q3066E	B6	MMS	10	94.87	2
WT	B6	MMS	10	76.70	1
Q3066E	B6	MMS	15	100.00	1
Q3066E	B6	MMS	15	93.04	2
WT	B6	MMS	15	71.84	1

Variant[a]	Batch ID[b]	Drug[c]	Concentration[d]	Percent Survived[e]	Clone ID[f]
Q3066E	B6	MMS	20	98.54	1
Q3066E	B6	MMS	20	74.07	2
WT	B6	MMS	20	64.18	1
Q3066E	B6	MMS	30	38.59	1
Q3066E	B6	MMS	30	25.65	2
WT	B6	MMS	30	19.39	1
Q3066E	B6	MMS	40	11.22	1
Q3066E	B6	MMS	40	7.94	2
WT	B6	MMS	40	6.23	1
Q3066E	B6	Parp	0.01	91.68	1
Q3066E	B6	Parp	0.01	93.97	2
WT	B6	Parp	0.01	93.33	1
Q3066E	B6	Parp	0.1	95.03	1
Q3066E	B6	Parp	0.1	91.57	2
WT	B6	Parp	0.1	79.98	1
Q3066E	B6	Parp	1	52.08	1
Q3066E	B6	Parp	1	70.21	2
WT	B6	Parp	1	44.35	1
Q3066E	B6	Parp	10	5.48	1
Q3066E	B6	Parp	10	10.92	2
WT	B6	Parp	10	6.67	1
Q3066E	B6	IR	50	81.77	1
Q3066E	B6	IR	50	85.86	2
WT	B6	IR	50	89.24	1
Q3066E	B6	IR	100	70.90	1
Q3066E	B6	IR	100	77.89	2
WT	B6	IR	100	76.02	1
Q3066E	B6	IR	200	61.71	1
Q3066E	B6	IR	200	61.02	2
WT	B6	IR	200	52.79	1
Q3066E	B6	IR	400	28.05	1
Q3066E	B6	IR	400	28.72	2
WT	B6	IR	400	26.16	1
Q3066E	B6	IR	600	19.57	1
Q3066E	B6	IR	600	18.88	2
WT	B6	IR	600	17.85	1
D3064Y	B7	Camp	2.5	96.29	1
D3064Y	B7	Camp	2.5	91.99	2
WT	B7	Camp	2.5	100.00	1
D3064Y	B7	Camp	5	100.00	1

Variant[a]	Batch ID[b]	Drug[c]	Concentration[d]	Percent Survived[e]	Clone ID[f]
D3064Y	B7	Camp	5	86.88	2
WT	B7	Camp	5	93.77	1
D3064Y	B7	Camp	25	75.07	1
D3064Y	B7	Camp	25	66.17	2
WT	B7	Camp	25	72.34	1
D3064Y	B7	Camp	50	37.99	1
D3064Y	B7	Camp	50	36.56	2
WT	B7	Camp	50	49.27	1
D3064Y	B7	Camp	100	18.68	1
D3064Y	B7	Camp	100	25.10	2
WT	B7	Camp	100	8.61	1
D3064Y	B7	Camp	200	17.73	1
D3064Y	B7	Camp	200	20.24	2
WT	B7	Camp	200	10.07	1
D3064Y	B7	MMC	5	59.12	1
D3064Y	B7	MMC	5	68.00	2
WT	B7	MMC	5	71.98	1
D3064Y	B7	MMC	10	60.69	1
D3064Y	B7	MMC	10	29.50	2
WT	B7	MMC	10	53.68	1
D3064Y	B7	MMC	20	57.23	1
D3064Y	B7	MMC	20	53.25	2
WT	B7	MMC	20	45.21	1
D3064Y	B7	MMC	40	25.79	1
D3064Y	B7	MMC	40	19.75	2
WT	B7	MMC	40	32.87	1
D3064Y	B7	MMC	60	28.30	1
D3064Y	B7	MMC	60	15.25	2
WT	B7	MMC	60	14.84	1
D3064Y	B7	MMC	80	17.92	1
D3064Y	B7	MMC	80	11.00	2
WT	B7	MMC	80	14.01	1
D3064Y	B7	Cisp	0.2	48.20	1
D3064Y	B7	Cisp	0.2	53.10	2
WT	B7	Cisp	0.2	51.44	1
D3064Y	B7	Cisp	0.4	19.66	1
D3064Y	B7	Cisp	0.4	33.21	2
WT	B7	Cisp	0.4	31.74	1
D3064Y	B7	Cisp	0.6	20.08	1
D3064Y	B7	Cisp	0.6	22.45	2

Variant[a]	Batch ID[b]	Drug[c]	Concentration[d]	Percent Survived[e]	Clone ID[f]
WT	B7	Cisp	0.6	25.09	1
D3064Y	B7	Cisp	1	10.36	1
D3064Y	B7	Cisp	1	9.31	2
WT	B7	Cisp	1	16.94	1
D3064Y	B7	Cisp	1.2	17.76	1
D3064Y	B7	Cisp	1.2	12.77	2
WT	B7	Cisp	1.2	16.31	1
D3064Y	B7	Cisp	1.5	11.84	1
D3064Y	B7	Cisp	1.5	13.87	2
WT	B7	Cisp	1.5	13.43	1
D3064Y	B7	MMS	5	58.25	1
D3064Y	B7	MMS	5	84.71	2
WT	B7	MMS	5	67.63	1
D3064Y	B7	MMS	10	46.28	1
D3064Y	B7	MMS	10	47.11	2
WT	B7	MMS	10	58.85	1
D3064Y	B7	MMS	15	41.75	1
D3064Y	B7	MMS	15	41.74	2
WT	B7	MMS	15	41.78	1
D3064Y	B7	MMS	20	37.22	1
D3064Y	B7	MMS	20	22.73	2
WT	B7	MMS	20	33.88	1
D3064Y	B7	MMS	30	13.59	1
D3064Y	B7	MMS	30	23.55	2
WT	B7	MMS	30	10.79	1
D3064Y	B7	MMS	40	5.50	1
D3064Y	B7	MMS	40	5.79	2
WT	B7	MMS	40	3.26	1
D3064Y	B7	Parp	0.01	75.46	1
D3064Y	B7	Parp	0.01	69.95	2
WT	B7	Parp	0.01	73.99	1
D3064Y	B7	Parp	0.1	52.93	1
D3064Y	B7	Parp	0.1	54.13	2
WT	B7	Parp	0.1	66.92	1
D3064Y	B7	Parp	1	15.16	1
D3064Y	B7	Parp	1	20.80	2
WT	B7	Parp	1	24.97	1
D3064Y	B7	Parp	10	2.81	1
D3064Y	B7	Parp	10	3.98	2
WT	B7	Parp	10	4.72	1

Variant[a]	Batch ID[b]	Drug[c]	Concentration[d]	Percent Survived[e]	Clone ID[f]
D3064Y	B7	IR	50	93.19	1
D3064Y	B7	IR	50	96.76	2
WT	B7	IR	50	100.00	1
D3064Y	B7	IR	100	90.93	1
D3064Y	B7	IR	100	94.45	2
WT	B7	IR	100	90.94	1
D3064Y	B7	IR	200	66.88	1
D3064Y	B7	IR	200	70.79	2
WT	B7	IR	200	81.68	1
D3064Y	B7	IR	400	48.42	1
D3064Y	B7	IR	400	52.39	2
WT	B7	IR	400	45.14	1
D3064Y	B7	IR	600	34.25	1
D3064Y	B7	IR	600	43.88	2
WT	B7	IR	600	36.95	1
P3063S	B8	Camp	2.5	100.00	1
P3063S	B8	Camp	2.5	100.00	2
WT	B8	Camp	2.5	100.00	1
P3063S	B8	Camp	5	100.00	1
P3063S	B8	Camp	5	100.00	2
WT	B8	Camp	5	100.00	1
P3063S	B8	Camp	25	100.00	1
P3063S	B8	Camp	25	100.00	2
WT	B8	Camp	25	100.00	1
P3063S	B8	Camp	50	100.00	1
P3063S	B8	Camp	50	100.00	2
WT	B8	Camp	50	100.00	1
P3063S	B8	Camp	100	59.38	1
P3063S	B8	Camp	100	41.10	2
WT	B8	Camp	100	44.17	1
P3063S	B8	Camp	200	7.39	1
P3063S	B8	Camp	200	9.48	2
WT	B8	Camp	200	10.56	1
P3063S	B8	MMC	5	87.78	1
P3063S	B8	MMC	5	95.47	2
WT	B8	MMC	5	100.00	1
P3063S	B8	MMC	10	78.72	1
P3063S	B8	MMC	10	86.96	2
WT	B8	MMC	10	92.75	1
P3063S	B8	MMC	20	69.27	1

Variant[a]	Batch ID[b]	Drug[c]	Concentration[d]	Percent Survived[e]	Clone ID[f]
P3063S	B8	MMC	20	75.00	2
WT	B8	MMC	20	81.75	1
P3063S	B8	MMC	40	29.60	1
P3063S	B8	MMC	40	38.22	2
WT	B8	MMC	40	45.13	1
P3063S	B8	MMC	60	15.24	1
P3063S	B8	MMC	60	21.20	2
WT	B8	MMC	60	22.88	1
P3063S	B8	MMC	80	12.72	1
P3063S	B8	MMC	80	12.86	2
WT	B8	MMC	80	17.50	1
P3063S	B8	Cisp	0.2	34.62	1
P3063S	B8	Cisp	0.2	31.09	2
WT	B8	Cisp	0.2	41.94	1
P3063S	B8	Cisp	0.4	11.27	1
P3063S	B8	Cisp	0.4	12.56	2
WT	B8	Cisp	0.4	16.12	1
P3063S	B8	Cisp	0.6	7.16	1
P3063S	B8	Cisp	0.6	8.07	2
WT	B8	Cisp	0.6	8.91	1
P3063S	B8	Cisp	1	4.24	1
P3063S	B8	Cisp	1	6.13	2
WT	B8	Cisp	1	6.54	1
P3063S	B8	Cisp	1.2	4.38	1
P3063S	B8	Cisp	1.2	6.58	2
WT	B8	Cisp	1.2	5.86	1
P3063S	B8	Cisp	1.5	3.05	1
P3063S	B8	Cisp	1.5	4.93	2
WT	B8	Cisp	1.5	4.85	1
P3063S	B8	MMS	5	100.00	1
P3063S	B8	MMS	5	99.03	2
WT	B8	MMS	5	100.00	1
P3063S	B8	MMS	10	100.00	1
P3063S	B8	MMS	10	100.00	2
WT	B8	MMS	10	100.00	1
P3063S	B8	MMS	15	100.00	1
P3063S	B8	MMS	15	100.00	2
WT	B8	MMS	15	95.24	1
P3063S	B8	MMS	20	98.48	1
P3063S	B8	MMS	20	100.00	2

Variant[a]	Batch ID[b]	Drug[c]	Concentration[d]	Percent Survived[e]	Clone ID[f]
WT	B8	MMS	20	100.00	1
P3063S	B8	MMS	30	72.73	1
P3063S	B8	MMS	30	55.52	2
WT	B8	MMS	30	69.84	1
P3063S	B8	MMS	40	28.37	1
P3063S	B8	MMS	40	18.99	2
WT	B8	MMS	40	44.02	1
P3063S	B8	Parp	0.01	100.00	1
P3063S	B8	Parp	0.01	95.55	2
WT	B8	Parp	0.01	100.00	1
P3063S	B8	Parp	0.1	100.00	1
P3063S	B8	Parp	0.1	78.49	2
WT	B8	Parp	0.1	100.00	1
P3063S	B8	Parp	1	59.54	1
P3063S	B8	Parp	1	30.71	2
WT	B8	Parp	1	92.96	1
P3063S	B8	Parp	10	6.06	1
P3063S	B8	Parp	10	11.42	2
WT	B8	Parp	10	12.68	1
P3063S	B8	IR	50	77.42	1
P3063S	B8	IR	50	75.36	2
WT	B8	IR	50	100.00	1
P3063S	B8	IR	100	49.26	1
P3063S	B8	IR	100	45.52	2
WT	B8	IR	100	80.46	1
P3063S	B8	IR	200	23.89	1
P3063S	B8	IR	200	21.33	2
WT	B8	IR	200	33.83	1
P3063S	B8	IR	400	8.46	1
P3063S	B8	IR	400	11.38	2
WT	B8	IR	400	14.40	1
P3063S	B8	IR	600	7.93	1
P3063S	B8	IR	600	7.62	2
WT	B8	IR	600	13.14	1
V1643A	B9	Camp	2.5	85.96	1
V1643A	B9	Camp	2.5	87.48	2
WT	B9	Camp	2.5	93.49	1
V1643A	B9	Camp	5	82.57	1
V1643A	B9	Camp	5	93.94	2
WT	B9	Camp	5	92.32	1

Variant[a]	Batch ID[b]	Drug[c]	Concentration[d]	Percent Survived[e]	Clone ID[f]
V1643A	B9	Camp	25	71.49	1
V1643A	B9	Camp	25	75.35	2
WT	B9	Camp	25	77.96	1
V1643A	B9	Camp	50	36.62	1
V1643A	B9	Camp	50	36.18	2
WT	B9	Camp	50	43.91	1
V1643A	B9	Camp	100	6.14	1
V1643A	B9	Camp	100	5.67	2
WT	B9	Camp	100	7.10	1
V1643A	B9	Camp	200	4.17	1
V1643A	B9	Camp	200	5.86	2
WT	B9	Camp	200	4.42	1
V1643A	B9	MMC	5	95.33	1
V1643A	B9	MMC	5	94.65	2
WT	B9	MMC	5	97.28	1
V1643A	B9	MMC	10	79.44	1
V1643A	B9	MMC	10	98.41	2
WT	B9	MMC	10	92.46	1
V1643A	B9	MMC	20	75.44	1
V1643A	B9	MMC	20	76.42	2
WT	B9	MMC	20	77.91	1
V1643A	B9	MMC	40	37.00	1
V1643A	B9	MMC	40	35.54	2
WT	B9	MMC	40	44.43	1
V1643A	B9	MMC	60	18.89	1
V1643A	B9	MMC	60	20.05	2
WT	B9	MMC	60	25.42	1
V1643A	B9	MMC	80	14.78	1
V1643A	B9	MMC	80	13.55	2
WT	B9	MMC	80	17.09	1
V1643A	B9	Cisp	0.2	58.81	1
V1643A	B9	Cisp	0.2	54.16	2
WT	B9	Cisp	0.2	74.03	1
V1643A	B9	Cisp	0.4	27.66	1
V1643A	B9	Cisp	0.4	27.03	2
WT	B9	Cisp	0.4	46.00	1
V1643A	B9	Cisp	0.6	16.29	1
V1643A	B9	Cisp	0.6	17.67	2
WT	B9	Cisp	0.6	24.40	1
V1643A	B9	Cisp	1	7.38	1

Variant[a]	Batch ID[b]	Drug[c]	Concentration[d]	Percent Survived[e]	Clone ID[f]
V1643A	B9	Cisp	1	9.04	2
WT	B9	Cisp	1	10.22	1
V1643A	B9	Cisp	1.2	5.94	1
V1643A	B9	Cisp	1.2	6.96	2
WT	B9	Cisp	1.2	5.85	1
V1643A	B9	Cisp	1.5	6.15	1
V1643A	B9	Cisp	1.5	7.07	2
WT	B9	Cisp	1.5	5.77	1
V1643A	B9	MMS	5	63.22	1
V1643A	B9	MMS	5	60.22	2
WT	B9	MMS	5	77.13	1
V1643A	B9	MMS	10	57.35	1
V1643A	B9	MMS	10	49.21	2
WT	B9	MMS	10	67.58	1
V1643A	B9	MMS	15	35.87	1
V1643A	B9	MMS	15	27.00	2
WT	B9	MMS	15	53.33	1
V1643A	B9	MMS	20	17.00	1
V1643A	B9	MMS	20	11.55	2
WT	B9	MMS	20	30.68	1
V1643A	B9	MMS	30	6.53	1
V1643A	B9	MMS	30	5.33	2
WT	B9	MMS	30	11.35	1
V1643A	B9	MMS	40	1.69	1
V1643A	B9	MMS	40	3.14	2
WT	B9	MMS	40	1.47	1
V1643A	B9	Parp	0.01	80.90	1
V1643A	B9	Parp	0.01	68.54	2
WT	B9	Parp	0.01	84.61	1
V1643A	B9	Parp	0.1	64.00	1
V1643A	B9	Parp	0.1	46.93	2
WT	B9	Parp	0.1	64.90	1
V1643A	B9	Parp	1	26.39	1
V1643A	B9	Parp	1	13.92	2
WT	B9	Parp	1	31.16	1
V1643A	B9	Parp	10	2.71	1
V1643A	B9	Parp	10	4.66	2
WT	B9	Parp	10	4.48	1
V1643A	B9	IR	50	77.18	1
V1643A	B9	IR	50	91.90	2

Variant[a]	Batch ID[b]	Drug[c]	Concentration[d]	Percent Survived[e]	Clone ID[f]
WT	B9	IR	50	85.33	1
V1643A	B9	IR	100	71.19	1
V1643A	B9	IR	100	81.93	2
WT	B9	IR	100	76.32	1
V1643A	B9	IR	200	57.97	1
V1643A	B9	IR	200	68.43	2
WT	B9	IR	200	55.84	1
V1643A	B9	IR	400	37.51	1
V1643A	B9	IR	400	40.60	2
WT	B9	IR	400	45.38	1
V1643A	B9	IR	600	29.49	1
V1643A	B9	IR	600	25.03	2
WT	B9	IR	600	24.75	1
P168T	B10	Camp	2.5	89.92	1
P168T	B10	Camp	2.5	100.00	2
WT	B10	Camp	2.5	89.55	1
P168T	B10	Camp	5	100.00	1
P168T	B10	Camp	5	75.10	2
WT	B10	Camp	5	87.23	1
P168T	B10	Camp	25	70.77	1
P168T	B10	Camp	25	44.88	2
WT	B10	Camp	25	65.46	1
P168T	B10	Camp	50	32.25	1
P168T	B10	Camp	50	21.29	2
WT	B10	Camp	50	42.82	1
P168T	B10	Camp	100	14.00	1
P168T	B10	Camp	100	11.55	2
WT	B10	Camp	100	8.35	1
P168T	B10	Camp	200	9.29	1
P168T	B10	Camp	200	5.22	2
WT	B10	Camp	200	2.69	1
P168T	B10	MMC	5	81.21	1
P168T	B10	MMC	5	97.95	2
WT	B10	MMC	5	87.13	1
P168T	B10	MMC	10	63.65	1
P168T	B10	MMC	10	88.48	2
WT	B10	MMC	10	67.21	1
P168T	B10	MMC	20	57.59	1
P168T	B10	MMC	20	61.79	2
WT	B10	MMC	20	68.19	1

Variant[a]	Batch ID[b]	Drug[c]	Concentration[d]	Percent Survived[e]	Clone ID[f]
P168T	B10	MMC	40	39.16	1
P168T	B10	MMC	40	46.72	2
WT	B10	MMC	40	48.14	1
P168T	B10	MMC	60	25.46	1
P168T	B10	MMC	60	21.85	2
WT	B10	MMC	60	19.99	1
P168T	B10	MMC	80	12.47	1
P168T	B10	MMC	80	13.24	2
WT	B10	MMC	80	9.14	1
P168T	B10	Cisp	0.2	62.41	1
P168T	B10	Cisp	0.2	59.48	2
WT	B10	Cisp	0.2	79.51	1
P168T	B10	Cisp	0.4	47.39	1
P168T	B10	Cisp	0.4	49.89	2
WT	B10	Cisp	0.4	68.21	1
P168T	B10	Cisp	0.6	30.78	1
P168T	B10	Cisp	0.6	35.56	2
WT	B10	Cisp	0.6	48.69	1
P168T	B10	Cisp	1	22.20	1
P168T	B10	Cisp	1	21.01	2
WT	B10	Cisp	1	29.47	1
P168T	B10	Cisp	1.2	15.39	1
P168T	B10	Cisp	1.2	18.43	2
WT	B10	Cisp	1.2	17.28	1
P168T	B10	Cisp	1.5	12.97	1
P168T	B10	Cisp	1.5	13.79	2
WT	B10	Cisp	1.5	15.48	1
P168T	B10	MMS	5	84.82	1
P168T	B10	MMS	5	79.63	2
WT	B10	MMS	5	91.78	1
P168T	B10	MMS	10	93.35	1
P168T	B10	MMS	10	64.57	2
WT	B10	MMS	10	98.70	1
P168T	B10	MMS	15	68.13	1
P168T	B10	MMS	15	64.25	2
WT	B10	MMS	15	80.78	1
P168T	B10	MMS	20	49.81	1
P168T	B10	MMS	20	39.54	2
WT	B10	MMS	20	75.86	1
P168T	B10	MMS	30	18.82	1

Variant[a]	Batch ID[b]	Drug[c]	Concentration[d]	Percent Survived[e]	Clone ID[f]
P168T	B10	MMS	30	13.65	2
WT	B10	MMS	30	32.17	1
P168T	B10	MMS	40	9.79	1
P168T	B10	MMS	40	6.18	2
WT	B10	MMS	40	10.91	1
P168T	B10	Parp	0.01	81.80	1
P168T	B10	Parp	0.01	89.20	2
WT	B10	Parp	0.01	93.34	1
P168T	B10	Parp	0.1	71.11	1
P168T	B10	Parp	0.1	69.84	2
WT	B10	Parp	0.1	87.76	1
P168T	B10	Parp	1	29.86	1
P168T	B10	Parp	1	29.39	2
WT	B10	Parp	1	59.14	1
P168T	B10	Parp	10	5.65	1
P168T	B10	Parp	10	6.40	2
WT	B10	Parp	10	2.99	1
P168T	B10	IR	50	70.62	1
P168T	B10	IR	50	70.36	2
WT	B10	IR	50	95.47	1
P168T	B10	IR	100	72.96	1
P168T	B10	IR	100	73.14	2
WT	B10	IR	100	85.43	1
P168T	B10	IR	200	55.89	1
P168T	B10	IR	200	53.88	2
WT	B10	IR	200	71.85	1
P168T	B10	IR	400	33.71	1
P168T	B10	IR	400	29.71	2
WT	B10	IR	400	51.49	1
P168T	B10	IR	600	22.10	1
P168T	B10	IR	600	20.60	2
WT	B10	IR	600	32.18	1
S2483N	B11	Camp	2.5	100.00	1
S2483N	B11	Camp	2.5	98.38	2
WT	B11	Camp	2.5	100.00	1
S2483N	B11	Camp	5	87.07	1
S2483N	B11	Camp	5	85.75	2
WT	B11	Camp	5	100.00	1
S2483N	B11	Camp	25	52.81	1
S2483N	B11	Camp	25	50.17	2

Variant[a]	Batch ID[b]	Drug[c]	Concentration[d]	Percent Survived[e]	Clone ID[f]
WT	B11	Camp	25	73.79	1
S2483N	B11	Camp	50	18.28	1
S2483N	B11	Camp	50	12.29	2
WT	B11	Camp	50	37.95	1
S2483N	B11	Camp	100	3.45	1
S2483N	B11	Camp	100	2.50	2
WT	B11	Camp	100	6.89	1
S2483N	B11	Camp	200	2.84	1
S2483N	B11	Camp	200	2.30	2
WT	B11	Camp	200	3.62	1
S2483N	B11	MMC	5	81.32	1
S2483N	B11	MMC	5	80.75	2
WT	B11	MMC	5	75.29	1
S2483N	B11	MMC	10	66.67	1
S2483N	B11	MMC	10	68.84	2
WT	B11	MMC	10	62.74	1
S2483N	B11	MMC	20	35.19	1
S2483N	B11	MMC	20	35.40	2
WT	B11	MMC	20	52.89	1
S2483N	B11	MMC	40	15.58	1
S2483N	B11	MMC	40	9.46	2
WT	B11	MMC	40	23.94	1
S2483N	B11	MMC	60	6.81	1
S2483N	B11	MMC	60	4.08	2
WT	B11	MMC	60	11.62	1
S2483N	B11	MMC	80	5.78	1
S2483N	B11	MMC	80	3.92	2
WT	B11	MMC	80	7.62	1
S2483N	B11	Cisp	0.2	27.19	1
S2483N	B11	Cisp	0.2	11.98	2
WT	B11	Cisp	0.2	26.13	1
S2483N	B11	Cisp	0.4	7.83	1
S2483N	B11	Cisp	0.4	6.90	2
WT	B11	Cisp	0.4	7.09	1
S2483N	B11	Cisp	0.6	3.26	1
S2483N	B11	Cisp	0.6	2.03	2
WT	B11	Cisp	0.6	3.55	1
S2483N	B11	Cisp	1	1.74	1
S2483N	B11	Cisp	1	1.74	2
WT	B11	Cisp	1	3.14	1

Variant[a]	Batch ID[b]	Drug[c]	Concentration[d]	Percent Survived[e]	Clone ID[f]
S2483N	B11	Cisp	1.2	1.52	1
S2483N	B11	Cisp	1.2	1.60	2
WT	B11	Cisp	1.2	2.05	1
S2483N	B11	Cisp	1.5	2.18	1
S2483N	B11	Cisp	1.5	1.89	2
WT	B11	Cisp	1.5	2.25	1
S2483N	B11	MMS	5	77.91	1
S2483N	B11	MMS	5	77.74	2
WT	B11	MMS	5	80.20	1
S2483N	B11	MMS	10	61.12	1
S2483N	B11	MMS	10	59.90	2
WT	B11	MMS	10	72.21	1
S2483N	B11	MMS	15	44.26	1
S2483N	B11	MMS	15	30.77	2
WT	B11	MMS	15	62.51	1
S2483N	B11	MMS	20	19.68	1
S2483N	B11	MMS	20	16.10	2
WT	B11	MMS	20	30.97	1
S2483N	B11	MMS	30	4.98	1
S2483N	B11	MMS	30	3.69	2
WT	B11	MMS	30	9.21	1
S2483N	B11	MMS	40	3.13	1
S2483N	B11	MMS	40	2.36	2
WT	B11	MMS	40	5.22	1
S2483N	B11	Parp	0.01	100.00	1
S2483N	B11	Parp	0.01	86.99	2
WT	B11	Parp	0.01	100.00	1
S2483N	B11	Parp	0.1	88.59	1
S2483N	B11	Parp	0.1	62.92	2
WT	B11	Parp	0.1	79.73	1
S2483N	B11	Parp	1	26.68	1
S2483N	B11	Parp	1	25.13	2
WT	B11	Parp	1	49.61	1
S2483N	B11	Parp	10	5.43	1
S2483N	B11	Parp	10	5.84	2
WT	B11	Parp	10	5.13	1
S2483N	B11	IR	50	76.69	1
S2483N	B11	IR	50	73.40	2
WT	B11	IR	50	90.45	1
S2483N	B11	IR	100	96.40	1

Variant[a]	Batch ID[b]	Drug[c]	Concentration[d]	Percent Survived[e]	Clone ID[f]
S2483N	B11	IR	100	63.64	2
WT	B11	IR	100	75.45	1
S2483N	B11	IR	200	72.97	1
S2483N	B11	IR	200	62.85	2
WT	B11	IR	200	84.38	1
S2483N	B11	IR	400	51.46	1
S2483N	B11	IR	400	48.04	2
WT	B11	IR	400	52.41	1
S2483N	B11	IR	600	27.82	1
S2483N	B11	IR	600	32.66	2
WT	B11	IR	600	28.39	1
S2483G	B12	Camp	2.5	76.70	1
S2483G	B12	Camp	2.5	78.58	2
WT	B12	Camp	2.5	91.57	1
S2483G	B12	Camp	5	68.86	1
S2483G	B12	Camp	5	72.18	2
WT	B12	Camp	5	75.55	1
S2483G	B12	Camp	25	36.51	1
S2483G	B12	Camp	25	51.60	2
WT	B12	Camp	25	57.49	1
S2483G	B12	Camp	50	17.45	1
S2483G	B12	Camp	50	16.98	2
WT	B12	Camp	50	28.14	1
S2483G	B12	Camp	100	5.60	1
S2483G	B12	Camp	100	4.78	2
WT	B12	Camp	100	8.13	1
S2483G	B12	Camp	200	4.88	1
S2483G	B12	Camp	200	3.20	2
WT	B12	Camp	200	7.45	1
S2483G	B12	MMC	5	80.30	1
S2483G	B12	MMC	5	76.27	2
WT	B12	MMC	5	83.26	1
S2483G	B12	MMC	10	63.45	1
S2483G	B12	MMC	10	60.31	2
WT	B12	MMC	10	70.70	1
S2483G	B12	MMC	20	44.81	1
S2483G	B12	MMC	20	47.63	2
WT	B12	MMC	20	51.81	1
S2483G	B12	MMC	40	19.92	1
S2483G	B12	MMC	40	20.97	2

Variant[a]	Batch ID[b]	Drug[c]	Concentration[d]	Percent Survived[e]	Clone ID[f]
WT	B12	MMC	40	24.32	1
S2483G	B12	MMC	60	10.70	1
S2483G	B12	MMC	60	13.63	2
WT	B12	MMC	60	8.82	1
S2483G	B12	MMC	80	17.90	1
S2483G	B12	MMC	80	13.46	2
WT	B12	MMC	80	12.33	1
S2483G	B12	Cisp	0.2	62.92	1
S2483G	B12	Cisp	0.2	71.67	2
WT	B12	Cisp	0.2	76.44	1
S2483G	B12	Cisp	0.4	29.53	1
S2483G	B12	Cisp	0.4	50.83	2
WT	B12	Cisp	0.4	39.60	1
S2483G	B12	Cisp	0.6	19.25	1
S2483G	B12	Cisp	0.6	37.84	2
WT	B12	Cisp	0.6	26.96	1
S2483G	B12	Cisp	1	7.55	1
S2483G	B12	Cisp	1	14.63	2
WT	B12	Cisp	1	13.75	1
S2483G	B12	Cisp	1.2	6.32	1
S2483G	B12	Cisp	1.2	9.19	2
WT	B12	Cisp	1.2	8.85	1
S2483G	B12	Cisp	1.5	7.08	1
S2483G	B12	Cisp	1.5	7.92	2
WT	B12	Cisp	1.5	11.15	1
S2483G	B12	MMS	5	58.00	1
S2483G	B12	MMS	5	85.47	2
WT	B12	MMS	5	78.43	1
S2483G	B12	MMS	10	41.75	1
S2483G	B12	MMS	10	57.12	2
WT	B12	MMS	10	66.58	1
S2483G	B12	MMS	15	13.09	1
S2483G	B12	MMS	15	26.52	2
WT	B12	MMS	15	41.26	1
S2483G	B12	MMS	20	7.87	1
S2483G	B12	MMS	20	14.03	2
WT	B12	MMS	20	21.40	1
S2483G	B12	MMS	30	2.99	1
S2483G	B12	MMS	30	3.31	2
WT	B12	MMS	30	9.38	1

Variant[a]	Batch ID[b]	Drug[c]	Concentration[d]	Percent Survived[e]	Clone ID[f]
S2483G	B12	MMS	40	1.88	1
S2483G	B12	MMS	40	2.54	2
WT	B12	MMS	40	5.20	1
S2483G	B12	Parp	0.01	75.94	1
S2483G	B12	Parp	0.01	83.01	2
WT	B12	Parp	0.01	100.00	1
S2483G	B12	Parp	0.1	54.19	1
S2483G	B12	Parp	0.1	57.22	2
WT	B12	Parp	0.1	73.35	1
S2483G	B12	Parp	1	14.24	1
S2483G	B12	Parp	1	14.31	2
WT	B12	Parp	1	32.02	1
S2483G	B12	Parp	10	3.95	1
S2483G	B12	Parp	10	3.85	2
WT	B12	Parp	10	4.90	1
S2483G	B12	IR	50	99.79	1
S2483G	B12	IR	50	100.00	2
WT	B12	IR	50	100.00	1
S2483G	B12	IR	100	79.69	1
S2483G	B12	IR	100	100.00	2
WT	B12	IR	100	89.17	1
S2483G	B12	IR	200	55.54	1
S2483G	B12	IR	200	76.92	2
WT	B12	IR	200	74.06	1
S2483G	B12	IR	400	26.84	1
S2483G	B12	IR	400	40.94	2
WT	B12	IR	400	37.94	1
S2483G	B12	IR	600	17.62	1
S2483G	B12	IR	600	26.84	2
WT	B12	IR	600	24.44	1
R2488S	B13	Camp	2.5	79.70	1
R2488S	B13	Camp	2.5	81.69	2
WT	B13	Camp	2.5	91.57	1
R2488S	B13	Camp	5	76.90	1
R2488S	B13	Camp	5	71.58	2
WT	B13	Camp	5	75.55	1
R2488S	B13	Camp	25	61.39	1
R2488S	B13	Camp	25	61.57	2
WT	B13	Camp	25	57.49	1
R2488S	B13	Camp	50	31.68	1

Variant[a]	Batch ID[b]	Drug[c]	Concentration[d]	Percent Survived[e]	Clone ID[f]
R2488S	B13	Camp	50	30.60	2
WT	B13	Camp	50	28.14	1
R2488S	B13	Camp	100	10.48	1
R2488S	B13	Camp	100	11.20	2
WT	B13	Camp	100	8.13	1
R2488S	B13	Camp	200	11.22	1
R2488S	B13	Camp	200	6.28	2
WT	B13	Camp	200	7.45	1
R2488S	B13	MMC	5	95.86	1
R2488S	B13	MMC	5	86.52	2
WT	B13	MMC	5	83.26	1
R2488S	B13	MMC	10	78.93	1
R2488S	B13	MMC	10	68.64	2
WT	B13	MMC	10	70.70	1
R2488S	B13	MMC	20	65.16	1
R2488S	B13	MMC	20	48.61	2
WT	B13	MMC	20	51.81	1
R2488S	B13	MMC	40	45.55	1
R2488S	B13	MMC	40	32.12	2
WT	B13	MMC	40	24.32	1
R2488S	B13	MMC	60	27.16	1
R2488S	B13	MMC	60	23.68	2
WT	B13	MMC	60	8.82	1
R2488S	B13	MMC	80	19.37	1
R2488S	B13	MMC	80	14.74	2
WT	B13	MMC	80	12.33	1
R2488S	B13	Cisp	0.2	59.39	1
R2488S	B13	Cisp	0.2	56.11	2
WT	B13	Cisp	0.2	76.44	1
R2488S	B13	Cisp	0.4	38.04	1
R2488S	B13	Cisp	0.4	32.88	2
WT	B13	Cisp	0.4	39.60	1
R2488S	B13	Cisp	0.6	26.81	1
R2488S	B13	Cisp	0.6	21.95	2
WT	B13	Cisp	0.6	26.96	1
R2488S	B13	Cisp	1	14.11	1
R2488S	B13	Cisp	1	11.66	2
WT	B13	Cisp	1	13.75	1
R2488S	B13	Cisp	1.2	13.25	1
R2488S	B13	Cisp	1.2	10.61	2

Variant[a]	Batch ID[b]	Drug[c]	Concentration[d]	Percent Survived[e]	Clone ID[f]
WT	B13	Cisp	1.2	8.85	1
R2488S	B13	Cisp	1.5	11.93	1
R2488S	B13	Cisp	1.5	8.76	2
WT	B13	Cisp	1.5	11.15	1
R2488S	B13	MMS	5	100.00	1
R2488S	B13	MMS	5	83.79	2
WT	B13	MMS	5	78.43	1
R2488S	B13	MMS	10	69.90	1
R2488S	B13	MMS	10	46.01	2
WT	B13	MMS	10	66.58	1
R2488S	B13	MMS	15	48.73	1
R2488S	B13	MMS	15	33.39	2
WT	B13	MMS	15	41.26	1
R2488S	B13	MMS	20	33.24	1
R2488S	B13	MMS	20	18.67	2
WT	B13	MMS	20	21.40	1
R2488S	B13	MMS	30	8.53	1
R2488S	B13	MMS	30	4.03	2
WT	B13	MMS	30	9.38	1
R2488S	B13	MMS	40	3.63	1
R2488S	B13	MMS	40	2.37	2
WT	B13	MMS	40	5.20	1
R2488S	B13	Parp	0.01	83.90	1
R2488S	B13	Parp	0.01	80.66	2
WT	B13	Parp	0.01	100.00	1
R2488S	B13	Parp	0.1	64.51	1
R2488S	B13	Parp	0.1	52.46	2
WT	B13	Parp	0.1	73.35	1
R2488S	B13	Parp	1	18.32	1
R2488S	B13	Parp	1	10.93	2
WT	B13	Parp	1	32.02	1
R2488S	B13	Parp	10	5.11	1
R2488S	B13	Parp	10	4.15	2
WT	B13	Parp	10	4.90	1
R2488S	B13	IR	50	100.00	1
R2488S	B13	IR	50	97.42	2
WT	B13	IR	50	100.00	1
R2488S	B13	IR	100	93.47	1
R2488S	B13	IR	100	82.25	2
WT	B13	IR	100	89.17	1

Variant[a]	Batch ID[b]	Drug[c]	Concentration[d]	Percent Survived[e]	Clone ID[f]
R2488S	B13	IR	200	80.88	1
R2488S	B13	IR	200	66.77	2
WT	B13	IR	200	74.06	1
R2488S	B13	IR	400	43.11	1
R2488S	B13	IR	400	31.68	2
WT	B13	IR	400	37.94	1
R2488S	B13	IR	600	29.69	1
R2488S	B13	IR	600	20.33	2
WT	B13	IR	600	24.44	1
T2681R	B14	Camp	2.5	63.30	1
T2681R	B14	Camp	2.5	62.38	2
WT	B14	Camp	2.5	60.95	1
T2681R	B14	Camp	5	53.37	1
T2681R	B14	Camp	5	38.61	2
WT	B14	Camp	5	62.14	1
T2681R	B14	Camp	25	37.88	1
T2681R	B14	Camp	25	35.77	2
WT	B14	Camp	25	44.40	1
T2681R	B14	Camp	50	14.48	1
T2681R	B14	Camp	50	11.26	2
WT	B14	Camp	50	23.79	1
T2681R	B14	Camp	100	4.46	1
T2681R	B14	Camp	100	3.96	2
WT	B14	Camp	100	9.51	1
T2681R	B14	Camp	200	3.37	1
T2681R	B14	Camp	200	3.71	2
WT	B14	Camp	200	5.85	1
T2681R	B14	MMC	5	50.69	1
T2681R	B14	MMC	5	43.78	2
WT	B14	MMC	5	51.63	1
T2681R	B14	MMC	10	48.94	1
T2681R	B14	MMC	10	39.72	2
WT	B14	MMC	10	47.53	1
T2681R	B14	MMC	20	24.10	1
T2681R	B14	MMC	20	25.59	2
WT	B14	MMC	20	32.42	1
T2681R	B14	MMC	40	9.02	1
T2681R	B14	MMC	40	9.93	2
WT	B14	MMC	40	18.15	1
T2681R	B14	MMC	60	5.89	1

Variant[a]	Batch ID[b]	Drug[c]	Concentration[d]	Percent Survived[e]	Clone ID[f]
T2681R	B14	MMC	60	3.64	2
WT	B14	MMC	60	9.34	1
T2681R	B14	MMC	80	5.24	1
T2681R	B14	MMC	80	3.22	2
WT	B14	MMC	80	8.60	1
T2681R	B14	Cisp	0.2	39.29	1
T2681R	B14	Cisp	0.2	31.16	2
WT	B14	Cisp	0.2	48.90	1
T2681R	B14	Cisp	0.4	21.65	1
T2681R	B14	Cisp	0.4	21.25	2
WT	B14	Cisp	0.4	31.66	1
T2681R	B14	Cisp	0.6	14.29	1
T2681R	B14	Cisp	0.6	16.57	2
WT	B14	Cisp	0.6	21.88	1
T2681R	B14	Cisp	1	8.59	1
T2681R	B14	Cisp	1	9.35	2
WT	B14	Cisp	1	13.45	1
T2681R	B14	Cisp	1.2	6.58	1
T2681R	B14	Cisp	1.2	7.08	2
WT	B14	Cisp	1.2	10.02	1
T2681R	B14	Cisp	1.5	6.14	1
T2681R	B14	Cisp	1.5	5.67	2
WT	B14	Cisp	1.5	11.61	1
T2681R	B14	MMS	5	48.07	1
T2681R	B14	MMS	5	55.82	2
WT	B14	MMS	5	70.61	1
T2681R	B14	MMS	10	24.97	1
T2681R	B14	MMS	10	32.93	2
WT	B14	MMS	10	46.49	1
T2681R	B14	MMS	15	12.02	1
T2681R	B14	MMS	15	15.66	2
WT	B14	MMS	15	34.89	1
T2681R	B14	MMS	20	4.49	1
T2681R	B14	MMS	20	3.21	2
WT	B14	MMS	20	17.68	1
T2681R	B14	MMS	30	1.88	1
T2681R	B14	MMS	30	1.00	2
WT	B14	MMS	30	9.02	1
T2681R	B14	MMS	40	2.19	1
T2681R	B14	MMS	40	1.00	2

Variant[a]	Batch ID[b]	Drug[c]	Concentration[d]	Percent Survived[e]	Clone ID[f]
WT	B14	MMS	40	4.45	1
T2681R	B14	Parp	0.01	58.98	1
T2681R	B14	Parp	0.01	46.04	2
WT	B14	Parp	0.01	58.12	1
T2681R	B14	Parp	0.1	29.42	1
T2681R	B14	Parp	0.1	25.93	2
WT	B14	Parp	0.1	50.61	1
T2681R	B14	Parp	1	7.54	1
T2681R	B14	Parp	1	8.46	2
WT	B14	Parp	1	24.58	1
T2681R	B14	Parp	10	1.57	1
T2681R	B14	Parp	10	6.92	2
WT	B14	Parp	10	6.14	1
T2681R	B14	IR	50	91.36	1
T2681R	B14	IR	50	94.13	2
WT	B14	IR	50	95.51	1
T2681R	B14	IR	100	65.76	1
T2681R	B14	IR	100	70.49	2
WT	B14	IR	100	71.47	1
T2681R	B14	IR	200	57.02	1
T2681R	B14	IR	200	65.19	2
WT	B14	IR	200	62.93	1
T2681R	B14	IR	400	25.91	1
T2681R	B14	IR	400	39.97	2
WT	B14	IR	400	34.62	1
T2681R	B14	IR	600	16.55	1
T2681R	B14	IR	600	26.36	2
WT	B14	IR	600	20.51	1
S869L	B15	Camp	2.5	99.64	1
S869L	B15	Camp	2.5	100.00	2
WT	B15	Camp	2.5	79.94	1
S869L	B15	Camp	5	98.71	1
S869L	B15	Camp	5	95.36	2
WT	B15	Camp	5	71.78	1
S869L	B15	Camp	25	83.12	1
S869L	B15	Camp	25	83.99	2
WT	B15	Camp	25	77.32	1
S869L	B15	Camp	50	49.57	1
S869L	B15	Camp	50	59.91	2
WT	B15	Camp	50	70.54	1

Variant[a]	Batch ID[b]	Drug[c]	Concentration[d]	Percent Survived[e]	Clone ID[f]
S869L	B15	Camp	100	16.67	1
S869L	B15	Camp	100	17.80	2
WT	B15	Camp	100	36.65	1
S869L	B15	Camp	200	3.09	1
S869L	B15	Camp	200	3.74	2
WT	B15	Camp	200	15.21	1
S869L	B15	MMC	5	88.12	1
S869L	B15	MMC	5	68.76	2
WT	B15	MMC	5	60.65	1
S869L	B15	MMC	10	70.47	1
S869L	B15	MMC	10	66.65	2
WT	B15	MMC	10	51.06	1
S869L	B15	MMC	20	64.52	1
S869L	B15	MMC	20	55.78	2
WT	B15	MMC	20	32.11	1
S869L	B15	MMC	40	59.51	1
S869L	B15	MMC	40	30.34	2
WT	B15	MMC	40	14.39	1
S869L	B15	MMC	60	25.91	1
S869L	B15	MMC	60	12.45	2
WT	B15	MMC	60	10.00	1
S869L	B15	MMC	80	15.13	1
S869L	B15	MMC	80	6.29	2
WT	B15	MMC	80	7.32	1
S869L	B15	Cisp	0.2	100.00	1
S869L	B15	Cisp	0.2	68.81	2
WT	B15	Cisp	0.2	74.97	1
S869L	B15	Cisp	0.4	57.97	1
S869L	B15	Cisp	0.4	30.30	2
WT	B15	Cisp	0.4	18.27	1
S869L	B15	Cisp	0.6	9.89	1
S869L	B15	Cisp	0.6	4.87	2
WT	B15	Cisp	0.6	9.47	1
S869L	B15	Cisp	1	22.06	1
S869L	B15	Cisp	1	10.81	2
WT	B15	Cisp	1	12.58	1
S869L	B15	Cisp	1.2	6.66	1
S869L	B15	Cisp	1.2	5.17	2
WT	B15	Cisp	1.2	11.10	1
S869L	B15	Cisp	1.5	8.07	1

Variant[a]	Batch ID[b]	Drug[c]	Concentration[d]	Percent Survived[e]	Clone ID[f]
S869L	B15	Cisp	1.5	4.46	2
WT	B15	Cisp	1.5	12.04	1
S869L	B15	MMS	5	91.20	1
S869L	B15	MMS	5	91.93	2
WT	B15	MMS	5	76.72	1
S869L	B15	MMS	10	87.45	1
S869L	B15	MMS	10	79.33	2
WT	B15	MMS	10	63.60	1
S869L	B15	MMS	15	58.72	1
S869L	B15	MMS	15	58.48	2
WT	B15	MMS	15	45.83	1
S869L	B15	MMS	20	30.55	1
S869L	B15	MMS	20	30.08	2
WT	B15	MMS	20	23.65	1
S869L	B15	MMS	30	2.44	1
S869L	B15	MMS	30	2.85	2
WT	B15	MMS	30	5.15	1
S869L	B15	MMS	40	1.00	1
S869L	B15	MMS	40	1.00	2
WT	B15	MMS	40	3.06	1
S869L	B15	Parp	0.01	79.95	1
S869L	B15	Parp	0.01	74.52	2
WT	B15	Parp	0.01	88.13	1
S869L	B15	Parp	0.1	56.80	1
S869L	B15	Parp	0.1	55.34	2
WT	B15	Parp	0.1	59.61	1
S869L	B15	Parp	1	23.78	1
S869L	B15	Parp	1	33.87	2
WT	B15	Parp	1	38.13	1
S869L	B15	Parp	10	2.10	1
S869L	B15	Parp	10	1.77	2
WT	B15	Parp	10	2.10	1
S869L	B15	IR	50	87.88	1
S869L	B15	IR	50	99.25	2
WT	B15	IR	50	93.55	1
S869L	B15	IR	100	73.82	1
S869L	B15	IR	100	82.31	2
WT	B15	IR	100	78.21	1
S869L	B15	IR	200	76.96	1
S869L	B15	IR	200	76.25	2

Variant[a]	Batch ID[b]	Drug[c]	Concentration[d]	Percent Survived[e]	Clone ID[f]
WT	B15	IR	200	84.45	1
S869L	B15	IR	400	49.86	1
S869L	B15	IR	400	54.99	2
WT	B15	IR	400	62.30	1
S869L	B15	IR	600	32.90	1
S869L	B15	IR	600	34.81	2
WT	B15	IR	600	44.77	1
G1529R	B16	Camp	2.5	100.00	1
G1529R	B16	Camp	2.5	100.00	2
WT	B16	Camp	2.5	79.94	1
G1529R	B16	Camp	5	97.85	1
G1529R	B16	Camp	5	94.55	2
WT	B16	Camp	5	71.78	1
G1529R	B16	Camp	25	84.26	1
G1529R	B16	Camp	25	95.45	2
WT	B16	Camp	25	77.32	1
G1529R	B16	Camp	50	66.74	1
G1529R	B16	Camp	50	78.08	2
WT	B16	Camp	50	70.54	1
G1529R	B16	Camp	100	39.05	1
G1529R	B16	Camp	100	42.68	2
WT	B16	Camp	100	36.65	1
G1529R	B16	Camp	200	5.49	1
G1529R	B16	Camp	200	15.52	2
WT	B16	Camp	200	15.21	1
G1529R	B16	MMC	5	92.40	1
G1529R	B16	MMC	5	95.46	2
WT	B16	MMC	5	60.65	1
G1529R	B16	MMC	10	89.56	1
G1529R	B16	MMC	10	68.80	2
WT	B16	MMC	10	51.06	1
G1529R	B16	MMC	20	77.05	1
G1529R	B16	MMC	20	72.90	2
WT	B16	MMC	20	32.11	1
G1529R	B16	MMC	40	53.73	1
G1529R	B16	MMC	40	54.61	2
WT	B16	MMC	40	14.39	1
G1529R	B16	MMC	60	28.72	1
G1529R	B16	MMC	60	26.57	2
WT	B16	MMC	60	10.00	1

Variant[a]	Batch ID[b]	Drug[c]	Concentration[d]	Percent Survived[e]	Clone ID[f]
G1529R	B16	MMC	80	15.48	1
G1529R	B16	MMC	80	11.57	2
WT	B16	MMC	80	7.32	1
G1529R	B16	Cisp	0.2	85.17	1
G1529R	B16	Cisp	0.2	90.61	2
WT	B16	Cisp	0.2	74.97	1
G1529R	B16	Cisp	0.4	43.33	1
G1529R	B16	Cisp	0.4	57.96	2
WT	B16	Cisp	0.4	18.27	1
G1529R	B16	Cisp	0.6	6.03	1
G1529R	B16	Cisp	0.6	6.97	2
WT	B16	Cisp	0.6	9.47	1
G1529R	B16	Cisp	1	17.74	1
G1529R	B16	Cisp	1	27.74	2
WT	B16	Cisp	1	12.58	1
G1529R	B16	Cisp	1.2	6.43	1
G1529R	B16	Cisp	1.2	6.90	2
WT	B16	Cisp	1.2	11.10	1
G1529R	B16	Cisp	1.5	5.96	1
G1529R	B16	Cisp	1.5	6.53	2
WT	B16	Cisp	1.5	12.04	1
G1529R	B16	MMS	5	94.80	1
G1529R	B16	MMS	5	99.77	2
WT	B16	MMS	5	76.72	1
G1529R	B16	MMS	10	88.43	1
G1529R	B16	MMS	10	99.31	2
WT	B16	MMS	10	63.60	1
G1529R	B16	MMS	15	72.43	1
G1529R	B16	MMS	15	84.58	2
WT	B16	MMS	15	45.83	1
G1529R	B16	MMS	20	50.78	1
G1529R	B16	MMS	20	69.92	2
WT	B16	MMS	20	23.65	1
G1529R	B16	MMS	30	9.75	1
G1529R	B16	MMS	30	13.51	2
WT	B16	MMS	30	5.15	1
G1529R	B16	MMS	40	2.10	1
G1529R	B16	MMS	40	1.67	2
WT	B16	MMS	40	3.06	1
G1529R	B16	Parp	0.01	78.99	1

Variant[a]	Batch ID[b]	Drug[c]	Concentration[d]	Percent Survived[e]	Clone ID[f]
G1529R	B16	Parp	0.01	62.49	2
WT	B16	Parp	0.01	88.13	1
G1529R	B16	Parp	0.1	62.82	1
G1529R	B16	Parp	0.1	37.61	2
WT	B16	Parp	0.1	59.61	1
G1529R	B16	Parp	1	38.12	1
G1529R	B16	Parp	1	9.77	2
WT	B16	Parp	1	38.13	1
G1529R	B16	Parp	10	2.52	1
G1529R	B16	Parp	10	1.37	2
WT	B16	Parp	10	2.10	1
G1529R	B16	IR	50	92.30	1
G1529R	B16	IR	50	91.90	2
WT	B16	IR	50	93.55	1
G1529R	B16	IR	100	85.24	1
G1529R	B16	IR	100	75.13	2
WT	B16	IR	100	78.21	1
G1529R	B16	IR	200	70.30	1
G1529R	B16	IR	200	86.16	2
WT	B16	IR	200	84.45	1
G1529R	B16	IR	400	54.20	1
G1529R	B16	IR	400	57.78	2
WT	B16	IR	400	62.30	1
G1529R	B16	IR	600	39.25	1
G1529R	B16	IR	600	42.58	2
WT	B16	IR	600	44.77	1
F1524V	B17	Camp	2.5	100.00	1
F1524V	B17	Camp	2.5	100.00	2
WT	B17	Camp	2.5	79.94	1
F1524V	B17	Camp	5	100.00	1
F1524V	B17	Camp	5	90.37	2
WT	B17	Camp	5	71.78	1
F1524V	B17	Camp	25	100.00	1
F1524V	B17	Camp	25	82.20	2
WT	B17	Camp	25	77.32	1
F1524V	B17	Camp	50	75.89	1
F1524V	B17	Camp	50	66.25	2
WT	B17	Camp	50	70.54	1
F1524V	B17	Camp	100	28.68	1
F1524V	B17	Camp	100	36.28	2

Variant[a]	Batch ID[b]	Drug[c]	Concentration[d]	Percent Survived[e]	Clone ID[f]
WT	B17	Camp	100	36.65	1
F1524V	B17	Camp	200	4.01	1
F1524V	B17	Camp	200	6.91	2
WT	B17	Camp	200	15.21	1
F1524V	B17	MMC	5	100.00	1
F1524V	B17	MMC	5	80.96	2
WT	B17	MMC	5	60.65	1
F1524V	B17	MMC	10	96.99	1
F1524V	B17	MMC	10	75.26	2
WT	B17	MMC	10	51.06	1
F1524V	B17	MMC	20	80.53	1
F1524V	B17	MMC	20	54.45	2
WT	B17	MMC	20	32.11	1
F1524V	B17	MMC	40	58.50	1
F1524V	B17	MMC	40	32.00	2
WT	B17	MMC	40	14.39	1
F1524V	B17	MMC	60	44.60	1
F1524V	B17	MMC	60	15.45	2
WT	B17	MMC	60	10.00	1
F1524V	B17	MMC	80	19.66	1
F1524V	B17	MMC	80	7.00	2
WT	B17	MMC	80	7.32	1
F1524V	B17	Cisp	0.2	94.90	1
F1524V	B17	Cisp	0.2	77.70	2
WT	B17	Cisp	0.2	74.97	1
F1524V	B17	Cisp	0.4	62.02	1
F1524V	B17	Cisp	0.4	24.47	2
WT	B17	Cisp	0.4	18.27	1
F1524V	B17	Cisp	0.6	12.64	1
F1524V	B17	Cisp	0.6	8.03	2
WT	B17	Cisp	0.6	9.47	1
F1524V	B17	Cisp	1	38.87	1
F1524V	B17	Cisp	1	12.31	2
WT	B17	Cisp	1	12.58	1
F1524V	B17	Cisp	1.2	9.44	1
F1524V	B17	Cisp	1.2	6.98	2
WT	B17	Cisp	1.2	11.10	1
F1524V	B17	Cisp	1.5	8.96	1
F1524V	B17	Cisp	1.5	7.96	2
WT	B17	Cisp	1.5	12.04	1

Variant[a]	Batch ID[b]	Drug[c]	Concentration[d]	Percent Survived[e]	Clone ID[f]
F1524V	B17	MMS	5	100.00	1
F1524V	B17	MMS	5	84.73	2
WT	B17	MMS	5	76.72	1
F1524V	B17	MMS	10	86.93	1
F1524V	B17	MMS	10	74.09	2
WT	B17	MMS	10	63.60	1
F1524V	B17	MMS	15	60.79	1
F1524V	B17	MMS	15	57.45	2
WT	B17	MMS	15	45.83	1
F1524V	B17	MMS	20	34.43	1
F1524V	B17	MMS	20	38.60	2
WT	B17	MMS	20	23.65	1
F1524V	B17	MMS	30	2.50	1
F1524V	B17	MMS	30	8.66	2
WT	B17	MMS	30	5.15	1
F1524V	B17	MMS	40	1.00	1
F1524V	B17	MMS	40	1.90	2
WT	B17	MMS	40	3.06	1
F1524V	B17	Parp	0.01	48.75	1
F1524V	B17	Parp	0.01	85.16	2
WT	B17	Parp	0.01	88.13	1
F1524V	B17	Parp	0.1	11.44	1
F1524V	B17	Parp	0.1	62.64	2
WT	B17	Parp	0.1	59.61	1
F1524V	B17	Parp	1	1.39	1
F1524V	B17	Parp	1	27.93	2
WT	B17	Parp	1	38.13	1
F1524V	B17	Parp	10	1.00	1
F1524V	B17	Parp	10	1.75	2
WT	B17	Parp	10	2.10	1
F1524V	B17	IR	50	78.60	1
F1524V	B17	IR	50	88.17	2
WT	B17	IR	50	93.55	1
F1524V	B17	IR	100	75.99	1
F1524V	B17	IR	100	64.93	2
WT	B17	IR	100	78.21	1
F1524V	B17	IR	200	65.76	1
F1524V	B17	IR	200	73.11	2
WT	B17	IR	200	84.45	1
F1524V	B17	IR	400	51.13	1

Variant[a]	Batch ID[b]	Drug[c]	Concentration[d]	Percent Survived[e]	Clone ID[f]
F1524V	B17	IR	400	47.34	2
WT	B17	IR	400	62.30	1
F1524V	B17	IR	600	30.36	1
F1524V	B17	IR	600	33.64	2
WT	B17	IR	600	44.77	1
R2336L	B18	Camp	2.5	98.05	1
R2336L	B18	Camp	2.5	76.80	2
WT	B18	Camp	2.5	78.21	1
R2336L	B18	Camp	5	64.45	1
R2336L	B18	Camp	5	30.09	2
WT	B18	Camp	5	45.76	1
R2336L	B18	Camp	25	47.53	1
R2336L	B18	Camp	25	22.73	2
WT	B18	Camp	25	43.43	1
R2336L	B18	Camp	50	23.96	1
R2336L	B18	Camp	50	15.05	2
WT	B18	Camp	50	29.24	1
R2336L	B18	Camp	100	13.80	1
R2336L	B18	Camp	100	12.38	2
WT	B18	Camp	100	14.13	1
R2336L	B18	Camp	200	9.38	1
R2336L	B18	Camp	200	10.19	2
WT	B18	Camp	200	4.45	1
R2336L	B18	MMC	5	70.68	1
R2336L	B18	MMC	5	59.40	2
WT	B18	MMC	5	84.14	1
R2336L	B18	MMC	10	43.15	1
R2336L	B18	MMC	10	26.06	2
WT	B18	MMC	10	76.52	1
R2336L	B18	MMC	20	24.11	1
R2336L	B18	MMC	20	13.48	2
WT	B18	MMC	20	66.53	1
R2336L	B18	MMC	40	11.92	1
R2336L	B18	MMC	40	11.88	2
WT	B18	MMC	40	46.84	1
R2336L	B18	MMC	60	7.26	1
R2336L	B18	MMC	60	8.69	2
WT	B18	MMC	60	30.36	1
R2336L	B18	MMC	80	9.32	1
R2336L	B18	MMC	80	9.75	2

Variant[a]	Batch ID[b]	Drug[c]	Concentration[d]	Percent Survived[e]	Clone ID[f]
WT	B18	MMC	80	19.07	1
R2336L	B18	Cisp	0.2	61.67	2
WT	B18	Cisp	0.2	79.51	1
R2336L	B18	Cisp	0.4	32.27	2
WT	B18	Cisp	0.4	68.21	1
R2336L	B18	Cisp	0.6	19.17	2
WT	B18	Cisp	0.6	48.69	1
R2336L	B18	Cisp	1	11.02	2
WT	B18	Cisp	1	29.47	1
R2336L	B18	Cisp	1.2	8.54	2
WT	B18	Cisp	1.2	17.28	1
R2336L	B18	Cisp	1.5	5.96	2
WT	B18	Cisp	1.5	15.48	1
R2336L	B18	Cisp	0.2	24.25	1
WT	B18	Cisp	0.2	65.69	1
R2336L	B18	Cisp	0.4	13.58	1
WT	B18	Cisp	0.4	27.71	1
R2336L	B18	Cisp	0.6	13.74	1
WT	B18	Cisp	0.6	19.51	1
R2336L	B18	Cisp	1	7.45	1
WT	B18	Cisp	1	7.40	1
R2336L	B18	Cisp	1.2	5.13	1
WT	B18	Cisp	1.2	6.73	1
R2336L	B18	Cisp	1.5	7.20	1
WT	B18	Cisp	1.5	6.67	1
R2336L	B18	MMS	5	71.00	1
R2336L	B18	MMS	5	63.42	2
WT	B18	MMS	5	75.79	1
R2336L	B18	MMS	10	40.08	1
R2336L	B18	MMS	10	25.56	2
WT	B18	MMS	10	56.59	1
R2336L	B18	MMS	15	20.38	1
R2336L	B18	MMS	15	11.02	2
WT	B18	MMS	15	45.40	1
R2336L	B18	MMS	20	11.49	1
R2336L	B18	MMS	20	7.51	2
WT	B18	MMS	20	29.64	1
R2336L	B18	MMS	30	5.06	1
R2336L	B18	MMS	30	3.35	2
WT	B18	MMS	30	12.64	1

Variant[a]	Batch ID[b]	Drug[c]	Concentration[d]	Percent Survived[e]	Clone ID[f]
R2336L	B18	MMS	40	4.10	1
R2336L	B18	MMS	40	3.35	2
WT	B18	MMS	40	4.46	1
R2336L	B18	Parp	0.01	81.12	1
R2336L	B18	Parp	0.01	55.35	2
WT	B18	Parp	0.01	88.62	1
R2336L	B18	Parp	0.1	34.31	1
R2336L	B18	Parp	0.1	20.66	2
WT	B18	Parp	0.1	78.48	1
R2336L	B18	Parp	1	13.83	1
R2336L	B18	Parp	1	12.37	2
WT	B18	Parp	1	64.12	1
R2336L	B18	Parp	10	8.38	1
R2336L	B18	Parp	10	10.11	2
WT	B18	Parp	10	6.05	1
R2336L	B18	IR	50	97.45	1
R2336L	B18	IR	50	93.56	2
WT	B18	IR	50	92.35	1
R2336L	B18	IR	100	94.09	1
R2336L	B18	IR	100	97.38	2
WT	B18	IR	100	97.06	1
R2336L	B18	IR	200	64.30	1
R2336L	B18	IR	200	62.37	2
WT	B18	IR	200	83.43	1
R2336L	B18	IR	400	23.36	1
R2336L	B18	IR	400	38.43	2
WT	B18	IR	400	12.29	1
R2336L	B18	IR	600	26.98	1
R2336L	B18	IR	600	35.81	2
WT	B18	IR	600	32.17	1
D2312V	B19	Camp	2.5	100.00	1
D2312V	B19	Camp	2.5	100.00	2
WT	B19	Camp	2.5	91.96	1
D2312V	B19	Camp	5	100.00	1
D2312V	B19	Camp	5	98.69	2
WT	B19	Camp	5	87.83	1
D2312V	B19	Camp	25	75.32	1
D2312V	B19	Camp	25	83.63	2
WT	B19	Camp	25	65.09	1
D2312V	B19	Camp	50	42.23	1

Variant[a]	Batch ID[b]	Drug[c]	Concentration[d]	Percent Survived[e]	Clone ID[f]
D2312V	B19	Camp	50	40.60	2
WT	B19	Camp	50	40.00	1
D2312V	B19	Camp	100	11.30	1
D2312V	B19	Camp	100	13.33	2
WT	B19	Camp	100	10.00	1
D2312V	B19	Camp	200	4.79	1
D2312V	B19	Camp	200	3.10	2
WT	B19	Camp	200	2.83	1
D2312V	B19	MMC	5	81.14	1
D2312V	B19	MMC	5	72.18	2
WT	B19	MMC	5	81.12	1
D2312V	B19	MMC	10	58.73	1
D2312V	B19	MMC	10	62.78	2
WT	B19	MMC	10	69.00	1
D2312V	B19	MMC	20	42.78	1
D2312V	B19	MMC	20	37.98	2
WT	B19	MMC	20	53.11	1
D2312V	B19	MMC	40	23.61	1
D2312V	B19	MMC	40	17.48	2
WT	B19	MMC	40	30.51	1
D2312V	B19	MMC	60	12.72	1
D2312V	B19	MMC	60	9.23	2
WT	B19	MMC	60	13.09	1
D2312V	B19	MMC	80	6.65	1
D2312V	B19	MMC	80	6.04	2
WT	B19	MMC	80	9.32	1
D2312V	B19	Cisp	0.2	88.09	1
D2312V	B19	Cisp	0.2	93.65	2
WT	B19	Cisp	0.2	100.00	1
D2312V	B19	Cisp	0.4	80.91	1
D2312V	B19	Cisp	0.4	78.52	2
WT	B19	Cisp	0.4	89.06	1
D2312V	B19	Cisp	0.6	76.56	1
D2312V	B19	Cisp	0.6	73.27	2
WT	B19	Cisp	0.6	81.83	1
D2312V	B19	Cisp	1	52.85	1
D2312V	B19	Cisp	1	56.49	2
WT	B19	Cisp	1	67.63	1
D2312V	B19	Cisp	1.2	43.18	1
D2312V	B19	Cisp	1.2	50.41	2

Variant[a]	Batch ID[b]	Drug[c]	Concentration[d]	Percent Survived[e]	Clone ID[f]
WT	B19	Cisp	1.2	60.96	1
D2312V	B19	Cisp	1.5	31.05	1
D2312V	B19	Cisp	1.5	37.93	2
WT	B19	Cisp	1.5	45.14	1
D2312V	B19	MMS	5	100.00	1
D2312V	B19	MMS	5	95.95	2
WT	B19	MMS	5	92.00	1
D2312V	B19	MMS	10	96.37	1
D2312V	B19	MMS	10	84.61	2
WT	B19	MMS	10	82.64	1
D2312V	B19	MMS	15	74.83	1
D2312V	B19	MMS	15	65.86	2
WT	B19	MMS	15	79.85	1
D2312V	B19	MMS	20	43.33	1
D2312V	B19	MMS	20	38.25	2
WT	B19	MMS	20	54.64	1
D2312V	B19	MMS	30	11.27	1
D2312V	B19	MMS	30	12.87	2
WT	B19	MMS	30	18.88	1
D2312V	B19	MMS	40	2.76	1
D2312V	B19	MMS	40	4.11	2
WT	B19	MMS	40	5.10	1
D2312V	B19	Parp	0.01	95.79	1
D2312V	B19	Parp	0.01	93.37	2
WT	B19	Parp	0.01	90.18	1
D2312V	B19	Parp	0.1	81.51	1
D2312V	B19	Parp	0.1	78.11	2
WT	B19	Parp	0.1	83.20	1
D2312V	B19	Parp	1	40.31	1
D2312V	B19	Parp	1	45.74	2
WT	B19	Parp	1	62.12	1
D2312V	B19	Parp	10	3.62	1
D2312V	B19	Parp	10	3.80	2
WT	B19	Parp	10	6.08	1
D2312V	B19	IR	50	68.32	1
D2312V	B19	IR	50	75.42	2
WT	B19	IR	50	94.32	1
D2312V	B19	IR	100	49.22	1
D2312V	B19	IR	100	49.52	2
WT	B19	IR	100	84.33	1

Variant[a]	Batch ID[b]	Drug[c]	Concentration[d]	Percent Survived[e]	Clone ID[f]
D2312V	B19	IR	200	25.32	1
D2312V	B19	IR	200	24.28	2
WT	B19	IR	200	74.34	1
D2312V	B19	IR	400	13.44	1
D2312V	B19	IR	400	5.85	2
WT	B19	IR	400	49.53	1
D2312V	B19	IR	600	14.43	1
D2312V	B19	IR	600	4.24	2
WT	B19	IR	600	39.75	1
G637G	B20	Camp	2.5	96.72	1
G637G	B20	Camp	2.5	98.50	2
WT	B20	Camp	2.5	94.31	1
G637G	B20	Camp	5	83.76	1
G637G	B20	Camp	5	87.38	2
WT	B20	Camp	5	87.37	1
G637G	B20	Camp	25	74.89	1
G637G	B20	Camp	25	79.43	2
WT	B20	Camp	25	80.39	1
G637G	B20	Camp	50	57.09	1
G637G	B20	Camp	50	61.92	2
WT	B20	Camp	50	58.51	1
G637G	B20	Camp	100	22.44	1
G637G	B20	Camp	100	22.99	2
WT	B20	Camp	100	20.43	1
G637G	B20	Camp	200	8.52	1
G637G	B20	Camp	200	12.84	2
WT	B20	Camp	200	7.13	1
G637G	B20	MMC	5	85.86	1
G637G	B20	MMC	5	89.31	2
WT	B20	MMC	5	93.69	1
G637G	B20	MMC	10	77.01	1
G637G	B20	MMC	10	78.15	2
WT	B20	MMC	10	81.78	1
G637G	B20	MMC	20	65.52	1
G637G	B20	MMC	20	62.43	2
WT	B20	MMC	20	67.97	1
G637G	B20	MMC	40	37.96	1
G637G	B20	MMC	40	39.40	2
WT	B20	MMC	40	42.99	1
G637G	B20	MMC	60	24.33	1

Variant[a]	Batch ID[b]	Drug[c]	Concentration[d]	Percent Survived[e]	Clone ID[f]
G637G	B20	MMC	60	25.64	2
WT	B20	MMC	60	24.58	1
G637G	B20	MMC	80	16.95	1
G637G	B20	MMC	80	20.25	2
WT	B20	MMC	80	16.24	1
G637G	B20	Cisp	0.2	64.04	1
G637G	B20	Cisp	0.2	65.93	2
WT	B20	Cisp	0.2	70.03	1
G637G	B20	Cisp	0.4	42.77	1
G637G	B20	Cisp	0.4	41.77	2
WT	B20	Cisp	0.4	48.72	1
G637G	B20	Cisp	0.6	26.87	1
G637G	B20	Cisp	0.6	27.21	2
WT	B20	Cisp	0.6	31.13	1
G637G	B20	Cisp	1	15.85	1
G637G	B20	Cisp	1	16.86	2
WT	B20	Cisp	1	15.08	1
G637G	B20	Cisp	1.2	12.23	1
G637G	B20	Cisp	1.2	13.06	2
WT	B20	Cisp	1.2	9.68	1
G637G	B20	Cisp	1.5	10.92	1
G637G	B20	Cisp	1.5	13.86	2
WT	B20	Cisp	1.5	7.26	1
G637G	B20	MMS	5	97.42	1
G637G	B20	MMS	5	86.00	2
WT	B20	MMS	5	93.06	1
G637G	B20	MMS	10	80.93	1
G637G	B20	MMS	10	73.26	2
WT	B20	MMS	10	82.92	1
G637G	B20	MMS	15	70.30	1
G637G	B20	MMS	15	61.36	2
WT	B20	MMS	15	74.69	1
G637G	B20	MMS	20	40.33	1
G637G	B20	MMS	20	40.09	2
WT	B20	MMS	20	56.13	1
G637G	B20	MMS	30	11.68	1
G637G	B20	MMS	30	16.37	2
WT	B20	MMS	30	16.17	1
G637G	B20	MMS	40	4.94	1
G637G	B20	MMS	40	6.39	2

Variant[a]	Batch ID[b]	Drug[c]	Concentration[d]	Percent Survived[e]	Clone ID[f]
WT	B20	MMS	40	4.59	1
G637G	B20	Parp	0.01	92.39	1
G637G	B20	Parp	0.01	82.45	2
WT	B20	Parp	0.01	86.37	1
G637G	B20	Parp	0.1	68.35	1
G637G	B20	Parp	0.1	61.08	2
WT	B20	Parp	0.1	71.32	1
G637G	B20	Parp	1	34.05	1
G637G	B20	Parp	1	31.28	2
WT	B20	Parp	1	50.27	1
G637G	B20	Parp	10	3.62	1
G637G	B20	Parp	10	4.69	2
WT	B20	Parp	10	2.08	1
G637G	B20	IR	50	89.30	1
G637G	B20	IR	50	94.35	2
WT	B20	IR	50	83.44	1
G637G	B20	IR	100	97.06	1
G637G	B20	IR	100	90.99	2
WT	B20	IR	100	81.37	1
G637G	B20	IR	200	77.66	1
G637G	B20	IR	200	60.76	2
WT	B20	IR	200	67.93	1
G637G	B20	IR	400	49.15	1
G637G	B20	IR	400	40.51	2
WT	B20	IR	400	46.92	1
G637G	B20	IR	600	39.09	1
G637G	B20	IR	600	33.49	2
WT	B20	IR	600	40.18	1
D2312E	B21	Camp	2.5	82.52	1
D2312E	B21	Camp	2.5	92.00	2
WT	B21	Camp	2.5	82.67	1
D2312E	B21	Camp	5	92.66	1
D2312E	B21	Camp	5	100.00	2
WT	B21	Camp	5	69.04	1
D2312E	B21	Camp	25	69.02	1
D2312E	B21	Camp	25	70.86	2
WT	B21	Camp	25	40.88	1
D2312E	B21	Camp	50	36.59	1
D2312E	B21	Camp	50	30.40	2
WT	B21	Camp	50	15.78	1

Variant[a]	Batch ID[b]	Drug[c]	Concentration[d]	Percent Survived[e]	Clone ID[f]
D2312E	B21	Camp	100	7.52	1
D2312E	B21	Camp	100	4.32	2
WT	B21	Camp	100	4.24	1
D2312E	B21	Camp	200	2.54	1
D2312E	B21	Camp	200	2.43	2
WT	B21	Camp	200	4.54	1
D2312E	B21	MMC	5	77.87	1
D2312E	B21	MMC	5	72.49	2
WT	B21	MMC	5	80.79	1
D2312E	B21	MMC	10	75.46	1
D2312E	B21	MMC	10	65.57	2
WT	B21	MMC	10	79.71	1
D2312E	B21	MMC	20	57.87	1
D2312E	B21	MMC	20	60.05	2
WT	B21	MMC	20	83.47	1
D2312E	B21	MMC	40	33.43	1
D2312E	B21	MMC	40	28.38	2
WT	B21	MMC	40	49.97	1
D2312E	B21	MMC	60	11.21	1
D2312E	B21	MMC	60	10.49	2
WT	B21	MMC	60	31.01	1
D2312E	B21	MMC	80	5.12	1
D2312E	B21	MMC	80	5.38	2
WT	B21	MMC	80	18.06	1
D2312E	B21	Cisp	0.2	66.03	1
D2312E	B21	Cisp	0.2	69.08	2
WT	B21	Cisp	0.2	74.12	1
D2312E	B21	Cisp	0.4	37.93	1
D2312E	B21	Cisp	0.4	38.56	2
WT	B21	Cisp	0.4	52.11	1
D2312E	B21	Cisp	0.6	24.20	1
D2312E	B21	Cisp	0.6	32.73	2
WT	B21	Cisp	0.6	27.89	1
D2312E	B21	Cisp	1	6.65	1
D2312E	B21	Cisp	1	7.71	2
WT	B21	Cisp	1	7.77	1
D2312E	B21	Cisp	1.2	4.03	1
D2312E	B21	Cisp	1.2	4.76	2
WT	B21	Cisp	1.2	5.64	1
D2312E	B21	Cisp	1.5	4.25	1

Variant[a]	Batch ID[b]	Drug[c]	Concentration[d]	Percent Survived[e]	Clone ID[f]
D2312E	B21	Cisp	1.5	1.61	2
WT	B21	Cisp	1.5	6.59	1
D2312E	B21	MMS	5	93.77	1
D2312E	B21	MMS	5	100.00	2
WT	B21	MMS	5	86.35	1
D2312E	B21	MMS	10	83.84	1
D2312E	B21	MMS	10	100.00	2
WT	B21	MMS	10	65.04	1
D2312E	B21	MMS	15	59.59	1
D2312E	B21	MMS	15	83.89	2
WT	B21	MMS	15	38.02	1
D2312E	B21	MMS	20	36.81	1
D2312E	B21	MMS	20	65.95	2
WT	B21	MMS	20	20.03	1
D2312E	B21	MMS	30	4.97	1
D2312E	B21	MMS	30	6.98	2
WT	B21	MMS	30	7.33	1
D2312E	B21	MMS	40	2.04	1
D2312E	B21	MMS	40	1.83	2
WT	B21	MMS	40	5.23	1
D2312E	B21	Parp	0.01	71.28	1
D2312E	B21	Parp	0.01	78.33	2
WT	B21	Parp	0.01	73.84	1
D2312E	B21	Parp	0.1	67.96	1
D2312E	B21	Parp	0.1	61.27	2
WT	B21	Parp	0.1	72.77	1
D2312E	B21	Parp	1	30.14	1
D2312E	B21	Parp	1	37.79	2
WT	B21	Parp	1	40.38	1
D2312E	B21	Parp	10	1.00	1
D2312E	B21	Parp	10	1.00	2
WT	B21	Parp	10	2.58	1
D2312E	B21	IR	50	100.00	1
D2312E	B21	IR	50	100.00	2
WT	B21	IR	50	100.00	1
D2312E	B21	IR	100	100.00	1
D2312E	B21	IR	100	87.04	2
WT	B21	IR	100	87.64	1
D2312E	B21	IR	200	78.34	1
D2312E	B21	IR	200	69.68	2

Variant[a]	Batch ID[b]	Drug[c]	Concentration[d]	Percent Survived[e]	Clone ID[f]
WT	B21	IR	200	94.93	1
D2312E	B21	IR	400	46.63	1
D2312E	B21	IR	400	48.26	2
WT	B21	IR	400	47.70	1
D2312E	B21	IR	600	28.68	1
D2312E	B21	IR	600	27.33	2
WT	B21	IR	600	32.09	1
T2708N*	B22	Camp	2.5	75.99	1
T2708N*	B22	Camp	2.5	100.00	2
WT	B22	Camp	2.5	90.67	1
T2708N*	B22	Camp	5	57.57	1
T2708N*	B22	Camp	5	86.75	2
WT	B22	Camp	5	87.86	1
T2708N*	B22	Camp	25	14.32	1
T2708N*	B22	Camp	25	23.05	2
WT	B22	Camp	25	67.12	1
T2708N*	B22	Camp	50	15.96	1
T2708N*	B22	Camp	50	3.79	2
WT	B22	Camp	50	50.72	1
T2708N*	B22	Camp	100	15.01	1
T2708N*	B22	Camp	100	1.78	2
WT	B22	Camp	100	8.65	1
T2708N*	B22	Camp	200	16.64	1
T2708N*	B22	Camp	200	2.45	2
WT	B22	Camp	200	4.98	1
T2708N*	B22	MMC	5	30.42	1
T2708N*	B22	MMC	5	62.36	2
WT	B22	MMC	5	94.39	1
T2708N*	B22	MMC	10	17.58	1
T2708N*	B22	MMC	10	26.09	2
WT	B22	MMC	10	78.61	1
T2708N*	B22	MMC	20	14.21	1
T2708N*	B22	MMC	20	9.89	2
WT	B22	MMC	20	70.14	1
T2708N*	B22	MMC	40	10.02	1
T2708N*	B22	MMC	40	3.33	2
WT	B22	MMC	40	44.23	1
T2708N*	B22	MMC	60	14.21	1
T2708N*	B22	MMC	60	2.15	2
WT	B22	MMC	60	20.47	1

Variant[a]	Batch ID[b]	Drug[c]	Concentration[d]	Percent Survived[e]	Clone ID[f]
T2708N*	B22	MMC	80	13.57	1
T2708N*	B22	MMC	80	3.28	2
WT	B22	MMC	80	11.18	1
T2708N*	B22	Cisp	0.2	15.49	1
T2708N*	B22	Cisp	0.2	14.99	2
WT	B22	Cisp	0.2	71.23	1
T2708N*	B22	Cisp	0.4	13.71	1
T2708N*	B22	Cisp	0.4	4.67	2
WT	B22	Cisp	0.4	37.93	1
T2708N*	B22	Cisp	0.6	12.51	1
T2708N*	B22	Cisp	0.6	2.92	2
WT	B22	Cisp	0.6	20.34	1
T2708N*	B22	Cisp	1	12.99	1
T2708N*	B22	Cisp	1	2.34	2
WT	B22	Cisp	1	7.28	1
T2708N*	B22	Cisp	1.2	10.73	1
T2708N*	B22	Cisp	1.2	3.02	2
WT	B22	Cisp	1.2	5.98	1
T2708N*	B22	Cisp	1.5	17.64	1
T2708N*	B22	Cisp	1.5	3.55	2
WT	B22	Cisp	1.5	5.83	1
T2708N*	B22	MMS	5	26.09	1
T2708N*	B22	MMS	5	65.67	2
WT	B22	MMS	5	95.29	1
T2708N*	B22	MMS	10	13.13	1
T2708N*	B22	MMS	10	30.32	2
WT	B22	MMS	10	86.79	1
T2708N*	B22	MMS	15	11.50	1
T2708N*	B22	MMS	15	13.62	2
WT	B22	MMS	15	75.46	1
T2708N*	B22	MMS	20	8.84	1
T2708N*	B22	MMS	20	3.44	2
WT	B22	MMS	20	55.10	1
T2708N*	B22	MMS	30	8.24	1
T2708N*	B22	MMS	30	2.29	2
WT	B22	MMS	30	16.86	1
T2708N*	B22	MMS	40	7.64	1
T2708N*	B22	MMS	40	1.32	2
WT	B22	MMS	40	3.74	1
T2708N*	B22	Parp	0.01	16.21	1

Variant[a]	Batch ID[b]	Drug[c]	Concentration[d]	Percent Survived[e]	Clone ID[f]
T2708N*	B22	Parp	0.01	52.29	2
WT	B22	Parp	0.01	86.56	1
T2708N*	B22	Parp	0.1	14.32	1
T2708N*	B22	Parp	0.1	14.76	2
WT	B22	Parp	0.1	77.74	1
T2708N*	B22	Parp	1	12.26	1
T2708N*	B22	Parp	1	5.02	2
WT	B22	Parp	1	52.91	1
T2708N*	B22	Parp	10	10.95	1
T2708N*	B22	Parp	10	2.94	2
WT	B22	Parp	10	4.82	1
T2708N*	B22	IR	50	68.32	1
T2708N*	B22	IR	50	75.42	2
WT	B22	IR	50	94.32	1
T2708N*	B22	IR	200	25.32	1
T2708N*	B22	IR	200	24.28	2
WT	B22	IR	200	74.34	1
T2708N*	B22	IR	400	13.44	1
T2708N*	B22	IR	400	5.85	2
WT	B22	IR	400	49.53	1
T2708N*	B22	IR	600	14.43	1
T2708N*	B22	IR	600	4.24	2
WT	B22	IR	600	39.75	1
G2281V	B23	Camp	2.5	93.34	1
G2281V	B23	Camp	2.5	100.00	2
WT	B23	Camp	2.5	90.67	1
G2281V	B23	Camp	5	78.13	1
G2281V	B23	Camp	5	100.00	2
WT	B23	Camp	5	87.86	1
G2281V	B23	Camp	25	83.78	1
G2281V	B23	Camp	25	75.29	2
WT	B23	Camp	25	67.12	1
G2281V	B23	Camp	50	34.02	1
G2281V	B23	Camp	50	39.17	2
WT	B23	Camp	50	50.72	1
G2281V	B23	Camp	100	4.86	1
G2281V	B23	Camp	100	4.33	2
WT	B23	Camp	100	8.65	1
G2281V	B23	Camp	200	2.69	1
G2281V	B23	Camp	200	2.63	2

Variant[a]	Batch ID[b]	Drug[c]	Concentration[d]	Percent Survived[e]	Clone ID[f]
WT	B23	Camp	200	4.98	1
G2281V	B23	MMC	5	96.93	1
G2281V	B23	MMC	5	87.64	2
WT	B23	MMC	5	94.39	1
G2281V	B23	MMC	10	69.12	1
G2281V	B23	MMC	10	78.58	2
WT	B23	MMC	10	78.61	1
G2281V	B23	MMC	20	61.19	1
G2281V	B23	MMC	20	55.51	2
WT	B23	MMC	20	70.14	1
G2281V	B23	MMC	40	27.48	1
G2281V	B23	MMC	40	28.40	2
WT	B23	MMC	40	44.23	1
G2281V	B23	MMC	60	10.58	1
G2281V	B23	MMC	60	11.29	2
WT	B23	MMC	60	20.47	1
G2281V	B23	MMC	80	5.52	1
G2281V	B23	MMC	80	5.91	2
WT	B23	MMC	80	11.18	1
G2281V	B23	Cisp	0.2	56.38	1
G2281V	B23	Cisp	0.2	56.24	2
WT	B23	Cisp	0.2	71.23	1
G2281V	B23	Cisp	0.4	20.04	1
G2281V	B23	Cisp	0.4	23.57	2
WT	B23	Cisp	0.4	37.93	1
G2281V	B23	Cisp	0.6	9.20	1
G2281V	B23	Cisp	0.6	11.27	2
WT	B23	Cisp	0.6	20.34	1
G2281V	B23	Cisp	1	3.55	1
G2281V	B23	Cisp	1	8.18	2
WT	B23	Cisp	1	7.28	1
G2281V	B23	Cisp	1.2	4.03	1
G2281V	B23	Cisp	1.2	5.61	2
WT	B23	Cisp	1.2	5.98	1
G2281V	B23	Cisp	1.5	3.26	1
G2281V	B23	Cisp	1.5	6.06	2
WT	B23	Cisp	1.5	5.83	1
G2281V	B23	MMS	5	100.00	1
G2281V	B23	MMS	5	94.32	2
WT	B23	MMS	5	95.29	1

Variant[a]	Batch ID[b]	Drug[c]	Concentration[d]	Percent Survived[e]	Clone ID[f]
G2281V	B23	MMS	10	80.76	1
G2281V	B23	MMS	10	83.29	2
WT	B23	MMS	10	86.79	1
G2281V	B23	MMS	15	51.20	1
G2281V	B23	MMS	15	61.17	2
WT	B23	MMS	15	75.46	1
G2281V	B23	MMS	20	18.75	1
G2281V	B23	MMS	20	24.34	2
WT	B23	MMS	20	55.10	1
G2281V	B23	MMS	30	3.77	1
G2281V	B23	MMS	30	5.19	2
WT	B23	MMS	30	16.86	1
G2281V	B23	MMS	40	1.73	1
G2281V	B23	MMS	40	2.60	2
WT	B23	MMS	40	3.74	1
G2281V	B23	Parp	0.01	100.00	1
G2281V	B23	Parp	0.01	89.09	2
WT	B23	Parp	0.01	86.56	1
G2281V	B23	Parp	0.1	70.89	1
G2281V	B23	Parp	0.1	79.94	2
WT	B23	Parp	0.1	77.74	1
G2281V	B23	Parp	1	39.28	1
G2281V	B23	Parp	1	35.43	2
WT	B23	Parp	1	52.91	1
G2281V	B23	Parp	10	2.82	1
G2281V	B23	Parp	10	3.25	2
WT	B23	Parp	10	4.82	1
G2281V	B23	IR	50	84.32	1
G2281V	B23	IR	50	84.28	2
WT	B23	IR	50	94.32	1
G2281V	B23	IR	200	54.35	1
G2281V	B23	IR	200	65.34	2
WT	B23	IR	200	74.34	1
G2281V	B23	IR	400	23.53	1
G2281V	B23	IR	400	37.35	2
WT	B23	IR	400	49.53	1
G2281V	B23	IR	600	17.57	1
G2281V	B23	IR	600	26.01	2
WT	B23	IR	600	39.75	1
N2622S	B24	Camp	2.5	100.00	1

Variant[a]	Batch ID[b]	Drug[c]	Concentration[d]	Percent Survived[e]	Clone ID[f]
N2622S	B24	Camp	2.5	93.72	2
WT	B24	Camp	2.5	90.67	1
N2622S	B24	Camp	5	87.94	1
N2622S	B24	Camp	5	100.00	2
WT	B24	Camp	5	87.86	1
N2622S	B24	Camp	25	42.78	1
N2622S	B24	Camp	25	51.65	2
WT	B24	Camp	25	67.12	1
N2622S	B24	Camp	50	6.26	1
N2622S	B24	Camp	50	10.65	2
WT	B24	Camp	50	50.72	1
N2622S	B24	Camp	100	1.00	1
N2622S	B24	Camp	100	2.51	2
WT	B24	Camp	100	8.65	1
N2622S	B24	Camp	200	1.39	1
N2622S	B24	Camp	200	4.56	2
WT	B24	Camp	200	4.98	1
N2622S	B24	MMC	5	81.15	1
N2622S	B24	MMC	5	83.05	2
WT	B24	MMC	5	94.39	1
N2622S	B24	MMC	10	62.03	1
N2622S	B24	MMC	10	67.97	2
WT	B24	MMC	10	78.61	1
N2622S	B24	MMC	20	28.41	1
N2622S	B24	MMC	20	40.05	2
WT	B24	MMC	20	70.14	1
N2622S	B24	MMC	40	7.58	1
N2622S	B24	MMC	40	11.66	2
WT	B24	MMC	40	44.23	1
N2622S	B24	MMC	60	3.96	1
N2622S	B24	MMC	60	5.52	2
WT	B24	MMC	60	20.47	1
N2622S	B24	MMC	80	3.52	1
N2622S	B24	MMC	80	4.32	2
WT	B24	MMC	80	11.18	1
N2622S	B24	Cisp	0.2	45.15	1
N2622S	B24	Cisp	0.2	37.43	2
WT	B24	Cisp	0.2	71.23	1
N2622S	B24	Cisp	0.4	10.05	1
N2622S	B24	Cisp	0.4	12.04	2

Variant[a]	Batch ID[b]	Drug[c]	Concentration[d]	Percent Survived[e]	Clone ID[f]
WT	B24	Cisp	0.4	37.93	1
N2622S	B24	Cisp	0.6	2.22	1
N2622S	B24	Cisp	0.6	6.42	2
WT	B24	Cisp	0.6	20.34	1
N2622S	B24	Cisp	1	1.00	1
N2622S	B24	Cisp	1	2.78	2
WT	B24	Cisp	1	7.28	1
N2622S	B24	Cisp	1.2	1.00	1
N2622S	B24	Cisp	1.2	3.17	2
WT	B24	Cisp	1.2	5.98	1
N2622S	B24	Cisp	1.5	1.99	1
N2622S	B24	Cisp	1.5	3.04	2
WT	B24	Cisp	1.5	5.83	1
N2622S	B24	MMS	5	77.25	1
N2622S	B24	MMS	5	89.64	2
WT	B24	MMS	5	95.29	1
N2622S	B24	MMS	10	48.74	1
N2622S	B24	MMS	10	60.53	2
WT	B24	MMS	10	86.79	1
N2622S	B24	MMS	15	15.55	1
N2622S	B24	MMS	15	36.66	2
WT	B24	MMS	15	75.46	1
N2622S	B24	MMS	20	3.41	1
N2622S	B24	MMS	20	13.96	2
WT	B24	MMS	20	55.10	1
N2622S	B24	MMS	30	1.00	1
N2622S	B24	MMS	30	3.49	2
WT	B24	MMS	30	16.86	1
N2622S	B24	MMS	40	1.00	1
N2622S	B24	MMS	40	1.80	2
WT	B24	MMS	40	3.74	1
N2622S	B24	Parp	0.01	74.35	1
N2622S	B24	Parp	0.01	85.79	2
WT	B24	Parp	0.01	86.56	1
N2622S	B24	Parp	0.1	31.32	1
N2622S	B24	Parp	0.1	39.71	2
WT	B24	Parp	0.1	77.74	1
N2622S	B24	Parp	1	4.92	1
N2622S	B24	Parp	1	8.80	2
WT	B24	Parp	1	52.91	1

Variant[a]	Batch ID[b]	Drug[c]	Concentration[d]	Percent Survived[e]	Clone ID[f]
N2622S	B24	Parp	10	2.46	1
N2622S	B24	Parp	10	2.79	2
WT	B24	Parp	10	4.82	1
N2622S	B24	IR	50	81.13	1
N2622S	B24	IR	50	90.32	2
WT	B24	IR	50	94.32	1
N2622S	B24	IR	200	55.52	1
N2622S	B24	IR	200	59.68	2
WT	B24	IR	200	74.34	1
N2622S	B24	IR	400	29.61	1
N2622S	B24	IR	400	30.38	2
WT	B24	IR	400	49.53	1
N2622S	B24	IR	600	14.38	1
N2622S	B24	IR	600	26.08	2
WT	B24	IR	600	39.75	1
K16R	B25	Camp	2.5	86.53	1
K16R	B25	Camp	2.5	100.00	2
WT	B25	Camp	2.5	71.59	1
K16R	B25	Camp	5	92.32	1
K16R	B25	Camp	5	100.00	2
WT	B25	Camp	5	79.19	1
K16R	B25	Camp	25	83.87	1
K16R	B25	Camp	25	95.23	2
WT	B25	Camp	25	71.83	1
K16R	B25	Camp	50	64.09	1
K16R	B25	Camp	50	69.35	2
WT	B25	Camp	50	56.46	1
K16R	B25	Camp	100	15.48	1
K16R	B25	Camp	100	13.71	2
WT	B25	Camp	100	21.17	1
K16R	B25	Camp	200	4.73	1
K16R	B25	Camp	200	6.96	2
WT	B25	Camp	200	8.61	1
K16R	B25	MMC	5	82.98	1
K16R	B25	MMC	5	90.89	2
WT	B25	MMC	5	87.15	1
K16R	B25	MMC	10	84.71	1
K16R	B25	MMC	10	100.00	2
WT	B25	MMC	10	88.19	1
K16R	B25	MMC	20	79.84	1

Variant[a]	Batch ID[b]	Drug[c]	Concentration[d]	Percent Survived[e]	Clone ID[f]
K16R	B25	MMC	20	84.86	2
WT	B25	MMC	20	94.35	1
K16R	B25	MMC	40	43.25	1
K16R	B25	MMC	40	42.26	2
WT	B25	MMC	40	59.38	1
K16R	B25	MMC	60	24.45	1
K16R	B25	MMC	60	23.36	2
WT	B25	MMC	60	34.91	1
K16R	B25	MMC	80	17.70	1
K16R	B25	MMC	80	18.08	2
WT	B25	MMC	80	25.50	1
K16R	B25	Cisp	0.2	78.66	1
K16R	B25	Cisp	0.2	74.03	2
WT	B25	Cisp	0.2	49.68	1
K16R	B25	Cisp	0.4	42.90	1
K16R	B25	Cisp	0.4	43.84	2
WT	B25	Cisp	0.4	25.65	1
K16R	B25	Cisp	0.6	27.12	1
K16R	B25	Cisp	0.6	27.87	2
WT	B25	Cisp	0.6	20.32	1
K16R	B25	Cisp	1	12.47	1
K16R	B25	Cisp	1	12.41	2
WT	B25	Cisp	1	14.02	1
K16R	B25	Cisp	1.2	9.99	1
K16R	B25	Cisp	1.2	9.59	2
WT	B25	Cisp	1.2	14.37	1
K16R	B25	Cisp	1.5	10.14	1
K16R	B25	Cisp	1.5	9.10	2
WT	B25	Cisp	1.5	11.28	1
K16R	B25	MMS	5	66.04	1
K16R	B25	MMS	5	74.03	2
WT	B25	MMS	5	49.68	1
K16R	B25	MMS	10	71.82	1
K16R	B25	MMS	10	43.84	2
WT	B25	MMS	10	25.65	1
K16R	B25	MMS	15	63.44	1
K16R	B25	MMS	15	27.87	2
WT	B25	MMS	15	20.32	1
K16R	B25	MMS	20	45.84	1
K16R	B25	MMS	20	12.41	2

Variant[a]	Batch ID[b]	Drug[c]	Concentration[d]	Percent Survived[e]	Clone ID[f]
WT	B25	MMS	20	14.02	1
K16R	B25	MMS	30	11.71	1
K16R	B25	MMS	30	9.59	2
WT	B25	MMS	30	14.37	1
K16R	B25	MMS	40	4.30	1
K16R	B25	MMS	40	9.10	2
WT	B25	MMS	40	11.28	1
K16R	B25	Parp	0.01	100.00	1
K16R	B25	Parp	0.01	76.15	2
WT	B25	Parp	0.01	99.58	1
K16R	B25	Parp	0.1	75.08	1
K16R	B25	Parp	0.1	22.00	2
WT	B25	Parp	0.1	99.82	1
K16R	B25	Parp	1	45.86	1
K16R	B25	Parp	1	32.57	2
WT	B25	Parp	1	85.05	1
K16R	B25	Parp	10	1.00	1
K16R	B25	Parp	10	1.00	2
WT	B25	Parp	10	16.33	1
K16R	B25	IR	40	89.12	1
K16R	B25	IR	40	90.88	2
WT	B25	IR	40	80.46	1
K16R	B25	IR	100	67.70	1
K16R	B25	IR	100	77.92	2
WT	B25	IR	100	67.37	1
K16R	B25	IR	200	76.82	1
K16R	B25	IR	200	84.15	2
WT	B25	IR	200	57.01	1
K16R	B25	IR	400	41.20	1
K16R	B25	IR	400	45.66	2
WT	B25	IR	400	31.55	1
K16R	B25	IR	600	20.08	1
K16R	B25	IR	600	25.77	2
WT	B25	IR	600	14.85	1
K1883N	B26	Camp	2.5	76.00	1
K1883N	B26	Camp	2.5	74.00	2
WT	B26	Camp	2.5	67.00	1
K1883N	B26	Camp	5	68.00	1
K1883N	B26	Camp	5	61.00	2
WT	B26	Camp	5	64.00	1

Variant[a]	Batch ID[b]	Drug[c]	Concentration[d]	Percent Survived[e]	Clone ID[f]
K1883N	B26	Camp	25	43.00	1
K1883N	B26	Camp	25	54.00	2
WT	B26	Camp	25	41.00	1
K1883N	B26	Camp	50	28.00	1
K1883N	B26	Camp	50	39.00	2
WT	B26	Camp	50	26.00	1
K1883N	B26	Camp	100	18.00	1
K1883N	B26	Camp	100	30.00	2
WT	B26	Camp	100	16.00	1
K1883N	B26	Camp	200	19.00	1
K1883N	B26	Camp	200	14.00	2
WT	B26	Camp	200	15.00	1
K1883N	B26	MMC	5	73.00	1
K1883N	B26	MMC	5	87.00	2
WT	B26	MMC	5	71.00	1
K1883N	B26	MMC	10	54.00	1
K1883N	B26	MMC	10	73.00	2
WT	B26	MMC	10	58.00	1
K1883N	B26	MMC	20	35.00	1
K1883N	B26	MMC	20	71.00	2
WT	B26	MMC	20	41.00	1
K1883N	B26	MMC	40	21.00	1
K1883N	B26	MMC	40	51.00	2
WT	B26	MMC	40	16.00	1
K1883N	B26	MMC	60	13.00	1
K1883N	B26	MMC	60	37.00	2
WT	B26	MMC	60	10.00	1
K1883N	B26	MMC	80	13.00	1
K1883N	B26	MMC	80	23.00	2
WT	B26	MMC	80	9.00	1
K1883N	B26	Cisp	0.2	47.00	1
K1883N	B26	Cisp	0.2	81.00	2
WT	B26	Cisp	0.2	46.00	1
K1883N	B26	Cisp	0.4	26.00	1
K1883N	B26	Cisp	0.4	66.00	2
WT	B26	Cisp	0.4	20.00	1
K1883N	B26	Cisp	0.6	22.00	1
K1883N	B26	Cisp	0.6	51.00	2
WT	B26	Cisp	0.6	15.00	1
K1883N	B26	Cisp	1	16.00	1

Variant[a]	Batch ID[b]	Drug[c]	Concentration[d]	Percent Survived[e]	Clone ID[f]
K1883N	B26	Cisp	1	28.00	2
WT	B26	Cisp	1	10.00	1
K1883N	B26	Cisp	1.2	16.00	1
K1883N	B26	Cisp	1.2	28.00	2
WT	B26	Cisp	1.2	9.00	1
K1883N	B26	Cisp	1.5	17.00	1
K1883N	B26	Cisp	1.5	23.00	2
WT	B26	Cisp	1.5	11.00	1
K1883N	B26	MMS	5	54.00	1
K1883N	B26	MMS	5	65.00	2
WT	B26	MMS	5	62.00	1
K1883N	B26	MMS	10	33.00	1
K1883N	B26	MMS	10	47.00	2
WT	B26	MMS	10	32.00	1
K1883N	B26	MMS	15	18.00	1
K1883N	B26	MMS	15	37.00	2
WT	B26	MMS	15	14.00	1
K1883N	B26	MMS	20	18.00	1
K1883N	B26	MMS	20	26.00	2
WT	B26	MMS	20	8.00	1
K1883N	B26	MMS	30	12.00	1
K1883N	B26	MMS	30	13.00	2
WT	B26	MMS	30	3.00	1
K1883N	B26	MMS	40	10.00	1
K1883N	B26	MMS	40	7.00	2
WT	B26	MMS	40	6.00	1
K1883N	B26	Parp	0.01	47.00	1
K1883N	B26	Parp	0.01	69.00	2
WT	B26	Parp	0.01	46.00	1
K1883N	B26	Parp	0.1	25.00	1
K1883N	B26	Parp	0.1	53.00	2
WT	B26	Parp	0.1	21.00	1
K1883N	B26	Parp	1	23.00	1
K1883N	B26	Parp	1	39.00	2
WT	B26	Parp	1	17.00	1
K1883N	B26	Parp	10	17.00	1
K1883N	B26	Parp	10	5.00	2
WT	B26	Parp	10	8.00	1
K1883N	B26	IR	50	24.00	1
K1883N	B26	IR	50	43.00	2

Variant[a]	Batch ID[b]	Drug[c]	Concentration[d]	Percent Survived[e]	Clone ID[f]
WT	B26	IR	50	26.00	1
K1883N	B26	IR	100	21.00	1
K1883N	B26	IR	100	34.00	2
WT	B26	IR	100	25.00	1
K1883N	B26	IR	200	15.00	1
K1883N	B26	IR	200	32.00	2
WT	B26	IR	200	18.00	1
K1883N	B26	IR	400	11.00	1
K1883N	B26	IR	400	21.00	2
WT	B26	IR	400	10.00	1
K1883N	B26	IR	600	11.00	1
K1883N	B26	IR	600	22.00	2
WT	B26	IR	600	8.00	1
D1781N	B27	Camp	2.5	66.07	1
D1781N	B27	Camp	2.5	87.79	2
WT	B27	Camp	2.5	60.95	1
D1781N	B27	Camp	5	69.18	1
D1781N	B27	Camp	5	80.02	2
WT	B27	Camp	5	62.14	1
D1781N	B27	Camp	25	44.80	1
D1781N	B27	Camp	25	59.81	2
WT	B27	Camp	25	44.40	1
D1781N	B27	Camp	50	15.51	1
D1781N	B27	Camp	50	20.63	2
WT	B27	Camp	50	23.79	1
D1781N	B27	Camp	100	4.37	1
D1781N	B27	Camp	100	4.09	2
WT	B27	Camp	100	9.51	1
D1781N	B27	Camp	200	1.66	1
D1781N	B27	Camp	200	1.72	2
WT	B27	Camp	200	5.85	1
D1781N	B27	MMC	5	59.31	1
D1781N	B27	MMC	5	58.15	2
WT	B27	MMC	5	51.63	1
D1781N	B27	MMC	10	43.70	1
D1781N	B27	MMC	10	46.91	2
WT	B27	MMC	10	47.53	1
D1781N	B27	MMC	20	34.81	1
D1781N	B27	MMC	20	41.58	2
WT	B27	MMC	20	32.42	1

Variant[a]	Batch ID[b]	Drug[c]	Concentration[d]	Percent Survived[e]	Clone ID[f]
D1781N	B27	MMC	40	15.11	1
D1781N	B27	MMC	40	19.16	2
WT	B27	MMC	40	18.15	1
D1781N	B27	MMC	60	8.09	1
D1781N	B27	MMC	60	5.07	2
WT	B27	MMC	60	9.34	1
D1781N	B27	MMC	80	7.02	1
D1781N	B27	MMC	80	4.49	2
WT	B27	MMC	80	8.60	1
D1781N	B27	Cisp	0.2	51.19	1
D1781N	B27	Cisp	0.2	50.93	2
WT	B27	Cisp	0.2	48.90	1
D1781N	B27	Cisp	0.4	33.73	1
D1781N	B27	Cisp	0.4	26.06	2
WT	B27	Cisp	0.4	31.66	1
D1781N	B27	Cisp	0.6	17.68	1
D1781N	B27	Cisp	0.6	16.13	2
WT	B27	Cisp	0.6	21.88	1
D1781N	B27	Cisp	1	5.65	1
D1781N	B27	Cisp	1	9.93	2
WT	B27	Cisp	1	13.45	1
D1781N	B27	Cisp	1.2	3.94	1
D1781N	B27	Cisp	1.2	8.89	2
WT	B27	Cisp	1.2	10.02	1
D1781N	B27	Cisp	1.5	4.31	1
D1781N	B27	Cisp	1.5	8.96	2
WT	B27	Cisp	1.5	11.61	1
D1781N	B27	MMS	5	59.40	1
D1781N	B27	MMS	5	63.66	2
WT	B27	MMS	5	70.61	1
D1781N	B27	MMS	10	45.17	1
D1781N	B27	MMS	10	45.05	2
WT	B27	MMS	10	46.49	1
D1781N	B27	MMS	15	19.64	1
D1781N	B27	MMS	15	25.95	2
WT	B27	MMS	15	34.89	1
D1781N	B27	MMS	20	15.08	1
D1781N	B27	MMS	20	15.25	2
WT	B27	MMS	20	17.68	1
D1781N	B27	MMS	30	4.95	1

Variant[a]	Batch ID[b]	Drug[c]	Concentration[d]	Percent Survived[e]	Clone ID[f]
D1781N	B27	MMS	30	2.61	2
WT	B27	MMS	30	9.02	1
D1781N	B27	MMS	40	4.02	1
D1781N	B27	MMS	40	2.24	2
WT	B27	MMS	40	4.45	1
D1781N	B27	Parp	0.01	62.87	1
D1781N	B27	Parp	0.01	90.06	2
WT	B27	Parp	0.01	58.12	1
D1781N	B27	Parp	0.1	44.25	1
D1781N	B27	Parp	0.1	62.27	2
WT	B27	Parp	0.1	50.61	1
D1781N	B27	Parp	1	9.72	1
D1781N	B27	Parp	1	15.80	2
WT	B27	Parp	1	24.58	1
D1781N	B27	Parp	10	4.70	1
D1781N	B27	Parp	10	2.67	2
WT	B27	Parp	10	6.14	1
D1781N	B27	IR	50	88.89	1
D1781N	B27	IR	50	100.00	2
WT	B27	IR	50	95.51	1
D1781N	B27	IR	100	72.05	1
D1781N	B27	IR	100	66.24	2
WT	B27	IR	100	71.47	1
D1781N	B27	IR	200	55.21	1
D1781N	B27	IR	200	67.08	2
WT	B27	IR	200	62.93	1
D1781N	B27	IR	400	23.25	1
D1781N	B27	IR	400	28.46	2
WT	B27	IR	400	34.62	1
D1781N	B27	IR	600	16.92	1
D1781N	B27	IR	600	20.25	2
WT	B27	IR	600	20.51	1
I1884T	B28	Camp	2.5	89.53	1
I1884T	B28	Camp	2.5	59.55	2
WT	B28	Camp	2.5	86.75	1
I1884T	B28	Camp	5	71.24	1
I1884T	B28	Camp	5	47.53	2
WT	B28	Camp	5	69.66	1
I1884T	B28	Camp	25	31.50	1
I1884T	B28	Camp	25	14.55	2

Variant[a]	Batch ID[b]	Drug[c]	Concentration[d]	Percent Survived[e]	Clone ID[f]
WT	B28	Camp	25	43.74	1
I1884T	B28	Camp	50	7.21	1
I1884T	B28	Camp	50	1.00	2
WT	B28	Camp	50	17.53	1
I1884T	B28	Camp	100	1.12	1
I1884T	B28	Camp	100	1.00	2
WT	B28	Camp	100	4.86	1
I1884T	B28	Camp	200	1.55	1
I1884T	B28	Camp	200	1.00	2
WT	B28	Camp	200	5.74	1
I1884T	B28	MMC	5	91.40	1
I1884T	B28	MMC	5	37.46	2
WT	B28	MMC	5	75.38	1
I1884T	B28	MMC	10	53.55	1
I1884T	B28	MMC	10	8.07	2
WT	B28	MMC	10	64.87	1
I1884T	B28	MMC	20	39.44	1
I1884T	B28	MMC	20	6.77	2
WT	B28	MMC	20	38.73	1
I1884T	B28	MMC	40	9.53	1
I1884T	B28	MMC	40	1.00	2
WT	B28	MMC	40	17.29	1
I1884T	B28	MMC	60	2.24	1
I1884T	B28	MMC	60	1.00	2
WT	B28	MMC	60	7.61	1
I1884T	B28	MMC	80	2.52	1
I1884T	B28	MMC	80	1.00	2
WT	B28	MMC	80	7.88	1
I1884T	B28	Cisp	0.2	26.25	1
I1884T	B28	Cisp	0.2	3.47	2
WT	B28	Cisp	0.2	41.86	1
I1884T	B28	Cisp	0.4	4.99	1
I1884T	B28	Cisp	0.4	1.00	2
WT	B28	Cisp	0.4	22.09	1
I1884T	B28	Cisp	0.6	3.05	1
I1884T	B28	Cisp	0.6	1.00	2
WT	B28	Cisp	0.6	13.29	1
I1884T	B28	Cisp	1	1.00	1
I1884T	B28	Cisp	1	1.00	2
WT	B28	Cisp	1	10.13	1

Variant[a]	Batch ID[b]	Drug[c]	Concentration[d]	Percent Survived[e]	Clone ID[f]
I1884T	B28	Cisp	1.2	1.00	1
I1884T	B28	Cisp	1.2	1.00	2
WT	B28	Cisp	1.2	6.98	1
I1884T	B28	Cisp	1.5	1.00	1
I1884T	B28	Cisp	1.5	1.00	2
WT	B28	Cisp	1.5	8.80	1
I1884T	B28	MMS	5	55.22	1
I1884T	B28	MMS	5	36.35	2
WT	B28	MMS	5	60.12	1
I1884T	B28	MMS	10	31.94	1
I1884T	B28	MMS	10	17.29	2
WT	B28	MMS	10	43.95	1
I1884T	B28	MMS	15	13.51	1
I1884T	B28	MMS	15	1.00	2
WT	B28	MMS	15	25.93	1
I1884T	B28	MMS	20	4.39	1
I1884T	B28	MMS	20	1.00	2
WT	B28	MMS	20	16.51	1
I1884T	B28	MMS	30	1.00	1
I1884T	B28	MMS	30	1.00	2
WT	B28	MMS	30	7.09	1
I1884T	B28	MMS	40	2.13	1
I1884T	B28	MMS	40	1.00	2
WT	B28	MMS	40	10.47	1
I1884T	B28	Parp	0.01	63.71	1
I1884T	B28	Parp	0.01	42.86	2
WT	B28	Parp	0.01	81.03	1
I1884T	B28	Parp	0.1	39.11	1
I1884T	B28	Parp	0.1	23.27	2
WT	B28	Parp	0.1	48.58	1
I1884T	B28	Parp	1	9.10	1
I1884T	B28	Parp	1	1.00	2
WT	B28	Parp	1	18.09	1
I1884T	B28	Parp	10	2.93	1
I1884T	B28	Parp	10	1.00	2
WT	B28	Parp	10	11.35	1
I1884T	B28	IR	50	85.31	1
I1884T	B28	IR	50	89.83	2
WT	B28	IR	50	100.00	1
I1884T	B28	IR	100	84.62	1

Variant[a]	Batch ID[b]	Drug[c]	Concentration[d]	Percent Survived[e]	Clone ID[f]
I1884T	B28	IR	100	80.60	2
WT	B28	IR	100	73.86	1
I1884T	B28	IR	200	55.33	1
I1884T	B28	IR	200	73.88	2
WT	B28	IR	200	72.81	1
I1884T	B28	IR	400	29.19	1
I1884T	B28	IR	400	30.41	2
WT	B28	IR	400	36.32	1
I1884T	B28	IR	600	23.37	1
I1884T	B28	IR	600	17.16	2
WT	B28	IR	600	26.32	1
V220Ifs	B29	Camp	2.5	100.00	1
V220Ifs	B29	Camp	2.5	85.95	2
WT	B29	Camp	2.5	99.27	1
V220Ifs	B29	Camp	5	100.00	1
V220Ifs	B29	Camp	5	78.71	2
WT	B29	Camp	5	100.00	1
V220Ifs	B29	Camp	25	52.82	1
V220Ifs	B29	Camp	25	5.79	2
WT	B29	Camp	25	84.28	1
V220Ifs	B29	Camp	50	6.21	1
V220Ifs	B29	Camp	50	1.00	2
WT	B29	Camp	50	61.46	1
V220Ifs	B29	Camp	100	1.00	1
V220Ifs	B29	Camp	100	1.00	2
WT	B29	Camp	100	10.31	1
V220Ifs	B29	Camp	200	1.00	1
V220Ifs	B29	Camp	200	1.00	2
WT	B29	Camp	200	1.92	1
V220Ifs	B29	MMC	5	75.93	1
V220Ifs	B29	MMC	5	40.31	2
WT	B29	MMC	5	100.00	1
V220Ifs	B29	MMC	10	65.24	1
V220Ifs	B29	MMC	10	13.36	2
WT	B29	MMC	10	100.00	1
V220Ifs	B29	MMC	20	18.56	1
V220Ifs	B29	MMC	20	1.00	2
WT	B29	MMC	20	80.58	1
V220Ifs	B29	MMC	40	3.60	1
V220Ifs	B29	MMC	40	1.00	2

Variant[a]	Batch ID[b]	Drug[c]	Concentration[d]	Percent Survived[e]	Clone ID[f]
WT	B29	MMC	40	62.97	1
V220Ifs	B29	MMC	60	1.35	1
V220Ifs	B29	MMC	60	1.00	2
WT	B29	MMC	60	39.00	1
V220Ifs	B29	MMC	80	2.25	1
V220Ifs	B29	MMC	80	1.00	2
WT	B29	MMC	80	20.94	1
V220Ifs	B29	Cisp	0.2	26.04	1
V220Ifs	B29	Cisp	0.2	9.23	2
WT	B29	Cisp	0.2	62.89	1
V220Ifs	B29	Cisp	0.4	6.38	1
V220Ifs	B29	Cisp	0.4	1.00	2
WT	B29	Cisp	0.4	46.89	1
V220Ifs	B29	Cisp	0.6	1.00	1
V220Ifs	B29	Cisp	0.6	1.00	2
WT	B29	Cisp	0.6	14.33	1
V220Ifs	B29	Cisp	1	1.51	1
V220Ifs	B29	Cisp	1	1.00	2
WT	B29	Cisp	1	11.45	1
V220Ifs	B29	Cisp	1.2	1.15	1
V220Ifs	B29	Cisp	1.2	1.00	2
WT	B29	Cisp	1.2	6.27	1
V220Ifs	B29	Cisp	1.5	1.29	1
V220Ifs	B29	Cisp	1.5	1.00	2
WT	B29	Cisp	1.5	5.18	1
V220Ifs	B29	MMS	5	81.19	1
V220Ifs	B29	MMS	5	66.87	2
WT	B29	MMS	5	100.00	1
V220Ifs	B29	MMS	10	43.49	1
V220Ifs	B29	MMS	10	13.51	2
WT	B29	MMS	10	99.87	1
V220Ifs	B29	MMS	15	14.55	1
V220Ifs	B29	MMS	15	2.67	2
WT	B29	MMS	15	95.09	1
V220Ifs	B29	MMS	20	2.67	1
V220Ifs	B29	MMS	20	1.00	2
WT	B29	MMS	20	71.64	1
V220Ifs	B29	MMS	30	1.00	1
V220Ifs	B29	MMS	30	1.00	2
WT	B29	MMS	30	32.39	1

Variant[a]	Batch ID[b]	Drug[c]	Concentration[d]	Percent Survived[e]	Clone ID[f]
V220Ifs	B29	MMS	40	1.07	1
V220Ifs	B29	MMS	40	1.00	2
WT	B29	MMS	40	8.00	1
V220Ifs	B29	Parp	0.01	73.54	1
V220Ifs	B29	Parp	0.01	37.82	2
WT	B29	Parp	0.01	98.15	1
V220Ifs	B29	Parp	0.1	34.81	1
V220Ifs	B29	Parp	0.1	2.13	2
WT	B29	Parp	0.1	87.48	1
V220Ifs	B29	Parp	1	3.34	1
V220Ifs	B29	Parp	1	1.00	2
WT	B29	Parp	1	64.64	1
V220Ifs	B29	Parp	10	2.00	1
V220Ifs	B29	Parp	10	1.00	2
WT	B29	Parp	10	11.39	1
V220Ifs	B29	IR	50	88.80	1
V220Ifs	B29	IR	50	82.48	2
WT	B29	IR	50	100.00	1
V220Ifs	B29	IR	100	55.06	1
V220Ifs	B29	IR	100	38.99	2
WT	B29	IR	100	80.92	1
V220Ifs	B29	IR	200	34.13	1
V220Ifs	B29	IR	200	24.31	2
WT	B29	IR	200	69.57	1
V220Ifs	B29	IR	400	8.80	1
V220Ifs	B29	IR	400	8.72	2
WT	B29	IR	400	45.41	1
V220Ifs	B29	IR	600	7.72	1
V220Ifs	B29	IR	600	7.52	2
WT	B29	IR	600	29.37	1
C3069X	B30	Camp	2.5	82.56	1
C3069X	B30	Camp	2.5	69.10	2
WT	B30	Camp	2.5	92.65	1
C3069X	B30	Camp	5	72.85	1
C3069X	B30	Camp	5	73.82	2
WT	B30	Camp	5	91.02	1
C3069X	B30	Camp	25	16.67	1
C3069X	B30	Camp	25	6.16	2
WT	B30	Camp	25	61.35	1
C3069X	B30	Camp	50	5.30	1

Variant[a]	Batch ID[b]	Drug[c]	Concentration[d]	Percent Survived[e]	Clone ID[f]
C3069X	B30	Camp	50	3.29	2
WT	B30	Camp	50	30.59	1
C3069X	B30	Camp	100	1.66	1
C3069X	B30	Camp	100	2.36	2
WT	B30	Camp	100	4.90	1
C3069X	B30	Camp	200	2.32	1
C3069X	B30	Camp	200	2.87	2
WT	B30	Camp	200	1.96	1
C3069X	B30	MMC	5	77.94	1
C3069X	B30	MMC	5	45.89	2
WT	B30	MMC	5	100.00	1
C3069X	B30	MMC	10	33.67	1
C3069X	B30	MMC	10	21.71	2
WT	B30	MMC	10	97.28	1
C3069X	B30	MMC	20	16.58	1
C3069X	B30	MMC	20	15.19	2
WT	B30	MMC	20	58.44	1
C3069X	B30	MMC	40	7.13	1
C3069X	B30	MMC	40	9.77	2
WT	B30	MMC	40	26.01	1
C3069X	B30	MMC	60	4.98	1
C3069X	B30	MMC	60	8.37	2
WT	B30	MMC	60	11.95	1
C3069X	B30	MMC	80	5.97	1
C3069X	B30	MMC	80	7.75	2
WT	B30	MMC	80	8.70	1
C3069X	B30	Cisp	0.2	26.71	1
C3069X	B30	Cisp	0.2	22.00	2
WT	B30	Cisp	0.2	58.07	1
C3069X	B30	Cisp	0.4	10.54	1
C3069X	B30	Cisp	0.4	8.57	2
WT	B30	Cisp	0.4	33.53	1
C3069X	B30	Cisp	0.6	6.83	1
C3069X	B30	Cisp	0.6	10.14	2
WT	B30	Cisp	0.6	19.32	1
C3069X	B30	Cisp	1	6.59	1
C3069X	B30	Cisp	1	6.57	2
WT	B30	Cisp	1	8.93	1
C3069X	B30	Cisp	1.2	6.83	1
C3069X	B30	Cisp	1.2	7.71	2

Variant[a]	Batch ID[b]	Drug[c]	Concentration[d]	Percent Survived[e]	Clone ID[f]
WT	B30	Cisp	1.2	6.99	1
C3069X	B30	Cisp	1.5	6.83	1
C3069X	B30	Cisp	1.5	6.86	2
WT	B30	Cisp	1.5	5.81	1
C3069X	B30	MMS	5	39.53	1
C3069X	B30	MMS	5	31.49	2
WT	B30	MMS	5	87.49	1
C3069X	B30	MMS	10	11.52	1
C3069X	B30	MMS	10	6.19	2
WT	B30	MMS	10	77.98	1
C3069X	B30	MMS	15	4.32	1
C3069X	B30	MMS	15	3.77	2
WT	B30	MMS	15	36.55	1
C3069X	B30	MMS	20	3.80	1
C3069X	B30	MMS	20	1.08	2
WT	B30	MMS	20	30.49	1
C3069X	B30	MMS	30	1.31	1
C3069X	B30	MMS	30	1.00	2
WT	B30	MMS	30	5.15	1
C3069X	B30	MMS	40	1.18	1
C3069X	B30	MMS	40	1.00	2
WT	B30	MMS	40	2.28	1
C3069X	B30	Parp	0.01	31.84	1
C3069X	B30	Parp	0.01	13.18	2
WT	B30	Parp	0.01	91.98	1
C3069X	B30	Parp	0.1	8.09	1
C3069X	B30	Parp	0.1	3.32	2
WT	B30	Parp	0.1	67.25	1
C3069X	B30	Parp	1	3.08	1
C3069X	B30	Parp	1	3.88	2
WT	B30	Parp	1	37.35	1
C3069X	B30	Parp	10	2.82	1
C3069X	B30	Parp	10	4.98	2
WT	B30	Parp	10	3.11	1
C3069X	B30	IR	50	91.30	1
C3069X	B30	IR	50	75.06	2
WT	B30	IR	50	100.00	1
C3069X	B30	IR	100	76.65	1
C3069X	B30	IR	100	57.52	2
WT	B30	IR	100	97.96	1

Variant[a]	Batch ID[b]	Drug[c]	Concentration[d]	Percent Survived[e]	Clone ID[f]
C3069X	B30	IR	200	44.28	1
C3069X	B30	IR	200	31.50	2
WT	B30	IR	200	67.13	1
C3069X	B30	IR	400	24.96	1
C3069X	B30	IR	400	19.21	2
WT	B30	IR	400	51.54	1
C3069X	B30	IR	600	19.81	1
C3069X	B30	IR	600	17.78	2
WT	B30	IR	600	17.49	1
R2336P	B31	Camp	2.5	76.49	1
R2336P	B31	Camp	2.5	73.16	2
WT	B31	Camp	2.5	82.34	1
R2336P	B31	Camp	5	60.07	1
R2336P	B31	Camp	5	70.83	2
WT	B31	Camp	5	87.74	1
R2336P	B31	Camp	25	12.37	1
R2336P	B31	Camp	25	43.64	2
WT	B31	Camp	25	63.21	1
R2336P	B31	Camp	50	9.45	1
R2336P	B31	Camp	50	33.26	2
WT	B31	Camp	50	61.27	1
R2336P	B31	Camp	100	5.40	1
R2336P	B31	Camp	100	4.99	2
WT	B31	Camp	100	12.37	1
R2336P	B31	Camp	200	3.60	1
R2336P	B31	Camp	200	1.00	2
WT	B31	Camp	200	1.83	1
R2336P	B31	MMC	5	24.48	1
R2336P	B31	MMC	5	75.72	2
WT	B31	MMC	5	73.23	1
R2336P	B31	MMC	10	9.64	1
R2336P	B31	MMC	10	57.19	2
WT	B31	MMC	10	68.09	1
R2336P	B31	MMC	20	5.12	1
R2336P	B31	MMC	20	39.53	2
WT	B31	MMC	20	50.82	1
R2336P	B31	MMC	40	3.91	1
R2336P	B31	MMC	40	13.58	2
WT	B31	MMC	40	25.28	1
R2336P	B31	MMC	60	3.91	1

Variant[a]	Batch ID[b]	Drug[c]	Concentration[d]	Percent Survived[e]	Clone ID[f]
R2336P	B31	MMC	60	4.89	2
WT	B31	MMC	60	11.51	1
R2336P	B31	MMC	80	3.39	1
R2336P	B31	MMC	80	1.00	2
WT	B31	MMC	80	4.42	1
R2336P	B31	Cisp	0.2	8.65	1
R2336P	B31	Cisp	0.2	46.73	2
WT	B31	Cisp	0.2	73.37	1
R2336P	B31	Cisp	0.4	4.66	1
R2336P	B31	Cisp	0.4	21.21	2
WT	B31	Cisp	0.4	48.08	1
R2336P	B31	Cisp	0.6	5.32	1
R2336P	B31	Cisp	0.6	12.95	2
WT	B31	Cisp	0.6	26.74	1
R2336P	B31	Cisp	1	4.88	1
R2336P	B31	Cisp	1	2.65	2
WT	B31	Cisp	1	11.10	1
R2336P	B31	Cisp	1.2	4.99	1
R2336P	B31	Cisp	1.2	1.79	2
WT	B31	Cisp	1.2	6.40	1
R2336P	B31	Cisp	1.5	3.66	1
R2336P	B31	Cisp	1.5	1.00	2
WT	B31	Cisp	1.5	3.78	1
R2336P	B31	MMS	5	42.33	1
R2336P	B31	MMS	5	79.43	2
WT	B31	MMS	5	88.55	1
R2336P	B31	MMS	10	11.99	1
R2336P	B31	MMS	10	59.13	2
WT	B31	MMS	10	74.33	1
R2336P	B31	MMS	15	8.10	1
R2336P	B31	MMS	15	54.38	2
WT	B31	MMS	15	62.83	1
R2336P	B31	MMS	20	2.81	1
R2336P	B31	MMS	20	18.13	2
WT	B31	MMS	20	45.53	1
R2336P	B31	MMS	30	2.27	1
R2336P	B31	MMS	30	2.04	2
WT	B31	MMS	30	7.11	1
R2336P	B31	MMS	40	1.73	1
R2336P	B31	MMS	40	1.00	2

Variant[a]	Batch ID[b]	Drug[c]	Concentration[d]	Percent Survived[e]	Clone ID[f]
WT	B31	MMS	40	1.83	1
R2336P	B31	Parp	0.01	24.61	1
R2336P	B31	Parp	0.01	71.43	2
WT	B31	Parp	0.01	95.72	1
R2336P	B31	Parp	0.1	11.02	1
R2336P	B31	Parp	0.1	56.25	2
WT	B31	Parp	0.1	97.06	1
R2336P	B31	Parp	1	8.24	1
R2336P	B31	Parp	1	17.08	2
WT	B31	Parp	1	71.78	1
R2336P	B31	Parp	10	5.77	1
R2336P	B31	Parp	10	4.06	2
WT	B31	Parp	10	4.44	1
R2336P	B31	IR	50	68.15	1
R2336P	B31	IR	50	92.28	2
WT	B31	IR	50	96.52	1
R2336P	B31	IR	100	49.70	1
R2336P	B31	IR	100	87.88	2
WT	B31	IR	100	83.74	1
R2336P	B31	IR	200	28.95	1
R2336P	B31	IR	200	63.59	2
WT	B31	IR	200	72.86	1
R2336P	B31	IR	400	13.03	1
R2336P	B31	IR	400	32.64	2
WT	B31	IR	400	49.26	1
R2336P	B31	IR	600	9.29	1
R2336P	B31	IR	600	22.10	2
WT	B31	IR	600	37.80	1
K2411T	B32	Camp	2.5	92.00	1
K2411T	B32	Camp	2.5	84.00	2
WT	B32	Camp	2.5	74.00	1
K2411T	B32	Camp	5	95.00	1
K2411T	B32	Camp	5	89.00	2
WT	B32	Camp	5	75.00	1
K2411T	B32	Camp	25	55.00	1
K2411T	B32	Camp	25	63.00	2
WT	B32	Camp	25	60.00	1
K2411T	B32	Camp	50	22.00	1
K2411T	B32	Camp	50	29.00	2
WT	B32	Camp	50	36.00	1

Variant[a]	Batch ID[b]	Drug[c]	Concentration[d]	Percent Survived[e]	Clone ID[f]
K2411T	B32	Camp	100	3.00	1
K2411T	B32	Camp	100	4.00	2
WT	B32	Camp	100	9.00	1
K2411T	B32	Camp	200	2.00	1
K2411T	B32	Camp	200	1.00	2
WT	B32	Camp	200	3.00	1
K2411T	B32	MMC	5	100.00	1
K2411T	B32	MMC	5	100.00	2
WT	B32	MMC	5	100.00	1
K2411T	B32	MMC	10	100.00	1
K2411T	B32	MMC	10	97.00	2
WT	B32	MMC	10	88.00	1
K2411T	B32	MMC	20	100.00	1
K2411T	B32	MMC	20	100.00	2
WT	B32	MMC	20	92.00	1
K2411T	B32	MMC	40	76.00	1
K2411T	B32	MMC	40	55.00	2
WT	B32	MMC	40	53.00	1
K2411T	B32	MMC	60	30.00	1
K2411T	B32	MMC	60	25.00	2
WT	B32	MMC	60	28.00	1
K2411T	B32	MMC	80	15.00	1
K2411T	B32	MMC	80	15.00	2
WT	B32	MMC	80	15.00	1
K2411T	B32	Cisp	0.2	49.00	1
K2411T	B32	Cisp	0.2	46.00	2
WT	B32	Cisp	0.2	52.00	1
K2411T	B32	Cisp	0.4	22.00	1
K2411T	B32	Cisp	0.4	21.00	2
WT	B32	Cisp	0.4	32.00	1
K2411T	B32	Cisp	0.6	12.00	1
K2411T	B32	Cisp	0.6	11.00	2
WT	B32	Cisp	0.6	18.00	1
K2411T	B32	Cisp	1	8.00	1
K2411T	B32	Cisp	1	6.00	2
WT	B32	Cisp	1	7.00	1
K2411T	B32	Cisp	1.2	6.00	1
K2411T	B32	Cisp	1.2	4.00	2
WT	B32	Cisp	1.2	7.00	1
K2411T	B32	Cisp	1.5	5.00	1

Variant[a]	Batch ID[b]	Drug[c]	Concentration[d]	Percent Survived[e]	Clone ID[f]
K2411T	B32	Cisp	1.5	3.00	2
WT	B32	Cisp	1.5	7.00	1
K2411T	B32	MMS	5	100.00	1
K2411T	B32	MMS	5	74.00	2
WT	B32	MMS	5	83.00	1
K2411T	B32	MMS	10	100.00	1
K2411T	B32	MMS	10	65.00	2
WT	B32	MMS	10	82.00	1
K2411T	B32	MMS	15	89.00	1
K2411T	B32	MMS	15	63.00	2
WT	B32	MMS	15	73.00	1
K2411T	B32	MMS	20	52.00	1
K2411T	B32	MMS	20	40.00	2
WT	B32	MMS	20	52.00	1
K2411T	B32	MMS	30	5.00	1
K2411T	B32	MMS	30	11.00	2
WT	B32	MMS	30	20.00	1
K2411T	B32	MMS	40	1.00	1
K2411T	B32	MMS	40	2.00	2
WT	B32	MMS	40	4.00	1
K2411T	B32	Parp	0.01	6.00	1
K2411T	B32	Parp	0.01	97.00	2
WT	B32	Parp	0.01	100.00	1
K2411T	B32	Parp	0.1	80.00	1
K2411T	B32	Parp	0.1	89.00	2
WT	B32	Parp	0.1	97.00	1
K2411T	B32	Parp	1	73.00	1
K2411T	B32	Parp	1	84.00	2
WT	B32	Parp	1	86.00	1
K2411T	B32	Parp	10	36.00	1
K2411T	B32	Parp	10	36.00	2
WT	B32	Parp	10	38.00	1
K2411T	B32	IR	50	76.00	1
K2411T	B32	IR	50	76.00	2
WT	B32	IR	50	68.00	1
K2411T	B32	IR	100	63.00	1
K2411T	B32	IR	100	64.00	2
WT	B32	IR	100	64.00	1
K2411T	B32	IR	200	33.00	1
K2411T	B32	IR	200	38.00	2

Variant[a]	Batch ID[b]	Drug[c]	Concentration[d]	Percent Survived[e]	Clone ID[f]
WT	B32	IR	200	38.00	1
K2411T	B32	IR	400	18.00	1
K2411T	B32	IR	400	13.00	2
WT	B32	IR	400	21.00	1
K2411T	B32	IR	600	11.00	1
K2411T	B32	IR	600	11.00	2
WT	B32	IR	600	11.00	1
N1880K	B33	Camp	2.5	71.00	1
N1880K	B33	Camp	2.5	63.00	2
WT	B33	Camp	2.5	74.00	1
N1880K	B33	Camp	5	67.00	1
N1880K	B33	Camp	5	61.00	2
WT	B33	Camp	5	75.00	1
N1880K	B33	Camp	25	59.00	1
N1880K	B33	Camp	25	45.00	2
WT	B33	Camp	25	60.00	1
N1880K	B33	Camp	50	28.00	1
N1880K	B33	Camp	50	27.00	2
WT	B33	Camp	50	36.00	1
N1880K	B33	Camp	100	5.00	1
N1880K	B33	Camp	100	7.00	2
WT	B33	Camp	100	9.00	1
N1880K	B33	Camp	200	4.00	1
N1880K	B33	Camp	200	4.00	2
WT	B33	Camp	200	3.00	1
N1880K	B33	MMC	5	100.00	1
N1880K	B33	MMC	5	94.00	2
WT	B33	MMC	5	100.00	1
N1880K	B33	MMC	10	100.00	1
N1880K	B33	MMC	10	88.00	2
WT	B33	MMC	10	88.00	1
N1880K	B33	MMC	20	100.00	1
N1880K	B33	MMC	20	91.00	2
WT	B33	MMC	20	92.00	1
N1880K	B33	MMC	40	96.00	1
N1880K	B33	MMC	40	65.00	2
WT	B33	MMC	40	53.00	1
N1880K	B33	MMC	60	66.00	1
N1880K	B33	MMC	60	35.00	2
WT	B33	MMC	60	28.00	1

Variant[a]	Batch ID[b]	Drug[c]	Concentration[d]	Percent Survived[e]	Clone ID[f]
N1880K	B33	MMC	80	31.00	1
N1880K	B33	MMC	80	18.00	2
WT	B33	MMC	80	15.00	1
N1880K	B33	Cisp	0.2	50.00	1
N1880K	B33	Cisp	0.2	51.00	2
WT	B33	Cisp	0.2	52.00	1
N1880K	B33	Cisp	0.4	24.00	1
N1880K	B33	Cisp	0.4	33.00	2
WT	B33	Cisp	0.4	32.00	1
N1880K	B33	Cisp	0.6	17.00	1
N1880K	B33	Cisp	0.6	25.00	2
WT	B33	Cisp	0.6	18.00	1
N1880K	B33	Cisp	1	9.00	1
N1880K	B33	Cisp	1	17.00	2
WT	B33	Cisp	1	7.00	1
N1880K	B33	Cisp	1.2	9.00	1
N1880K	B33	Cisp	1.2	13.00	2
WT	B33	Cisp	1.2	7.00	1
N1880K	B33	Cisp	1.5	8.00	1
N1880K	B33	Cisp	1.5	9.00	2
WT	B33	Cisp	1.5	7.00	1
N1880K	B33	MMS	5	84.00	1
N1880K	B33	MMS	5	85.00	2
WT	B33	MMS	5	83.00	1
N1880K	B33	MMS	10	99.00	1
N1880K	B33	MMS	10	87.00	2
WT	B33	MMS	10	82.00	1
N1880K	B33	MMS	15	76.00	1
N1880K	B33	MMS	15	58.00	2
WT	B33	MMS	15	73.00	1
N1880K	B33	MMS	20	48.00	1
N1880K	B33	MMS	20	34.00	2
WT	B33	MMS	20	52.00	1
N1880K	B33	MMS	30	10.00	1
N1880K	B33	MMS	30	6.00	2
WT	B33	MMS	30	20.00	1
N1880K	B33	MMS	40	4.00	1
N1880K	B33	MMS	40	4.00	2
WT	B33	MMS	40	4.00	1
N1880K	B33	Parp	0.01	100.00	1

Variant[a]	Batch ID[b]	Drug[c]	Concentration[d]	Percent Survived[e]	Clone ID[f]
N1880K	B33	Parp	0.01	96.00	2
WT	B33	Parp	0.01	99.00	1
N1880K	B33	Parp	0.1	93.00	1
N1880K	B33	Parp	0.1	76.00	2
WT	B33	Parp	0.1	99.00	1
N1880K	B33	Parp	1	91.00	1
N1880K	B33	Parp	1	82.00	2
WT	B33	Parp	1	85.00	1
N1880K	B33	Parp	10	21.00	1
N1880K	B33	Parp	10	5.00	2
WT	B33	Parp	10	16.00	1
N1880K	B33	IR	50	76.00	1
N1880K	B33	IR	50	71.00	2
WT	B33	IR	50	68.00	1
N1880K	B33	IR	100	61.00	1
N1880K	B33	IR	100	71.00	2
WT	B33	IR	100	64.00	1
N1880K	B33	IR	200	32.00	1
N1880K	B33	IR	200	38.00	2
WT	B33	IR	200	38.00	1
N1880K	B33	IR	400	14.00	1
N1880K	B33	IR	400	17.00	2
WT	B33	IR	400	21.00	1
N1880K	B33	IR	600	10.00	1
N1880K	B33	IR	600	12.00	2
WT	B33	IR	600	11.00	1
R2488K	B34	Camp	2.5	83.00	1
R2488K	B34	Camp	2.5	81.00	2
WT	B34	Camp	2.5	100.00	1
R2488K	B34	Camp	5	97.00	1
R2488K	B34	Camp	5	92.00	2
WT	B34	Camp	5	94.00	1
R2488K	B34	Camp	25	93.00	1
R2488K	B34	Camp	25	63.00	2
WT	B34	Camp	25	80.00	1
R2488K	B34	Camp	50	44.00	1
R2488K	B34	Camp	50	30.00	2
WT	B34	Camp	50	59.00	1
R2488K	B34	Camp	100	13.00	1
R2488K	B34	Camp	100	8.00	2

Variant[a]	Batch ID[b]	Drug[c]	Concentration[d]	Percent Survived[e]	Clone ID[f]
WT	B34	Camp	100	19.00	1
R2488K	B34	Camp	200	8.00	1
R2488K	B34	Camp	200	4.00	2
WT	B34	Camp	200	9.00	1
R2488K	B34	MMC	5	81.00	1
R2488K	B34	MMC	5	85.00	2
WT	B34	MMC	5	92.00	1
R2488K	B34	MMC	10	79.00	1
R2488K	B34	MMC	10	90.00	2
WT	B34	MMC	10	88.00	1
R2488K	B34	MMC	20	67.00	1
R2488K	B34	MMC	20	78.00	2
WT	B34	MMC	20	71.00	1
R2488K	B34	MMC	40	52.00	1
R2488K	B34	MMC	40	43.00	2
WT	B34	MMC	40	55.00	1
R2488K	B34	MMC	60	36.00	1
R2488K	B34	MMC	60	28.00	2
WT	B34	MMC	60	45.00	1
R2488K	B34	MMC	80	28.00	1
R2488K	B34	MMC	80	17.00	2
WT	B34	MMC	80	41.00	1
R2488K	B34	Cisp	0.2	61.00	1
R2488K	B34	Cisp	0.2	61.00	2
WT	B34	Cisp	0.2	65.00	1
R2488K	B34	Cisp	0.4	28.00	1
R2488K	B34	Cisp	0.4	27.00	2
WT	B34	Cisp	0.4	43.00	1
R2488K	B34	Cisp	0.6	9.00	1
R2488K	B34	Cisp	0.6	13.00	2
WT	B34	Cisp	0.6	23.00	1
R2488K	B34	Cisp	1	5.00	1
R2488K	B34	Cisp	1	5.00	2
WT	B34	Cisp	1	8.00	1
R2488K	B34	Cisp	1.2	3.00	1
R2488K	B34	Cisp	1.2	5.00	2
WT	B34	Cisp	1.2	4.00	1
R2488K	B34	Cisp	1.5	2.00	1
R2488K	B34	Cisp	1.5	3.00	2
WT	B34	Cisp	1.5	3.00	1

Variant[a]	Batch ID[b]	Drug[c]	Concentration[d]	Percent Survived[e]	Clone ID[f]
R2488K	B34	MMS	5	100.00	1
R2488K	B34	MMS	5	100.00	2
WT	B34	MMS	5	100.00	1
R2488K	B34	MMS	10	81.00	1
R2488K	B34	MMS	10	87.00	2
WT	B34	MMS	10	100.00	1
R2488K	B34	MMS	15	47.00	1
R2488K	B34	MMS	15	47.00	2
WT	B34	MMS	15	85.00	1
R2488K	B34	MMS	20	14.00	1
R2488K	B34	MMS	20	19.00	2
WT	B34	MMS	20	59.00	1
R2488K	B34	MMS	30	1.00	1
R2488K	B34	MMS	30	1.00	2
WT	B34	MMS	30	24.00	1
R2488K	B34	MMS	40	2.00	1
R2488K	B34	MMS	40	1.00	2
WT	B34	MMS	40	6.00	1
R2488K	B34	Parp	0.01	70.00	1
R2488K	B34	Parp	0.01	77.00	2
WT	B34	Parp	0.01	91.00	1
R2488K	B34	Parp	0.1	43.00	1
R2488K	B34	Parp	0.1	49.00	2
WT	B34	Parp	0.1	70.00	1
R2488K	B34	Parp	1	5.00	1
R2488K	B34	Parp	1	6.00	2
WT	B34	Parp	1	7.00	1
R2488K	B34	Parp	10	6.00	1
R2488K	B34	Parp	10	7.00	2
WT	B34	Parp	10	6.00	1
R2488K	B34	IR	50	86.24	1
R2488K	B34	IR	50	78.46	2
WT	B34	IR	50	100.00	1
R2488K	B34	IR	100	81.02	1
R2488K	B34	IR	100	76.63	2
WT	B34	IR	100	87.06	1
R2488K	B34	IR	200	53.63	1
R2488K	B34	IR	200	58.00	2
WT	B34	IR	200	61.99	1
R2488K	B34	IR	400	34.65	1

Variant[a]	Batch ID[b]	Drug[c]	Concentration[d]	Percent Survived[e]	Clone ID[f]
R2488K	B34	IR	400	33.23	2
WT	B34	IR	400	48.78	1
R2488K	B34	IR	600	21.01	1
R2488K	B34	IR	600	27.60	2
WT	B34	IR	600	31.94	1
D2489G	B35	Camp	2.5	75.00	1
D2489G	B35	Camp	2.5	92.00	2
WT	B35	Camp	2.5	100.00	1
D2489G	B35	Camp	5	85.00	1
D2489G	B35	Camp	5	93.00	2
WT	B35	Camp	5	94.00	1
D2489G	B35	Camp	25	77.00	1
D2489G	B35	Camp	25	65.00	2
WT	B35	Camp	25	80.00	1
D2489G	B35	Camp	50	33.00	1
D2489G	B35	Camp	50	38.00	2
WT	B35	Camp	50	59.00	1
D2489G	B35	Camp	100	20.00	1
D2489G	B35	Camp	100	7.00	2
WT	B35	Camp	100	19.00	1
D2489G	B35	Camp	200	7.00	1
D2489G	B35	Camp	200	2.00	2
WT	B35	Camp	200	9.00	1
D2489G	B35	MMC	5	94.00	1
D2489G	B35	MMC	5	100.00	2
WT	B35	MMC	5	92.00	1
D2489G	B35	MMC	10	94.00	1
D2489G	B35	MMC	10	100.00	2
WT	B35	MMC	10	88.00	1
D2489G	B35	MMC	20	95.00	1
D2489G	B35	MMC	20	92.00	2
WT	B35	MMC	20	71.00	1
D2489G	B35	MMC	40	60.00	1
D2489G	B35	MMC	40	77.00	2
WT	B35	MMC	40	55.00	1
D2489G	B35	MMC	60	66.00	1
D2489G	B35	MMC	60	58.00	2
WT	B35	MMC	60	45.00	1
D2489G	B35	MMC	80	42.00	1
D2489G	B35	MMC	80	34.00	2

Variant[a]	Batch ID[b]	Drug[c]	Concentration[d]	Percent Survived[e]	Clone ID[f]
WT	B35	MMC	80	41.00	1
D2489G	B35	Cisp	0.2	52.00	1
D2489G	B35	Cisp	0.2	36.00	2
WT	B35	Cisp	0.2	65.00	1
D2489G	B35	Cisp	0.4	25.00	1
D2489G	B35	Cisp	0.4	17.00	2
WT	B35	Cisp	0.4	43.00	1
D2489G	B35	Cisp	0.6	13.00	1
D2489G	B35	Cisp	0.6	6.00	2
WT	B35	Cisp	0.6	23.00	1
D2489G	B35	Cisp	1	5.00	1
D2489G	B35	Cisp	1	3.00	2
WT	B35	Cisp	1	8.00	1
D2489G	B35	Cisp	1.2	4.00	1
D2489G	B35	Cisp	1.2	2.00	2
WT	B35	Cisp	1.2	4.00	1
D2489G	B35	Cisp	1.5	3.00	1
D2489G	B35	Cisp	1.5	2.00	2
WT	B35	Cisp	1.5	3.00	1
D2489G	B35	MMS	5	96.00	1
D2489G	B35	MMS	5	81.00	2
WT	B35	MMS	5	100.00	1
D2489G	B35	MMS	10	65.00	1
D2489G	B35	MMS	10	59.00	2
WT	B35	MMS	10	100.00	1
D2489G	B35	MMS	15	44.00	1
D2489G	B35	MMS	15	39.00	2
WT	B35	MMS	15	85.00	1
D2489G	B35	MMS	20	19.00	1
D2489G	B35	MMS	20	22.00	2
WT	B35	MMS	20	59.00	1
D2489G	B35	MMS	30	1.00	1
D2489G	B35	MMS	30	1.00	2
WT	B35	MMS	30	24.00	1
D2489G	B35	MMS	40	1.00	1
D2489G	B35	MMS	40	1.00	2
WT	B35	MMS	40	6.00	1
D2489G	B35	Parp	0.01	62.00	1
D2489G	B35	Parp	0.01	60.00	2
WT	B35	Parp	0.01	91.00	1

Variant[a]	Batch ID[b]	Drug[c]	Concentration[d]	Percent Survived[e]	Clone ID[f]
D2489G	B35	Parp	0.1	31.00	1
D2489G	B35	Parp	0.1	25.00	2
WT	B35	Parp	0.1	66.00	1
D2489G	B35	Parp	1	7.00	1
D2489G	B35	Parp	1	1.00	2
WT	B35	Parp	1	7.00	1
D2489G	B35	Parp	10	9.00	1
D2489G	B35	Parp	10	1.00	2
WT	B35	Parp	10	6.00	1
D2489G	B35	IR	50	99.00	1
D2489G	B35	IR	50	100.00	2
WT	B35	IR	50	100.00	1
D2489G	B35	IR	100	74.00	1
D2489G	B35	IR	100	91.00	2
WT	B35	IR	100	87.00	1
D2489G	B35	IR	200	60.00	1
D2489G	B35	IR	200	58.00	2
WT	B35	IR	200	62.00	1
D2489G	B35	IR	400	42.00	1
D2489G	B35	IR	400	35.00	2
WT	B35	IR	400	49.00	1
D2489G	B35	IR	600	28.00	1
D2489G	B35	IR	600	23.00	2
WT	B35	IR	600	32.00	1
F3065L	B36	Camp	2.5	74.00	1
F3065L	B36	Camp	2.5	90.00	2
WT	B36	Camp	2.5	74.00	1
F3065L	B36	Camp	5	80.00	1
F3065L	B36	Camp	5	92.00	2
WT	B36	Camp	5	75.00	1
F3065L	B36	Camp	25	48.00	1
F3065L	B36	Camp	25	55.00	2
WT	B36	Camp	25	60.00	1
F3065L	B36	Camp	50	24.00	1
F3065L	B36	Camp	50	17.00	2
WT	B36	Camp	50	36.00	1
F3065L	B36	Camp	100	8.00	1
F3065L	B36	Camp	100	3.00	2
WT	B36	Camp	100	9.00	1
F3065L	B36	Camp	200	12.00	1

Variant[a]	Batch ID[b]	Drug[c]	Concentration[d]	Percent Survived[e]	Clone ID[f]
F3065L	B36	Camp	200	2.00	2
WT	B36	Camp	200	3.00	1
F3065L	B36	MMC	5	100.00	1
F3065L	B36	MMC	5	100.00	2
WT	B36	MMC	5	100.00	1
F3065L	B36	MMC	10	99.00	1
F3065L	B36	MMC	10	90.00	2
WT	B36	MMC	10	88.00	1
F3065L	B36	MMC	20	94.00	1
F3065L	B36	MMC	20	79.00	2
WT	B36	MMC	20	92.00	1
F3065L	B36	MMC	40	70.00	1
F3065L	B36	MMC	40	41.00	2
WT	B36	MMC	40	53.00	1
F3065L	B36	MMC	60	37.00	1
F3065L	B36	MMC	60	18.00	2
WT	B36	MMC	60	28.00	1
F3065L	B36	MMC	80	17.00	1
F3065L	B36	MMC	80	7.00	2
WT	B36	MMC	80	15.00	1
F3065L	B36	Cisp	0.2	43.00	1
F3065L	B36	Cisp	0.2	47.00	2
WT	B36	Cisp	0.2	52.00	1
F3065L	B36	Cisp	0.4	21.00	1
F3065L	B36	Cisp	0.4	24.00	2
WT	B36	Cisp	0.4	32.00	1
F3065L	B36	Cisp	0.6	16.00	1
F3065L	B36	Cisp	0.6	14.00	2
WT	B36	Cisp	0.6	18.00	1
F3065L	B36	Cisp	1	10.00	1
F3065L	B36	Cisp	1	8.00	2
WT	B36	Cisp	1	7.00	1
F3065L	B36	Cisp	1.2	7.00	1
F3065L	B36	Cisp	1.2	6.00	2
WT	B36	Cisp	1.2	7.00	1
F3065L	B36	Cisp	1.5	7.00	1
F3065L	B36	Cisp	1.5	6.00	2
WT	B36	Cisp	1.5	7.00	1
F3065L	B36	MMS	5	97.00	1
F3065L	B36	MMS	5	92.00	2

Variant[a]	Batch ID[b]	Drug[c]	Concentration[d]	Percent Survived[e]	Clone ID[f]
WT	B36	MMS	5	83.00	1
F3065L	B36	MMS	10	81.00	1
F3065L	B36	MMS	10	76.00	2
WT	B36	MMS	10	82.00	1
F3065L	B36	MMS	15	38.00	1
F3065L	B36	MMS	15	52.00	2
WT	B36	MMS	15	73.00	1
F3065L	B36	MMS	20	19.00	1
F3065L	B36	MMS	20	18.00	2
WT	B36	MMS	20	52.00	1
F3065L	B36	MMS	30	5.00	1
F3065L	B36	MMS	30	6.00	2
WT	B36	MMS	30	20.00	1
F3065L	B36	MMS	40	5.00	1
F3065L	B36	MMS	40	4.00	2
WT	B36	MMS	40	4.00	1
F3065L	B36	Parp	0.01	100.00	1
F3065L	B36	Parp	0.01	100.00	2
WT	B36	Parp	0.01	99.00	1
F3065L	B36	Parp	0.1	85.00	1
F3065L	B36	Parp	0.1	78.00	2
WT	B36	Parp	0.1	99.00	1
F3065L	B36	Parp	1	85.00	1
F3065L	B36	Parp	1	53.00	2
WT	B36	Parp	1	85.00	1
F3065L	B36	Parp	10	7.00	1
F3065L	B36	Parp	10	4.00	2
WT	B36	Parp	10	16.00	1
F3065L	B36	IR	50	78.00	1
F3065L	B36	IR	50	62.00	2
WT	B36	IR	50	68.00	1
F3065L	B36	IR	100	74.00	1
F3065L	B36	IR	100	53.00	2
WT	B36	IR	100	64.00	1
F3065L	B36	IR	200	31.00	1
F3065L	B36	IR	200	25.00	2
WT	B36	IR	200	38.00	1
F3065L	B36	IR	400	23.00	1
F3065L	B36	IR	400	15.00	2
WT	B36	IR	400	21.00	1

Variant[a]	Batch ID[b]	Drug[c]	Concentration[d]	Percent Survived[e]	Clone ID[f]
F3065L	B36	IR	600	23.00	1
F3065L	B36	IR	600	12.00	2
WT	B36	IR	600	11.00	1
Y3308X	B37	IR	50	87.35	2
WT	B37	IR	50	88.91	1
Y3308X	B37	IR	100	68.11	2
WT	B37	IR	100	83.75	1
Y3308X	B37	IR	200	27.11	2
WT	B37	IR	200	67.42	1
Y3308X	B37	IR	400	12.18	2
WT	B37	IR	400	31.96	1
Y3308X	B37	IR	600	10.20	2
WT	B37	IR	600	8.52	1
Y3308X	B37	Cisp	0.2	36.98	2
WT	B37	Cisp	0.2	79.79	1
Y3308X	B37	Cisp	0.4	15.83	2
WT	B37	Cisp	0.4	50.94	1
Y3308X	B37	Cisp	0.6	10.61	2
WT	B37	Cisp	0.6	34.70	1
Y3308X	B37	Cisp	1	9.16	2
WT	B37	Cisp	1	13.56	1
Y3308X	B37	Cisp	1.2	8.45	2
WT	B37	Cisp	1.2	9.09	1
Y3308X	B37	Cisp	1.5	8.03	2
WT	B37	Cisp	1.5	5.48	1
Y3308X	B37	MMC	5	68.17	2
WT	B37	MMC	5	89.84	1
Y3308X	B37	MMC	10	36.72	2
WT	B37	MMC	10	80.12	1
Y3308X	B37	MMC	20	15.47	2
WT	B37	MMC	20	51.73	1
Y3308X	B37	MMC	40	10.34	2
WT	B37	MMC	40	21.68	1
Y3308X	B37	MMC	60	8.80	2
WT	B37	MMC	60	9.59	1
Y3308X	B37	MMC	80	9.06	2
WT	B37	MMC	80	4.33	1
Y3308X	B37	MMS	5	82.56	2
WT	B37	MMS	5	100.00	1
Y3308X	B37	MMS	10	41.16	2

Variant[a]	Batch ID[b]	Drug[c]	Concentration[d]	Percent Survived[e]	Clone ID[f]
WT	B37	MMS	10	100.00	1
Y3308X	B37	MMS	15	16.49	2
WT	B37	MMS	15	89.93	1
Y3308X	B37	MMS	20	8.14	2
WT	B37	MMS	20	62.44	1
Y3308X	B37	MMS	30	5.64	2
WT	B37	MMS	30	20.20	1
Y3308X	B37	MMS	40	4.71	2
WT	B37	MMS	40	4.39	1
T598A	B38	Camp	2.5	100.00	1
T598A	B38	Camp	2.5	100.00	2
WT	B38	Camp	2.5	100.00	1
T598A	B38	Camp	5	99.78	1
T598A	B38	Camp	5	100.00	2
WT	B38	Camp	5	100.00	1
T598A	B38	Camp	25	80.82	1
T598A	B38	Camp	25	100.00	2
WT	B38	Camp	25	87.01	1
T598A	B38	Camp	50	43.68	1
T598A	B38	Camp	50	64.06	2
WT	B38	Camp	50	34.26	1
T598A	B38	Camp	100	14.08	1
T598A	B38	Camp	100	17.21	2
WT	B38	Camp	100	5.67	1
T598A	B38	Camp	200	8.09	1
T598A	B38	Camp	200	3.44	2
WT	B38	Camp	200	1.31	1
T598A	B38	MMC	5	100.00	1
T598A	B38	MMC	5	100.00	2
WT	B38	MMC	5	94.13	1
T598A	B38	MMC	10	93.96	1
T598A	B38	MMC	10	100.00	2
WT	B38	MMC	10	90.04	1
T598A	B38	MMC	20	92.70	1
T598A	B38	MMC	20	92.06	2
WT	B38	MMC	20	77.36	1
T598A	B38	MMC	40	58.36	1
T598A	B38	MMC	40	57.78	2
WT	B38	MMC	40	52.52	1
T598A	B38	MMC	60	35.72	1

Variant[a]	Batch ID[b]	Drug[c]	Concentration[d]	Percent Survived[e]	Clone ID[f]
T598A	B38	MMC	60	29.29	2
WT	B38	MMC	60	23.27	1
T598A	B38	MMC	80	22.39	1
T598A	B38	MMC	80	21.35	2
WT	B38	MMC	80	17.09	1
T598A	B38	Cisp	0.2	47.97	1
T598A	B38	Cisp	0.2	49.93	2
WT	B38	Cisp	0.2	45.49	1
T598A	B38	Cisp	0.4	40.09	1
T598A	B38	Cisp	0.4	49.19	2
WT	B38	Cisp	0.4	32.54	1
T598A	B38	Cisp	0.6	26.55	1
T598A	B38	Cisp	0.6	33.38	2
WT	B38	Cisp	0.6	18.50	1
T598A	B38	Cisp	1	15.35	1
T598A	B38	Cisp	1	15.44	2
WT	B38	Cisp	1	9.10	1
T598A	B38	Cisp	1.2	11.62	1
T598A	B38	Cisp	1.2	10.59	2
WT	B38	Cisp	1.2	6.63	1
T598A	B38	Cisp	1.5	7.25	1
T598A	B38	Cisp	1.5	7.06	2
WT	B38	Cisp	1.5	3.78	1
T598A	B38	MMS	5	92.32	1
T598A	B38	MMS	5	100.00	2
WT	B38	MMS	5	86.00	1
T598A	B38	MMS	10	80.23	1
T598A	B38	MMS	10	100.00	2
WT	B38	MMS	10	75.64	1
T598A	B38	MMS	15	65.19	1
T598A	B38	MMS	15	96.78	2
WT	B38	MMS	15	71.30	1
T598A	B38	MMS	20	50.26	1
T598A	B38	MMS	20	78.55	2
WT	B38	MMS	20	57.50	1
T598A	B38	MMS	30	18.09	1
T598A	B38	MMS	30	24.42	2
WT	B38	MMS	30	18.15	1
T598A	B38	MMS	40	7.47	1
T598A	B38	MMS	40	6.27	2

Variant[a]	Batch ID[b]	Drug[c]	Concentration[d]	Percent Survived[e]	Clone ID[f]
WT	B38	MMS	40	6.21	1
T598A	B38	Parp	0.01	72.51	1
T598A	B38	Parp	0.01	81.83	2
WT	B38	Parp	0.01	98.62	1
T598A	B38	Parp	0.1	27.61	1
T598A	B38	Parp	0.1	20.50	2
WT	B38	Parp	0.1	60.13	1
T598A	B38	Parp	1	7.19	1
T598A	B38	Parp	1	3.42	2
WT	B38	Parp	1	2.44	1
T598A	B38	Parp	10	7.31	1
T598A	B38	Parp	10	4.25	2
WT	B38	Parp	10	2.44	1
T598A	B38	IR	50	84.98	1
T598A	B38	IR	50	89.27	2
WT	B38	IR	50	89.94	1
T598A	B38	IR	100	68.72	1
T598A	B38	IR	100	79.74	2
WT	B38	IR	100	69.72	1
T598A	B38	IR	200	45.96	1
T598A	B38	IR	200	57.17	2
WT	B38	IR	200	52.19	1
T598A	B38	IR	400	29.48	1
T598A	B38	IR	400	31.22	2
WT	B38	IR	400	37.18	1
T598A	B38	IR	600	16.59	1
T598A	B38	IR	600	14.57	2
WT	B38	IR	600	15.50	1
S1479T	B39	Camp	2.5	100.00	1
S1479T	B39	Camp	2.5	100.00	2
WT	B39	Camp	2.5	100.00	1
S1479T	B39	Camp	5	100.00	1
S1479T	B39	Camp	5	100.00	2
WT	B39	Camp	5	100.00	1
S1479T	B39	Camp	25	75.27	1
S1479T	B39	Camp	25	87.43	2
WT	B39	Camp	25	87.01	1
S1479T	B39	Camp	50	30.14	1
S1479T	B39	Camp	50	36.92	2
WT	B39	Camp	50	34.26	1

Variant[a]	Batch ID[b]	Drug[c]	Concentration[d]	Percent Survived[e]	Clone ID[f]
S1479T	B39	Camp	100	6.08	1
S1479T	B39	Camp	100	6.12	2
WT	B39	Camp	100	5.67	1
S1479T	B39	Camp	200	3.11	1
S1479T	B39	Camp	200	1.59	2
WT	B39	Camp	200	1.31	1
S1479T	B39	MMC	5	94.76	1
S1479T	B39	MMC	5	86.53	2
WT	B39	MMC	5	94.13	1
S1479T	B39	MMC	10	86.26	1
S1479T	B39	MMC	10	91.42	2
WT	B39	MMC	10	90.04	1
S1479T	B39	MMC	20	77.23	1
S1479T	B39	MMC	20	76.85	2
WT	B39	MMC	20	77.36	1
S1479T	B39	MMC	40	45.29	1
S1479T	B39	MMC	40	45.30	2
WT	B39	MMC	40	52.52	1
S1479T	B39	MMC	60	23.82	1
S1479T	B39	MMC	60	22.97	2
WT	B39	MMC	60	23.27	1
S1479T	B39	MMC	80	17.93	1
S1479T	B39	MMC	80	15.96	2
WT	B39	MMC	80	17.09	1
S1479T	B39	Cisp	0.2	48.71	1
S1479T	B39	Cisp	0.2	49.03	2
WT	B39	Cisp	0.2	45.49	1
S1479T	B39	Cisp	0.4	42.49	1
S1479T	B39	Cisp	0.4	43.55	2
WT	B39	Cisp	0.4	32.54	1
S1479T	B39	Cisp	0.6	25.03	1
S1479T	B39	Cisp	0.6	24.79	2
WT	B39	Cisp	0.6	18.50	1
S1479T	B39	Cisp	1	12.31	1
S1479T	B39	Cisp	1	10.31	2
WT	B39	Cisp	1	9.10	1
S1479T	B39	Cisp	1.2	9.20	1
S1479T	B39	Cisp	1.2	7.52	2
WT	B39	Cisp	1.2	6.63	1
S1479T	B39	Cisp	1.5	5.68	1

Variant[a]	Batch ID[b]	Drug[c]	Concentration[d]	Percent Survived[e]	Clone ID[f]
S1479T	B39	Cisp	1.5	4.46	2
WT	B39	Cisp	1.5	3.78	1
S1479T	B39	MMS	5	95.92	1
S1479T	B39	MMS	5	98.47	2
WT	B39	MMS	5	100.00	1
S1479T	B39	MMS	10	89.38	1
S1479T	B39	MMS	10	88.40	2
WT	B39	MMS	10	100.00	1
S1479T	B39	MMS	15	68.87	1
S1479T	B39	MMS	15	67.59	2
WT	B39	MMS	15	72.85	1
S1479T	B39	MMS	20	62.89	1
S1479T	B39	MMS	20	59.44	2
WT	B39	MMS	20	62.73	1
S1479T	B39	MMS	30	12.25	1
S1479T	B39	MMS	30	10.74	2
WT	B39	MMS	30	17.35	1
S1479T	B39	MMS	40	6.53	1
S1479T	B39	MMS	40	6.62	2
WT	B39	MMS	40	6.75	1
S1479T	B39	Parp	0.01	52.47	1
S1479T	B39	Parp	0.01	44.88	2
WT	B39	Parp	0.01	80.40	1
S1479T	B39	Parp	0.1	22.38	1
S1479T	B39	Parp	0.1	6.93	2
WT	B39	Parp	0.1	54.36	1
S1479T	B39	Parp	1	1.00	1
S1479T	B39	Parp	1	1.00	2
WT	B39	Parp	1	1.00	1
S1479T	B39	Parp	10	1.00	1
S1479T	B39	Parp	10	1.00	2
WT	B39	Parp	10	1.00	1
S1479T	B39	IR	50	57.85	1
S1479T	B39	IR	50	58.68	2
WT	B39	IR	50	84.04	1
S1479T	B39	IR	100	53.01	1
S1479T	B39	IR	100	35.85	2
WT	B39	IR	100	78.67	1
S1479T	B39	IR	200	14.52	1
S1479T	B39	IR	200	10.42	2

Variant[a]	Batch ID[b]	Drug[c]	Concentration[d]	Percent Survived[e]	Clone ID[f]
WT	B39	IR	200	25.20	1
S1479T	B39	IR	400	6.76	1
S1479T	B39	IR	400	6.68	2
WT	B39	IR	400	6.48	1
S1479T	B39	IR	600	6.18	1
S1479T	B39	IR	600	6.60	2
WT	B39	IR	600	5.85	1
T598I	B40	Camp	2.5	100.00	1
T598I	B40	Camp	2.5	95.30	2
WT	B40	Camp	2.5	100.00	1
T598I	B40	Camp	5	93.69	1
T598I	B40	Camp	5	100.00	2
WT	B40	Camp	5	99.69	1
T598I	B40	Camp	25	84.85	1
T598I	B40	Camp	25	76.75	2
WT	B40	Camp	25	80.49	1
T598I	B40	Camp	50	41.26	1
T598I	B40	Camp	50	41.14	2
WT	B40	Camp	50	46.50	1
T598I	B40	Camp	100	10.10	1
T598I	B40	Camp	100	10.80	2
WT	B40	Camp	100	12.12	1
T598I	B40	Camp	200	2.23	1
T598I	B40	Camp	200	2.47	2
WT	B40	Camp	200	3.46	1
T598I	B40	MMC	5	80.37	1
T598I	B40	MMC	5	71.81	2
WT	B40	MMC	5	87.56	1
T598I	B40	MMC	10	58.69	1
T598I	B40	MMC	10	68.14	2
WT	B40	MMC	10	74.88	1
T598I	B40	MMC	20	51.56	1
T598I	B40	MMC	20	50.26	2
WT	B40	MMC	20	66.67	1
T598I	B40	MMC	40	18.55	1
T598I	B40	MMC	40	16.70	2
WT	B40	MMC	40	33.95	1
T598I	B40	MMC	60	9.47	1
T598I	B40	MMC	60	10.05	2
WT	B40	MMC	60	15.90	1

Variant[a]	Batch ID[b]	Drug[c]	Concentration[d]	Percent Survived[e]	Clone ID[f]
T598I	B40	MMC	80	5.96	1
T598I	B40	MMC	80	6.39	2
WT	B40	MMC	80	7.83	1
T598I	B40	Cisp	0.2	63.01	1
T598I	B40	Cisp	0.2	58.68	2
WT	B40	Cisp	0.2	77.90	1
T598I	B40	Cisp	0.4	28.48	1
T598I	B40	Cisp	0.4	28.54	2
WT	B40	Cisp	0.4	50.75	1
T598I	B40	Cisp	0.6	15.68	1
T598I	B40	Cisp	0.6	10.78	2
WT	B40	Cisp	0.6	35.18	1
T598I	B40	Cisp	1	1.00	1
T598I	B40	Cisp	1	1.00	2
WT	B40	Cisp	1	14.98	1
T598I	B40	Cisp	1.2	4.10	1
T598I	B40	Cisp	1.2	3.89	2
WT	B40	Cisp	1.2	8.03	1
T598I	B40	Cisp	1.5	4.71	1
T598I	B40	Cisp	1.5	5.19	2
WT	B40	Cisp	1.5	5.38	1
T598I	B40	MMS	5	100.00	1
T598I	B40	MMS	5	100.00	2
WT	B40	MMS	5	100.00	1
T598I	B40	MMS	10	87.47	1
T598I	B40	MMS	10	73.22	2
WT	B40	MMS	10	80.68	1
T598I	B40	MMS	15	72.77	1
T598I	B40	MMS	15	64.03	2
WT	B40	MMS	15	79.08	1
T598I	B40	MMS	20	45.06	1
T598I	B40	MMS	20	39.58	2
WT	B40	MMS	20	55.80	1
T598I	B40	MMS	30	7.95	1
T598I	B40	MMS	30	6.75	2
WT	B40	MMS	30	13.01	1
T598I	B40	MMS	40	1.20	1
T598I	B40	MMS	40	1.63	2
WT	B40	MMS	40	4.43	1
T598I	B40	Parp	0.01	66.47	1

Variant[a]	Batch ID[b]	Drug[c]	Concentration[d]	Percent Survived[e]	Clone ID[f]
T598I	B40	Parp	0.01	72.93	2
WT	B40	Parp	0.01	93.12	1
T598I	B40	Parp	0.1	24.21	1
T598I	B40	Parp	0.1	35.71	2
WT	B40	Parp	0.1	54.62	1
T598I	B40	Parp	1	1.00	1
T598I	B40	Parp	1	1.68	2
WT	B40	Parp	1	6.68	1
T598I	B40	Parp	10	1.00	1
T598I	B40	Parp	10	2.65	2
WT	B40	Parp	10	8.94	1
T598I	B40	IR	50	87.50	1
T598I	B40	IR	50	86.16	2
WT	B40	IR	50	96.96	1
T598I	B40	IR	100	78.28	1
T598I	B40	IR	100	72.40	2
WT	B40	IR	100	83.99	1
T598I	B40	IR	200	54.70	1
T598I	B40	IR	200	51.32	2
WT	B40	IR	200	57.73	1
T598I	B40	IR	400	28.10	1
T598I	B40	IR	400	27.76	2
WT	B40	IR	400	41.79	1
T598I	B40	IR	600	14.27	1
T598I	B40	IR	600	12.36	2
WT	B40	IR	600	29.78	1
K169R	B41	Camp	2.5	99.65	1
K169R	B41	Camp	2.5	92.77	2
WT	B41	Camp	2.5	100.00	1
K169R	B41	Camp	5	100.00	1
K169R	B41	Camp	5	100.00	2
WT	B41	Camp	5	96.92	1
K169R	B41	Camp	25	83.26	1
K169R	B41	Camp	25	79.11	2
WT	B41	Camp	25	92.70	1
K169R	B41	Camp	50	59.18	1
K169R	B41	Camp	50	61.65	2
WT	B41	Camp	50	66.74	1
K169R	B41	Camp	100	12.99	1
K169R	B41	Camp	100	16.73	2

Variant[a]	Batch ID[b]	Drug[c]	Concentration[d]	Percent Survived[e]	Clone ID[f]
WT	B41	Camp	100	19.46	1
K169R	B41	Camp	200	1.48	1
K169R	B41	Camp	200	1.39	2
WT	B41	Camp	200	1.93	1
K169R	B41	MMC	5	82.90	1
K169R	B41	MMC	5	84.85	2
WT	B41	MMC	5	100.00	1
K169R	B41	MMC	10	66.93	1
K169R	B41	MMC	10	67.91	2
WT	B41	MMC	10	100.00	1
K169R	B41	MMC	20	44.16	1
K169R	B41	MMC	20	45.11	2
WT	B41	MMC	20	65.29	1
K169R	B41	MMC	40	12.31	1
K169R	B41	MMC	40	15.30	2
WT	B41	MMC	40	39.48	1
K169R	B41	MMC	60	4.78	1
K169R	B41	MMC	60	5.21	2
WT	B41	MMC	60	14.19	1
K169R	B41	MMC	80	3.56	1
K169R	B41	MMC	80	3.74	2
WT	B41	MMC	80	6.15	1
K169R	B41	Cisp	0.2	66.48	1
K169R	B41	Cisp	0.2	61.93	2
WT	B41	Cisp	0.2	78.17	1
K169R	B41	Cisp	0.4	42.12	1
K169R	B41	Cisp	0.4	43.03	2
WT	B41	Cisp	0.4	54.62	1
K169R	B41	Cisp	0.6	29.98	1
K169R	B41	Cisp	0.6	26.78	2
WT	B41	Cisp	0.6	40.90	1
K169R	B41	Cisp	1	12.15	1
K169R	B41	Cisp	1	11.83	2
WT	B41	Cisp	1	14.44	1
K169R	B41	Cisp	1.2	7.45	1
K169R	B41	Cisp	1.2	7.27	2
WT	B41	Cisp	1.2	11.88	1
K169R	B41	Cisp	1.5	5.62	1
K169R	B41	Cisp	1.5	6.53	2
WT	B41	Cisp	1.5	5.76	1

Variant[a]	Batch ID[b]	Drug[c]	Concentration[d]	Percent Survived[e]	Clone ID[f]
K169R	B41	MMS	5	90.89	1
K169R	B41	MMS	5	97.43	2
WT	B41	MMS	5	100.00	1
K169R	B41	MMS	10	84.69	1
K169R	B41	MMS	10	79.75	2
WT	B41	MMS	10	100.00	1
K169R	B41	MMS	15	63.67	1
K169R	B41	MMS	15	70.35	2
WT	B41	MMS	15	79.91	1
K169R	B41	MMS	20	53.77	1
K169R	B41	MMS	20	55.05	2
WT	B41	MMS	20	88.30	1
K169R	B41	MMS	30	11.85	1
K169R	B41	MMS	30	11.41	2
WT	B41	MMS	30	43.27	1
K169R	B41	MMS	40	1.70	1
K169R	B41	MMS	40	2.85	2
WT	B41	MMS	40	3.90	1
K169R	B41	Parp	0.01	75.61	1
K169R	B41	Parp	0.01	68.38	2
WT	B41	Parp	0.01	87.08	1
K169R	B41	Parp	0.1	27.85	1
K169R	B41	Parp	0.1	33.92	2
WT	B41	Parp	0.1	62.50	1
K169R	B41	Parp	1	2.35	1
K169R	B41	Parp	1	3.37	2
WT	B41	Parp	1	5.62	1
K169R	B41	Parp	10	3.45	1
K169R	B41	Parp	10	3.19	2
WT	B41	Parp	10	2.17	1
K169R	B41	IR	50	82.41	1
K169R	B41	IR	50	89.68	2
WT	B41	IR	50	88.13	1
K169R	B41	IR	100	67.83	1
K169R	B41	IR	100	71.98	2
WT	B41	IR	100	76.27	1
K169R	B41	IR	200	63.42	1
K169R	B41	IR	200	62.34	2
WT	B41	IR	200	74.15	1
K169R	B41	IR	400	36.82	1

Variant[a]	Batch ID[b]	Drug[c]	Concentration[d]	Percent Survived[e]	Clone ID[f]
K169R	B41	IR	400	32.97	2
WT	B41	IR	400	43.50	1
K169R	B41	IR	600	19.05	1
K169R	B41	IR	600	21.09	2
WT	B41	IR	600	23.40	1
C3233Wfs	B42	Camp	2.5	85.54	1
C3233Wfs	B42	Camp	2.5	97.15	2
WT	B42	Camp	2.5	89.91	1
C3233Wfs	B42	Camp	5	76.81	1
C3233Wfs	B42	Camp	5	91.55	2
WT	B42	Camp	5	87.41	1
C3233Wfs	B42	Camp	25	20.95	1
C3233Wfs	B42	Camp	25	34.96	2
WT	B42	Camp	25	74.40	1
C3233Wfs	B42	Camp	50	3.29	1
C3233Wfs	B42	Camp	50	6.86	2
WT	B42	Camp	50	61.29	1
C3233Wfs	B42	Camp	100	1.00	1
C3233Wfs	B42	Camp	100	1.00	2
WT	B42	Camp	100	16.21	1
C3233Wfs	B42	Camp	200	1.45	1
C3233Wfs	B42	Camp	200	1.00	2
WT	B42	Camp	200	2.41	1
C3233Wfs	B42	MMC	5	27.50	1
C3233Wfs	B42	MMC	5	52.81	2
WT	B42	MMC	5	73.55	1
C3233Wfs	B42	MMC	10	8.25	1
C3233Wfs	B42	MMC	10	21.04	2
WT	B42	MMC	10	66.47	1
C3233Wfs	B42	MMC	20	1.87	1
C3233Wfs	B42	MMC	20	1.00	2
WT	B42	MMC	20	43.72	1
C3233Wfs	B42	MMC	40	1.08	1
C3233Wfs	B42	MMC	40	1.00	2
WT	B42	MMC	40	15.43	1
C3233Wfs	B42	MMC	60	1.00	1
C3233Wfs	B42	MMC	60	1.00	2
WT	B42	MMC	60	5.38	1
C3233Wfs	B42	MMC	80	1.00	1
C3233Wfs	B42	MMC	80	1.00	2

Variant[a]	Batch ID[b]	Drug[c]	Concentration[d]	Percent Survived[e]	Clone ID[f]
WT	B42	MMC	80	4.42	1
C3233Wfs	B42	Cisp	0.2	10.31	1
C3233Wfs	B42	Cisp	0.2	18.28	2
WT	B42	Cisp	0.2	53.96	1
C3233Wfs	B42	Cisp	0.4	1.67	1
C3233Wfs	B42	Cisp	0.4	3.34	2
WT	B42	Cisp	0.4	25.79	1
C3233Wfs	B42	Cisp	0.6	1.67	1
C3233Wfs	B42	Cisp	0.6	1.00	2
WT	B42	Cisp	0.6	14.56	1
C3233Wfs	B42	Cisp	1	1.11	1
C3233Wfs	B42	Cisp	1	1.00	2
WT	B42	Cisp	1	5.14	1
C3233Wfs	B42	Cisp	1.2	1.00	1
C3233Wfs	B42	Cisp	1.2	1.00	2
WT	B42	Cisp	1.2	2.61	1
C3233Wfs	B42	Cisp	1.5	1.30	1
C3233Wfs	B42	Cisp	1.5	1.00	2
WT	B42	Cisp	1.5	1.50	1
C3233Wfs	B42	MMS	5	65.39	1
C3233Wfs	B42	MMS	5	85.51	2
WT	B42	MMS	5	100.00	1
C3233Wfs	B42	MMS	10	14.55	1
C3233Wfs	B42	MMS	10	43.09	2
WT	B42	MMS	10	88.83	1
C3233Wfs	B42	MMS	15	6.49	1
C3233Wfs	B42	MMS	15	24.73	2
WT	B42	MMS	15	78.70	1
C3233Wfs	B42	MMS	20	2.85	1
C3233Wfs	B42	MMS	20	6.16	2
WT	B42	MMS	20	72.03	1
C3233Wfs	B42	MMS	30	1.00	1
C3233Wfs	B42	MMS	30	1.00	2
WT	B42	MMS	30	13.16	1
C3233Wfs	B42	MMS	40	1.38	1
C3233Wfs	B42	MMS	40	1.00	2
WT	B42	MMS	40	2.25	1
C3233Wfs	B42	Parp	0.01	1.99	1
C3233Wfs	B42	Parp	0.01	18.75	2
WT	B42	Parp	0.01	69.09	1

Variant[a]	Batch ID[b]	Drug[c]	Concentration[d]	Percent Survived[e]	Clone ID[f]
C3233Wfs	B42	Parp	0.1	1.22	1
C3233Wfs	B42	Parp	0.1	1.76	2
WT	B42	Parp	0.1	42.13	1
C3233Wfs	B42	Parp	1	1.88	1
C3233Wfs	B42	Parp	1	1.00	2
WT	B42	Parp	1	1.97	1
C3233Wfs	B42	Parp	10	2.99	1
C3233Wfs	B42	Parp	10	1.00	2
WT	B42	Parp	10	3.84	1
C3233Wfs	B42	IR	50	71.41	1
C3233Wfs	B42	IR	50	94.09	2
WT	B42	IR	50	89.72	1
C3233Wfs	B42	IR	100	53.61	1
C3233Wfs	B42	IR	100	62.17	2
WT	B42	IR	100	78.08	1
C3233Wfs	B42	IR	200	21.46	1
C3233Wfs	B42	IR	200	40.07	2
WT	B42	IR	200	54.70	1
C3233Wfs	B42	IR	400	10.57	1
C3233Wfs	B42	IR	400	9.26	2
WT	B42	IR	400	27.22	1
C3233Wfs	B42	IR	600	9.70	1
C3233Wfs	B42	IR	600	7.78	2
WT	B42	IR	600	17.80	1
L2587F	B43	Camp	2.5	98.32	1
L2587F	B43	Camp	2.5	100.00	2
WT	B43	Camp	2.5	100.00	1
L2587F	B43	Camp	5	100.00	1
L2587F	B43	Camp	5	100.00	2
WT	B43	Camp	5	97.28	1
L2587F	B43	Camp	25	87.01	1
L2587F	B43	Camp	25	97.31	2
WT	B43	Camp	25	96.28	1
L2587F	B43	Camp	50	77.80	1
L2587F	B43	Camp	50	83.28	2
WT	B43	Camp	50	80.04	1
L2587F	B43	Camp	100	35.84	1
L2587F	B43	Camp	100	50.81	2
WT	B43	Camp	100	52.07	1
L2587F	B43	Camp	200	1.87	1

Variant[a]	Batch ID[b]	Drug[c]	Concentration[d]	Percent Survived[e]	Clone ID[f]
L2587F	B43	Camp	200	6.08	2
WT	B43	Camp	200	14.14	1
L2587F	B43	MMC	5	80.21	1
L2587F	B43	MMC	5	73.42	2
WT	B43	MMC	5	80.27	1
L2587F	B43	MMC	10	67.43	1
L2587F	B43	MMC	10	68.06	2
WT	B43	MMC	10	65.97	1
L2587F	B43	MMC	20	41.31	1
L2587F	B43	MMC	20	33.38	2
WT	B43	MMC	20	45.85	1
L2587F	B43	MMC	40	7.29	1
L2587F	B43	MMC	40	5.58	2
WT	B43	MMC	40	20.59	1
L2587F	B43	MMC	60	2.72	1
L2587F	B43	MMC	60	1.49	2
WT	B43	MMC	60	5.10	1
L2587F	B43	MMC	80	1.46	1
L2587F	B43	MMC	80	1.17	2
WT	B43	MMC	80	3.43	1
L2587F	B43	Cisp	0.2	66.56	1
L2587F	B43	Cisp	0.2	76.50	2
WT	B43	Cisp	0.2	67.49	1
L2587F	B43	Cisp	0.4	41.49	1
L2587F	B43	Cisp	0.4	38.22	2
WT	B43	Cisp	0.4	48.92	1
L2587F	B43	Cisp	0.6	21.13	1
L2587F	B43	Cisp	0.6	21.00	2
WT	B43	Cisp	0.6	31.79	1
L2587F	B43	Cisp	1	4.56	1
L2587F	B43	Cisp	1	2.20	2
WT	B43	Cisp	1	11.37	1
L2587F	B43	Cisp	1.2	2.28	1
L2587F	B43	Cisp	1.2	1.00	2
WT	B43	Cisp	1.2	7.51	1
L2587F	B43	Cisp	1.5	1.97	1
L2587F	B43	Cisp	1.5	1.00	2
WT	B43	Cisp	1.5	5.20	1
L2587F	B43	MMS	5	99.65	1
L2587F	B43	MMS	5	100.00	2

Variant[a]	Batch ID[b]	Drug[c]	Concentration[d]	Percent Survived[e]	Clone ID[f]
WT	B43	MMS	5	95.15	1
L2587F	B43	MMS	10	88.24	1
L2587F	B43	MMS	10	93.21	2
WT	B43	MMS	10	83.60	1
L2587F	B43	MMS	15	58.27	1
L2587F	B43	MMS	15	70.26	2
WT	B43	MMS	15	80.44	1
L2587F	B43	MMS	20	21.75	1
L2587F	B43	MMS	20	37.94	2
WT	B43	MMS	20	57.75	1
L2587F	B43	MMS	30	1.00	1
L2587F	B43	MMS	30	1.66	2
WT	B43	MMS	30	6.59	1
L2587F	B43	MMS	40	1.00	1
L2587F	B43	MMS	40	1.00	2
WT	B43	MMS	40	1.73	1
L2587F	B43	Parp	0.01	73.40	1
L2587F	B43	Parp	0.01	93.79	2
WT	B43	Parp	0.01	82.98	1
L2587F	B43	Parp	0.1	38.59	1
L2587F	B43	Parp	0.1	44.12	2
WT	B43	Parp	0.1	48.32	1
L2587F	B43	Parp	1	1.00	1
L2587F	B43	Parp	1	1.00	2
WT	B43	Parp	1	1.00	1
L2587F	B43	Parp	10	1.00	1
L2587F	B43	Parp	10	1.00	2
WT	B43	Parp	10	1.37	1
L2587F	B43	IR	50	87.16	1
L2587F	B43	IR	50	81.99	2
WT	B43	IR	50	80.37	1
L2587F	B43	IR	100	76.37	1
L2587F	B43	IR	100	83.34	2
WT	B43	IR	100	67.18	1
L2587F	B43	IR	200	69.46	1
L2587F	B43	IR	200	64.92	2
WT	B43	IR	200	60.54	1
L2587F	B43	IR	400	40.21	1
L2587F	B43	IR	400	35.86	2
WT	B43	IR	400	41.64	1

Variant[a]	Batch ID[b]	Drug[c]	Concentration[d]	Percent Survived[e]	Clone ID[f]
L2587F	B43	IR	600	25.19	1
L2587F	B43	IR	600	30.93	2
WT	B43	IR	600	30.94	1
N986S	B44	Camp	2.5	96.25	1
N986S	B44	Camp	2.5	100.00	2
WT	B44	Camp	2.5	100.00	1
N986S	B44	Camp	5	97.42	1
N986S	B44	Camp	5	96.63	2
WT	B44	Camp	5	97.28	1
N986S	B44	Camp	25	88.34	1
N986S	B44	Camp	25	77.56	2
WT	B44	Camp	25	96.28	1
N986S	B44	Camp	50	76.12	1
N986S	B44	Camp	50	66.25	2
WT	B44	Camp	50	80.04	1
N986S	B44	Camp	100	50.89	1
N986S	B44	Camp	100	39.80	2
WT	B44	Camp	100	52.07	1
N986S	B44	Camp	200	12.03	1
N986S	B44	Camp	200	3.11	2
WT	B44	Camp	200	14.14	1
N986S	B44	MMC	5	82.22	1
N986S	B44	MMC	5	87.03	2
WT	B44	MMC	5	80.27	1
N986S	B44	MMC	10	50.82	1
N986S	B44	MMC	10	67.01	2
WT	B44	MMC	10	65.97	1
N986S	B44	MMC	20	33.72	1
N986S	B44	MMC	20	42.33	2
WT	B44	MMC	20	45.85	1
N986S	B44	MMC	40	15.70	1
N986S	B44	MMC	40	11.72	2
WT	B44	MMC	40	20.59	1
N986S	B44	MMC	60	2.14	1
N986S	B44	MMC	60	2.58	2
WT	B44	MMC	60	5.10	1
N986S	B44	MMC	80	1.00	1
N986S	B44	MMC	80	1.42	2
WT	B44	MMC	80	3.43	1
N986S	B44	Cisp	0.2	58.71	1

Variant[a]	Batch ID[b]	Drug[c]	Concentration[d]	Percent Survived[e]	Clone ID[f]
N986S	B44	Cisp	0.2	71.83	2
WT	B44	Cisp	0.2	67.49	1
N986S	B44	Cisp	0.4	36.88	1
N986S	B44	Cisp	0.4	43.86	2
WT	B44	Cisp	0.4	48.92	1
N986S	B44	Cisp	0.6	16.21	1
N986S	B44	Cisp	0.6	22.37	2
WT	B44	Cisp	0.6	31.79	1
N986S	B44	Cisp	1	3.76	1
N986S	B44	Cisp	1	5.59	2
WT	B44	Cisp	1	11.37	1
N986S	B44	Cisp	1.2	1.68	1
N986S	B44	Cisp	1.2	2.42	2
WT	B44	Cisp	1.2	7.51	1
N986S	B44	Cisp	1.5	1.27	1
N986S	B44	Cisp	1.5	1.50	2
WT	B44	Cisp	1.5	5.20	1
N986S	B44	MMS	5	98.26	1
N986S	B44	MMS	5	100.00	2
WT	B44	MMS	5	95.15	1
N986S	B44	MMS	10	82.84	1
N986S	B44	MMS	10	89.61	2
WT	B44	MMS	10	83.60	1
N986S	B44	MMS	15	61.28	1
N986S	B44	MMS	15	66.94	2
WT	B44	MMS	15	80.44	1
N986S	B44	MMS	20	35.01	1
N986S	B44	MMS	20	39.81	2
WT	B44	MMS	20	57.75	1
N986S	B44	MMS	30	5.28	1
N986S	B44	MMS	30	3.10	2
WT	B44	MMS	30	6.59	1
N986S	B44	MMS	40	1.10	1
N986S	B44	MMS	40	1.00	2
WT	B44	MMS	40	1.73	1
N986S	B44	Parp	0.01	57.15	1
N986S	B44	Parp	0.01	77.17	2
WT	B44	Parp	0.01	82.98	1
N986S	B44	Parp	0.1	7.83	1
N986S	B44	Parp	0.1	51.69	2

Variant[a]	Batch ID[b]	Drug[c]	Concentration[d]	Percent Survived[e]	Clone ID[f]
WT	B44	Parp	0.1	48.32	1
N986S	B44	Parp	1	1.00	1
N986S	B44	Parp	1	3.25	2
WT	B44	Parp	1	1.00	1
N986S	B44	Parp	10	1.00	1
N986S	B44	Parp	10	1.00	2
WT	B44	Parp	10	1.37	1
N986S	B44	IR	50	100.00	1
N986S	B44	IR	50	92.06	2
WT	B44	IR	50	80.37	1
N986S	B44	IR	100	91.71	1
N986S	B44	IR	100	79.59	2
WT	B44	IR	100	67.18	1
N986S	B44	IR	200	80.45	1
N986S	B44	IR	200	70.11	2
WT	B44	IR	200	60.54	1
N986S	B44	IR	400	35.34	1
N986S	B44	IR	400	33.68	2
WT	B44	IR	400	41.64	1
N986S	B44	IR	600	26.90	1
N986S	B44	IR	600	23.54	2
WT	B44	IR	600	30.94	1
W2788S	B45	Camp	2.5	88.00	1
W2788S	B45	Camp	2.5	79.00	2
WT	B45	Camp	2.5	100.00	1
W2788S	B45	Camp	5	82.00	1
W2788S	B45	Camp	5	71.00	2
WT	B45	Camp	5	100.00	1
W2788S	B45	Camp	25	42.00	1
W2788S	B45	Camp	25	10.00	2
WT	B45	Camp	25	100.00	1
W2788S	B45	Camp	50	13.00	1
W2788S	B45	Camp	50	5.00	2
WT	B45	Camp	50	78.00	1
W2788S	B45	Camp	100	5.00	1
W2788S	B45	Camp	100	4.00	2
WT	B45	Camp	100	22.00	1
W2788S	B45	Camp	200	4.00	1
W2788S	B45	Camp	200	5.00	2
WT	B45	Camp	200	5.00	1

Variant[a]	Batch ID[b]	Drug[c]	Concentration[d]	Percent Survived[e]	Clone ID[f]
W2788S	B45	MMC	5	64.00	1
W2788S	B45	MMC	5	42.00	2
WT	B45	MMC	5	95.00	1
W2788S	B45	MMC	10	45.00	1
W2788S	B45	MMC	10	12.00	2
WT	B45	MMC	10	90.00	1
W2788S	B45	MMC	20	29.00	1
W2788S	B45	MMC	20	5.00	2
WT	B45	MMC	20	84.00	1
W2788S	B45	MMC	40	10.00	1
W2788S	B45	MMC	40	4.00	2
WT	B45	MMC	40	58.00	1
W2788S	B45	MMC	60	7.00	1
W2788S	B45	MMC	60	3.00	2
WT	B45	MMC	60	36.00	1
W2788S	B45	MMC	80	6.00	1
W2788S	B45	MMC	80	4.00	2
WT	B45	MMC	80	17.00	1
W2788S	B45	Cisp	0.2	40.00	1
W2788S	B45	Cisp	0.2	13.00	2
WT	B45	Cisp	0.2	74.00	1
W2788S	B45	Cisp	0.4	13.00	1
W2788S	B45	Cisp	0.4	6.00	2
WT	B45	Cisp	0.4	60.00	1
W2788S	B45	Cisp	0.6	8.00	1
W2788S	B45	Cisp	0.6	5.00	2
WT	B45	Cisp	0.6	43.00	1
W2788S	B45	Cisp	1	6.00	1
W2788S	B45	Cisp	1	4.00	2
WT	B45	Cisp	1	16.00	1
W2788S	B45	Cisp	1.2	4.00	1
W2788S	B45	Cisp	1.2	2.00	2
WT	B45	Cisp	1.2	10.00	1
W2788S	B45	Cisp	1.5	5.00	1
W2788S	B45	Cisp	1.5	2.00	2
WT	B45	Cisp	1.5	6.00	1
W2788S	B45	MMS	5	87.00	1
W2788S	B45	MMS	5	54.00	2
WT	B45	MMS	5	100.00	1
W2788S	B45	MMS	10	46.00	1

Variant[a]	Batch ID[b]	Drug[c]	Concentration[d]	Percent Survived[e]	Clone ID[f]
W2788S	B45	MMS	10	11.00	2
WT	B45	MMS	10	100.00	1
W2788S	B45	MMS	15	16.00	1
W2788S	B45	MMS	15	5.00	2
WT	B45	MMS	15	100.00	1
W2788S	B45	MMS	20	6.00	1
W2788S	B45	MMS	20	3.00	2
WT	B45	MMS	20	84.00	1
W2788S	B45	MMS	30	1.00	1
W2788S	B45	MMS	30	1.00	2
WT	B45	MMS	30	26.00	1
W2788S	B45	MMS	40	2.00	1
W2788S	B45	MMS	40	1.00	2
WT	B45	MMS	40	8.00	1
W2788S	B45	Parp	0.01	66.00	1
W2788S	B45	Parp	0.01	38.00	2
WT	B45	Parp	0.01	100.00	1
W2788S	B45	Parp	0.1	16.00	1
W2788S	B45	Parp	0.1	7.00	2
WT	B45	Parp	0.1	89.00	1
W2788S	B45	Parp	1	6.00	1
W2788S	B45	Parp	1	6.00	2
WT	B45	Parp	1	71.00	1
W2788S	B45	Parp	10	5.00	1
W2788S	B45	Parp	10	4.00	2
WT	B45	Parp	10	6.00	1
W2788S	B45	IR	50	83.47	1
W2788S	B45	IR	50	69.32	2
WT	B45	IR	50	80.51	1
W2788S	B45	IR	100	71.14	1
W2788S	B45	IR	100	74.67	2
WT	B45	IR	100	73.32	1
W2788S	B45	IR	200	54.37	1
W2788S	B45	IR	200	55.05	2
WT	B45	IR	200	78.57	1
W2788S	B45	IR	400	47.78	1
W2788S	B45	IR	400	22.59	2
WT	B45	IR	400	53.73	1
W2788S	B45	IR	600	21.80	1
W2788S	B45	IR	600	18.67	2

Variant[a]	Batch ID[b]	Drug[c]	Concentration[d]	Percent Survived[e]	Clone ID[f]
WT	B45	IR	600	38.97	1
R2784W	B46	Camp	2.5	100.00	1
R2784W	B46	Camp	2.5	100.00	2
WT	B46	Camp	2.5	100.00	1
R2784W	B46	Camp	5	99.00	1
R2784W	B46	Camp	5	100.00	2
WT	B46	Camp	5	100.00	1
R2784W	B46	Camp	25	28.00	1
R2784W	B46	Camp	25	65.00	2
WT	B46	Camp	25	100.00	1
R2784W	B46	Camp	50	7.00	1
R2784W	B46	Camp	50	19.00	2
WT	B46	Camp	50	78.00	1
R2784W	B46	Camp	100	2.00	1
R2784W	B46	Camp	100	3.00	2
WT	B46	Camp	100	22.00	1
R2784W	B46	Camp	200	3.00	1
R2784W	B46	Camp	200	1.00	2
WT	B46	Camp	200	5.00	1
R2784W	B46	MMC	5	70.00	1
R2784W	B46	MMC	5	68.00	2
WT	B46	MMC	5	95.00	1
R2784W	B46	MMC	10	42.00	1
R2784W	B46	MMC	10	50.00	2
WT	B46	MMC	10	90.00	1
R2784W	B46	MMC	20	19.00	1
R2784W	B46	MMC	20	25.00	2
WT	B46	MMC	20	84.00	1
R2784W	B46	MMC	40	7.00	1
R2784W	B46	MMC	40	7.00	2
WT	B46	MMC	40	58.00	1
R2784W	B46	MMC	60	5.00	1
R2784W	B46	MMC	60	3.00	2
WT	B46	MMC	60	36.00	1
R2784W	B46	MMC	80	4.00	1
R2784W	B46	MMC	80	3.00	2
WT	B46	MMC	80	17.00	1
R2784W	B46	Cisp	0.2	18.00	1
R2784W	B46	Cisp	0.2	51.00	2
WT	B46	Cisp	0.2	74.00	1

Variant[a]	Batch ID[b]	Drug[c]	Concentration[d]	Percent Survived[e]	Clone ID[f]
R2784W	B46	Cisp	0.4	8.00	1
R2784W	B46	Cisp	0.4	16.00	2
WT	B46	Cisp	0.4	60.00	1
R2784W	B46	Cisp	0.6	6.00	1
R2784W	B46	Cisp	0.6	9.00	2
WT	B46	Cisp	0.6	43.00	1
R2784W	B46	Cisp	1	4.00	1
R2784W	B46	Cisp	1	4.00	2
WT	B46	Cisp	1	16.00	1
R2784W	B46	Cisp	1.2	3.00	1
R2784W	B46	Cisp	1.2	2.00	2
WT	B46	Cisp	1.2	10.00	1
R2784W	B46	Cisp	1.5	3.00	1
R2784W	B46	Cisp	1.5	2.00	2
WT	B46	Cisp	1.5	6.00	1
R2784W	B46	MMS	5	58.00	1
R2784W	B46	MMS	5	100.00	2
WT	B46	MMS	5	100.00	1
R2784W	B46	MMS	10	16.00	1
R2784W	B46	MMS	10	100.00	2
WT	B46	MMS	10	100.00	1
R2784W	B46	MMS	15	6.00	1
R2784W	B46	MMS	15	40.00	2
WT	B46	MMS	15	100.00	1
R2784W	B46	MMS	20	3.00	1
R2784W	B46	MMS	20	11.00	2
WT	B46	MMS	20	84.00	1
R2784W	B46	MMS	30	1.00	1
R2784W	B46	MMS	30	1.00	2
WT	B46	MMS	30	26.00	1
R2784W	B46	MMS	40	2.00	1
R2784W	B46	MMS	40	2.00	2
WT	B46	MMS	40	8.00	1
R2784W	B46	Parp	0.01	61.00	1
R2784W	B46	Parp	0.01	85.00	2
WT	B46	Parp	0.01	100.00	1
R2784W	B46	Parp	0.1	19.00	1
R2784W	B46	Parp	0.1	19.00	2
WT	B46	Parp	0.1	89.00	1
R2784W	B46	Parp	1	7.00	1

Variant[a]	Batch ID[b]	Drug[c]	Concentration[d]	Percent Survived[e]	Clone ID[f]
R2784W	B46	Parp	1	7.00	2
WT	B46	Parp	1	71.00	1
R2784W	B46	Parp	10	6.00	1
R2784W	B46	Parp	10	6.00	2
WT	B46	Parp	10	6.00	1
R2784W	B46	IR	50	74.90	1
R2784W	B46	IR	50	68.45	2
WT	B46	IR	50	80.51	1
R2784W	B46	IR	100	62.40	1
R2784W	B46	IR	100	64.00	2
WT	B46	IR	100	73.32	1
R2784W	B46	IR	200	57.12	1
R2784W	B46	IR	200	50.17	2
WT	B46	IR	200	78.57	1
R2784W	B46	IR	400	29.41	1
R2784W	B46	IR	400	26.01	2
WT	B46	IR	400	53.73	1
R2784W	B46	IR	600	29.41	1
R2784W	B46	IR	600	16.50	2
WT	B46	IR	600	38.97	1
T1624A	B47	Camp	2.5	100.00	1
T1624A	B47	Camp	2.5	100.00	2
WT	B47	Camp	2.5	100.00	1
T1624A	B47	Camp	5	98.26	1
T1624A	B47	Camp	5	94.98	2
WT	B47	Camp	5	100.00	1
T1624A	B47	Camp	25	69.37	1
T1624A	B47	Camp	25	79.45	2
WT	B47	Camp	25	97.14	1
T1624A	B47	Camp	50	31.88	1
T1624A	B47	Camp	50	29.79	2
WT	B47	Camp	50	60.48	1
T1624A	B47	Camp	100	7.34	1
T1624A	B47	Camp	100	6.96	2
WT	B47	Camp	100	11.75	1
T1624A	B47	Camp	200	3.86	1
T1624A	B47	Camp	200	4.00	2
WT	B47	Camp	200	5.08	1
T1624A	B47	MMC	5	90.57	1
T1624A	B47	MMC	5	80.81	2

Variant[a]	Batch ID[b]	Drug[c]	Concentration[d]	Percent Survived[e]	Clone ID[f]
WT	B47	MMC	5	97.81	1
T1624A	B47	MMC	10	72.36	1
T1624A	B47	MMC	10	75.76	2
WT	B47	MMC	10	100.00	1
T1624A	B47	MMC	20	48.83	1
T1624A	B47	MMC	20	51.40	2
WT	B47	MMC	20	89.83	1
T1624A	B47	MMC	40	18.42	1
T1624A	B47	MMC	40	24.24	2
WT	B47	MMC	40	57.80	1
T1624A	B47	MMC	60	8.77	1
T1624A	B47	MMC	60	14.25	2
WT	B47	MMC	60	28.94	1
T1624A	B47	MMC	80	6.44	1
T1624A	B47	MMC	80	8.08	2
WT	B47	MMC	80	17.33	1
T1624A	B47	Cisp	0.2	70.78	1
T1624A	B47	Cisp	0.2	71.25	2
WT	B47	Cisp	0.2	92.48	1
T1624A	B47	Cisp	0.4	35.13	1
T1624A	B47	Cisp	0.4	42.01	2
WT	B47	Cisp	0.4	77.35	1
T1624A	B47	Cisp	0.6	17.51	1
T1624A	B47	Cisp	0.6	21.69	2
WT	B47	Cisp	0.6	45.67	1
T1624A	B47	Cisp	1	7.56	1
T1624A	B47	Cisp	1	11.40	2
WT	B47	Cisp	1	26.94	1
T1624A	B47	Cisp	1.2	5.39	1
T1624A	B47	Cisp	1.2	6.57	2
WT	B47	Cisp	1.2	13.02	1
T1624A	B47	Cisp	1.5	4.87	1
T1624A	B47	Cisp	1.5	7.56	2
WT	B47	Cisp	1.5	6.77	1
T1624A	B47	MMS	5	85.10	1
T1624A	B47	MMS	5	77.82	2
WT	B47	MMS	5	100.00	1
T1624A	B47	MMS	10	59.79	1
T1624A	B47	MMS	10	42.94	2
WT	B47	MMS	10	100.00	1

Variant[a]	Batch ID[b]	Drug[c]	Concentration[d]	Percent Survived[e]	Clone ID[f]
T1624A	B47	MMS	15	32.54	1
T1624A	B47	MMS	15	27.22	2
WT	B47	MMS	15	84.68	1
T1624A	B47	MMS	20	13.23	1
T1624A	B47	MMS	20	15.83	2
WT	B47	MMS	20	61.26	1
T1624A	B47	MMS	30	2.56	1
T1624A	B47	MMS	30	2.62	2
WT	B47	MMS	30	15.32	1
T1624A	B47	MMS	40	1.76	1
T1624A	B47	MMS	40	2.12	2
WT	B47	MMS	40	2.30	1
T1624A	B47	Parp	0.01	88.48	1
T1624A	B47	Parp	0.01	96.93	2
WT	B47	Parp	0.01	100.00	1
T1624A	B47	Parp	0.1	58.07	1
T1624A	B47	Parp	0.1	62.37	2
WT	B47	Parp	0.1	100.00	1
T1624A	B47	Parp	1	10.83	1
T1624A	B47	Parp	1	15.75	2
WT	B47	Parp	1	64.48	1
T1624A	B47	Parp	10	1.77	1
T1624A	B47	Parp	10	3.38	2
WT	B47	Parp	10	5.02	1
T1624A	B47	IR	50	79.11	1
T1624A	B47	IR	50	70.39	2
WT	B47	IR	50	86.71	1
T1624A	B47	IR	100	68.63	1
T1624A	B47	IR	100	63.05	2
WT	B47	IR	100	80.26	1
T1624A	B47	IR	200	54.33	1
T1624A	B47	IR	200	49.21	2
WT	B47	IR	200	59.28	1
T1624A	B47	IR	400	26.69	1
T1624A	B47	IR	400	29.69	2
WT	B47	IR	400	46.77	1
T1624A	B47	IR	600	18.35	1
T1624A	B47	IR	600	18.18	2
WT	B47	IR	600	33.63	1
L452V	B48	Camp	2.5	97.98	1

Variant[a]	Batch ID[b]	Drug[c]	Concentration[d]	Percent Survived[e]	Clone ID[f]
L452V	B48	Camp	2.5	100.00	2
WT	B48	Camp	2.5	83.11	1
L452V	B48	Camp	5	100.00	1
L452V	B48	Camp	5	100.00	2
WT	B48	Camp	5	99.29	1
L452V	B48	Camp	25	97.23	1
L452V	B48	Camp	25	94.59	2
WT	B48	Camp	25	92.74	1
L452V	B48	Camp	50	70.21	1
L452V	B48	Camp	50	57.09	2
WT	B48	Camp	50	69.85	1
L452V	B48	Camp	100	17.34	1
L452V	B48	Camp	100	3.72	2
WT	B48	Camp	100	21.07	1
L452V	B48	Camp	200	1.81	1
L452V	B48	Camp	200	1.69	2
WT	B48	Camp	200	3.79	1
L452V	B48	MMC	5	88.72	1
L452V	B48	MMC	5	68.68	2
WT	B48	MMC	5	82.15	1
L452V	B48	MMC	10	78.68	1
L452V	B48	MMC	10	59.95	2
WT	B48	MMC	10	75.74	1
L452V	B48	MMC	20	53.92	1
L452V	B48	MMC	20	38.25	2
WT	B48	MMC	20	60.53	1
L452V	B48	MMC	40	28.37	1
L452V	B48	MMC	40	14.04	2
WT	B48	MMC	40	29.70	1
L452V	B48	MMC	60	14.89	1
L452V	B48	MMC	60	3.69	2
WT	B48	MMC	60	16.01	1
L452V	B48	MMC	80	6.08	1
L452V	B48	MMC	80	1.71	2
WT	B48	MMC	80	5.76	1
L452V	B48	Cisp	0.2	61.39	1
L452V	B48	Cisp	0.2	57.04	2
WT	B48	Cisp	0.2	65.70	1
L452V	B48	Cisp	0.4	32.57	1
L452V	B48	Cisp	0.4	31.44	2

Variant[a]	Batch ID[b]	Drug[c]	Concentration[d]	Percent Survived[e]	Clone ID[f]
WT	B48	Cisp	0.4	40.68	1
L452V	B48	Cisp	0.6	16.01	1
L452V	B48	Cisp	0.6	16.00	2
WT	B48	Cisp	0.6	27.69	1
L452V	B48	Cisp	1	4.85	1
L452V	B48	Cisp	1	4.32	2
WT	B48	Cisp	1	6.24	1
L452V	B48	Cisp	1.2	2.56	1
L452V	B48	Cisp	1.2	2.48	2
WT	B48	Cisp	1.2	4.90	1
L452V	B48	Cisp	1.5	2.10	1
L452V	B48	Cisp	1.5	1.92	2
WT	B48	Cisp	1.5	4.83	1
L452V	B48	MMS	5	100.00	1
L452V	B48	MMS	5	99.42	2
WT	B48	MMS	5	100.00	1
L452V	B48	MMS	10	99.49	1
L452V	B48	MMS	10	85.60	2
WT	B48	MMS	10	100.00	1
L452V	B48	MMS	15	78.04	1
L452V	B48	MMS	15	59.71	2
WT	B48	MMS	15	72.85	1
L452V	B48	MMS	20	77.32	1
L452V	B48	MMS	20	59.42	2
WT	B48	MMS	20	62.73	1
L452V	B48	MMS	30	15.93	1
L452V	B48	MMS	30	3.38	2
WT	B48	MMS	30	17.35	1
L452V	B48	MMS	40	8.17	1
L452V	B48	MMS	40	2.13	2
WT	B48	MMS	40	6.75	1
L452V	B48	Parp	0.01	55.07	1
L452V	B48	Parp	0.01	54.23	2
WT	B48	Parp	0.01	80.40	1
L452V	B48	Parp	0.1	20.66	1
L452V	B48	Parp	0.1	16.22	2
WT	B48	Parp	0.1	54.36	1
L452V	B48	Parp	1	1.00	1
L452V	B48	Parp	1	1.00	2
WT	B48	Parp	1	1.00	1

Variant[a]	Batch ID[b]	Drug[c]	Concentration[d]	Percent Survived[e]	Clone ID[f]
L452V	B48	Parp	10	1.00	1
L452V	B48	Parp	10	1.00	2
WT	B48	Parp	10	1.00	1
L452V	B48	IR	50	64.94	1
L452V	B48	IR	50	58.97	2
WT	B48	IR	50	84.04	1
L452V	B48	IR	100	39.86	1
L452V	B48	IR	100	23.81	2
WT	B48	IR	100	78.67	1
L452V	B48	IR	200	13.72	1
L452V	B48	IR	200	7.06	2
WT	B48	IR	200	25.20	1
L452V	B48	IR	400	6.55	1
L452V	B48	IR	400	5.67	2
WT	B48	IR	400	6.48	1
L452V	B48	IR	600	5.79	1
L452V	B48	IR	600	5.08	2
WT	B48	IR	600	5.85	1
N986I	B49	Camp	2.5	100.00	1
N986I	B49	Camp	2.5	100.00	2
WT	B49	Camp	2.5	100.00	1
N986I	B49	Camp	5	100.00	1
N986I	B49	Camp	5	97.57	2
WT	B49	Camp	5	100.00	1
N986I	B49	Camp	25	75.66	1
N986I	B49	Camp	25	83.83	2
WT	B49	Camp	25	89.36	1
N986I	B49	Camp	50	59.72	1
N986I	B49	Camp	50	61.70	2
WT	B49	Camp	50	66.90	1
N986I	B49	Camp	100	10.81	1
N986I	B49	Camp	100	13.01	2
WT	B49	Camp	100	14.74	1
N986I	B49	Camp	200	6.44	1
N986I	B49	Camp	200	2.04	2
WT	B49	Camp	200	3.55	1
N986I	B49	MMC	5	95.74	1
N986I	B49	MMC	5	91.55	2
WT	B49	MMC	5	100.00	1
N986I	B49	MMC	10	100.00	1

Variant[a]	Batch ID[b]	Drug[c]	Concentration[d]	Percent Survived[e]	Clone ID[f]
N986I	B49	MMC	10	100.00	2
WT	B49	MMC	10	100.00	1
N986I	B49	MMC	20	100.00	1
N986I	B49	MMC	20	100.00	2
WT	B49	MMC	20	96.73	1
N986I	B49	MMC	40	64.04	1
N986I	B49	MMC	40	48.59	2
WT	B49	MMC	40	55.16	1
N986I	B49	MMC	60	22.30	1
N986I	B49	MMC	60	16.50	2
WT	B49	MMC	60	24.19	1
N986I	B49	MMC	80	9.57	1
N986I	B49	MMC	80	5.41	2
WT	B49	MMC	80	13.51	1
N986I	B49	Cisp	0.2	69.25	1
N986I	B49	Cisp	0.2	67.69	2
WT	B49	Cisp	0.2	81.42	1
N986I	B49	Cisp	0.4	51.15	1
N986I	B49	Cisp	0.4	49.74	2
WT	B49	Cisp	0.4	66.27	1
N986I	B49	Cisp	0.6	35.17	1
N986I	B49	Cisp	0.6	27.33	2
WT	B49	Cisp	0.6	56.26	1
N986I	B49	Cisp	1	17.14	1
N986I	B49	Cisp	1	5.35	2
WT	B49	Cisp	1	28.47	1
N986I	B49	Cisp	1.2	10.04	1
N986I	B49	Cisp	1.2	3.79	2
WT	B49	Cisp	1.2	24.52	1
N986I	B49	Cisp	1.5	4.86	1
N986I	B49	Cisp	1.5	1.18	2
WT	B49	Cisp	1.5	12.64	1
N986I	B49	MMS	5	93.36	1
N986I	B49	MMS	5	100.00	2
WT	B49	MMS	5	100.00	1
N986I	B49	MMS	10	97.63	1
N986I	B49	MMS	10	100.00	2
WT	B49	MMS	10	100.00	1
N986I	B49	MMS	15	76.51	1
N986I	B49	MMS	15	100.00	2

Variant[a]	Batch ID[b]	Drug[c]	Concentration[d]	Percent Survived[e]	Clone ID[f]
WT	B49	MMS	15	100.00	1
N986I	B49	MMS	20	55.57	1
N986I	B49	MMS	20	77.52	2
WT	B49	MMS	20	100.00	1
N986I	B49	MMS	30	23.66	1
N986I	B49	MMS	30	28.33	2
WT	B49	MMS	30	49.08	1
N986I	B49	MMS	40	5.77	1
N986I	B49	MMS	40	4.11	2
WT	B49	MMS	40	9.98	1
N986I	B49	Parp	0.01	100.00	1
N986I	B49	Parp	0.01	94.45	2
WT	B49	Parp	0.01	90.17	1
N986I	B49	Parp	0.1	89.11	1
N986I	B49	Parp	0.1	89.63	2
WT	B49	Parp	0.1	94.48	1
N986I	B49	Parp	1	75.06	1
N986I	B49	Parp	1	65.41	2
WT	B49	Parp	1	72.05	1
N986I	B49	Parp	10	15.28	1
N986I	B49	Parp	10	1.29	2
WT	B49	Parp	10	16.55	1
N986I	B49	IR	50	86.85	1
N986I	B49	IR	50	70.64	2
WT	B49	IR	50	73.53	1
N986I	B49	IR	100	98.34	1
N986I	B49	IR	100	59.84	2
WT	B49	IR	100	52.31	1
N986I	B49	IR	200	61.82	1
N986I	B49	IR	200	57.96	2
WT	B49	IR	200	56.31	1
N986I	B49	IR	400	35.67	1
N986I	B49	IR	400	38.04	2
WT	B49	IR	400	35.24	1
N986I	B49	IR	600	6.02	1
N986I	B49	IR	600	5.32	2
WT	B49	IR	600	8.71	1
V2728I	B50	Camp	2.5	98.54	1
V2728I	B50	Camp	2.5	95.83	2
WT	B50	Camp	2.5	100.00	1

Variant[a]	Batch ID[b]	Drug[c]	Concentration[d]	Percent Survived[e]	Clone ID[f]
V2728I	B50	Camp	5	100.00	1
V2728I	B50	Camp	5	100.00	2
WT	B50	Camp	5	100.00	1
V2728I	B50	Camp	25	79.35	1
V2728I	B50	Camp	25	79.66	2
WT	B50	Camp	25	92.11	1
V2728I	B50	Camp	50	53.30	1
V2728I	B50	Camp	50	55.65	2
WT	B50	Camp	50	75.32	1
V2728I	B50	Camp	100	14.48	1
V2728I	B50	Camp	100	9.89	2
WT	B50	Camp	100	19.47	1
V2728I	B50	Camp	200	1.00	1
V2728I	B50	Camp	200	1.06	2
WT	B50	Camp	200	1.00	1
V2728I	B50	MMC	5	91.40	1
V2728I	B50	MMC	5	94.63	2
WT	B50	MMC	5	100.00	1
V2728I	B50	MMC	10	97.95	1
V2728I	B50	MMC	10	98.36	2
WT	B50	MMC	10	96.55	1
V2728I	B50	MMC	20	86.16	1
V2728I	B50	MMC	20	84.23	2
WT	B50	MMC	20	93.34	1
V2728I	B50	MMC	40	61.51	1
V2728I	B50	MMC	40	69.83	2
WT	B50	MMC	40	66.46	1
V2728I	B50	MMC	60	26.13	1
V2728I	B50	MMC	60	37.57	2
WT	B50	MMC	60	40.39	1
V2728I	B50	MMC	80	8.44	1
V2728I	B50	MMC	80	13.68	2
WT	B50	MMC	80	14.28	1
V2728I	B50	Cisp	0.2	64.68	1
V2728I	B50	Cisp	0.2	64.96	2
WT	B50	Cisp	0.2	96.74	1
V2728I	B50	Cisp	0.4	40.58	1
V2728I	B50	Cisp	0.4	40.75	2
WT	B50	Cisp	0.4	79.99	1
V2728I	B50	Cisp	0.6	23.40	1

Variant[a]	Batch ID[b]	Drug[c]	Concentration[d]	Percent Survived[e]	Clone ID[f]
V2728I	B50	Cisp	0.6	23.04	2
WT	B50	Cisp	0.6	60.98	1
V2728I	B50	Cisp	1	7.10	1
V2728I	B50	Cisp	1	3.44	2
WT	B50	Cisp	1	16.86	1
V2728I	B50	Cisp	1.2	4.91	1
V2728I	B50	Cisp	1.2	2.27	2
WT	B50	Cisp	1.2	12.55	1
V2728I	B50	Cisp	1.5	2.98	1
V2728I	B50	Cisp	1.5	2.14	2
WT	B50	Cisp	1.5	6.14	1
V2728I	B50	MMS	5	100.00	1
V2728I	B50	MMS	5	91.01	2
WT	B50	MMS	5	100.00	1
V2728I	B50	MMS	10	94.68	1
V2728I	B50	MMS	10	83.30	2
WT	B50	MMS	10	98.85	1
V2728I	B50	MMS	15	69.83	1
V2728I	B50	MMS	15	74.53	2
WT	B50	MMS	15	100.00	1
V2728I	B50	MMS	20	47.76	1
V2728I	B50	MMS	20	55.36	2
WT	B50	MMS	20	82.46	1
V2728I	B50	MMS	30	10.57	1
V2728I	B50	MMS	30	11.34	2
WT	B50	MMS	30	32.01	1
V2728I	B50	MMS	40	1.08	1
V2728I	B50	MMS	40	1.00	2
WT	B50	MMS	40	2.10	1
V2728I	B50	Parp	0.01	90.26	1
V2728I	B50	Parp	0.01	89.18	2
WT	B50	Parp	0.01	100.00	1
V2728I	B50	Parp	0.1	54.99	1
V2728I	B50	Parp	0.1	65.73	2
WT	B50	Parp	0.1	90.67	1
V2728I	B50	Parp	1	21.95	1
V2728I	B50	Parp	1	13.11	2
WT	B50	Parp	1	79.68	1
V2728I	B50	Parp	10	1.28	1
V2728I	B50	Parp	10	1.00	2

Variant[a]	Batch ID[b]	Drug[c]	Concentration[d]	Percent Survived[e]	Clone ID[f]
WT	B50	Parp	10	2.48	1
V2728I	B50	IR	50	96.74	1
V2728I	B50	IR	50	97.78	2
WT	B50	IR	50	100.00	1
V2728I	B50	IR	100	80.89	1
V2728I	B50	IR	100	87.81	2
WT	B50	IR	100	100.00	1
V2728I	B50	IR	200	21.91	1
V2728I	B50	IR	200	18.78	2
WT	B50	IR	200	98.18	1
V2728I	B50	IR	400	23.15	1
V2728I	B50	IR	400	36.03	2
WT	B50	IR	400	77.98	1
V2728I	B50	IR	600	15.31	1
V2728I	B50	IR	600	25.66	2
WT	B50	IR	600	55.44	1
L2106P	B51	Camp	2.5	100.00	1
L2106P	B51	Camp	2.5	100.00	2
WT	B51	Camp	2.5	100.00	1
L2106P	B51	Camp	5	91.27	1
L2106P	B51	Camp	5	98.59	2
WT	B51	Camp	5	100.00	1
L2106P	B51	Camp	25	72.65	1
L2106P	B51	Camp	25	71.44	2
WT	B51	Camp	25	85.85	1
L2106P	B51	Camp	50	25.71	1
L2106P	B51	Camp	50	44.84	2
WT	B51	Camp	50	58.71	1
L2106P	B51	Camp	100	3.84	1
L2106P	B51	Camp	100	14.66	2
WT	B51	Camp	100	27.88	1
L2106P	B51	Camp	200	1.00	1
L2106P	B51	Camp	200	2.17	2
WT	B51	Camp	200	2.45	1
L2106P	B51	MMC	5	66.80	1
L2106P	B51	MMC	5	38.25	2
WT	B51	MMC	5	78.25	1
L2106P	B51	MMC	10	34.61	1
L2106P	B51	MMC	10	6.72	2
WT	B51	MMC	10	53.63	1

Variant[a]	Batch ID[b]	Drug[c]	Concentration[d]	Percent Survived[e]	Clone ID[f]
L2106P	B51	MMC	20	18.61	1
L2106P	B51	MMC	20	1.00	2
WT	B51	MMC	20	34.47	1
L2106P	B51	MMC	40	1.00	1
L2106P	B51	MMC	40	1.00	2
WT	B51	MMC	40	1.00	1
L2106P	B51	MMC	60	1.00	1
L2106P	B51	MMC	60	1.00	2
WT	B51	MMC	60	1.00	1
L2106P	B51	MMC	80	1.00	1
L2106P	B51	MMC	80	1.00	2
WT	B51	MMC	80	1.00	1
L2106P	B51	Cisp	0.2	85.03	1
L2106P	B51	Cisp	0.2	61.72	2
WT	B51	Cisp	0.2	78.17	1
L2106P	B51	Cisp	0.4	56.62	1
L2106P	B51	Cisp	0.4	27.61	2
WT	B51	Cisp	0.4	55.93	1
L2106P	B51	Cisp	0.6	27.49	1
L2106P	B51	Cisp	0.6	13.11	2
WT	B51	Cisp	0.6	30.48	1
L2106P	B51	Cisp	1	6.21	1
L2106P	B51	Cisp	1	1.04	2
WT	B51	Cisp	1	9.23	1
L2106P	B51	Cisp	1.2	4.58	1
L2106P	B51	Cisp	1.2	1.00	2
WT	B51	Cisp	1.2	5.93	1
L2106P	B51	Cisp	1.5	3.36	1
L2106P	B51	Cisp	1.5	3.02	2
WT	B51	Cisp	1.5	4.70	1
L2106P	B51	MMS	5	84.14	1
L2106P	B51	MMS	5	78.64	2
WT	B51	MMS	5	88.45	1
L2106P	B51	MMS	10	75.57	1
L2106P	B51	MMS	10	65.39	2
WT	B51	MMS	10	83.86	1
L2106P	B51	MMS	15	49.16	1
L2106P	B51	MMS	15	59.07	2
WT	B51	MMS	15	78.93	1
L2106P	B51	MMS	20	25.62	1

Variant[a]	Batch ID[b]	Drug[c]	Concentration[d]	Percent Survived[e]	Clone ID[f]
L2106P	B51	MMS	20	34.96	2
WT	B51	MMS	20	67.80	1
L2106P	B51	MMS	30	1.00	1
L2106P	B51	MMS	30	9.90	2
WT	B51	MMS	30	35.00	1
L2106P	B51	MMS	40	1.00	1
L2106P	B51	MMS	40	1.19	2
WT	B51	MMS	40	6.63	1
L2106P	B51	Parp	0.01	97.21	1
L2106P	B51	Parp	0.01	95.22	2
WT	B51	Parp	0.01	98.56	1
L2106P	B51	Parp	0.1	64.98	1
L2106P	B51	Parp	0.1	75.72	2
WT	B51	Parp	0.1	84.59	1
L2106P	B51	Parp	1	12.58	1
L2106P	B51	Parp	1	28.43	2
WT	B51	Parp	1	59.61	1
L2106P	B51	Parp	10	1.00	1
L2106P	B51	Parp	10	1.00	2
WT	B51	Parp	10	3.34	1
L2106P	B51	IR	50	79.75	1
L2106P	B51	IR	50	79.67	2
WT	B51	IR	50	89.09	1
L2106P	B51	IR	100	80.85	1
L2106P	B51	IR	100	73.60	2
WT	B51	IR	100	68.18	1
L2106P	B51	IR	200	51.41	1
L2106P	B51	IR	200	54.55	2
WT	B51	IR	200	60.06	1
L2106P	B51	IR	400	31.16	1
L2106P	B51	IR	400	32.93	2
WT	B51	IR	400	42.73	1
L2106P	B51	IR	600	19.31	1
L2106P	B51	IR	600	16.38	2
WT	B51	IR	600	24.68	1
S2152Y	B52	Camp	2.5	98.52	1
S2152Y	B52	Camp	2.5	98.65	2
WT	B52	Camp	2.5	100.00	1
S2152Y	B52	Camp	5	94.57	1
S2152Y	B52	Camp	5	94.34	2

Variant[a]	Batch ID[b]	Drug[c]	Concentration[d]	Percent Survived[e]	Clone ID[f]
WT	B52	Camp	5	97.50	1
S2152Y	B52	Camp	25	59.43	1
S2152Y	B52	Camp	25	69.16	2
WT	B52	Camp	25	77.81	1
S2152Y	B52	Camp	50	24.78	1
S2152Y	B52	Camp	50	38.73	2
WT	B52	Camp	50	37.45	1
S2152Y	B52	Camp	100	6.78	1
S2152Y	B52	Camp	100	5.42	2
WT	B52	Camp	100	3.50	1
S2152Y	B52	Camp	200	2.47	1
S2152Y	B52	Camp	200	1.59	2
WT	B52	Camp	200	1.00	1
S2152Y	B52	MMC	5	67.04	1
S2152Y	B52	MMC	5	80.87	2
WT	B52	MMC	5	75.57	1
S2152Y	B52	MMC	10	53.43	1
S2152Y	B52	MMC	10	82.65	2
WT	B52	MMC	10	74.07	1
S2152Y	B52	MMC	20	30.42	1
S2152Y	B52	MMC	20	54.16	2
WT	B52	MMC	20	61.17	1
S2152Y	B52	MMC	40	10.73	1
S2152Y	B52	MMC	40	29.38	2
WT	B52	MMC	40	29.19	1
S2152Y	B52	MMC	60	3.87	1
S2152Y	B52	MMC	60	12.03	2
WT	B52	MMC	60	9.97	1
S2152Y	B52	MMC	80	1.55	1
S2152Y	B52	MMC	80	5.25	2
WT	B52	MMC	80	3.52	1
S2152Y	B52	Cisp	0.2	46.89	1
S2152Y	B52	Cisp	0.2	67.04	2
WT	B52	Cisp	0.2	66.60	1
S2152Y	B52	Cisp	0.4	19.88	1
S2152Y	B52	Cisp	0.4	47.44	2
WT	B52	Cisp	0.4	43.04	1
S2152Y	B52	Cisp	0.6	13.05	1
S2152Y	B52	Cisp	0.6	32.54	2
WT	B52	Cisp	0.6	19.82	1

Variant[a]	Batch ID[b]	Drug[c]	Concentration[d]	Percent Survived[e]	Clone ID[f]
S2152Y	B52	Cisp	1	6.01	1
S2152Y	B52	Cisp	1	12.01	2
WT	B52	Cisp	1	9.70	1
S2152Y	B52	Cisp	1.2	4.18	1
S2152Y	B52	Cisp	1.2	7.41	2
WT	B52	Cisp	1.2	6.38	1
S2152Y	B52	Cisp	1.5	2.75	1
S2152Y	B52	Cisp	1.5	5.28	2
WT	B52	Cisp	1.5	4.37	1
S2152Y	B52	MMS	5	80.46	1
S2152Y	B52	MMS	5	91.42	2
WT	B52	MMS	5	100.00	1
S2152Y	B52	MMS	10	58.37	1
S2152Y	B52	MMS	10	88.92	2
WT	B52	MMS	10	82.63	1
S2152Y	B52	MMS	15	26.31	1
S2152Y	B52	MMS	15	72.65	2
WT	B52	MMS	15	75.09	1
S2152Y	B52	MMS	20	19.67	1
S2152Y	B52	MMS	20	61.88	2
WT	B52	MMS	20	67.63	1
S2152Y	B52	MMS	30	2.17	1
S2152Y	B52	MMS	30	7.88	2
WT	B52	MMS	30	6.49	1
S2152Y	B52	MMS	40	1.00	1
S2152Y	B52	MMS	40	1.40	2
WT	B52	MMS	40	1.05	1
S2152Y	B52	Parp	0.01	53.92	1
S2152Y	B52	Parp	0.01	77.02	2
WT	B52	Parp	0.01	100.00	1
S2152Y	B52	Parp	0.1	33.18	1
S2152Y	B52	Parp	0.1	73.34	2
WT	B52	Parp	0.1	93.20	1
S2152Y	B52	Parp	1	1.91	1
S2152Y	B52	Parp	1	48.73	2
WT	B52	Parp	1	71.52	1
S2152Y	B52	Parp	10	1.00	1
S2152Y	B52	Parp	10	2.04	2
WT	B52	Parp	10	2.80	1
S2152Y	B52	IR	50	71.36	1

Variant[a]	Batch ID[b]	Drug[c]	Concentration[d]	Percent Survived[e]	Clone ID[f]
S2152Y	B52	IR	50	90.86	2
WT	B52	IR	50	89.71	1
S2152Y	B52	IR	100	77.76	1
S2152Y	B52	IR	100	98.96	2
WT	B52	IR	100	85.35	1
S2152Y	B52	IR	200	47.93	1
S2152Y	B52	IR	200	79.26	2
WT	B52	IR	200	60.77	1
S2152Y	B52	IR	400	22.83	1
S2152Y	B52	IR	400	47.58	2
WT	B52	IR	400	36.92	1
S2152Y	B52	IR	600	18.21	1
S2152Y	B52	IR	600	29.96	2
WT	B52	IR	600	16.28	1
D1781G	B53	Camp	2.5	100.00	1
D1781G	B53	Camp	2.5	100.00	2
WT	B53	Camp	2.5	97.00	1
D1781G	B53	Camp	5	100.00	1
D1781G	B53	Camp	5	100.00	2
WT	B53	Camp	5	95.00	1
D1781G	B53	Camp	25	88.00	1
D1781G	B53	Camp	25	89.00	2
WT	B53	Camp	25	72.00	1
D1781G	B53	Camp	50	60.00	1
D1781G	B53	Camp	50	53.00	2
WT	B53	Camp	50	52.00	1
D1781G	B53	Camp	100	13.00	1
D1781G	B53	Camp	100	5.00	2
WT	B53	Camp	100	13.00	1
D1781G	B53	Camp	200	4.00	1
D1781G	B53	Camp	200	3.00	2
WT	B53	Camp	200	2.00	1
D1781G	B53	MMC	5	90.00	1
D1781G	B53	MMC	5	100.00	2
WT	B53	MMC	5	100.00	1
D1781G	B53	MMC	10	93.00	1
D1781G	B53	MMC	10	100.00	2
WT	B53	MMC	10	100.00	1
D1781G	B53	MMC	20	84.00	1
D1781G	B53	MMC	20	100.00	2

Variant[a]	Batch ID[b]	Drug[c]	Concentration[d]	Percent Survived[e]	Clone ID[f]
WT	B53	MMC	20	89.00	1
D1781G	B53	MMC	40	36.00	1
D1781G	B53	MMC	40	55.00	2
WT	B53	MMC	40	55.00	1
D1781G	B53	MMC	60	11.00	1
D1781G	B53	MMC	60	16.00	2
WT	B53	MMC	60	28.00	1
D1781G	B53	MMC	80	6.00	1
D1781G	B53	MMC	80	6.00	2
WT	B53	MMC	80	16.00	1
D1781G	B53	Cisp	0.2	69.00	1
D1781G	B53	Cisp	0.2	77.00	2
WT	B53	Cisp	0.2	78.00	1
D1781G	B53	Cisp	0.4	45.00	1
D1781G	B53	Cisp	0.4	62.00	2
WT	B53	Cisp	0.4	67.00	1
D1781G	B53	Cisp	0.6	30.00	1
D1781G	B53	Cisp	0.6	38.00	2
WT	B53	Cisp	0.6	59.00	1
D1781G	B53	Cisp	1	12.00	1
D1781G	B53	Cisp	1	15.00	2
WT	B53	Cisp	1	45.00	1
D1781G	B53	Cisp	1.2	7.00	1
D1781G	B53	Cisp	1.2	7.00	2
WT	B53	Cisp	1.2	33.00	1
D1781G	B53	Cisp	1.5	5.00	1
D1781G	B53	Cisp	1.5	4.00	2
WT	B53	Cisp	1.5	30.00	1
D1781G	B53	MMS	5	97.00	1
D1781G	B53	MMS	5	93.00	2
WT	B53	MMS	5	100.00	1
D1781G	B53	MMS	10	100.00	1
D1781G	B53	MMS	10	87.00	2
WT	B53	MMS	10	100.00	1
D1781G	B53	MMS	15	100.00	1
D1781G	B53	MMS	15	85.00	2
WT	B53	MMS	15	95.00	1
D1781G	B53	MMS	20	89.00	1
D1781G	B53	MMS	20	47.00	2
WT	B53	MMS	20	76.00	1

Variant[a]	Batch ID[b]	Drug[c]	Concentration[d]	Percent Survived[e]	Clone ID[f]
D1781G	B53	MMS	30	24.00	1
D1781G	B53	MMS	30	9.00	2
WT	B53	MMS	30	47.00	1
D1781G	B53	MMS	40	6.00	1
D1781G	B53	MMS	40	3.00	2
WT	B53	MMS	40	21.00	1
D1781G	B53	Parp	0.01	92.39	1
D1781G	B53	Parp	0.01	82.45	2
WT	B53	Parp	0.01	86.37	1
D1781G	B53	Parp	0.1	68.35	1
D1781G	B53	Parp	0.1	61.08	2
WT	B53	Parp	0.1	71.32	1
D1781G	B53	Parp	1	34.05	1
D1781G	B53	Parp	1	31.28	2
WT	B53	Parp	1	50.27	1
D1781G	B53	Parp	10	3.62	1
D1781G	B53	Parp	10	4.69	2
WT	B53	Parp	10	2.08	1
D1781G	B53	IR	50	81.00	1
D1781G	B53	IR	50	83.00	2
WT	B53	IR	50	84.40	1
D1781G	B53	IR	100	55.00	1
D1781G	B53	IR	100	58.00	2
WT	B53	IR	100	60.57	1
D1781G	B53	IR	200	41.00	1
D1781G	B53	IR	200	43.00	2
WT	B53	IR	200	54.17	1
D1781G	B53	IR	400	17.00	1
D1781G	B53	IR	400	16.00	2
WT	B53	IR	400	22.95	1
D1781G	B53	IR	600	13.00	1
D1781G	B53	IR	600	11.00	2
WT	B53	IR	600	13.08	1
T1302del	B54	Camp	2.5	98.00	1
T1302del	B54	Camp	2.5	98.00	2
WT	B54	Camp	2.5	97.00	1
T1302del	B54	Camp	5	100.00	1
T1302del	B54	Camp	5	96.00	2
WT	B54	Camp	5	95.00	1
T1302del	B54	Camp	25	67.00	1

Variant[a]	Batch ID[b]	Drug[c]	Concentration[d]	Percent Survived[e]	Clone ID[f]
T1302del	B54	Camp	25	76.00	2
WT	B54	Camp	25	72.00	1
T1302del	B54	Camp	50	39.00	1
T1302del	B54	Camp	50	34.00	2
WT	B54	Camp	50	52.00	1
T1302del	B54	Camp	100	5.00	1
T1302del	B54	Camp	100	3.00	2
WT	B54	Camp	100	13.00	1
T1302del	B54	Camp	200	2.00	1
T1302del	B54	Camp	200	1.00	2
WT	B54	Camp	200	2.00	1
T1302del	B54	MMC	5	100.00	1
T1302del	B54	MMC	5	95.00	2
WT	B54	MMC	5	100.00	1
T1302del	B54	MMC	10	80.00	1
T1302del	B54	MMC	10	98.00	2
WT	B54	MMC	10	100.00	1
T1302del	B54	MMC	20	70.00	1
T1302del	B54	MMC	20	100.00	2
WT	B54	MMC	20	89.00	1
T1302del	B54	MMC	40	25.00	1
T1302del	B54	MMC	40	44.00	2
WT	B54	MMC	40	55.00	1
T1302del	B54	MMC	60	6.00	1
T1302del	B54	MMC	60	9.00	2
WT	B54	MMC	60	28.00	1
T1302del	B54	MMC	80	6.00	1
T1302del	B54	MMC	80	4.00	2
WT	B54	MMC	80	16.00	1
T1302del	B54	Cisp	0.2	43.00	1
T1302del	B54	Cisp	0.2	63.00	2
WT	B54	Cisp	0.2	78.00	1
T1302del	B54	Cisp	0.4	19.00	1
T1302del	B54	Cisp	0.4	33.00	2
WT	B54	Cisp	0.4	67.00	1
T1302del	B54	Cisp	0.6	10.00	1
T1302del	B54	Cisp	0.6	34.00	2
WT	B54	Cisp	0.6	59.00	1
T1302del	B54	Cisp	1	4.00	1
T1302del	B54	Cisp	1	11.00	2

Variant[a]	Batch ID[b]	Drug[c]	Concentration[d]	Percent Survived[e]	Clone ID[f]
WT	B54	Cisp	1	45.00	1
T1302del	B54	Cisp	1.2	3.00	1
T1302del	B54	Cisp	1.2	6.00	2
WT	B54	Cisp	1.2	33.00	1
T1302del	B54	Cisp	1.5	2.00	1
T1302del	B54	Cisp	1.5	3.00	2
WT	B54	Cisp	1.5	30.00	1
T1302del	B54	MMS	5	85.00	1
T1302del	B54	MMS	5	100.00	2
WT	B54	MMS	5	100.00	1
T1302del	B54	MMS	10	74.00	1
T1302del	B54	MMS	10	100.00	2
WT	B54	MMS	10	100.00	1
T1302del	B54	MMS	15	50.00	1
T1302del	B54	MMS	15	85.00	2
WT	B54	MMS	15	95.00	1
T1302del	B54	MMS	20	26.00	1
T1302del	B54	MMS	20	53.00	2
WT	B54	MMS	20	76.00	1
T1302del	B54	MMS	30	6.00	1
T1302del	B54	MMS	30	10.00	2
WT	B54	MMS	30	47.00	1
T1302del	B54	MMS	40	2.00	1
T1302del	B54	MMS	40	3.00	2
WT	B54	MMS	40	21.00	1
T1302del	B54	Parp	0.01	94.00	1
T1302del	B54	Parp	0.01	82.00	2
WT	B54	Parp	0.01	100.00	1
T1302del	B54	Parp	0.1	63.00	1
T1302del	B54	Parp	0.1	52.00	2
WT	B54	Parp	0.1	91.00	1
T1302del	B54	Parp	1	33.00	1
T1302del	B54	Parp	1	25.00	2
WT	B54	Parp	1	80.00	1
T1302del	B54	Parp	10	5.00	1
T1302del	B54	Parp	10	1.00	2
WT	B54	Parp	10	14.00	1
T1302del	B54	IR	50	100.00	1
T1302del	B54	IR	50	86.00	2
WT	B54	IR	50	84.40	1

Variant[a]	Batch ID[b]	Drug[c]	Concentration[d]	Percent Survived[e]	Clone ID[f]
T1302del	B54	IR	100	77.00	1
T1302del	B54	IR	100	62.00	2
WT	B54	IR	100	60.57	1
T1302del	B54	IR	200	42.00	1
T1302del	B54	IR	200	55.00	2
WT	B54	IR	200	54.17	1
T1302del	B54	IR	400	22.00	1
T1302del	B54	IR	400	17.00	2
WT	B54	IR	400	22.95	1
T1302del	B54	IR	600	17.00	1
T1302del	B54	IR	600	13.00	2
WT	B54	IR	600	13.08	1
M1300I+1301 Tdel	B55	Camp	2.5	100.00	1
M1300I+1301 Tdel	B55	Camp	2.5	100.00	2
WT	B55	Camp	2.5	97.00	1
M1300I+1301 Tdel	B55	Camp	5	100.00	1
M1300I+1301 Tdel	B55	Camp	5	100.00	2
WT	B55	Camp	5	95.00	1
M1300I+1301 Tdel	B55	Camp	25	100.00	1
M1300I+1301 Tdel	B55	Camp	25	100.00	2
WT	B55	Camp	25	72.00	1
M1300I+1301 Tdel	B55	Camp	50	68.00	1
M1300I+1301 Tdel	B55	Camp	50	61.00	2
WT	B55	Camp	50	52.00	1
M1300I+1301 Tdel	B55	Camp	100	13.00	1
M1300I+1301 Tdel	B55	Camp	100	15.00	2
WT	B55	Camp	100	13.00	1
M1300I+1301 Tdel	B55	Camp	200	2.00	1
M1300I+1301 Tdel	B55	Camp	200	2.00	2
WT	B55	Camp	200	2.00	1
M1300I+1301 Tdel	B55	MMC	5	100.00	1
M1300I+1301 Tdel	B55	MMC	5	97.00	2
WT	B55	MMC	5	100.00	1
M1300I+1301 Tdel	B55	MMC	10	100.00	1

Variant[a]	Batch ID[b]	Drug[c]	Concentration[d]	Percent Survived[e]	Clone ID[f]
M1300I+1301 Tdel	B55	MMC	10	86.00	2
WT	B55	MMC	10	100.00	1
M1300I+1301 Tdel	B55	MMC	20	100.00	1
M1300I+1301 Tdel	B55	MMC	20	88.00	2
WT	B55	MMC	20	89.00	1
M1300I+1301 Tdel	B55	MMC	40	37.00	1
M1300I+1301 Tdel	B55	MMC	40	22.00	2
WT	B55	MMC	40	55.00	1
M1300I+1301 Tdel	B55	MMC	60	12.00	1
M1300I+1301 Tdel	B55	MMC	60	7.00	2
WT	B55	MMC	60	28.00	1
M1300I+1301 Tdel	B55	MMC	80	6.00	1
M1300I+1301 Tdel	B55	MMC	80	5.00	2
WT	B55	MMC	80	16.00	1
M1300I+1301 Tdel	B55	Cisp	0.2	73.00	1
M1300I+1301 Tdel	B55	Cisp	0.2	71.00	2
WT	B55	Cisp	0.2	78.00	1
M1300I+1301 Tdel	B55	Cisp	0.4	38.00	1
M1300I+1301 Tdel	B55	Cisp	0.4	33.00	2
WT	B55	Cisp	0.4	67.00	1
M1300I+1301 Tdel	B55	Cisp	0.6	16.00	1
M1300I+1301 Tdel	B55	Cisp	0.6	19.00	2
WT	B55	Cisp	0.6	59.00	1
M1300I+1301 Tdel	B55	Cisp	1	7.00	1
M1300I+1301 Tdel	B55	Cisp	1	6.00	2
WT	B55	Cisp	1	45.00	1
M1300I+1301 Tdel	B55	Cisp	1.2	5.00	1
M1300I+1301 Tdel	B55	Cisp	1.2	5.00	2
WT	B55	Cisp	1.2	33.00	1
M1300I+1301 Tdel	B55	Cisp	1.5	3.00	1
M1300I+1301 Tdel	B55	Cisp	1.5	3.00	2

Variant[a]	Batch ID[b]	Drug[c]	Concentration[d]	Percent Survived[e]	Clone ID[f]
WT	B55	Cisp	1.5	30.00	1
M1300I+1301 Tdel	B55	MMS	5	100.00	1
M1300I+1301 Tdel	B55	MMS	5	100.00	2
WT	B55	MMS	5	100.00	1
M1300I+1301 Tdel	B55	MMS	10	100.00	1
M1300I+1301 Tdel	B55	MMS	10	100.00	2
WT	B55	MMS	10	100.00	1
M1300I+1301 Tdel	B55	MMS	15	100.00	1
M1300I+1301 Tdel	B55	MMS	15	100.00	2
WT	B55	MMS	15	95.00	1
M1300I+1301 Tdel	B55	MMS	20	71.00	1
M1300I+1301 Tdel	B55	MMS	20	64.00	2
WT	B55	MMS	20	76.00	1
M1300I+1301 Tdel	B55	MMS	30	17.00	1
M1300I+1301 Tdel	B55	MMS	30	15.00	2
WT	B55	MMS	30	47.00	1
M1300I+1301 Tdel	B55	MMS	40	7.00	1
M1300I+1301 Tdel	B55	MMS	40	5.00	2
WT	B55	MMS	40	21.00	1
M1300I+1301 Tdel	B55	Parp	0.01	100.00	1
M1300I+1301 Tdel	B55	Parp	0.01	100.00	2
WT	B55	Parp	0.01	100.00	1
M1300I+1301 Tdel	B55	Parp	0.1	100.00	1
M1300I+1301 Tdel	B55	Parp	0.1	100.00	2
WT	B55	Parp	0.1	91.00	1
M1300I+1301 Tdel	B55	Parp	1	61.00	1
M1300I+1301 Tdel	B55	Parp	1	49.00	2
WT	B55	Parp	1	80.00	1
M1300I+1301 Tdel	B55	Parp	10	4.00	1
M1300I+1301 Tdel	B55	Parp	10	3.00	2
WT	B55	Parp	10	14.00	1

Variant[a]	Batch ID[b]	Drug[c]	Concentration[d]	Percent Survived[e]	Clone ID[f]
M1300I+1301 Tdel	B55	IR	50	100.00	1
M1300I+1301 Tdel	B55	IR	50	1.45	2
WT	B55	IR	50	86.00	1
M1300I+1301 Tdel	B55	IR	100	77.00	1
M1300I+1301 Tdel	B55	IR	100	1.32	2
WT	B55	IR	100	62.00	1
M1300I+1301 Tdel	B55	IR	200	42.00	1
M1300I+1301 Tdel	B55	IR	200	1.46	2
WT	B55	IR	200	55.00	1
M1300I+1301 Tdel	B55	IR	400	22.00	1
M1300I+1301 Tdel	B55	IR	400	1.00	2
WT	B55	IR	400	17.00	1
M1300I+1301 Tdel	B55	IR	600	17.00	1
M1300I+1301 Tdel	B55	IR	600	1.00	2
WT	B55	IR	600	13.00	1
N277K	B57	Camp	2.5	100.00	1
N277K	B57	Camp	2.5	100.00	2
WT	B57	Camp	2.5	92.00	1
N277K	B57	Camp	5	99.00	1
N277K	B57	Camp	5	100.00	2
WT	B57	Camp	5	83.00	1
N277K	B57	Camp	25	83.00	1
N277K	B57	Camp	25	94.00	2
WT	B57	Camp	25	69.00	1
N277K	B57	Camp	50	56.00	1
N277K	B57	Camp	50	55.00	2
WT	B57	Camp	50	52.00	1
N277K	B57	Camp	100	10.00	1
N277K	B57	Camp	100	10.00	2
WT	B57	Camp	100	10.00	1
N277K	B57	Camp	200	3.00	1
N277K	B57	Camp	200	1.00	2
WT	B57	Camp	200	2.00	1
N277K	B57	MMC	5	100.00	1
N277K	B57	MMC	5	89.00	2
WT	B57	MMC	5	100.00	1

Variant[a]	Batch ID[b]	Drug[c]	Concentration[d]	Percent Survived[e]	Clone ID[f]
N277K	B57	MMC	10	95.00	1
N277K	B57	MMC	10	98.00	2
WT	B57	MMC	10	100.00	1
N277K	B57	MMC	20	82.00	1
N277K	B57	MMC	20	78.00	2
WT	B57	MMC	20	89.00	1
N277K	B57	MMC	40	37.00	1
N277K	B57	MMC	40	31.00	2
WT	B57	MMC	40	51.00	1
N277K	B57	MMC	60	13.00	1
N277K	B57	MMC	60	11.00	2
WT	B57	MMC	60	37.00	1
N277K	B57	MMC	80	5.00	1
N277K	B57	MMC	80	7.00	2
WT	B57	MMC	80	19.00	1
N277K	B57	Cisp	0.2	100.00	1
N277K	B57	Cisp	0.2	77.00	2
WT	B57	Cisp	0.2	86.00	1
N277K	B57	Cisp	0.4	60.00	1
N277K	B57	Cisp	0.4	51.00	2
WT	B57	Cisp	0.4	70.00	1
N277K	B57	Cisp	0.6	32.00	1
N277K	B57	Cisp	0.6	27.00	2
WT	B57	Cisp	0.6	66.00	1
N277K	B57	Cisp	1	10.00	1
N277K	B57	Cisp	1	11.00	2
WT	B57	Cisp	1	37.00	1
N277K	B57	Cisp	1.2	5.00	1
N277K	B57	Cisp	1.2	6.00	2
WT	B57	Cisp	1.2	28.00	1
N277K	B57	Cisp	1.5	4.00	1
N277K	B57	Cisp	1.5	4.00	2
WT	B57	Cisp	1.5	24.00	1
N277K	B57	MMS	5	90.00	1
N277K	B57	MMS	5	92.00	2
WT	B57	MMS	5	93.00	1
N277K	B57	MMS	10	88.00	1
N277K	B57	MMS	10	79.00	2
WT	B57	MMS	10	91.00	1
N277K	B57	MMS	15	70.00	1

Variant[a]	Batch ID[b]	Drug[c]	Concentration[d]	Percent Survived[e]	Clone ID[f]
N277K	B57	MMS	15	61.00	2
WT	B57	MMS	15	86.00	1
N277K	B57	MMS	20	48.00	1
N277K	B57	MMS	20	51.00	2
WT	B57	MMS	20	73.00	1
N277K	B57	MMS	30	11.00	1
N277K	B57	MMS	30	12.00	2
WT	B57	MMS	30	44.00	1
N277K	B57	MMS	40	3.00	1
N277K	B57	MMS	40	4.00	2
WT	B57	MMS	40	23.00	1
N277K	B57	Parp	0.01	91.00	1
N277K	B57	Parp	0.01	100.00	2
WT	B57	Parp	0.01	93.00	1
N277K	B57	Parp	0.1	76.00	1
N277K	B57	Parp	0.1	96.00	2
WT	B57	Parp	0.1	93.00	1
N277K	B57	Parp	1	47.00	1
N277K	B57	Parp	1	68.00	2
WT	B57	Parp	1	81.00	1
N277K	B57	Parp	10	4.00	1
N277K	B57	Parp	10	8.00	2
WT	B57	Parp	10	11.00	1
N277K	B57	IR	50	87.00	1
N277K	B57	IR	50	93.00	2
WT	B57	IR	50	88.00	1
N277K	B57	IR	100	83.00	1
N277K	B57	IR	100	77.00	2
WT	B57	IR	100	74.00	1
N277K	B57	IR	200	69.00	1
N277K	B57	IR	200	70.00	2
WT	B57	IR	200	63.00	1
N277K	B57	IR	400	47.00	1
N277K	B57	IR	400	37.00	2
WT	B57	IR	400	46.00	1
N277K	B57	IR	600	42.00	1
N277K	B57	IR	600	35.00	2
WT	B57	IR	600	43.00	1

[a]*BRCA2* variant used in the drug sensitivity assay (DS)

[b]Batch number indicates samples analyzed in one set with common WT control

[c]DNA damaging agent used (Camp: camptothecin; MMC: mitomycin C; Cisp: Cisplatin; MMS: methyl methanesulfonate; Parp: PAPR inhibitor olaparib; IR: γ-irradiation

[d]Concentration used, Camp in µM; MMC in ng/ml; Cisp in µM; MMS in µg/ml; Parp in µM; IR in RAD. NOTE: cells were treated in triplicate with each drug at each concentration/dose

[e]Percent Survived indicates cell survival relative to untreated cells.

[f]Clone ID refers to the two clones expressing the same variant

Supplementary Table 4: List of PCR primers used in the study and their sequences and location.

**Primers**	**Sequence (5´-3´)**	**Used for alternative splicing analysis**
Ex2-RT-Fwd	GACACGCTGCAACAAAGCA	V220Ifs
Ex10-RT-Rev	CAGCGTTTGCTTCATGGA	V220Ifs
BRCA2-11F	TGGTTTTGTCAAATTCAAGAATTGG	R2336L, R2336P
BRCA2-14R	GTGAAAGTTACAGCTACTGCTTGATTGG	R2336L, R2336P
Exon 11-RT-Fwd	AACAGAAGCAGTAGAAATTGC	G2281V, D2312V, D2312E
Exon 14 RT-Rev	AACAAAGACTTTGGTTGGTCTGCC	G2281V, D2312V, D2312E
Exon 15-RT-Fwd	TGATGGTGGATGGCTCATACC	R2659G, R2659K, R2659T, S2691F
Exon 19 RT-Rev	GAATAATTACATCAACACAACC	R2659G, R2659K, R2659T, S2691F
ex15-Fwd-3	AAGTTCCCTCTGCGTGTTCT	T2798Nfs
Ex20-Rev2	GTCTCTTTTGTTGGGCCTCCA	T2798Nfs
Ex17-RT-Fwd	AAGGAATTTGCTAATAGATGCC	Q2829L, Q2829R, Q2829X
Ex22-RT-Rev	ATTATTCAAGGCTCTTAACTGC	Q2829L, Q2829R, Q2829X
Ex23-RT-For	CATACAGTTAGCAGCGAC	C3233Wfs
Ex27-RT-Rev	CAACTCCTTGGTGGCTGAAA	C3233Wfs

Supplemental Methods

ImageJ Script for colony counting

Script #1, for getting threshold by clicking on the dimmest colony pre-selected by user

```
// ************************* BEFORE RUNNING THE SCRIPT
*************************
// Walls region of the petri dish should be filled with background color in ImageJ
// The background color should be picked from INSIDE of petri dish before processing:
// otherwise dark spot created by walls shadow will contribute to colonies count.
// The image window to work with needs to be renamed as "A" before running this script.
// Image should be zoomed in to see representative lowest intensity colony in high magnification.
// ************************* USER ADJUSTABLE PARAMETERS
************************
// Two parameters below depend on the sample type and dpi settings of the scanner.
// Both need to be adjusted in "trial and error" mode for each dataset.
// Set the size of Gaussian filter for background subtraction.
// It should be bigger than any colony of interest
Blur_for_background = 200; // in pixels
// Set the size of the Gaussian filter to exclude very small spots merged together
 Blur_for_small_separation_rejection = 2;  // ~ to the minimal meaningful distance between two
colonies
//
*****************************************************************************
selectWindow("A");
// Rename window which will be used for clicking on "threshold" colony, remove XY
calibration.
rename("TO_CLICK");
run("Set Scale...", "distance=0 known=0 pixel=1 unit=pixel");
// Create a copy and converting it into a stack with 3 RGB colors separated
run("Duplicate...", "title=ABCDEFG duplicate");
run("Split Channels");
run("Images to Stack", "name=Stack_R_G_B title=ABCDEFG use");
run("Grays");
// Slices are arranged in RGB order, so the first slice will be "red". Blue dye absorbs red most,
// so that we use red for finding peaks. For different samples, another color may work better
setSlice(1); // red
// Original A image does not exist anymore, just reusing name
run("Duplicate...", "title=A");
// To find peaks, while colonies are actually dips in intensity, we need to flip black and white
run("Invert");
// Create window with approximation of background, and subtract it from the original image
run("Duplicate...", "title=Gauss");
run("Gaussian Blur...", "sigma=Blur_for_background");
imageCalculator("Subtract create", "A","Gauss");
```

```
// Blur background-free image so that very small features will not stand out
selectWindow("Result of A");
run("Gaussian Blur...", "sigma=Blur_for_small_separation_rejection");
rename("Image_A_filtered");
close("Gauss");
// Bring up original image zoomed out on the dimmest colony that counts, pre-selected to
represent threshold level
selectWindow("TO_CLICK");
showMessage("Press OK and then click TO_CLICK image exactly at center the dimmest peak
which still counts");
// Get user input. Variable x and y will contain coordinates of the first click.
leftButton=16;
getCursorLoc(x, y, z, flags);
while (flags&leftButton==0) {
   getCursorLoc(x, y, z, flags);
   wait(10); }
close();
// Recovering gray value from filtered gray image, which will be processed. Correcting for user's
1 pixel clicking error.
selectWindow("Image_A_filtered");
answer = getPixel(x, y); // Value of the clicked pixel. Will be compared to 8 nearest neighbors.
answer = maxOf(answer,getPixel(x+1, y+1));
answer = maxOf(answer,getPixel(x+1, y  ));
answer = maxOf(answer,getPixel(x+1, y-1));
answer = maxOf(answer,getPixel(x  , y+1));
answer = maxOf(answer,getPixel(x  , y-1));
answer = maxOf(answer,getPixel(x-1, y+1));
answer = maxOf(answer,getPixel(x-1, y  ));
answer = maxOf(answer,getPixel(x-1, y-1));
answer = answer -1; // to get the peak we clicked in
showMessage("The value used for thresholding is " + answer);
// Deleting everything below threshold. Because of integer type, all negative values are zeros.
run("Subtract...", "value=" + answer);
// Using "Find Maxima" build in ImageJ function, and creating a copy for printing
run("Find Maxima...", "noise=0 output=[Single Points]");
rename("To_count");
run("Duplicate...", "title=Maxima");
// Counting white pixels on BW image: the result will be an "Integrated Intensity"
selectWindow("To_count");
run("Divide...", "value=255");
run("Set Measurements...", "  integrated redirect=None decimal=0");
run("Measure");
selectWindow("Maxima");
run("Grays");
// Clean up
close("To_count"); close("Image_A_filtered"); close("A");
```

```
// Printing square around the colony used as a reference for finding minimal intensity.
selectWindow("Stack_R_G_B");
run("8-bit");
setSlice(1);
setForegroundColor(255, 255, 255);
setLineWidth(2);
drawRect(x-15, y-15, 30, 30);
// Prining dots in each channel, to show colonies which has been counted
imageCalculator("Subtract", "Stack_R_G_B" , "Maxima");
setSlice(2); // for green
imageCalculator("Add", "Stack_R_G_B" , "Maxima");
setSlice(3); // for blue
imageCalculator("Subtract", "Stack_R_G_B" , "Maxima");
run("Stack to RGB");
// Presenting final result, the number of colonies in the window name
title_string = "Number_of_peaks_" + getResultString("RawIntDen") +
"_when_processed_with_" + answer + "_at_cell_";
rename(title_string);
// Clean up
selectWindow("Results"); run("Close");
close("Maxima"); close("Stack_R_G_B");
```

Script # 2, for using threshold found by script # 1 on a different image

```
// This is the script to be used for all images of a dataset except the one processed with the script
#1.
// The threshold intensity produced by script #1, and representing the dimmest colony to be
counted, needs to be
// manually entered each time.
// Make sure that two parameters below are same as in script #1 version used.
Blur_for_background = 200;
Blur_for_small_separation_rejection = 2;
// *********************************************
selectWindow("A");
rename("ABCDEFG ");
run("Set Scale...", "distance=0 known=0 pixel=1 unit=pixel");
run("Split Channels");
run("Images to Stack", "name=Stack_R_G_B title=ABCDEFG use");
run("Grays");
setSlice(1);
run("Duplicate...", "title=A");
run("Invert");
run("Duplicate...", "title=Gauss");
run("Gaussian Blur...", "sigma=Blur_for_background");
imageCalculator("Subtract create", "A","Gauss");
selectWindow("Result of A");
```

```
run("Gaussian Blur...", "sigma=Blur_for_small_separation_rejection");
rename("Image_A_filtered");
close("Gauss");
// Ask user for threshold value
answer = getNumber("Please enter the number obtained from clicking on a spot", 100000);
// Proceed the same was as in script #1
run("Subtract...", "value=" + answer);
run("Find Maxima...", "noise=0 output=[Single Points]");
rename("To_count");
run("Duplicate...", "title=Maxima");
selectWindow("To_count");
run("Divide...", "value=255");
run("Set Measurements...", "  integrated redirect=None decimal=0");
run("Measure");
selectWindow("Maxima");
run("Grays");
close("To_count");
close("Image_A_filtered");
close("A");
selectWindow("Stack_R_G_B");
run("8-bit");
imageCalculator("Subtract", "Stack_R_G_B" , "Maxima");
setSlice(2); // for green
imageCalculator("Add", "Stack_R_G_B" , "Maxima");
setSlice(3); // for blue
imageCalculator("Subtract", "Stack_R_G_B" , "Maxima");
run("Stack to RGB");
title_string = "Number_of_peaks_" + getResultString("RawIntDen") +
"_when_processed_with_" + answer + "_at_cell_";
rename(title_string);
selectWindow("Results");
run("Close");
close("Maxima");
close("Stack_R_G_B");
```
