# Supplementary material for: A computational model for classification of BRCA2 variants using mouse embryonic stem cell-based functional assays
Source: NPJ Genom Med. 2020 Dec 8;5:52. doi: 10.1038/s41525-020-00158-5 (PMC7722754; doi:10.1038/s41525-020-00158-5)
Supplement: Supplementary file 2 — Reporting Summary Checklist [file 41525_2020_158_MOESM2_ESM.pdf]

nature research

Corresponding author(s): Edwin S. Iversen and Shyam K. Sharan

Last updated by author(s): Sep 30, 2020

Reporting Summary

Nature Research wishes to improve the reproducibility of the work that we publish. This form provides structure for consistency and transparency in reporting. For further information on Nature Research policies, see our Editorial Policies and the Editorial Policy Checklist.

Statistics

For all statistical analyses, confirm that the following items are present in the figure legend, table legend, main text, or Methods section.

n/a	Confirmed	
☐	☒	The exact sample size (*n*) for each experimental group/condition, given as a discrete number and unit of measurement
☐	☒	A statement on whether measurements were taken from distinct samples or whether the same sample was measured repeatedly
☒	☐	The statistical test(s) used AND whether they are one- or two-sided<br>*Only common tests should be described solely by name; describe more complex techniques in the Methods section.*
☐	☒	A description of all covariates tested
☐	☒	A description of any assumptions or corrections, such as tests of normality and adjustment for multiple comparisons
☐	☒	A full description of the statistical parameters including central tendency (e.g. means) or other basic estimates (e.g. regression coefficient) AND variation (e.g. standard deviation) or associated estimates of uncertainty (e.g. confidence intervals)
☒	☐	For null hypothesis testing, the test statistic (e.g. *F*, *t*, *r*) with confidence intervals, effect sizes, degrees of freedom and *P* value noted<br>*Give P values as exact values whenever suitable.*
☐	☒	For Bayesian analysis, information on the choice of priors and Markov chain Monte Carlo settings
☐	☒	For hierarchical and complex designs, identification of the appropriate level for tests and full reporting of outcomes
☐	☒	Estimates of effect sizes (e.g. Cohen's *d*, Pearson's *r*), indicating how they were calculated

Our web collection on statistics for biologists contains articles on many of the points above.

Software and code

Policy information about availability of computer code

Data collection	No software used.
Data analysis	All analyses were conducted in the R programming language, Version 3.6.3. The R code and appropriately formatted input data files are available upon request.

For manuscripts utilizing custom algorithms or software that are central to the research but not yet described in published literature, software must be made available to editors and reviewers. We strongly encourage code deposition in a community repository (e.g. GitHub). See the Nature Research guidelines for submitting code & software for further information.

Data

Policy information about availability of data

All manuscripts must include a data availability statement. This statement should provide the following information, where applicable:

- Accession codes, unique identifiers, or web links for publicly available datasets
- A list of figures that have associated raw data
- A description of any restrictions on data availability

The raw data are provided in Supplemental Tables 2 and 3.

Field-specific reporting

Please select the one below that is the best fit for your research. If you are not sure, read the appropriate sections before making your selection.

☒ Life sciences ☐ Behavioural & social sciences ☐ Ecological, evolutionary & environmental sciences

For a reference copy of the document with all sections, see nature.com/documents/nr-reporting-summary-flat.pdf

Life sciences study design

All studies must disclose on these points even when the disclosure is negative.

Sample size	Two independently generated mouse ES cell clones expressing each BRCA2 variants were used in every experiment. The reason for selecting two independent clones expressing each BRCA2 variant was to rule out any "position effect" related to the site of integration of the BAC in the mouse ES cell genomic DNA.
Data exclusions	No experimental data was excluded.
Replication	All Drug sensitivity assays were done in triplicate using two independent ES cell clones.<br>all cell viability assays were performed in two independently generated mES cells expressing each variant.
Randomization	Cells were randomly plated for drug sensitivity and HAT selection assay.
Blinding	All experiments were blindly performed by individuals who were not aware of the pathogenicity of the variants.

Reporting for specific materials, systems and methods

We require information from authors about some types of materials, experimental systems and methods used in many studies. Here, indicate whether each material, system or method listed is relevant to your study. If you are not sure if a list item applies to your research, read the appropriate section before selecting a response.

Materials & experimental systems

n/a	Involved in the study	
☐	☒	Antibodies
☐	☒	Eukaryotic cell lines
☒	☐	Palaeontology and archaeology
☒	☐	Animals and other organisms
☒	☐	Human research participants
☒	☐	Clinical data
☒	☐	Dual use research of concern

Methods

n/a	Involved in the study	
☒	☐	ChIP-seq
☒	☐	Flow cytometry
☐	☐	MRI-based neuroimaging

Antibodies

Antibodies used	Rabbit polyclonal BRCA2 (recognizes an epitope between residues 450-500) antibody (BETHYL lab, Cat # A303-434A-T-1), rabbit monoclonal GAPDH antibody (Cell Signaling technologies, Cat# 5174) and mouse monoclonal Vinculin antibody (Santa Cruz biotech, Cat# sc25336)<br>Rabbit polyclonal RAD51 antibody (Calbiochem, Cat# PC-130) and mouse monoclonal anti phospho-histone H2A.X (Millipore, Cat# 05-636)<br>Anti-BrdU antibody (mouse, #347580, Becton Dickinson) and Anti-BrdU antibody (rat, ab6326, Abcam)
Validation	Rabbit polyclonal BRCA2(BETHYL lab, Cat # A303-434A-T-1)<br>Application: Western Blot<br>Reference: Ubiquitinated-PCNA protects replication forks from DNA2-mediated degradation by regulating Okazaki fragment maturation and chromatin assembly.<br>In Nature Communications on 1 May 2020 by Thakar, T., Leung, W., et al..<br><br>Rabbit monoclonal GAPDH antibody (Cell Signaling technologies, Cat# 5174)<br>Application: Western blot<br>Reference: TNFAIP8 regulates autophagy, cell steatosis, and promotes hepatocellular carcinoma cell proliferation<br>In Cell Death Dis. 2020 Mar 9;11(3):178. doi: 10.1038/s41419-020-2369-4. by Niture et al.<br><br>Mouse monoclonal Vinculin antibody (Santa Cruz biotech, Cat# sc25336)<br>Application: Western blot<br>Reference: Interplay between c-Src and the APC/C co-activator Cdh1 regulates mammary tumorigenesis<br>In Nat Commun. 2019 Aug 16;10(1):3716. doi: 10.1038/s41467-019-11618-7. by Han et al.

Rabbit polyclonal RAD51 antibody (Calbiochem, Cat# PC-130)
Application: Immunofluorescence
Reference: CDK2 is required for proper homologous pairing, recombination and sex-body formation during male mouse meiosis
In J. cell Science 2009. 122, 2149. by Viera et al.

Mouse monoclonal anti phospho-histone H2A.X (Millipore, Cat# 05-636)
Application: Immunofluorescence
Reference: DNA damage signaling regulates age-dependent proliferative capacity of quiescent inner ear supporting cells
In Aging (Albany NY). 2014 Jun;6(6):496-510. doi: 10.18632/aging.100668. by Laos et al.

Anti-BrdU antibody (mouse, #347580, Becton Dickinson) and Anti-BrdU antibody (rat, ab6326, Abcam)
Application: Immunofluorescence
Reference: Replication fork stability confers chemoresistance in BRCA-deficient cells
In Nature. 2016 Jul 21;535(7612):382-7. doi: 10.1038/nature18325. by Ray Chaudhuri et al.

Eukaryotic cell lines

Policy information about cell lines

Cell line source(s)	PL2 F7 mouse embryonic stem cells.
Authentication	Reference: Mouse embryonic stem cell-based functional assay to evaluate mutations in BRCA2<br>In Nat Med. 2008 Aug;14(8):875-81. doi: 10.1038/nm.1719. Epub 2008 Jul 6. by Kuznetsov et al.
Mycoplasma contamination	Cell line were routinely tested and are Mycoplasma free
Commonly misidentified lines<br>(See ICLAC register)	n/a

Magnetic resonance imaging

Experimental design

Design type	N/A
Design specifications	*Specify the number of blocks, trials or experimental units per session and/or subject, and specify the length of each trial or block (if trials are blocked) and interval between trials.*
Behavioral performance measures	*State number and/or type of variables recorded (e.g. correct button press, response time) and what statistics were used to establish that the subjects were performing the task as expected (e.g. mean, range, and/or standard deviation across subjects).*

Acquisition

Imaging type(s)	*Specify: functional, structural, diffusion, perfusion.*
Field strength	*Specify in Tesla*
Sequence & imaging parameters	*Specify the pulse sequence type (gradient echo, spin echo, etc.), imaging type (EPI, spiral, etc.), field of view, matrix size, slice thickness, orientation and TE/TR/flip angle.*
Area of acquisition	*State whether a whole brain scan was used OR define the area of acquisition, describing how the region was determined.*

Diffusion MRI ☐ Used ☐ Not used

Preprocessing

Preprocessing software	*Provide detail on software version and revision number and on specific parameters (model/functions, brain extraction, segmentation, smoothing kernel size, etc.).*
Normalization	*If data were normalized/standardized, describe the approach(es): specify linear or non-linear and define image types used for transformation OR indicate that data were not normalized and explain rationale for lack of normalization.*
Normalization template	*Describe the template used for normalization/transformation, specifying subject space or group standardized space (e.g. original Talairach, MNI305, ICBM152) OR indicate that the data were not normalized.*
Noise and artifact removal	*Describe your procedure(s) for artifact and structured noise removal, specifying motion parameters, tissue signals and physiological signals (heart rate, respiration).*
Volume censoring	*Define your software and/or method and criteria for volume censoring, and state the extent of such censoring.*

Statistical modeling & inference

Model type and settings	*Specify type (mass univariate, multivariate, RSA, predictive, etc.) and describe essential details of the model at the first and second levels (e.g. fixed, random or mixed effects; drift or auto-correlation).*
Effect(s) tested	*Define precise effect in terms of the task or stimulus conditions instead of psychological concepts and indicate whether ANOVA or factorial designs were used.*

Specify type of analysis: ☐ Whole brain ☐ ROI-based ☐ Both

Statistic type for inference (See Eklund et al. 2016)	*Specify voxel-wise or cluster-wise and report all relevant parameters for cluster-wise methods.*
Correction	*Describe the type of correction and how it is obtained for multiple comparisons (e.g. FWE, FDR, permutation or Monte Carlo).*

Models & analysis

n/a	Involved in the study
☐	☐ Functional and/or effective connectivity
☐	☐ Graph analysis
☐	☐ Multivariate modeling or predictive analysis

Functional and/or effective connectivity	*Report the measures of dependence used and the model details (e.g. Pearson correlation, partial correlation, mutual information).*
Graph analysis	*Report the dependent variable and connectivity measure, specifying weighted graph or binarized graph, subject- or group-level, and the global and/or node summaries used (e.g. clustering coefficient, efficiency, etc.).*
Multivariate modeling and predictive analysis	*Specify independent variables, features extraction and dimension reduction, model, training and evaluation metrics.*
